# Strategy for Engineering High Photolysis Efficiency of Photocleavable Protecting Groups through Cation Stabilization

**DOI:** 10.1021/jacs.2c04262

**Published:** 2022-07-01

**Authors:** Albert M. Schulte, Georgios Alachouzos, Wiktor Szymański, Ben L. Feringa

**Affiliations:** † Centre for Systems Chemistry, Stratingh Institute for Chemistry, Faculty for Science and Engineering, University of Groningen, Nijenborgh 4, 9747 AG Groningen, The Netherlands; ‡ Department of Radiology, Medical Imaging Center, University Medical Center Groningen, University of Groningen, Hanzeplein 1, 9713 GZ Groningen, The Netherlands

## Abstract

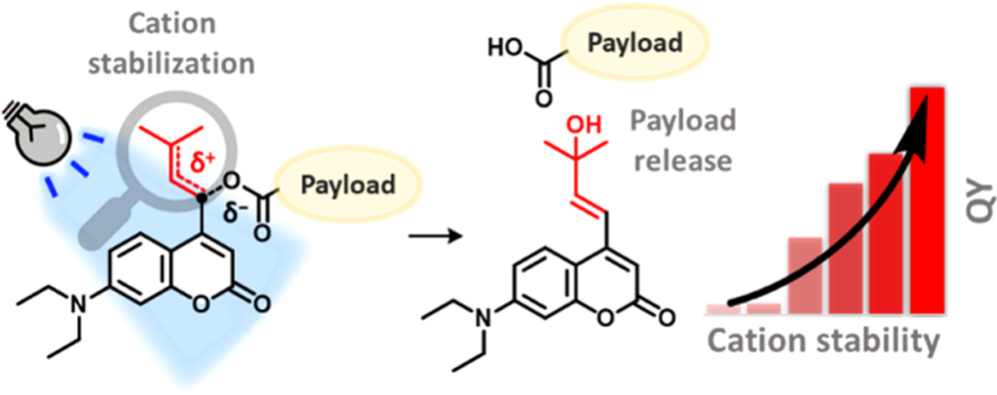

Photolabile protecting groups (PPGs) enable the precise activation of molecular function with light in many research areas, such as photopharmacology, where remote spatiotemporal control over the release of a molecule is needed. The design and application of PPGs in recent years have particularly focused on the development of molecules with high molar absorptivity at long irradiation wavelengths. However, a crucial parameter, which is pivotal to the efficiency of uncaging and which has until now proven highly challenging to improve, is the photolysis quantum yield (QY). Here, we describe a novel and general approach to greatly increase the photolysis QY of heterolytic PPGs through stabilization of an intermediate chromophore cation. When applied to coumarin PPGs, our strategy resulted in systems possessing an up to a 35-fold increase in QY and a convenient fluorescent readout during their uncaging, all while requiring the same number of synthetic steps for their preparation as the usual coumarin systems. We demonstrate that the same QY engineering strategy applies to different photolysis payloads and even different classes of PPGs. Furthermore, analysis of the DFT-calculated energy barriers in the first singlet excited state reveals valuable insights into the important factors that determine photolysis efficiency. The strategy reported herein will enable the development of efficient PPGs tailored for many applications.

## Introduction

Photolabile protecting groups (PPGs) are chemical moieties that can be synthetically introduced to a substrate of interest and subsequently be removed simply by irradiation with light of a suitable wavelength. Due to this unique property, they are widely used to remotely control the concentration of molecules with a particular function of interest (payloads)^1−6^ because they allow for high spatiotemporal control over the payload release. PPGs have proven their utility in a variety of fields, ranging from molecular biology and organic synthesis to materials science.^7−12^

A critical property fundamental to the applicability of PPGs is the efficiency of payload release. This efficiency is captured in the mathematical product of two factors: (i) the ability of the PPG to absorb photons at the irradiation wavelength (represented by the molar attenuation coefficient ε, M^–1^ cm^–1^) and (ii) the photochemical uncaging process quantum yield (φ, QY). In recent years, significant progress has been made in improving the first parameter, especially directed at the development of PPGs with high ε at bathochromically shifted irradiation wavelengths.^13−19^ However, thus far, enhancing the second parameter—the photochemical QY—has remained challenging. Given the role of QY in the overall efficiency of uncaging, a general strategy for reliably increasing QY is needed, and this would represent a major step forward for the field of PPG development.

The importance of photochemical quantum yield is exemplified in the field of photopharmacology, where PPGs are used to suppress the activity of a bioactive payload in the covalently bound photocaged form. Subsequent on-site release of the bioactive molecule is achieved through light irradiation, preventing systemic side effects in nonirradiated areas.^1,2,20−24^ The importance of molar absorptivity (ε) has been widely recognized in photopharmacology and has led to impressive progress for red- or green-light responsive PPGs featuring high ε, such as those derived from the BODIPY and coumarin scaffolds.^14,17−19,25^ However, in most cases, high ε at long irradiation wavelengths was achieved at the expense of photochemical QY, negatively affecting the overall efficiency of these PPGs.^26^ Few exceptions to this trend often rely on the introduction of extended π-systems with strongly electron-donating moieties into the PPG structure, yet these strategies come at a great synthetic step cost, and the flat, extended π-systems suffer from low aqueous solubility, restricting their use in photopharmacology.^27,28^

Here, we present a rational strategy for increasing PPG efficiency, hinging on a detailed investigation into the important factors that determine PPG QYs. Taking fundamental organic chemistry principles as the starting point and supporting them with a DFT computational approach, we focused on 7-diethylaminocoumarins as a model class of PPGs. The synthetic accessibility of coumarins and their visible light responsiveness^12,29^ have led to their privileged use for the photocaging of various functionalities, such as carboxylic acids, alcohols, and amines (as their carbonate and carbamate esters, respectively),^30^ making them an ideal showcase for our strategy. Furthermore, we also show that our strategy is general and extends to other classes of PPGs that utilize heterolytic bond cleavage in their photochemical step, as opposed to different classes of PPGs, such as *ortho*-nitrobenzyls, whose uncaging mechanism relies on other processes.^3^ Finally, we demonstrate how the fluorescence increase that we observe during the uncaging of the new subtype of PPGs introduced here can be used as a convenient readout of the progress of the uncaging process in a complex biological system.

### Mechanism of Coumarin PPG Photocleavage

**Scheme 1:**
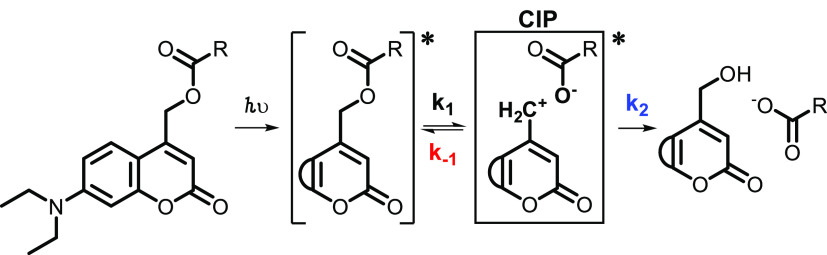
Schematic Representation of the Coumarin Photocleavage Mechanism Highlighting the Contact Ion Pair (CIP) Intermediate Shown is a model coumarin with a model carboxylic acid payload, in which the fused aromatic ring of the coumarin chromophore is abbreviated by a semicircle.

The mechanism of photocleavage of coumarin PPGs relies on heterolytic bond cleavage in the first singlet excited state (S_1_).^30^ After photon absorption to S_1_, weakening of the bond between the payload and the α-carbon allows for the excited state breakage of this bond (Scheme 1, k_1_).^30,31^ Upon breakage of the payload-PPG bond, a contact ion pair (CIP) intermediate is formed, in which the positive charge is located on the coumarin chromophore and the negative charge on the payload.^30−33^ The formed CIP intermediate can now undergo two different processes: (i) the unproductive process, i.e., recombination to reform the substrate (Scheme 1, red k_–1_), or (ii) the productive process that results in payload release: diffusion to a solvent separated ion pair (SSIP, not shown) accompanied by solvent trapping to quench the carbocation (Scheme 1, both processes combined in blue k_2_).

Lowering the energy of the CIP through the use of payloads that effectively stabilize the generated negative charge (i.e., payloads with a low pKa of their conjugate acid) has been shown to improve photochemical QY.^31^ However, engineering an increase of the QY in this manner is impractical or often even impossible, since the stability of the negative charge is inherent to the payload and cannot be conveniently engineered without compromising other payload chemical properties (e.g., its bioactivity). On the other hand, the stabilization of the other component of the CIP, the PPG chromophore cation, represents an underexplored and potentially superior approach in that it would offer a general strategy for increasing the QY of PPGs that rely on heterolytic bond cleavage, independent of the chemical nature of the payload.

### Design

Although CIP formation is a fast process in coumarin PPGs, recombination of the CIP (k_–1_) is significantly faster than the competing productive pathway k_2_.^31^ Based on this observation, it was deduced that rather than promoting heterolysis toward CIP formation, slowing down CIP recombination itself (i.e., lowering k_–1_) would be a viable strategy to significantly increase PPG photochemical QY.

**Figure 1:**
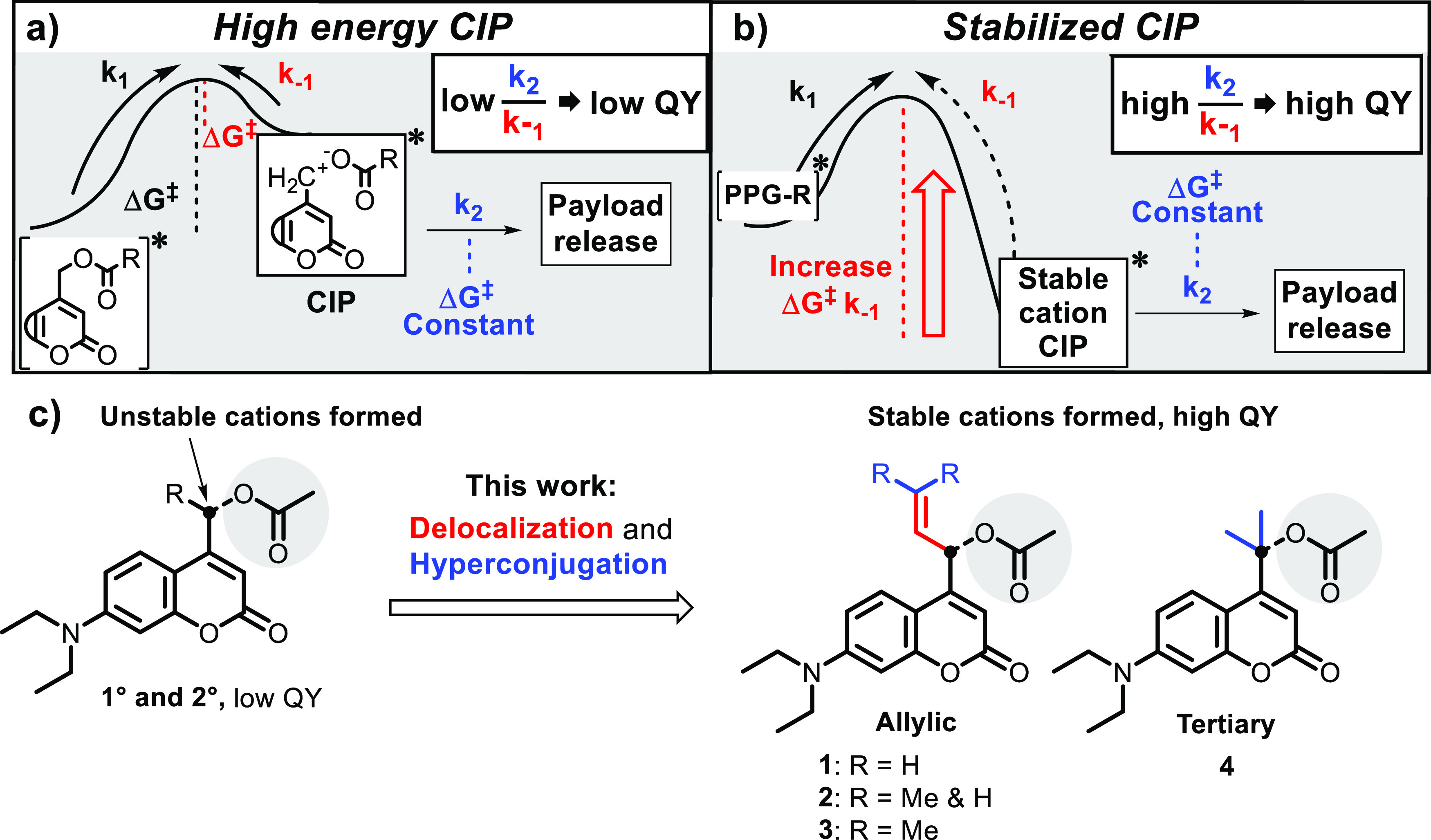
(a) Energy diagram of the coumarin photolysis mechanism after excitation, showing an unstable CIP intermediate resulting in a high rate of CIP recombination, a relatively low rate of the productive step (k_2_), and consequentially a low photochemical QY. (b) A stabilized CIP intermediate, reducing the rate of CIP recombination and consequentially increasing the photochemical QY. (c) Coumarin PPGs bearing substituents on the α-carbon (black) stabilizing the cationic intermediate through delocalization and hyperconjugation, as described in this paper. All coumarins are caging an acetic acid payload (gray).

In the coumarin photocleavage process, the positive charge on the α-carbon (adjacent to the payload, Scheme 1) in the CIP intermediate cannot be efficiently stabilized through delocalization or hyperconjugation. Being a primary cation, it is expected to be high in energy, favoring CIP recombination back to the substrate rather than the productive step (Figure 1a, k_–1_ and k_2_, respectively). Based on this simple analysis, we sought to increase photolysis efficiency through direct stabilization of this cationic intermediate, thereby retarding its unproductive recombination by increasing the kinetic barrier for this process (Figure 1a vs 1b, k_–1_). We hypothesized that the rate of CIP diffusion followed by solvent trapping (Figure 1, k_2_) depends on constant coulombic forces and will be independent of the overall CIP stability. Therefore, while stabilization of the cationic intermediate will increase the barrier of the unproductive step k_–1_, it will not increase the barrier of the productive step k_2_. Consequentially, it will result in a higher ratio of the rates of k_2_ over k_–1_, increasing the overall PPG photochemical QY (Figure 1b).

In fact, intermediate cation stabilization through hyperconjugation via methyl substitution at the carbon bearing the payload to produce “secondary coumarins” has been previously attempted, but only showed marginal improvements in quantum yield.^34^ Consequently, given the stability of allylic cations, we hypothesized that installing an allylic system onto the coumarin α-carbon (Figure 1c, **Allylic**) should significantly stabilize the incipient positive charge formed upon photoheterolysis. Since the double bond in the allylic substituent is not in conjugation with the chromophore, its influence on photochemical and photophysical properties would likely be restricted to CIP cation stability after photoheterolysis, while the wavelength of absorption and the molar attenuation coefficient should be relatively unaffected. Mono- or di-methyl substitution on the allylic system (Figure 1c, compounds **2** and **3**) was expected to confer extra stabilization through hyperconjugation and further increase cation stability. In a similar fashion, we hypothesized that direct geminal dimethylation of the α-carbon itself would stabilize the incipient CIP tertiary cation through hyperconjugation (Figure 1c, Tertiary **4**), in line with results we reported earlier this year.^19^

**Figure 2:**
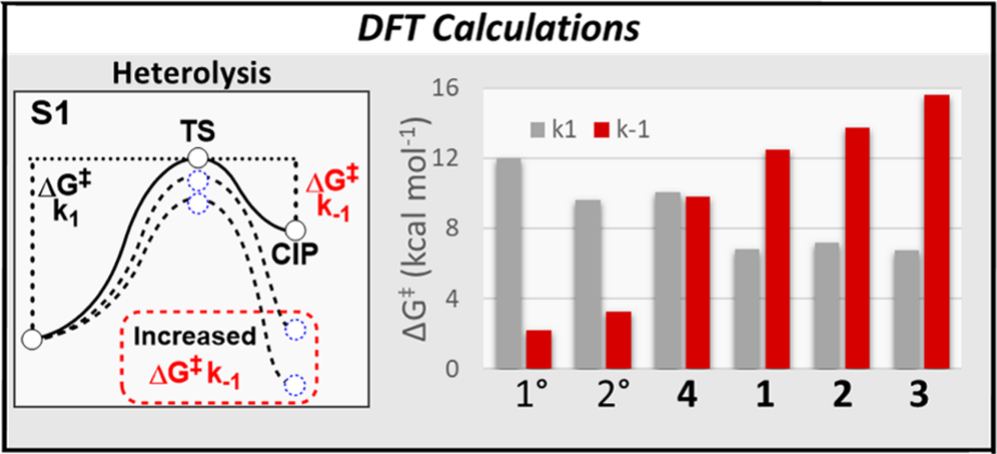
Energy barriers for the k1 and k_–1_ steps for designed coumarins **1–4** and model primary and secondary coumarins as calculated by DFT (obtained at the MN15/Def2SVP/SMD = H_2_O level of theory).

**Figure 3:**
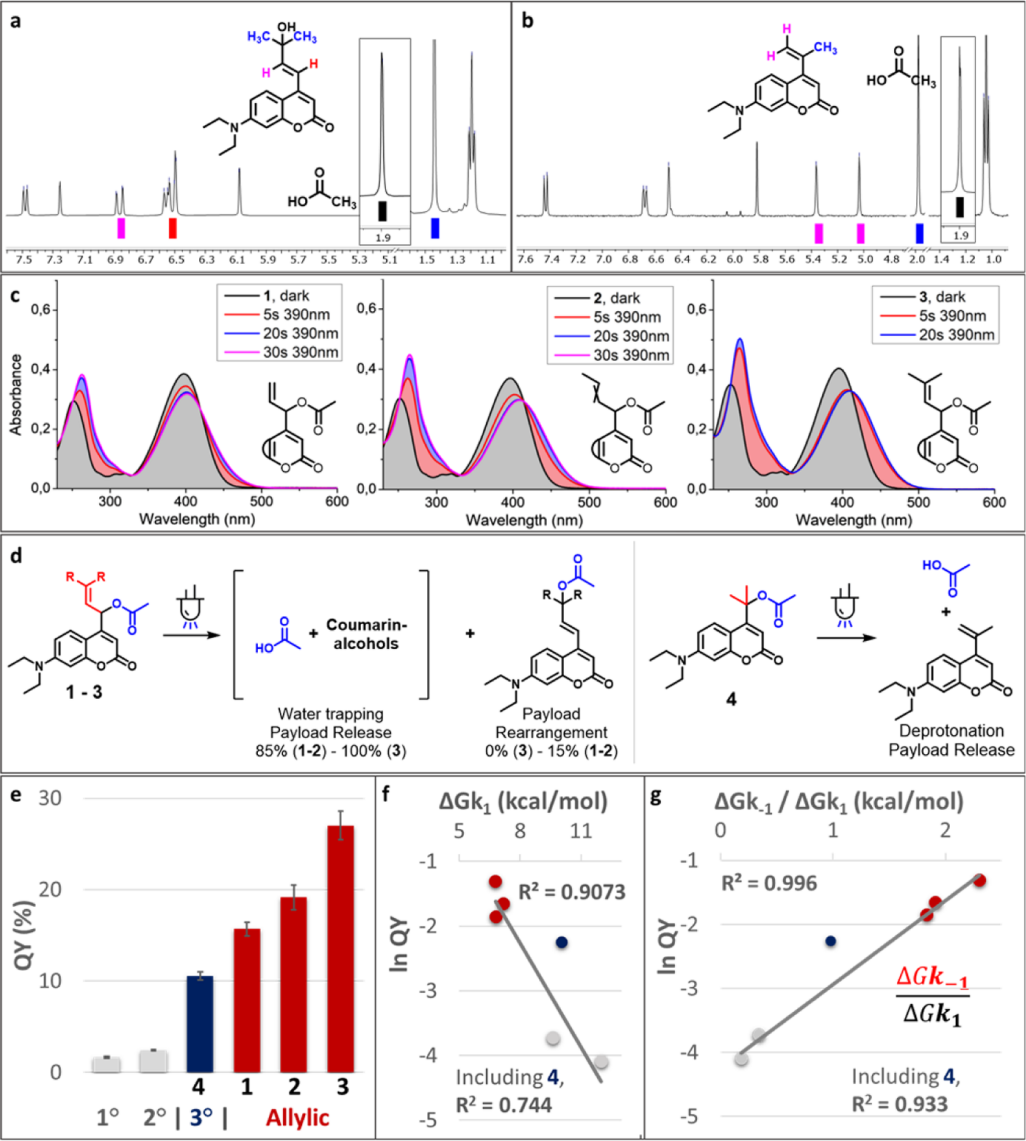
(a) Partial ^1^H-NMR spectrum of the alcohol photoproduct isolated after dilute irradiation of **3** (water/MeCN 9:1 ∼190 μM, λ = 400 nm, 5 min, spectrum in CDCl_3_) and AcOH signal obtained after irradiation of **3** in 1:1 DMSO-*d*_6_/D_2_O. For the full spectra, please see the Supporting Information, Section 7. (b) Partial ^1^H-NMR spectrum obtained after irradiation of **4** in 1:1 DMSO-*d*_6_/D_2_O (λ = 400 nm, 30 min) and AcOH signal obtained after irradiation of **3** in 1:1 DMSO-*d*_6_/D_2_O and spiking with AcOH. (c) Absorption spectra of the allylic coumarins (water/acetonitrile, 99:1), freshly prepared solutions, and solutions after irradiation (λ = 390 nm) for the times indicated. (d) Overview of the observed photoproducts after irradiation for the allylic coumarins **1–3** and tertiary coumarin **4**, relative amounts were determined by UPLC-MS peak integration. (e) Quantum yields for the uncaging of coumarin acetates in water/acetonitrile (3–5% MeCN), shown are averages and SD-values from triplicate measurements. (f) Exponential plot of photochemical QY and DFT-calculated k_1_ barrier height. (g) Exponential plot of photochemical QY and DFT-calculated k_–1_ over k_1_ barrier height. The linear curve fit indicates the exponential dependence of QY on the relative barrier height.

## Results

### DFT Calculations

Density functional theory (DFT) calculations were performed to study the effect of the nature of the allylic or tertiary cations on the calculated energy barriers of CIP formation and recombination in the first singlet excited state (S_1_, Figure 2, Δ*G*k_1_ and Δ*G*k_–1_, respectively). In all computed structures, acetic acid was chosen as the simple model payload. Geometries were optimized at the MN15/Def2SVP/SMD = H_2_O level of theory,^35−37^ and the energy barriers shown are extrapolated from the ZPE-corrected free energies obtained at the same level of theory. The employed level of theory was expected to give a good balance of computational cost to computed free energy accuracy, although it should be noted that DFT ground state or TD-DFT excited-state calculations with implicit solvent model SMD for reactions involving neutral and ion pair species may be prone to errors. Given that literature describes CIP recombination and relaxation to S_0_ as a concerted process,^30,31^ we decided to consider both S_0_ and S_1_ cations in our calculations. However, we were unable to find a stable CIP at the S_0_ energy level (vide infra, page 10) and therefore based our calculations on the S_1_ energy level. For the designed compounds **1**–**4** (Figure 1c), the barrier of CIP recombination was calculated to be significantly increased as compared to a reference primary coumarin with the same acetic acid payload (Figure 2, red bars). The extent of increase followed an expected trend depending on the degree of hyperconjugation and delocalization. Cation stabilization through allyl substitution on coumarins **1**–**3** was found to increase the energy barrier of CIP recombination more than hyperconjugation did in tertiary coumarin **4**. Although the heterolysis barrier k_1_ also differed depending on the substituents on the α-carbon (Figure 2, gray bars), less variation was observed in the energy levels of this barrier. The k_1_ barriers for allylic coumarins **1–3** were predicted to be energetically nearly identical (Figure 2, gray bars).

### Synthesis

**Scheme 2:**
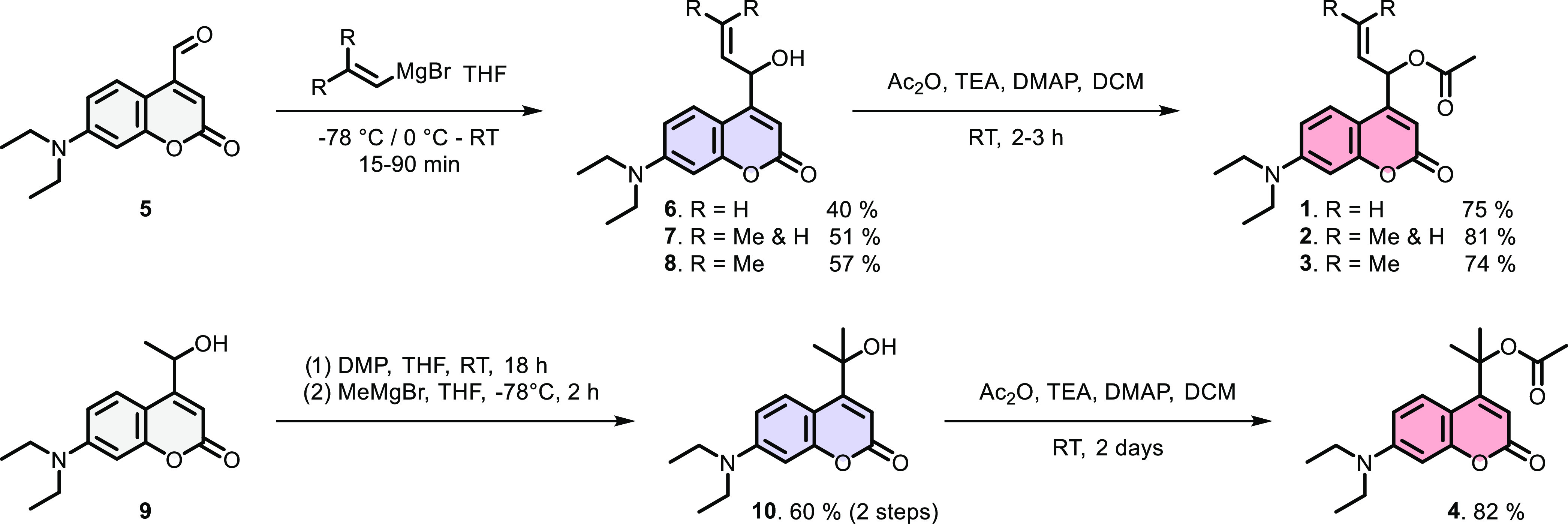
Synthetic Route for the Formation of Allylic Coumarins **1–3** and Tertiary Coumarin **4** Alcohol precursors (blue) were synthesized and acetylated to yield model compounds **1**–**4** (red).

Encouraged by the DFT calculations that predicted significantly increased energy barriers for CIP recombination in the designed coumarin-based PPGs, we set out to synthesize compounds **1**–**4** and study their photochemical properties. The newly designed photocages were all synthesized through a previously reported aldehyde intermediate **5**.^38^ Reactions of this aldehyde with a variety of allylic Grignard reagents gave the three allylic coumarin alcohols with varying methyl substitution at the northern sp^2^-carbon (Scheme 2). The model photocage—primary coumarin alcohol—is generally also obtained through one synthetic step from the same aldehyde, highlighting the synthetic accessibility of allylic coumarins **6–8**. Subsequently, the allylic coumarin alcohols were loaded with the model payload acetic acid through acetylation with acetic anhydride in the presence of DMAP as a nucleophilic catalyst.

The synthesis of tertiary coumarin **4** started from secondary coumarin alcohol **9**, which was oxidized to the ketone and treated with a methyl Grignard reagent to produce the tertiary alcohol **10** (Scheme 2). Tertiary alcohol **10** was loaded with the same model payload acetic acid through acetylation to yield coumarin **4**.

### Photochemistry

With the synthesized compounds **1**–**4** in hand, we sought to explore their photochemical properties using UV–vis spectroscopy and ^1^H-NMR analysis. Gratifyingly, irradiation (λ = 400 nm) of **1**–**4** followed by ^1^H-NMR analysis confirmed that all compounds released the payload acetic acid upon irradiation (Figure 3a,b and SI Section 7). UV–vis spectroscopy (Figure 3c) revealed that the absorption maxima of **1**–**3** were indeed comparable to that of primary coumarin: absorption spectra of **1**–**3** showed an average 4 nm red-shift as compared to that of the primary coumarin model (λ_max_ = 396 and 392 nm, respectively). As presumed, the molar attenuation coefficients of allylic coumarins **1**–**3** were similar to that of primary coumarin with an acetic acid payload (ε values = 13.700–17.500 M^–1^ cm^–1^). Irradiation (λ = 390 nm) of compounds **1**–**4** in water led to rapid (within seconds) changes in absorption spectra with clear isosbestic points (Figure 3c). Analysis of irradiated samples by ^1^H-NMR and UPLC-MS revealed that allylic coumarins **1**–**3** formed the corresponding allylic alcohols upon irradiation, the result of water trapping of the cationic intermediate (Figure 3a,d). In contrast, tertiary coumarin **4** underwent elimination toward an alkene rather than water trapping (Figure 3b,d). Furthermore, for two allylic coumarins **1**–**2**, upon irradiation, a small fraction (∼15%) of a product with a mass identical to the substrate was observed. This is believed to be the result of acetate payload rearrangement to the allylic carbon (Figure 3d and SI Section 9). Irradiation of allylic coumarin **3** did not result in payload rearrangement, and this compound displayed clean conversion to the alcohol as a result of water trapping (Figure 3d).

The quantum yields of PPG consumption were determined by UV–vis spectroscopy in water with a small amount of acetonitrile. As reference model compounds, widely used primary and secondary coumarin PPGs with the same acetic acid payload were also studied. To our delight, cation stabilization resulted in a remarkably high QY for all newly designed coumarins, with the allylic coumarin **3** showing a QY as high as 27%, a 16-fold increase over its primary coumarin model (Figure 3e). Notably, uncaging cross sections of up to ∼3.7 × 10^3^ were achieved with the allylic coumarins **1**–**3** (Table 1).

**Table 1:** Photochemical Properties of Coumarin PPGs **1**–**4** and Model 1 and 2° Coumarins^a^

PPG	CPD no.	λ_max_ (nm)	ε (L mol^–1^ cm^–1^)/10^3^	QY	ε × QY/10^3^	Δ*G*^‡^k_–1_ (kcal mol^–1^)
1°		393	16.4	0.017	0.27	2.23
2°		389	18.2	0.024	0.44	3.27
3°	**4**	394	15.9	0.105	1.7	9.82
	**1**	398	17.5	0.157	2.7	12.51
allylic	**2**	397	16.6	0.192	3.2	13.74
	**3**	396	13.8	0.270	3.7	15.60

a Included are λ_max_ values, molar absorptivity (ε) at λ = 400 nm, quantum yield (QY), uncaging cross section at λ = 400 nm (ε × QY), and the energy barrier for CIP recombination, as calculated by DFT (Δ*G*^‡^k_–1_).

To rationalize these results, the observed heterolysis QYs were compared to the heterolysis energy barriers of CIP formation and recombination, as calculated by DFT. Gratifyingly, we found that the barrier height of the unproductive k_–1_ process (the CIP recombination) was a good predictor for the quantum yield (SI, Figure S93). In contrast, we observed that the calculated energy barrier for the k_1_ process correlated poorly to photochemical QY (Figure 3f). These findings confirm our initial hypothesis that the limiting factor in coumarin photochemical QY is CIP recombination, rather than CIP formation itself. Also, the relative barrier height ratio of k_–1_ over k_1_ showed a strong correlation with photochemical QY, exhibiting an exponential relationship between this relative barrier height ratio and the QY (Figure 3g). These results demonstrate that retarding CIP recombination results in drastic enhancements in photochemical QY, and, moreover, confirm this as a viable strategy for improving the PPG efficiency. A possible explanation for the poor correlation of k_1_ barrier height to the QY could be that this barrier is partially overcome through excitation to a higher vibrational level in S_1_, regardless of the nature of the incipient CIP cation. This hypothesis is supported by the large Stokes shift of 7-diethylamino-coumarins (Supporting Information, Figure S49), suggesting that a higher S_1_ vibrational level is almost always accessed upon excitation. Although the barrier height of the seemingly important productive forward step k_2_ is not included in this calculation, our calculations nonetheless provide an exponential trend correlating the reversibility of CIP formation to QY. This suggests that the k_2_ barrier height is energetically comparable for all shown coumarin photocages, also confirming our initial hypothesis that this barrier is largely independent of CIP stability. This is plausible, since both the acetate payload and the photoproduct cations in the CIPs formed from the photoheterolysis of each PPG variant **1**–**4** are similar in their electrostatic topology and thus must be similar in their Coulombic nature. Thus, the diffusion rate out of the CIP, ultimately resulting in productive cation trapping by the solvent, would be expected to be near-identical for each PPG variant **1**–**4**.

The photochemical quantum yield of tertiary coumarin **4** (Figure 3g, dark blue) did not fit the predicted trend. This discrepancy can be explained by the fact that tertiary coumarin **4** exhibits a different mechanism of CIP escape. Whereas the usual mechanism of CIP escape is diffusion followed by solvent trapping, tertiary coumarin exhibited elimination toward an alkene (Figure 3b). Since the photochemical quantum yield of tertiary coumarin is higher than expected (i.e., it can be found above the curve fit), it can be concluded that its deprotonation barrier must be lower than the k_2_ barrier for the Allylic coumarins **1**–**3** and primary and secondary coumarins. Currently, we are performing further studies on the effect of the k_2_ barrier on PPG photoheterolysis efficiency.

In literature describing the coumarin PPG mechanism of photocleavage, often CIP recombination and relaxation to S_0_ is reported as a concerted process (i.e., a conical intersection of the S_1_ and S_0_ potential energy surfaces exists at the CIP recombination event).^30,31^ However, one must also consider the possibility that relaxation back to S_0_ happens before CIP recombination and, thus, that CIP recombination happens on the S_0_ potential energy surface. We could not find definitive evidence for this situation, but nonetheless, we attempted to calculate the energy barriers for CIP recombination on the S_0_ potential energy surface as well. Our DFT calculations were unable to find a stable CIP in the S_0_ state or even a heterolysis transition state for all described coumarins **1**–**4**. Although we can’t fully exclude some extent of recombination occurring in the ground state, the good correlation of the measured QYs with the S_1_ energy barriers suggests that CIP recombination in S_1_ seems the most reasonable. In a recent paper by Contreras-García et al., no local minimum was found either for the ion pair—and thus no energy barrier for CIP recombination—in the ground state.^39^

### Allylic Substitution as a General Strategy for Quantum Yield Enhancement

**Figure 4:**
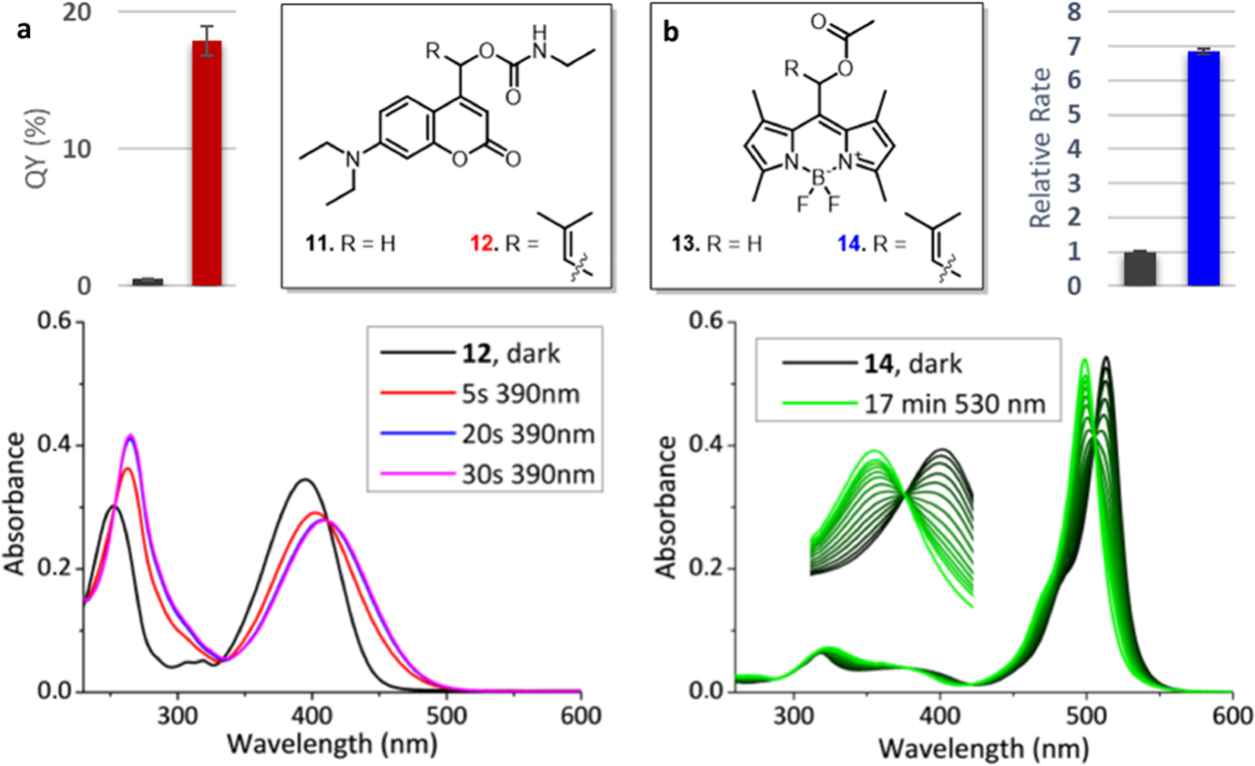
(a) Quantum yields (55 μM, 9:1 water/MeCN) of coumarin-carbamates **11** (black bar) and **12** (red bar). Irradiation-dependent absorption spectra (20 μM, water/MeCN 99:1) of coumarin-carbamate **12**. (b) Relative uncaging rate of BODIPY **13** (black bar) and **14** (blue bar) (10 μM, water/MeCN 9:1). Irradiation-dependent absorption spectra (10 μM, water/MeCN 9:1) of BODIPY **14**.

Since the photochemical QY improvement of coumarin photocages was achieved through the stabilization of the cationic chromophore component of the CIP, we predicted that it would be a general strategy for QY improvement that would also hold for payloads other than carboxylic acids. To explore this generality, the allylic PPG with the highest QY (compound **3**) was also used to cage and release another moiety that is often photocaged in bioactive payloads: an amine group. Due to their inability to stabilize a negative charge, amines are generally photocaged as their carbamate analogues.^40^ Two photocages bearing an amine payload were synthesized, one bearing the allylic substituent on the *α*-carbon (Figure 4a, **12**, red) and a primary reference compound (Figure 4a, **11**, black). Upon comparing their photochemical quantum yields, we were pleased to find that the allylic carbamate also displayed very efficient photolysis. Its quantum yield underwent an >35-fold improvement over that of the model primary coumarin bearing the same carbamate payload, an even more pronounced effect than the one that was observed for acetate payloads. These results highlight the robustness of this approach: PPG cation stabilization is a general way to slow down CIP recombination, which is expected to improve photochemical QY for a diverse subset of payloads.

Finally, we aimed to see if this optimization strategy holds also for a different member of the class of heterolytic PPGs. Arguably, all PPGs belonging to this class would benefit from a stabilized incipient chromophore cation through allylic substitution at the α-carbon. To illustrate this, we set out to introduce allylic substitution on another widely used class of photocages, BODIPY PPGs. Allylic-BODIPY PPG **14** (Figure 4b, blue) was synthesized and confirmed to release acetic acid upon irradiation (SI Section 7). Interestingly, rather than photobleaching that is often observed for BODIPY photocages, Allylic-BODIPY PPG **14** showed clean conversion to a different BODIPY photoproduct with a clear isosbestic point (Figure 4b). The photoproduct is likely the result of water trapping of the cation (^1^H-NMR, SI Figure S60). Finally, the rate of deprotection of Allylic-BODIPY PPG **14** was compared to that of primary BODIPY **13** at the same concentration. A 7-fold improvement in rate was found. Since the molar attenuation coefficients at the irradiation wavelength (λ = 530 nm) for both compounds are virtually the same (see the Supporting Information for details), the samples exhibited very similar absorption, allowing us to translate the rate improvement directly to the 7-fold improvement in quantum yield. These findings illustrate the versatility of this simple principle: our strategy of cation stabilization results in significant improvements in photochemical QY and holds for multiple types of PPGs.

### Fluorescence Properties of the Coumarin Uncaging Product

**Figure 5:**
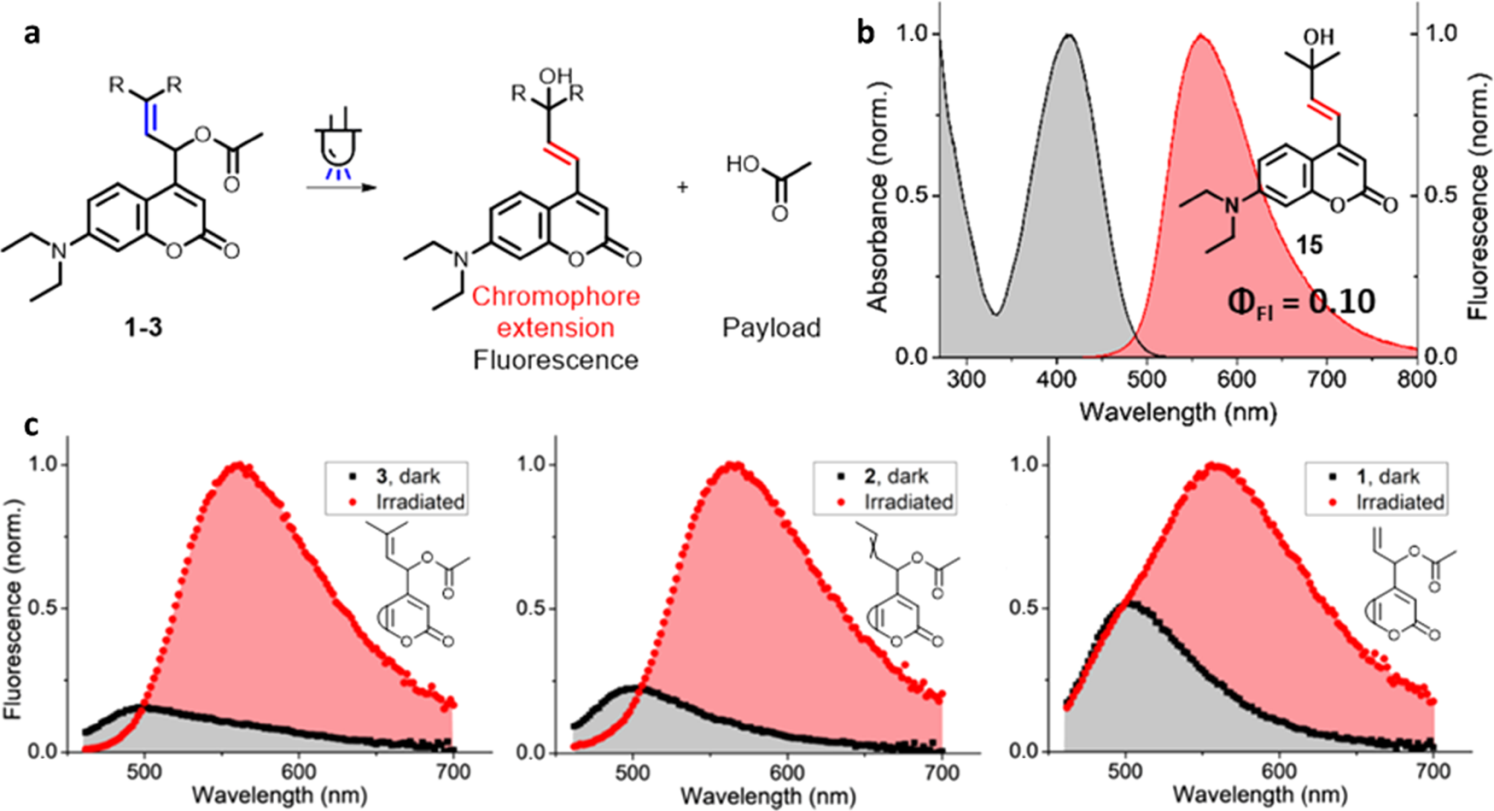
(a) Structure of the fluorescent coumarin uncaging product formed after irradiation of allylic coumarins **1**–**3**. (b) Normalized fluorescence spectrum of the photoproduct of allylic coumarin **3** (20 μM, Water/MeCN 99:1). (c) Normalized fluorescence spectra of allylic coumarins **1–3** (20 μM, Water/MeCN 99:1, λ_exc_: 434 nm). Fluorescence spectra of the same samples were recorded before (black curves) and after irradiation (λ_max_ = 400 nm, 90 s, red curves).

In the photoheterolysis process in aqueous media, the allylic coumarin PPGs **1**–**3** undergo water trapping to form alcohols. Large-scale irradiation of allylic coumarin **3** with dimethyl substitution allowed for the detailed characterization of its photoproduct. The isolated alcohol **15** (Figure 5b) was presumably the result of an irradiation-dependent S_N_1′ type of reaction, in which the double bond had rearranged (Figure 5a) and water trapping occurred at the northern tertiary carbon atom, likely due to the higher stability of the cation at this position. Due to the extended conjugation of the chromophore established after the double bond had rearranged, the absorption spectrum of this photoproduct showed a bathochromic shift as compared to the starting material (Figure 3c). Fluorescence characterization of this compound also revealed a pronounced bathochromic shift of its fluorescence emission spectrum as compared to the photocaged substrate, with the photoproduct showing an intense emission band between 550 and 650 nm (Figure 5b) and a fluorescence quantum yield of ∼10% (See the SI, Section 6). Because of the bathochromic shift of both the absorption- and emission spectrum of the photoproduct, during the deprotection process, the fluorescence signal of the photoproduct could be addressed almost exclusively through excitation and emission readout at red-shifted wavelengths (λ = 434 and 565 nm, respectively).

In a similar way, allylic coumarin **2** also showed conversion to the rearranged alcohol upon irradiation, enabling a similar fluorescence readout of the uncaging process (Figure 5c). In contrast, upon irradiation, allylic coumarin **1** produced a mixture of both the rearranged alcohol and the product of direct water substitution at the α-carbon, slightly affecting its fluorescence response (Figure 5c). However, since the rearranged alcohol had distinct fluorescence properties, a similar fluorescent sensitivity for the uncaging process could be achieved at red-shifted wavelengths. It should be strongly emphasized that the significant enhancement in fluorescent properties for allylic coumarins **1–3** upon irradiation can be used to follow the payload release through normalization of the fluorescence signal.^41−43^

To illustrate the utility of the allylic PPGs **1**–**3** and their fluorescence properties, we aimed to use the PPGs to photocage a model bioactive compound and release this compound upon irradiation in a process whose progress could be independently followed solely by fluorescence. Due to our lab’s experience in the photoactivation of antibiotics,^44−47^ we have chosen piperacillin—a potent member of the β-lactam class—as a model bioactive compound.

Since the caged antibiotics were to be used in a 24-h bacterial growth assay, the hydrolytic stability of the model photocaged compounds **1**–**3** was first evaluated. Allylic photocages **1** and **2** showed excellent hydrolytic stability, forming no hydrolysis product after 15 h at 25 °C in the dark. Allylic photocage **3**, however, showed slight hydrolysis after 15 h, likely by virtue of its superior cation stability after hydrolysis (SI, Figure S89). Given its greater hydrolytic stability, it was decided to use allylic photocage **1** for the construction of the caged antibiotics whose activation can be followed by fluorescence.

**Figure 6:**
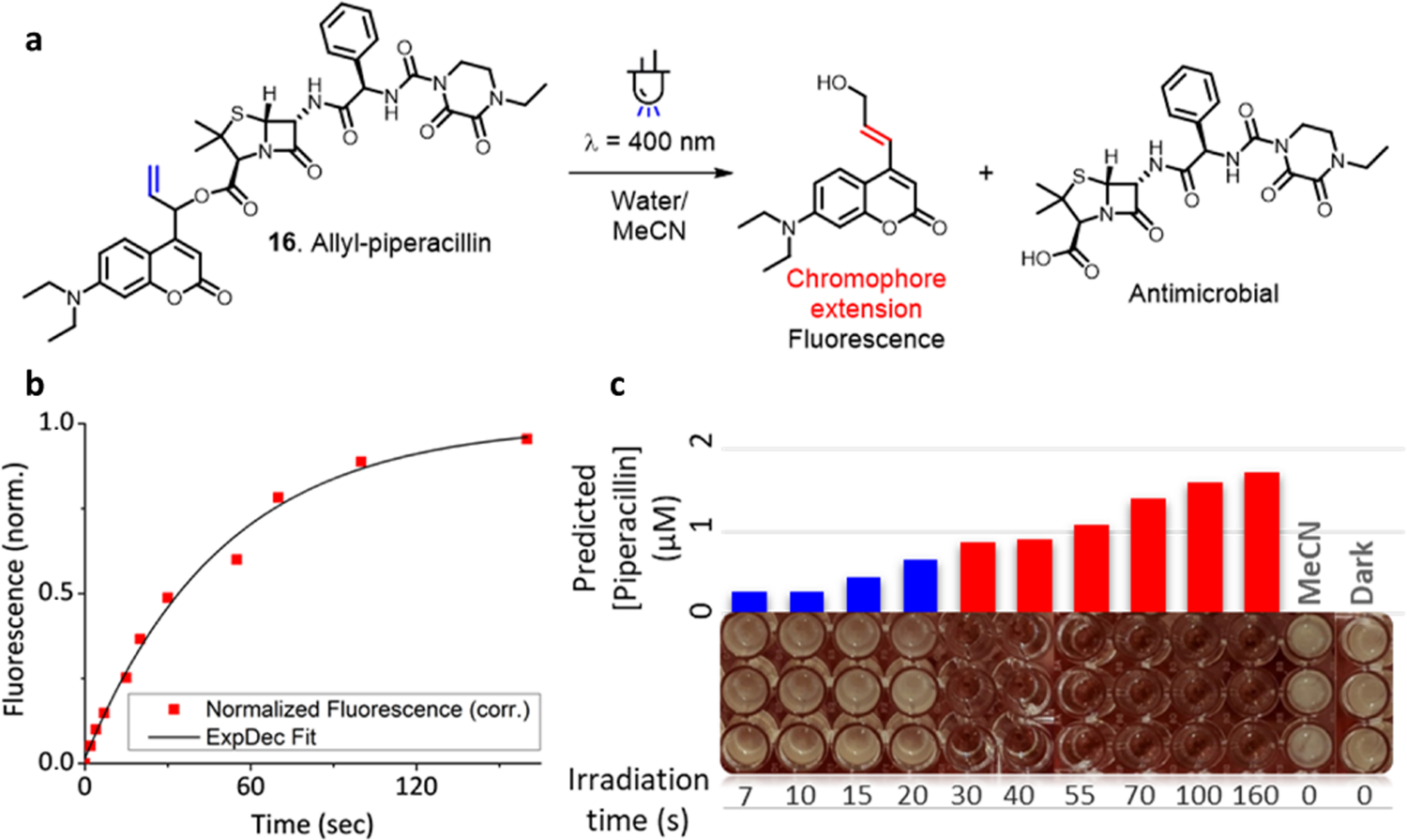
(a) Irradiation of allyl-piperacillin **16** resulting in the formation of the antimicrobial and a fluorescent reporter of payload release. (b) Fluorescence response of allyl-piperacillin **16** upon irradiation (20 μM, water/acetonitrile 8:2, irradiation at λ_max_ = 400 nm, fluorescence excitation at λ = 434 nm, readout at λ = 565 nm). (c) Results of the antimicrobial assay. The predicted piperacillin concentrations calculated from the fluorescence response are reported, as well as the irradiation time (in seconds). Blue bars show bacterial growth, indicating piperacillin concentrations below the MIC. Red bars show full growth inhibition, indicating piperacillin concentrations above the MIC.

In an initial assay, the minimal inhibitory concentration (MIC) of piperacillin toward*Escherichia coli*CS1562 was determined to be 0.5–1.0 μM (SI, Figure S84). The required photocaged piperacillin **16** (Figure 6a) was conveniently obtained through a Steglich esterification of piperacillin with the corresponding allylic alcohol **6**.

Irradiation of photocage **16** showed the expected emergence of a fluorescent response due to double bond rearrangement (Figure 6b). The predicted degree of payload release was calculated from the fluorescence signal through an exponential decay curve fit and was corrected for the observed amount of payload rearrangement (10%, UPLC-MS, SI). To our delight, the predicted concentrations of the liberated piperacillin (obtained after irradiation with λ = 400 nm light for much less than a minute) corresponded perfectly to bacterial growth inhibition (Figure 6c). A predicted piperacillin concentration of 0.88 μM showed bacterial growth inhibition, whereas 0.66 μM still allowed for bacterial growth. This indicates that the fluorescence-determined MIC was 0.66–0.88 μM, well within our independently determined MIC of 0.5–1.0 μM.

These results confirm the practical utility of the allylic photocages reported herein and illustrate the reliability of the fluorescent signal that can be used to quantitatively predict the amount of bioactive payload released in biological assays. In sum, our newly developed PPGs **1–3** developed using the QY enhancement strategy described herein allowed for the rapid release of complex biological payloads while enabling a convenient fluorescence readout method for monitoring the uncaging process.

## Discussion

In this study, we identify an approach for preventing CIP recombination in PPG photolysis as a key strategy to greatly increase the photolysis QY. The QY enhancement strategy is supported by DFT calculations, which predict higher energy barriers for the recombination process. An excellent correlation between the reversibility k_–1_ of the heterolytic step and the photochemical quantum yield was observed. We also concluded that a poor correlation exists between the calculated k_1_ barrier and our measured QYs and have thus demonstrated that preventing recombination of the CIP, rather than kinetically stimulating its formation, is a crucial strategy in increasing PPG efficiency. Furthermore, we showed that our strategy for increasing PPG efficiency through incipient cation stabilization is general for different payloads (carboxylic acids and amines) and for different PPG classes (coumarin and BODIPY PPGs), again demonstrating the versatility of this simple principle.

Given their synthetic accessibility and high photochemical QYs, the efficient PPGs **1–3** themselves, designed applying the cation stabilization strategy showcased herein, could prove valuable in assays that require near-instant release of a payload, such as those in time-resolved structural biology studies.^48^ Furthermore, the fluorescent readout of PPGs **1–3** can be reliably used to quantify the amount of complex biological payload release in complex biological environments, as is also demonstrated.

We believe the new PPG design strategy demonstrated herein directly addresses the need for increasing PPG efficiency in the rapidly expanding research fields that rely on precise activation of molecular function with light. Our QY engineering strategy will undoubtedly prove valuable for the development of ideal photocages tailored for more complex applications, for example (but not limited to), red-shifted photocages with high quantum yields for use in photopharmacology.

## Supporting Information Available

Synthetic methods, NMR-spectra, photochemical data, bacterial growth curves, computational data, and ambient light stability (PDF)
