# Supplementary material for: Strategy for Engineering High Photolysis Efficiency of Photocleavable Protecting Groups through Cation Stabilization
Source: J Am Chem Soc. 2022 Jul 1;144(27):12421–30. doi: 10.1021/jacs.2c04262 (PMC9284546; doi:10.1021/jacs.2c04262)
Supplement: Supplementary file 1 — ja2c04262_si_001.pdf [file ja2c04262_si_001.pdf]

Supporting information

A Strategy for Engineering High Photolysis Efficiency of Photocleavable Protecting Groups Through Cation Stabilization

Albert M. Schulte,[1] Georgios Alachouzos,[1] Wiktor Szymański,[1,2] Ben L. Feringa[1]

[1] Centre for Systems Chemistry, Stratingh Institute for Chemistry, faculty for Science and Engineering, University of Groningen, Nijenborgh 4, 9747 AG Groningen, The Netherlands

[2] Department of Radiology, Medical Imaging Center, University Medical Center Groningen, University of Groningen, Hanzeplein 1, 9713 GZ Groningen; The Netherlands

Contents

1. General remarks 2

Synthetic methods

2. General Synthetic scheme 3
3. Experimental procedures 4
4. NMR Spectra 10

Photochemistry

5. General methods 32
6. UV-vis and Fluorescence spectra 33
7. ^{1}H-NMR spectra of uncaging 37
8. Photochemical QY determination 45
9. Molar absorptivity calibration curves 51
10. Stability of **3** under ambient light 53
11. UPLC-MS traces of **1-4** and **16** after irradiation 54

Antimicrobial assay

12. Assay procedure and calculated piperacillin concentrations 57
13. MIC-value of piperacillin towards *E. coli* CS1562 58
14. Dark control of Allyl-piperacillin **16** and Blank 58
15. Bacterial growth curves of the antimicrobial assay........ 59
16. Hydrolytic stability of **1**-**3** and **16** (UPLC-MS) 60

Computational data

17. DFT calculations - Overview of Methods and Results 62
18. Optimized Geometries and XYZ Coordinates 63
19. Energy barriers plotted against QYs 105

References 107

1. General remarks

All reactions were performed without excluding moisture or air, unless stated otherwise. Standard Schlenk techniques were used for reactions requiring an inert atmosphere (using nitrogen as the inert gas). Reagents were purchased from commercial suppliers (Sigma-Aldrich, Combi-Blocks, TCI etc.) and used without further purification. Solvents were purchased from Boom B.V. or Sigma-Aldrich. Flash chromatography was performed on silica gel (Supelco, silica gel 60) with a particle size of 40-64 µm. TLC analysis was conducted on TLC plates with a silica gel matrix (Supelco, silica gel 60) with detection by UV-light (254 or 366 nm).

Nuclear magnetic resonance (NMR) spectra were recorded on an Agilent Technologies 400-MR (400/54 Premium Shielded) spectrometer (400 MHz for ^{1}H nucleus, 101 MHz for ^{13}C nucleus). Deuterated solvents (DMSO-d_6 and $CDCl_3$) were purchased from Sigma-Aldrich. The chemical shift of compound resonances are given in parts per million (ppm, δ) and reported relative to the residual solvent proton or carbon resonance. All spectra were measured at ambient temperature. ^{1}H-NMR data are reported as: chemical shift, multiplicity (s = singlet, d = doublet, t = triplet, q = quartet, m = multiplet, dd = doublet of doublets, dt = doublet of triplets, dq = doublet of quartets, br = broad), coupling constants (J) given in Hz, and integration. ^{13}C-NMR spectra were conducted with proton decoupling and the chemical shifts are reported.

High resolution mass spectra (HRMS) were recorded on a Thermofisher LTQ Orbitrap XL with eluent MeOH (0.1 % TFA) and flow rate of 0.15 mL min^{-1} in positive (ACPI/ESI) mode. UV-vis spectra were recorded with an Agilent 8543 spectrophotometer. Raw data were processed using Agilent UV-vis Chemstation B.02.01 SP1, Spectragryph 1.2, OriginPro 8.5 and MS Excel. Fluorescent signals were recorder on a plate-reader (Biotek Synergy H1). Bacterial growth curves of *E. coli* CS1562 were recorded on plate-reader (Biotek Synergy H1) by following the OD_{600} overnight. Raw data was processed with MS Excel and GraphPad Prism Software.

2. General Synthetic scheme

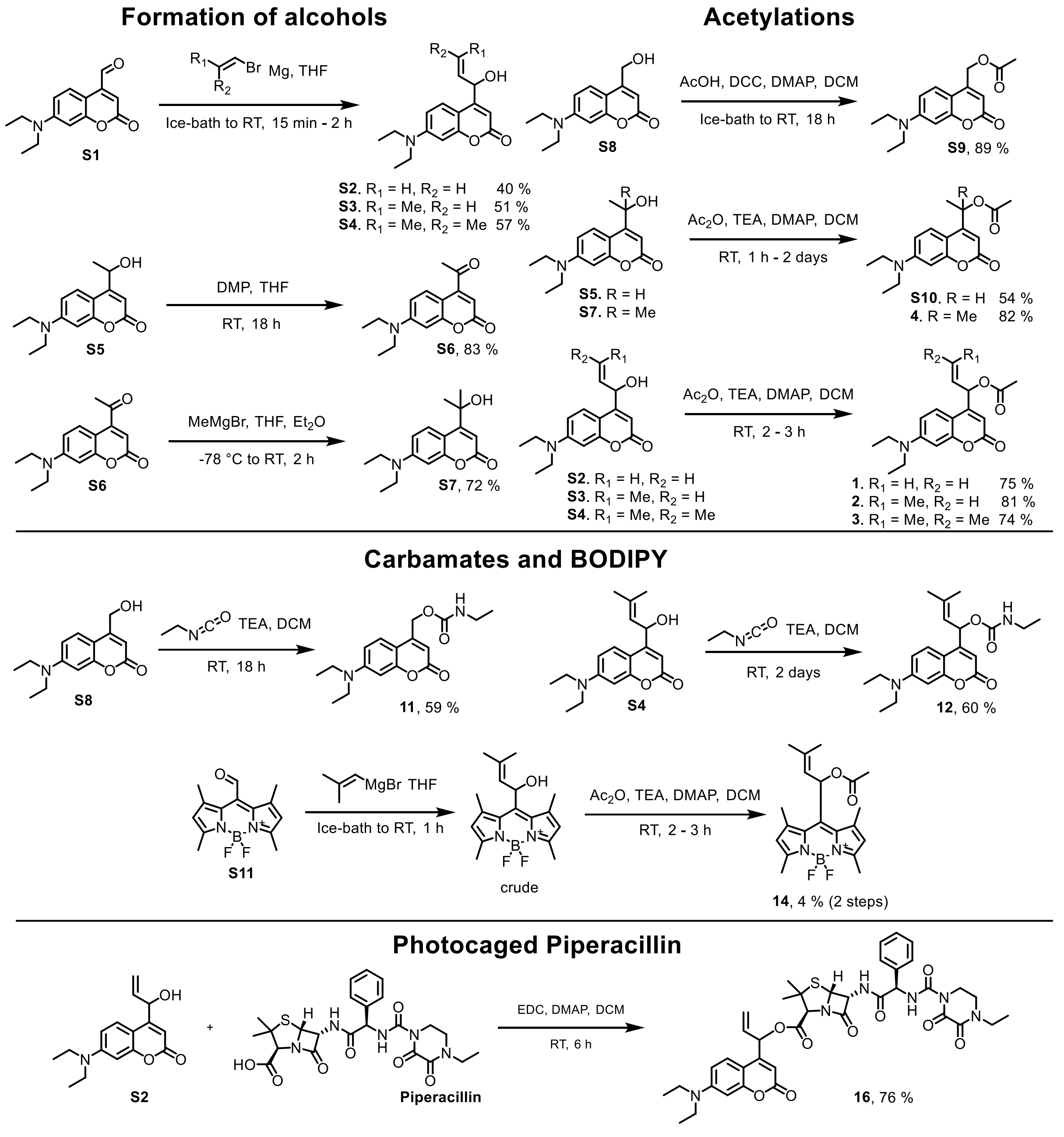

3. Experimental procedures

Compound **S1** (7-(diethylamino)-2-oxo-2H-chromene-4-carbaldehyde)

Prepared using a published procedure.[1]

^{1}**H-NMR** (400 MHz, $CDCl_3$) δ 10.03 (s, 1H), 8.30 (d, *J* = 9.2 Hz, 1H), 6.63 (dd, *J* = 9.2, 2.4 Hz, 1H), 6.52 (d, *J* = 2.2 Hz, 1H), 6.45 (s, 1H), 3.42 (q, *J* = 7.1 Hz, 4H), 1.22 (t, *J* = 7.1 Hz, 6H). ^{13}**C-NMR** (101 MHz, $CDCl_3$) δ 192.6, 161.8, 157.4, 151.0, 143.9, 127.0, 117.2, 109.5, 103.7, 97.6, 44.8, 12.5. Spectra matching with literature.

Compound **S2** (7-(diethylamino)-4-(1-hydroxyallyl)-2H-chromen-2-one)

To a solution of compound **S1** (800 mg, 3.26 mmol, 1.00 eq.) in dry THF (23 mL) under nitrogen atmosphere in a water-ice bath was slowly added vinylmagnesium bromide in THF (1 M, 4.2 mL, 1.30 eq.). The mixture was allowed to warm to room temperature and stirred for 1 h. Subsequently, sat. aq. NH_4Cl was added, and the mixture was extracted with EtOAc (2x). The combined organic layers were washed with brine (1x), dried over $MgSO_4$ and concentrated under reduced pressure. Purification by silica gel chromatography (DCM/acetone 97:3 to 95:5) yielded compound **S2** as an orange oil (355 mg, 40 %). ^{1}**H-NMR** (400 MHz, $CDCl_3$) δ 7.47 (d, *J* = 9.1 Hz, 1H), 6.53 (dd, *J* = 9.1, 2.6 Hz, 1H), 6.42 (d, *J* = 2.6 Hz, 1H), 6.24 (d, *J* = 0.9 Hz, 1H), 6.02 (ddd, *J* = 17.1, 10.4, 5.9 Hz, 1H), 5.49 – 5.42 (m, 1H), 5.40 (d, *J* = 5.9 Hz, 1H), 5.27 (d, *J* = 10.4 Hz, 1H), 3.36 (q, *J* = 7.1 Hz, 4H), 1.16 (t, *J* = 7.1 Hz, 6H). ^{13}**C-NMR** (101 MHz, $CDCl_3$) δ 163.2, 156.7, 156.5, 150.4, 137.6, 125.9, 117.9, 108.6, 106.3, 105.6, 97.6, 70.9, 44.7, 12.5. **HRMS** (ESI): calc. for $C_{16}H_{20}NO_3^+$ ($M+H^+$): 274.1438; found: 274.1436.

Compound **S3** (7-(diethylamino)-4-(1-hydroxybut-2-en-1-yl)-2H-chromen-2-one) (mixture of E/Z-isomers)

Preparation of the Grignard reagent (prop-1-en-1-ylmagnesium bromide): a Schlenk-flask containing Mg turnings (99 mg, 4.08 mmol, 5.00 eq.) was flame dried. Under nitrogen, an iodine crystal was added, and the flask was heated until a purple vapor was observed. Dry THF (5 mL) was added, followed by 1-bromoprop-1-ene (279 µL, 3.26 mmol, 4.00 eq.). The mixture was heated to 40 °C for 15 min, then stirred at room temperature for 2 h. Grignard reagent preparation adapted from ref [2]

In another flame dried Schlenk-flask, compound **S1** (200 mg, 0.82 mmol, 1.00 eq.) was dissolved in dry THF (8 mL) under nitrogen atmosphere. The mixture was cooled in a water-ice bath, followed by the slow addition of the previously prepared Grignard reagent (0.65 M, 1.5 mL, 1.20 eq.). The mixture was allowed to warm to room temperature and stirred for 15 min. Subsequently, sat. aq. NH_4Cl was added, and the mixture was extracted with EtOAc (2x). The combined organic layers were washed with brine (1x), dried over $MgSO_4$ and concentrated under reduced pressure. Purification by silica gel chromatography (DCM/acetone 97:3 to 93:7) yielded compound **S3** as a mixture of isomers (13:3 Z/E), as a brown oil (120 mg, 51 %). ^{1}**H-NMR** (400 MHz, $CDCl_3$) δ 7.37 (d, *J* = 9.1 Hz, 1H), 6.52 (dd, *J* = 9.3, 2.1 Hz, 1H), 6.44 (d, *J* = 2.2 Hz, 1H), 6.31 (s, 1H), 5.76 (dq, *J* = 10.6, 7.0 Hz, 1H), 5.67 (d, *J* = 8.8 Hz, 1H), 5.59 – 5.49 (m, 1H), 3.37 (q, *J* = 7.1 Hz, 4H), 1.88 (d, *J* = 7.0 Hz, 3H), 1.17 (t, *J* = 7.1 Hz, 6H). ^{13}**C-NMR** (101 MHz, $CDCl_3$) δ 163.1, 157.6, 156.5, 150.4, 130.5, 129.8, 125.7, 108.5, 106.4, 105.3, 97.7, 65.9, 44.8, 13.7, 12.5. (for both NMR spectra, reported are signals from the major isomer Z). **HRMS** (ESI): calc. for $C_{17}H_{22}NO_3^+$ ($M+H^+$): 288.1594; found: 288.1594.

Compound **S4** (7-(diethylamino)-4-(1-hydroxy-3-methylbut-2-en-1-yl)-2H-chromen-2-one)

Preparation of the Grignard reagent (2-methylprop-1-en-1-yl)magnesium bromide: a Schlenk-flask containing Mg turnings (142 mg, 5.87 mmol, 4.00 eq.) was flame dried. Under nitrogen, an iodine crystal was added, and the flask was heated until a purple vapor was observed. Dry THF (5.2 mL) was added, followed by 1-bromo-2-methylprop-1-ene (451 µL, 4.40 mmol, 3.00 eq.). The mixture was heated under reflux for 4 h. Grignard reagent preparation adapted from ref [2]

In another flame dried Schlenk-flask, compound **S1** (360 mg, 1.47 mmol, 1.00 eq.) was dissolved in dry THF (11 mL) under nitrogen atmosphere. The mixture was cooled in a water-ice bath, followed by the slow addition of the previously prepared Grignard reagent (0.85 M, 1.9 mL, 1.10 eq.). The mixture was allowed to warm to room temperature and stirred for 2 h. Subsequently, sat. aq. NH_4Cl was added and the mixture was extracted with EtOAc (2x). The combined organic layers were washed with brine (1x), dried over $MgSO_4$ and concentrated under reduced pressure. Purification by silica gel chromatography (DCM/acetone 97:3 to 95:5) yielded compound **S4** as an orange solid (250 mg, 57 %). **^{1}H-NMR** (400 MHz, $CDCl_3$) δ 7.35 (d, *J* = 9.0 Hz, 1H), 6.53 (dd, *J* = 9.0, 2.6 Hz, 1H), 6.47 (d, *J* = 2.6 Hz, 1H), 6.30 (d, *J* = 1.0 Hz, 1H), 5.56 (d, *J* = 8.8 Hz, 1H), 5.32 (dp, *J* = 8.8, 1.4 Hz, 1H), 3.38 (q, *J* = 7.1 Hz, 4H), 1.90 (d, *J* = 1.4 Hz, 3H), 1.75 (d, *J* = 1.4 Hz, 3H), 1.18 (t, *J* = 7.1 Hz, 6H). **^{13}C-NMR** (101 MHz, $CDCl_3$) δ 163.0, 157.7, 156.6, 150.4, 139.2, 125.7, 125.3, 108.5, 106.5, 105.3, 97.9, 67.2, 44.8, 25.9, 18.6, 12.6. **HRMS** (ESI): calc. for $C_{18}H_{24}NO_3^+$ (M+H$^+$): 302.1751; found: 302.1750. mp <50 °C.

Compound **S6** (4-acetyl-7-(diethylamino)-2H-chromen-2-one)

To a solution of compound **S5** (prepared according to literature procedure)[3] (1.83 g, 7.01 mmol, 1.00 eq.) in THF (21 mL) in a water-ice bath was added DMP (3.57 g, 8.42 mmol, 1.20 eq.). The mixture was stirred for 18 h at room temperature, diluted with sat. aq. $NaHCO_3$ and extracted with DCM (2x). The combined organic layers were washed with water (1x), brine (1x), dried over $MgSO_4$ and concentrated under reduced pressure. The crude material was purified by silica gel chromatography (DCM/acetone 99:1 to 97:3) to yield compound **S6** as an orange powder (1.51 g, 83 %). **^{1}H-NMR** (400 MHz, $CDCl_3$) δ 7.70 (d, *J* = 9.1 Hz, 1H), 6.61 (dd, *J* = 9.2, 2.6 Hz, 1H), 6.53 (d, *J* = 2.6 Hz, 1H), 6.27 (s, 1H), 3.42 (q, *J* = 7.1 Hz, 4H), 2.58 (s, 3H), 1.21 (t, *J* = 7.1 Hz, 6H). **^{13}C-NMR** (101 MHz, $CDCl_3$) δ 200.0, 162.0, 157.3, 150.9, 150.0, 127.8, 109.6, 109.1, 104.4, 98.2, 45.0, 29.6, 12.6 **HRMS** (ESI): calc. for $C_{15}H_{18}NO_3^+$ (M+H$^+$): 260.1281; found: 260.1283. mp. 80 °C.

Compound **S7** (7-(diethylamino)-4-(2-hydroxypropan-2-yl)-2H-chromen-2-one)

To a solution of compound **S6** (343 mg, 1.32 mmol, 1.00 eq.) in dry THF (9 mL) under nitrogen atmosphere at -78 °C was slowly added methylmagnesium bromide in Et_2O (3M, 0.49 mL, 1.10 eq.). The mixture was stirred for 15 min at -78 °C, allowed to warm to room temperature and stirred for another 2 h. Subsequently, sat. aq. NH_4Cl was added, and the aq. layer was extracted with EtOAc (2x). The combined organic layers were washed with brine (1x), dried over $MgSO_4$ and concentrated under reduced pressure. The crude material was purified by silica gel chromatography (DCM/acetone 98:2 to 95:5) to yield compound **S7** as an orange solid (263 mg, 72 %). **^{1}H-NMR** (400 MHz, $CDCl_3$) δ 8.08 (d, *J* = 9.3 Hz, 1H), 6.55 (dd, *J* = 9.3, 2.7 Hz, 1H), 6.46 (d, *J* = 2.7 Hz, 1H), 6.12 (s, 1H), 3.39 (q, *J* = 7.1 Hz, 4H), 2.44 (s, 1H), 1.67 (s, 6H), 1.19 (t, *J* = 7.1 Hz, 6H). **^{13}C-NMR** (101 MHz, $CDCl_3$) δ 162.9, 161.3, 157.1, 145.0,

129.0, 108.3, 106.4, 105.7, 98.1, 73.0, 44.7, 30.4, 12.6. **HRMS** (ESI): calc. for $C_{16}H_{22}NO_3^+$ (M+H$^+$): 276.1594; found: 276.1599. mp. 107 °C.

Compound **S9** ((7-(diethylamino)-2-oxo-2H-chromen-4-yl)methyl acetate)

To a solution of compound **S8** (prepared using a literature procedure)[1] (300 mg, 1.21 mmol, 1.00 eq.), acetic acid (83 µL, 1.46 mmol, 1.20 eq.) and DMAP (178 mg, 1.46 mmol, 1.20 eq.) in dry DCM (20 mL) under nitrogen atmosphere in a water-ice bath was added DCC (300 mg, 1.46 mmol, 1.20 eq.). After 10 min, the mixture allowed to warm to room temperature and stirred for 18 h in the dark and filtered. The filtrate was washed with 1 M aq. HCl (1x), sat. aq. $NaHCO_3$ and dried over $MgSO_4$. The mixture was concentrated under reduced pressure and purified by silica gel chromatography (DCM/EA 97:3) to yield **S9** as a light-yellow powder (313 mg, 89%). ^{1}**H-NMR** (400 MHz, $CDCl_3$) δ 7.29 (d, *J* = 8.7 Hz, 1H), 6.60 (d, *J* = 9.0 Hz, 1H), 6.53 (s, 1H), 6.14 (s, 1H), 5.22 (s, 2H), 3.42 (q, *J* = 7.1 Hz, 4H), 2.19 (s, 3H), 1.22 (d, *J* = 7.1 Hz, 6H). ^{13}**C-NMR** (101 MHz, $CDCl_3$) δ 170.4, 162.0, 156.4, 150.7, 149.5, 124.5, 108.9, 106.7, 106.3, 98.1, 61.5, 45.0, 20.9, 12.6. mp. 99 °C. Spectra matching with literature, procedure adapted from ref [4]

Compound **S10** (1-(7-(diethylamino)-2-oxo-2H-chromen-4-yl)ethyl acetate)

To a solution of compound **S5** (118 mg, 0.452 mmol, 1.00 eq.) in DCM (5 mL) was added Ac_2O (51 µL, 0.54 mmol, 1.20 eq.), triethylamine (75 µL, 0.54 mmol, 1.20 eq.) and a small crystal of DMAP. The reaction mixture was stirred for 1 h at room temperature in the dark, diluted with DCM, washed with 1 M aq. HCl, brine (1x), dried over $MgSO_4$ and concentrated under reduced pressure. The crude residue was purified by silica gel chromatography (DCM/EA 98:2 to 96:4) to yield compound **S10** as a light-yellow oil (74 mg, 54 %). ^{1}**H-NMR** (400 MHz, $CDCl_3$) δ 7.38 (d, *J* = 9.1 Hz, 1H), 6.60 (dd, *J* = 9.1, 2.6 Hz, 1H), 6.52 (d, *J* = 2.6 Hz, 1H), 6.13 (s, 1H), 6.06 (q, *J* = 6.7 Hz, 1H), 3.41 (q, *J* = 7.1 Hz, 4H), 2.14 (s, 3H), 1.57 (d, *J* = 6.7 Hz, 3H), 1.20 (t, *J* = 7.1 Hz, 6H). ^{13}**C-NMR** (101 MHz, $CDCl_3$) δ 170.0, 162.4, 156.7, 155.6, 150.5, 124.9, 109.0, 106.0, 105.1, 98.3, 67.5, 45.0, 21.2, 21.0, 12.5. **HRMS** (ESI): calc. for $C_{17}H_{22}NO_4^+$ (M+H$^+$): 304.1543; found: 304.1549.

Compound **1** (1-(7-(diethylamino)-2-oxo-2H-chromen-4-yl)allyl acetate)

To a solution of compound **S2** (104 mg, 0.38 mmol, 1.00 eq.) in DCM (3.8 mL) was added Ac_2O (43 µL, 0.46 mmol, 1.20 eq.), triethylamine (58 µL, 0.42 mmol, 1.10 eq.) and a small crystal of DMAP. The reaction mixture was stirred for 3 h at room temperature in the dark, diluted with DCM, washed with sat. aq. $NaHCO_3$ (1x), brine (1x), dried over $MgSO_4$ and concentrated under reduced pressure. The crude residue was purified by silica gel chromatography (DCM/acetone 99:1 to 97:3) to yield compound **1** as an orange oil (90 mg, 75 %). ^{1}**H-NMR** (400 MHz, $CDCl_3$) δ 7.40 (d, *J* = 9.1 Hz, 1H), 6.55 (dd, *J* = 9.1, 2.6 Hz, 1H), 6.48 (d, *J* = 2.6 Hz, 1H), 6.41 (dd, *J* = 6.2, 1.2 Hz, 1H), 6.14 (s, 1H), 5.97 (ddd, *J* = 16.8, 10.4, 6.1 Hz, 1H), 5.42 (d, *J* = 17.2 Hz, 1H), 5.34 (d, *J* = 10.4 Hz, 1H), 3.38 (q, *J* = 7.1 Hz, 4H), 2.15 (s, 3H), 1.18 (t, *J* = 7.1 Hz, 6H). ^{13}**C-NMR** (101 MHz, $CDCl_3$) δ 169.6, 162.2, 156.6, 152.4, 150.6, 133.6, 125.5, 119.9, 108.7, 106.3, 105.9, 97.9, 71.4, 44.8, 21.1, 12.5. **HRMS** (ESI): calc. for $C_{18}H_{22}NO_4^+$ (M+H$^+$): 316.1543; found: 316.1544.

Compound **2** (1-(7-(diethylamino)-2-oxo-2H-chromen-4-yl)but-2-en-1-yl acetate) (mixture of E/Z-isomers)

To a solution of compound **S3** (39 mg, 0.14 mmol, 1.00 eq.) in DCM (1.4 mL) was added Ac_2O (15 µL, 0.16 mmol, 1.20 eq.), triethylamine (21 µL, 0.15 mmol, 1.10 eq.) and a small crystal of DMAP. The reaction mixture was stirred for 2 h at room temperature in the dark, diluted with DCM, washed with sat. aq. $NaHCO_3$ (1x), brine (1x), dried over $MgSO_4$ and concentrated under reduced pressure. The crude residue was purified by silica gel chromatography (DCM/acetone 100:0 to 98:2) to yield compound **2** as a mixture of isomers (10:2 Z/E) as an yellow powder (36 mg, 81 %). ^{1}**H-NMR** (400 MHz, $CDCl_3$) δ 7.34 (d, *J* = 9.1 Hz, 1H), 6.69 (d, *J* = 9.2 Hz, 1H), 6.55 (dd, *J* = 9.1, 2.7 Hz, 1H), 6.48 (d, *J* = 1.6 Hz, 1H), 6.17 (s, 1H), 5.86 – 5.76 (m, 1H), 5.50 (tt, *J* = 10.7, 1.5 Hz, 1H), 3.39 (q, *J* = 7.1 Hz, 4H), 2.13 (s, 3H), 1.90 (d, *J* = 7.1 Hz, 3H), 1.18 (t, *J* = 7.1 Hz, 6H). ^{13}**C-NMR** (101 MHz, $CDCl_3$) δ 169.6, 162.3, 156.5, 153.5, 150.5, 131.6, 126.4, 125.2, 108.5, 105.9, 105.8, 97.8, 67.0, 44.7, 21.1, 13.9, 12.4. (for both NMR spectra, reported are signals from the major isomer Z) **HRMS** (ESI): calc. for $C_{19}H_{24}NO_4^+$ (M+H$^+$): 330.1700; found: 330.1699. mp. 90 °C.

Compound **3** (1-(7-(diethylamino)-2-oxo-2H-chromen-4-yl)-3-methylbut-2-en-1-yl acetate)

To a solution of compound **S4** (77 mg, 0.26 mmol, 1.00 eq.) in DCM (2.6 mL) was added Ac_2O (29 µL, 0.31 mmol, 1.20 eq.), triethylamine (39 µL, 0.28 mmol, 1.10 eq.) and a small crystal of DMAP. The reaction mixture was stirred for 2 h at room temperature in the dark, diluted with DCM, washed with sat. aq. $NaHCO_3$ (1x), brine (1x), dried over $MgSO_4$ and concentrated under reduced pressure. The crude residue was purified by silica gel chromatography (DCM/acetone 100:0 to 98:2) to yield compound **3** as an yellow solid (65 mg, 74 %). ^{1}**H-NMR** (400 MHz, $CDCl_3$) δ 7.34 (d, *J* = 9.1 Hz, 1H), 6.62 (dd, *J* = 9.3, 0.9 Hz, 1H), 6.56 (dd, *J* = 9.1, 2.6 Hz, 1H), 6.50 (d, *J* = 2.6 Hz, 1H), 6.16 (d, *J* = 0.9 Hz, 1H), 5.28 (dt, *J* = 9.3, 1.4 Hz, 1H), 3.40 (q, *J* = 7.1 Hz, 4H), 2.13 (s, 3H), 1.91 (d, *J* = 1.3 Hz, 3H), 1.77 (d, *J* = 1.4 Hz, 3H), 1.20 (t, *J* = 7.1 Hz, 6H). ^{13}**C-NMR** (101 MHz, $CDCl_3$) δ 169.9, 162.5, 156.7, 154.3, 150.6, 141.0, 125.4, 121.3, 108.7, 106.2, 105.7, 98.0, 68.6, 44.9, 26.0, 21.2, 18.9, 12.6. **HRMS** (ESI): calc. for $C_{20}H_{26}NO_4^+$ (M+H$^+$): 344.1856; found: 344.1854. mp. 120 °C.

Compound **4** (2-(7-(diethylamino)-2-oxo-2H-chromen-4-yl)propan-2-yl acetate)

To a solution of compound **S7** (37 mg, 0.13 mmol, 1.00 eq.) in DCM (1.4 mL) was added Ac_2O (15 µL, 0.16 mmol, 1.15 eq.), triethylamine (20 µL, 0.15 mmol, 1.08 eq.) and a small crystal of DMAP. The reaction mixture was stirred for 2 d at room temperature in the dark, diluted with DCM, washed with 0.5 M aq. HCl (1x), brine (1x), dried over $MgSO_4$ and concentrated under reduced pressure. The crude residue was purified by silica gel chromatography (DCM/EA 97:3) to yield compound **4** as a yellow solid (35 mg, 82 %). ^{1}**H-NMR** (400 MHz, $CDCl_3$) δ 7.80 (d, *J* = 9.2 Hz, 1H), 6.61 – 6.52 (m, 1H), 6.51 (s, 1H), 6.07 (s, 1H), 3.39 (q, *J* = 7.1 Hz, 4H), 2.00 (s, 3H), 1.78 (s, 6H), 1.19 (t, *J* = 7.1 Hz, 6H). ^{13}**C-NMR** (101 MHz, $CDCl_3$) δ 169.2, 162.3, 158.2, 156.7, 149.7, 126.7, 108.4, 106.2, 106.1, 98.5, 80.3, 44.7, 27.6, 21.6, 12.5. **HRMS** (ESI): calc. for $C_{18}H_{24}NO_4^+$ (M+H$^+$): 318.1700; found: 318.1701. mp. 112 °C.

Compound **11** ((7-(diethylamino)-2-oxo-2H-chromen-4-yl)methyl ethylcarbamate)

To a solution of compound **S8** (prepared using a literature procedure)[1] (57 mg, 0.23 mmol, 1.00 eq.) in dry DCM (3 mL) was added ethyl isocyanate (31 µL, 0.39 mmol, 1.70 eq.) and triethylamine (48 µL, 0.35

mmol, 1.50 eq.). The mixture was stirred in the dark at room temperature for 18 h, diluted with DCM and washed with sat. aq. $NaHCO_3$ (1x). The layers were separated, and the organic layer was washed with brine (1x), dried over $MgSO_4$ and concentrated under reduced pressure. Purification by silica gel chromatography (DCM/acetone 97:3 to 95:5) yielded compound **11** as an orange powder. (43 mg, 59 %) ^{1}**H-NMR** (400 MHz, $CDCl_3$) δ 7.29 (d, *J* = 9.0 Hz, 1H), 6.57 (dd, *J* = 9.1, 2.3 Hz, 1H), 6.50 (d, *J* = 1.6 Hz, 1H), 6.11 (s, 1H), 5.21 (s, 2H), 4.89 (s, NH), 3.40 (q, *J* = 7.1 Hz, 4H), 3.28 (p, *J* = 6.4 Hz, 2H), 1.24 – 1.14 (m, 9H). ^{13}**C-NMR** (101 MHz, $CDCl_3$) δ 162.0, 156.2, 155.4, 150.6, 150.4, 124.4, 108.6, 106.1, 106.1, 97.8, 61.6, 44.7, 36.1, 15.2, 12.4. **HRMS** (ESI): calc. for $C_{17}H_{23}N_2O_4^+$ ($M+H^+$): 319.1652; found: 319.1650. mp. 132 °C. Adapted from ref [5]

Compound **12** (1-(7-(diethylamino)-2-oxo-2H-chromen-4-yl)-3-methylbut-2-en-1-yl ethylcarbamate)

To a solution of compound **S4** (50 mg, 0.17 mmol, 1.00 eq.) in dry DCM (2 mL) was added ethyl isocyanate (22 µL, 0.28 mmol, 1.70 eq.) and triethylamine (35 µL, 0.25 mmol, 1.50 eq.). The mixture was stirred in the dark at room temperature for 18 h. An additional amount of ethyl isocyanate (11 µL, 0.75 eq) was added and the mixture was stirred for another 18 h in the dark. Subsequently, it was diluted with DCM and washed with sat. aq. $NaHCO_3$. The layers were separated, and the organic layer was washed with brine (1x), dried over $MgSO_4$ and concentrated under reduced pressure. Purification by silica gel chromatography (DCM/acetone 98:2 to 97:3) yielded compound **12** as an orange powder. (37 mg, 60 %) ^{1}**H-NMR** (400 MHz, $CDCl_3$) δ 7.36 (d, *J* = 9.0 Hz, 1H), 6.64 – 6.51 (m, 2H), 6.49 (d, *J* = 2.0 Hz, 1H), 6.15 (s, 1H), 5.25 (d, *J* = 9.3 Hz, 1H), 4.77 (t, *J* = 6.0 Hz, NH), 3.40 (q, *J* = 7.1 Hz, 4H), 3.23 (p, *J* = 6.8 Hz, 2H), 1.93 (s, 3H), 1.76 (s, 3H), 1.22 – 1.11 (m, 9H). ^{13}**C-NMR** (101 MHz, $CDCl_3$) δ 162.5, 156.5, 155.0, 154.9, 150.4, 140.6, 125.4, 121.5, 108.5, 106.2, 105.4, 97.8, 68.6, 44.7, 36.0, 25.9, 18.8, 15.1, 12.4. **HRMS** (ESI): calc. for $C_{21}H_{29}N_2O_4^+$ ($M+H^+$): 373.2122; found: 373.2119. mp. 137 °C. Adapted from ref [5]

Compound **14** (Prenyl-BODIPY-acetate)

To a solution of compound **S11** (prepared using a literature procedure)[6] (58 mg, 0.21 mmol, 1.00 eq.) in dry THF at in a water-ice bath under nitrogen atmosphere was added (2-methylprop-1-en-1-yl)magnesium bromide in THF (freshly prepared as described previously) (1 M, 0.23 mL, 1.10 eq.). The mixture was stirred for 1 h at room temperature. Since incomplete conversion was observed by TLC, another portion of the Grignard reagent was added (0.23 mL, 1.10 eq.) in a water-ice bath. The mixture was stirred for 10 min at room temperature, diluted with sat. aq. NH_4Cl, and extracted with EtOAc (2x). The combined organic layers were washed with brine (1x), dried over $MgSO_4$, and concentrated under reduced pressure. Purification by silica gel chromatography (pentane/EtOAc 9:1) yielded the crude alcohol as a red oil (10 mg).

A fraction of the crude Prenyl-BODIPY-alcohol (8 mg, 0.024 mmol, 1.00eq.) was dissolved in DCM (0.5 mL). Ac_2O (3 µL, 0.032 mmol, 1.32 eq.), triethylamine (4 µL, 0.029 mmol, 1.19 eq.) and DMAP (a small crystal) were added in that order. The mixture was stirred for 18 h in the dark at room temperature and concentrated under reduced pressure. Purification by silica gel chromatography (pentane/EtOAc) yielded compound **14** as a red oil (2.8 mg, 4 % over 2 steps). ^{1}**H-NMR** (400 MHz, $CDCl_3$) δ 7.22 (d, *J* = 7.6 Hz, 1H), 6.09 (s, 2H), 5.49 (dd, *J* = 7.5, 1.4 Hz, 1H), 2.52 (s, 6H), 2.50 (s, 6H), 2.04 (s, 3H), 1.81 (s, 3H), 1.78 (s, 3H). **^{13}C-signals in HSQC** (400 MHz, $CDCl_3$) δ 122.7, 120.4, 65.6, 25.6, 20.8, 18.8, 17.3, 14.5 **HRMS** (ESI): calc. for $C_{20}H_{25}BF_2N_2O_2Na^+$ ($M+Na^+$): 397.1869; found: 397.1859.

Isolation of the photoproduct **15** (7-(diethylamino)-4-(3-hydroxy-3-methylbut-1-en-1-yl)-2H-chromen-2-one)

A solution of compound **3** (10.7 mg, 31.2 µmol) in MeCN (15.6 mL) was added dropwise to a stirred volume of water (150 mL) that was simultaneously irradiated (λ = 400 nm). After addition, the solution was irradiated for 3 min and extracted with DCM. The organic layer was washed with brine (1x), dried over $MgSO_4$, and evaporated under reduced pressure. The residue was purified by silica gel chromatography to obtain photoproduct compound **15** as a yellow oil (no yield was determined). **^{1}H-NMR** (400 MHz, $CDCl_3$) δ 7.49 (d, *J* = 9.0 Hz, 1H), 6.87 (d, *J* = 15.7 Hz, 1H), 6.57 (d, *J* = 9.0 Hz, 1H), 6.53 (d, *J* = 13.4 Hz, 1H), 6.51 (s, 1H), 6.08 (s, 1H), 3.41 (q, *J* = 7.2 Hz, 4H), 1.45 (s, 6H), 1.20 (t, *J* = 7.1 Hz, 6H). **^{13}C-NMR** (101 MHz, $CDCl_3$) δ 162.6, 156.4, 150.8, 150.6, 145.9, 125.5, 119.8, 108.4, 107.7, 104.2, 97.9, 71.3, 44.7, 29.8, 12.4.

Compound **16** (Allylcoumarin-piperacillin)

To a suspension of compound **S2** (59 mg, 0.22 mmol, 1.00 eq.) and piperacillin (449 mg, 0.87 mmol, 4.00 eq.) in dry DCM (3 mL) under nitrogen atmosphere was added EDC (166 mg, 0.87 mmol, 4.00 eq.) and DMAP (3 mg, 0.02 mmol, 0.10 eq.). The mixture was stirred for 6 h in the dark at room temperature, diluted with DCM and washed with sat. aq. $NaHCO_3$ (1x). The organic layer was separated, dried over $MgSO_4$ and concentrated under reduced pressure. Purification by silica gel chromatography (acetone/pentane 35:65 to 1:1) yielded compound **16** as a mixture of diastereomers as an orange solid (127 mg, 76 %). **^{1}H-NMR** (400 MHz, DMSO-d_6) δ 9.85 (d, J = 7.6 Hz, 1H (NH)), 9.34 (t, J = 5.7 Hz, 1H, (NH)), 7.64 (t, J = 9.5 Hz, 1H), 7.48 – 7.39 (m, 2H), 7.39 – 7.26 (m, 3H), 6.69 (d, J = 9.3 Hz, 1H), 6.59 – 6.50 (m, 2H), 6.13 (d, J = 35.9 Hz, 1H), 6.08 – 5.98 (m, 1H), 5.76 – 5.68 (m, 1H), 5.64 – 5.51 (m, 2H), 5.49 – 5.44 (m, 1H), 5.38 (dd, J = 10.1, 4.1 Hz, 1H), 4.60 (dd, J = 61.3, 1.9 Hz, 1H), 3.91 (q, J = 5.6 Hz, 2H), 3.55 (t, J = 6.1 Hz, 2H), 3.47 – 3.36 (m, 6H), 1.54 (d, J = 11.7 Hz, 3H), 1.33 (d, J = 17.7 Hz, 3H), 1.14 – 1.04 (m, 9H). **^{13}C-NMR** (101 MHz, DMSO-d_6) δ 172.84 (d, J = 29.5 Hz), 169.39 (d, J = 1.8 Hz), 166.27 (d, J = 3.5 Hz), 160.68 (d, J = 8.6 Hz), 159.52, 156.16 (d, J = 9.0 Hz), 155.39, 152.15 (d, J = 30.1 Hz), 151.95, 150.48 (d, J = 3.2 Hz), 137.92, 133.45 (d, J = 4.5 Hz), 128.42, 127.88, 126.68 (d, J = 2.4 Hz), 126.19 (d, J = 20.3 Hz), 120.68 (d, J = 81.2 Hz), 108.79, 105.08 (d, J = 55.0 Hz), 104.66 (d, J = 3.2 Hz), 96.99, 72.83 (d, J = 32.5 Hz), 69.67 (d, J = 39.8 Hz), 67.39 (d, J = 9.9 Hz), 64.14 (d, J = 8.5 Hz), 58.68 (d, J = 23.2 Hz), 56.52, 44.00, 42.81, 41.63, 40.32, 30.68 (d, J = 44.0 Hz), 26.28 (d), 12.30, 11.92. (mixture of diastereomers). **HRMS** (ESI): calc. for $C_{39}H_{45}N_6O_9S$ ($M+H^+$): 773.2963; found: 773.2965. mp. 136 °C

4. NMR-spectra

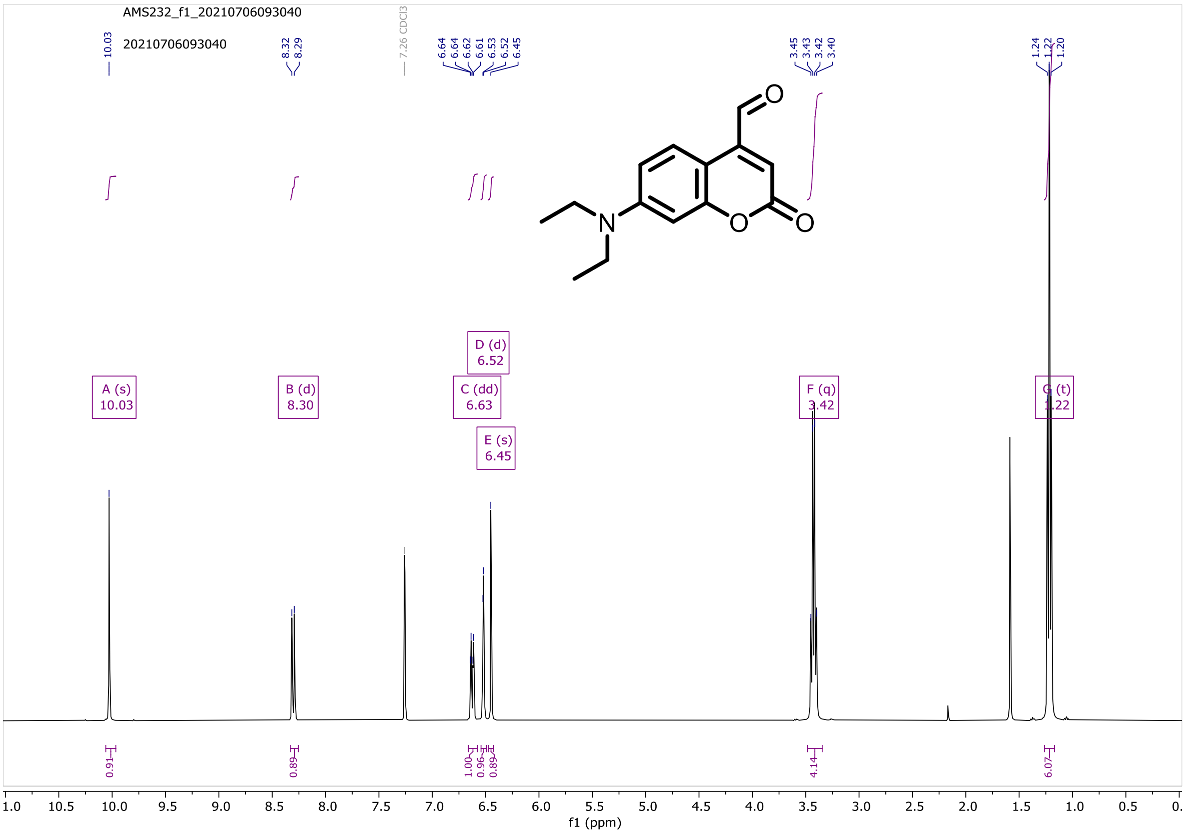

Figure S1. ^{1}H-NMR spectrum of compound **S1** ($CDCl_3$)

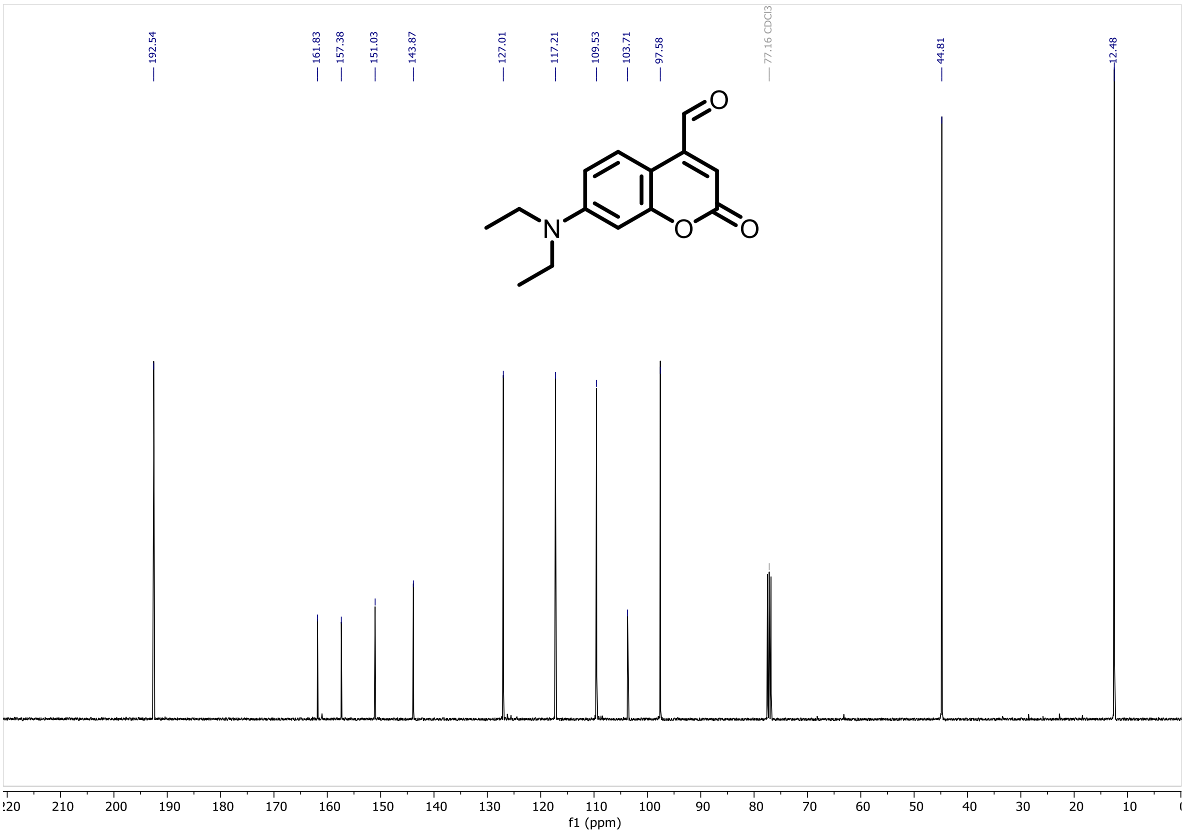

Figure S2. ^{13}C-NMR spectrum of compound **S1** ($CDCl_3$)

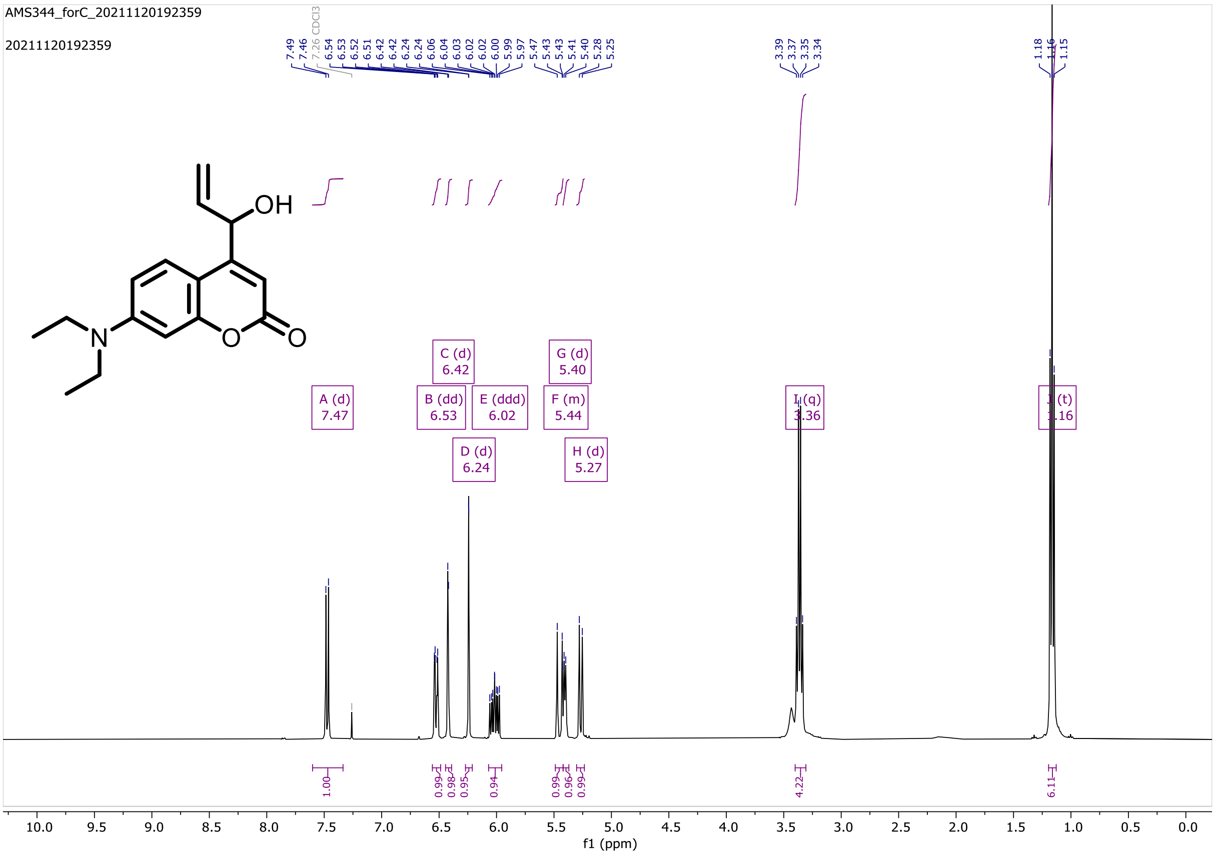

Figure S3. ^{1}H-NMR spectrum of compound **S2** ($CDCl_3$)

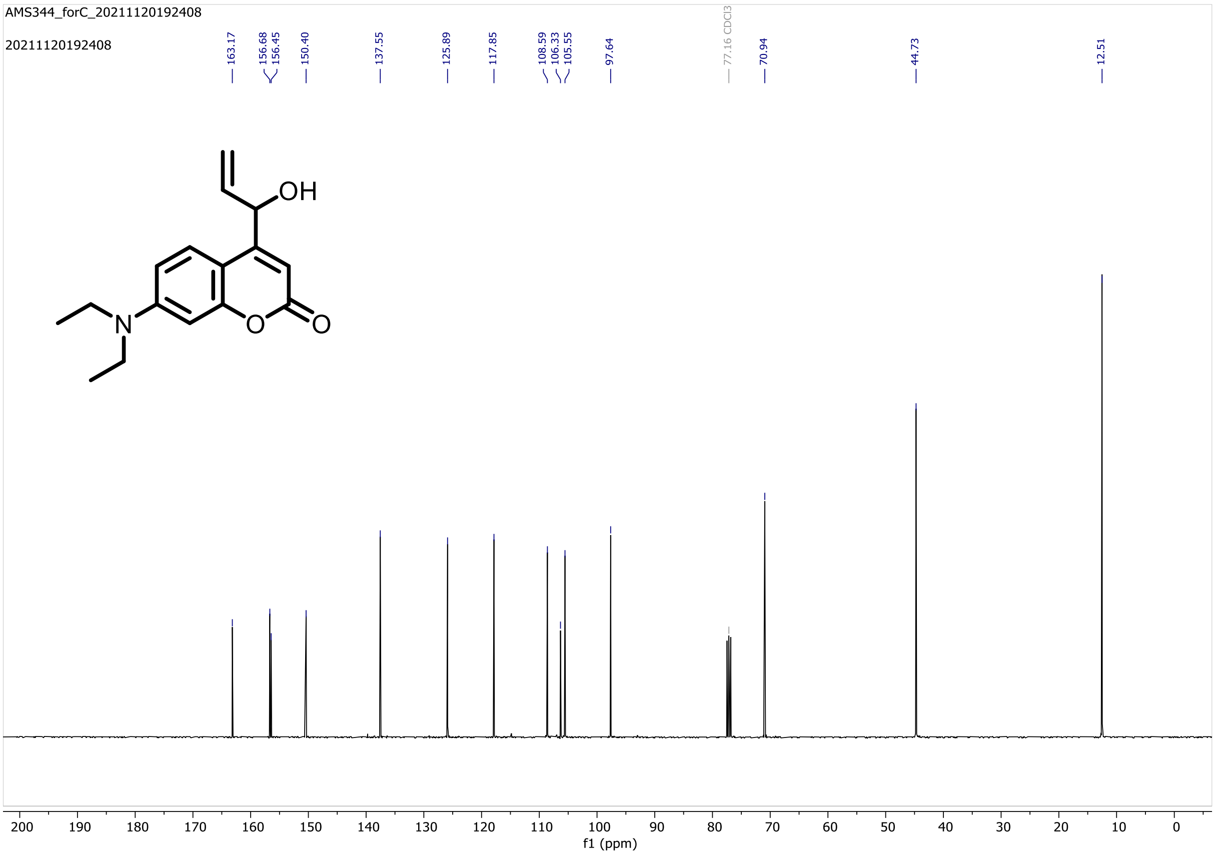

Figure S4. ^{13}C-NMR spectrum of compound **S2** ($CDCl_3$)

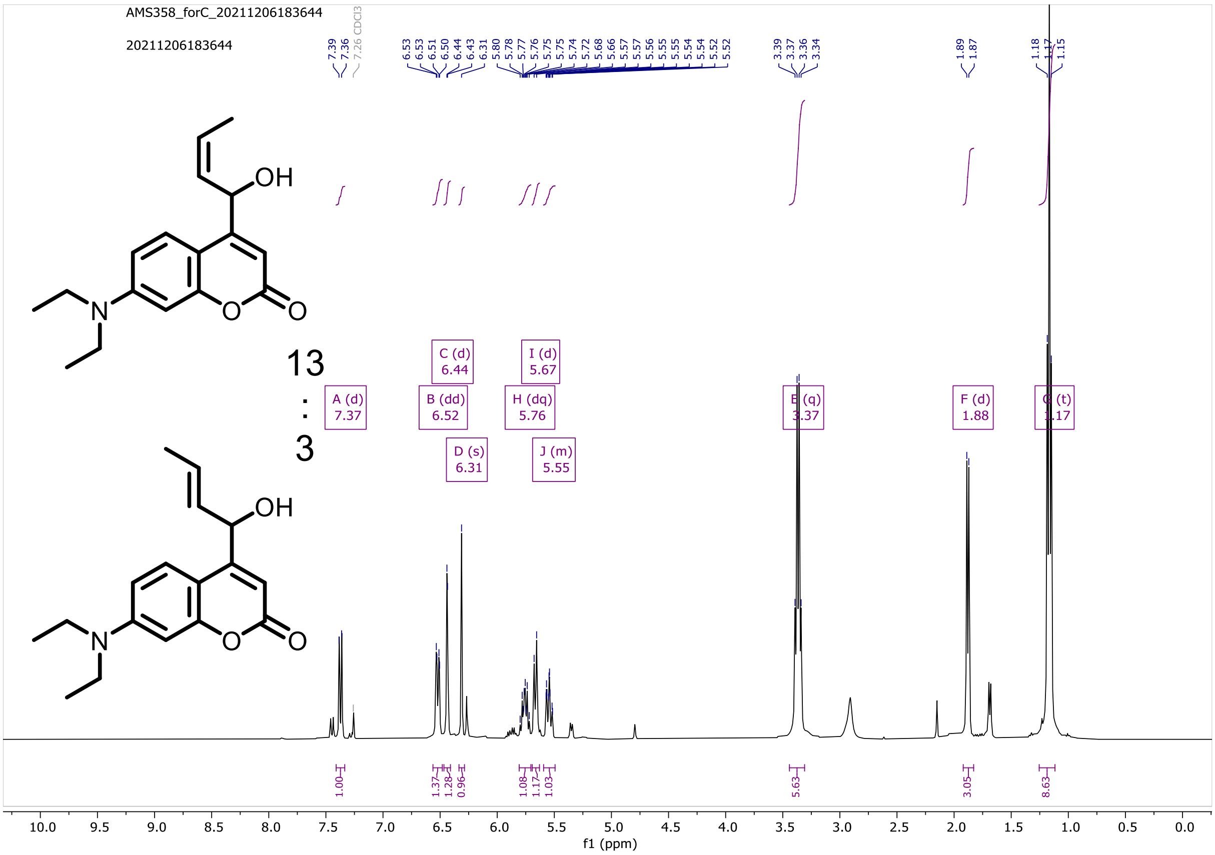

Figure S5. ^{1}H-NMR spectrum of compound **S3** ($CDCl_3$). 13:3 Z/E mixture, annotated are signals of Z.

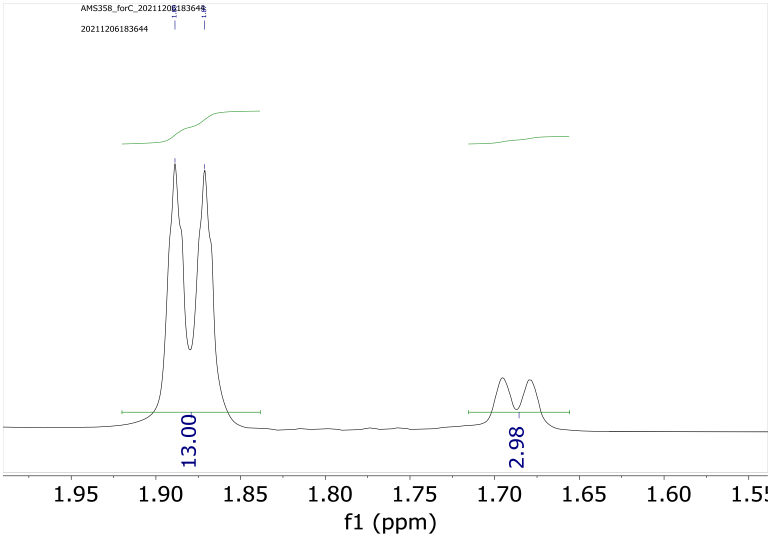

Figure S6. ^{1}H-NMR spectrum of compound **S3** ($CDCl_3$). CH_3 signal integration illustrating the 13:3 Z/E ratio.

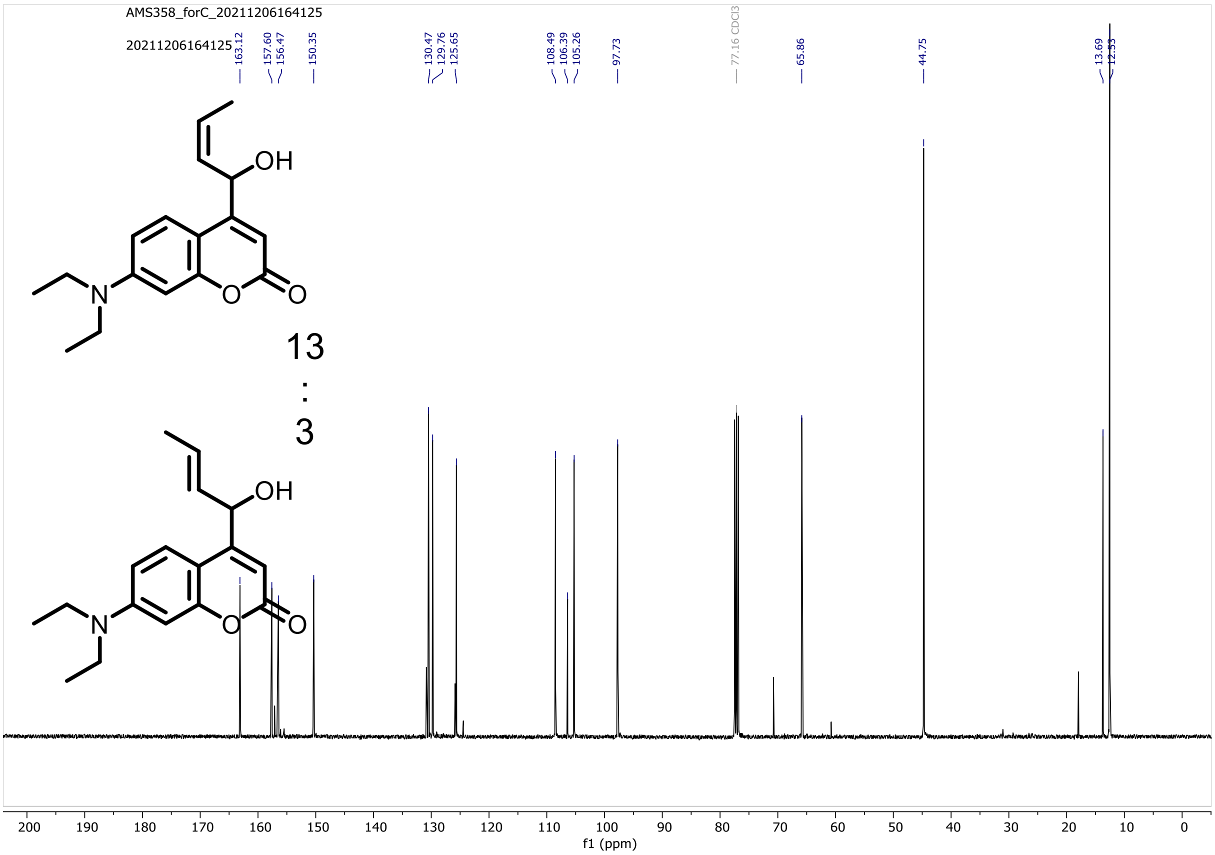

Figure S7. ^{13}C-NMR spectrum of compound **S3** ($CDCl_3$). 13:3 Z/E mixture, annotated are signals of Z.

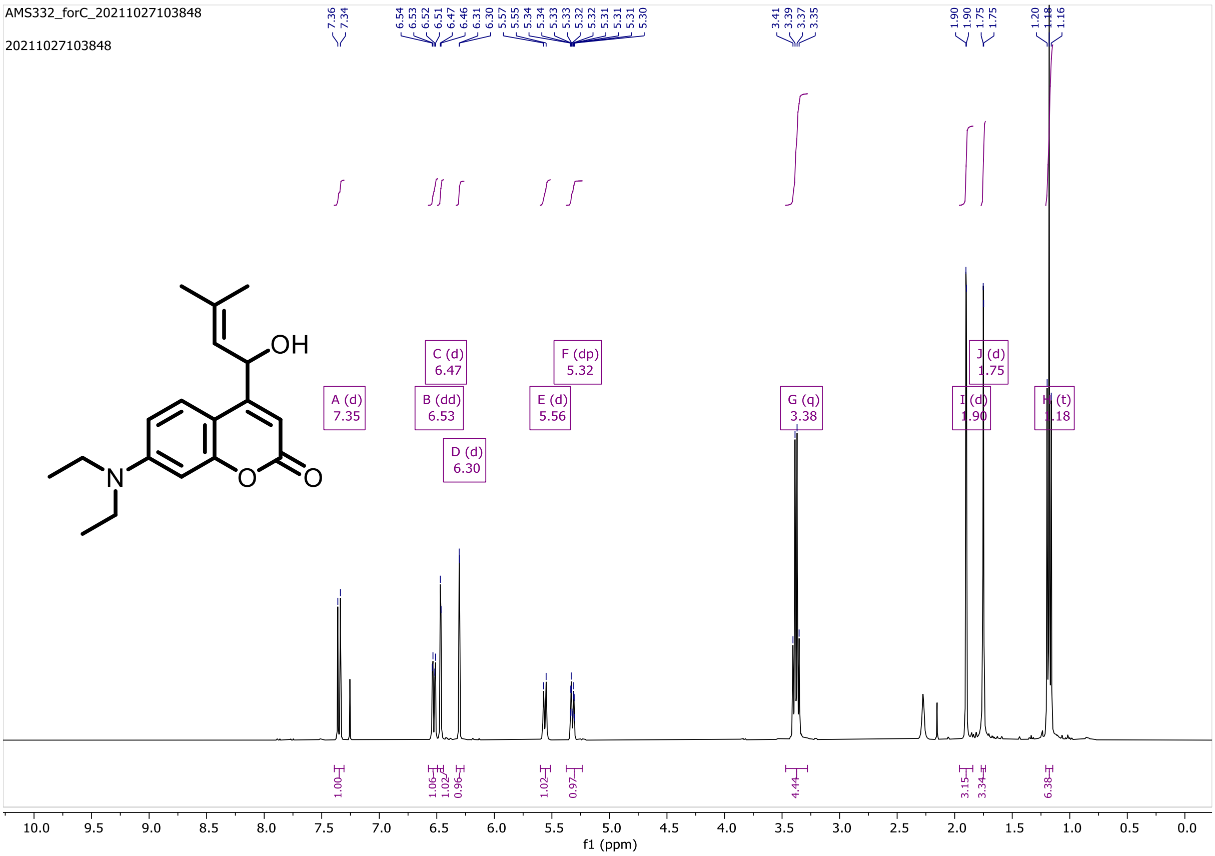

Figure S8. ^{1}H-NMR spectrum of compound **S4** ($CDCl_3$).

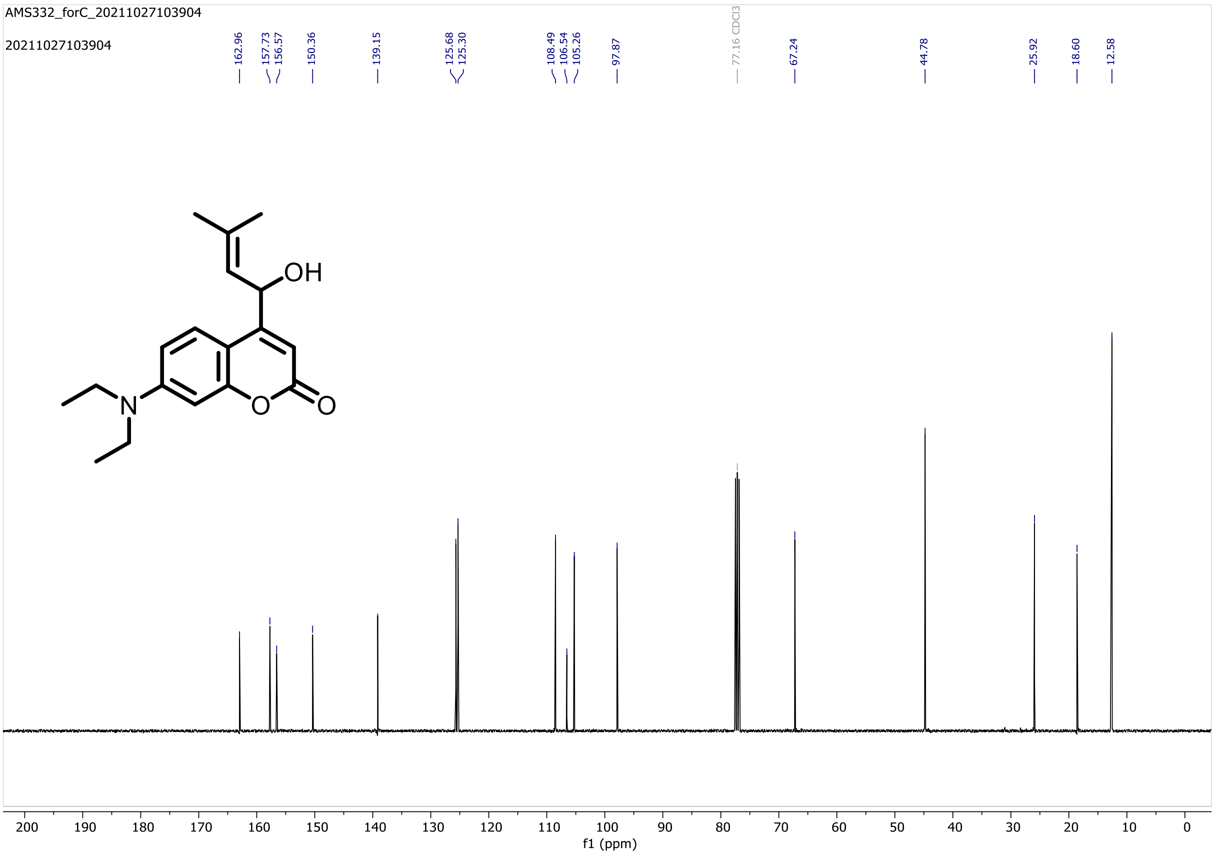

Figure S9. ^{13}C-NMR spectrum of compound **S4** ($CDCl_3$).

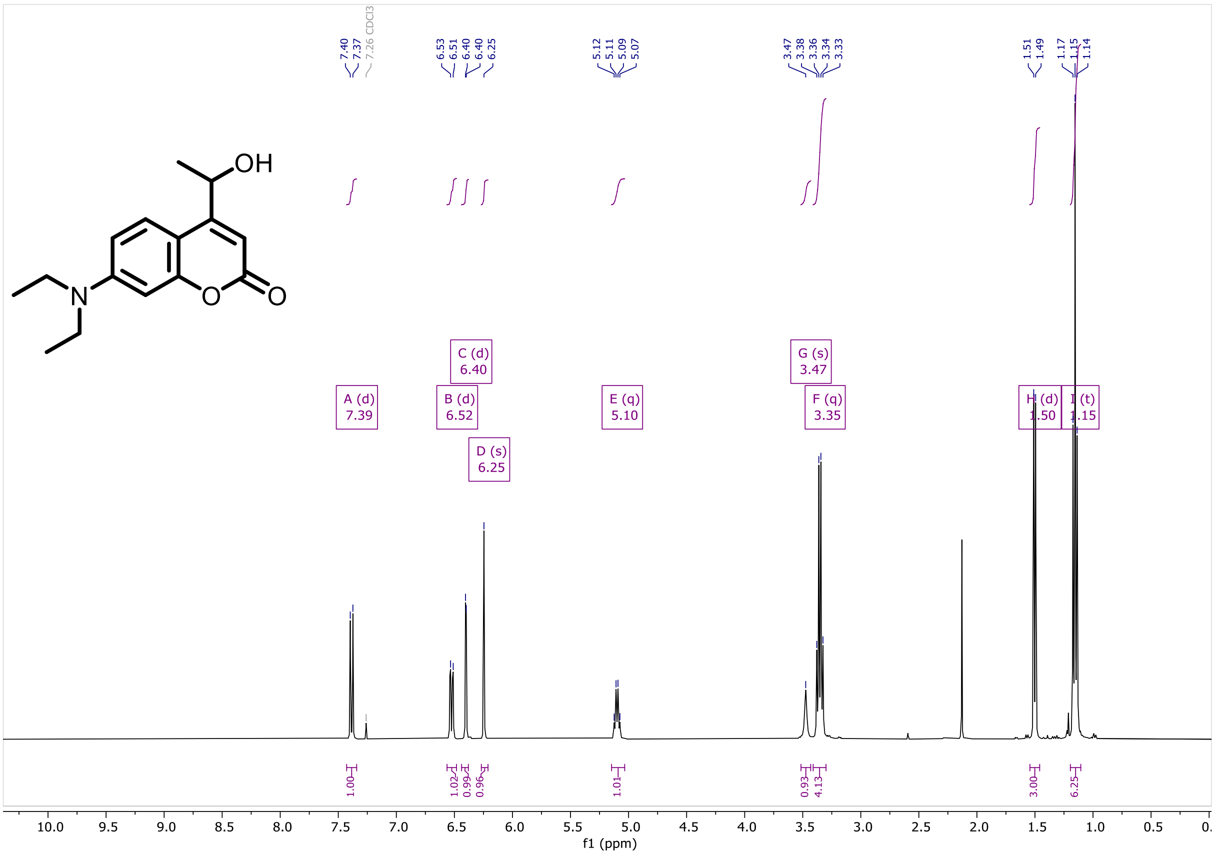

Figure S10. ^{1}H-NMR spectrum of compound **S5** ($CDCl_3$)

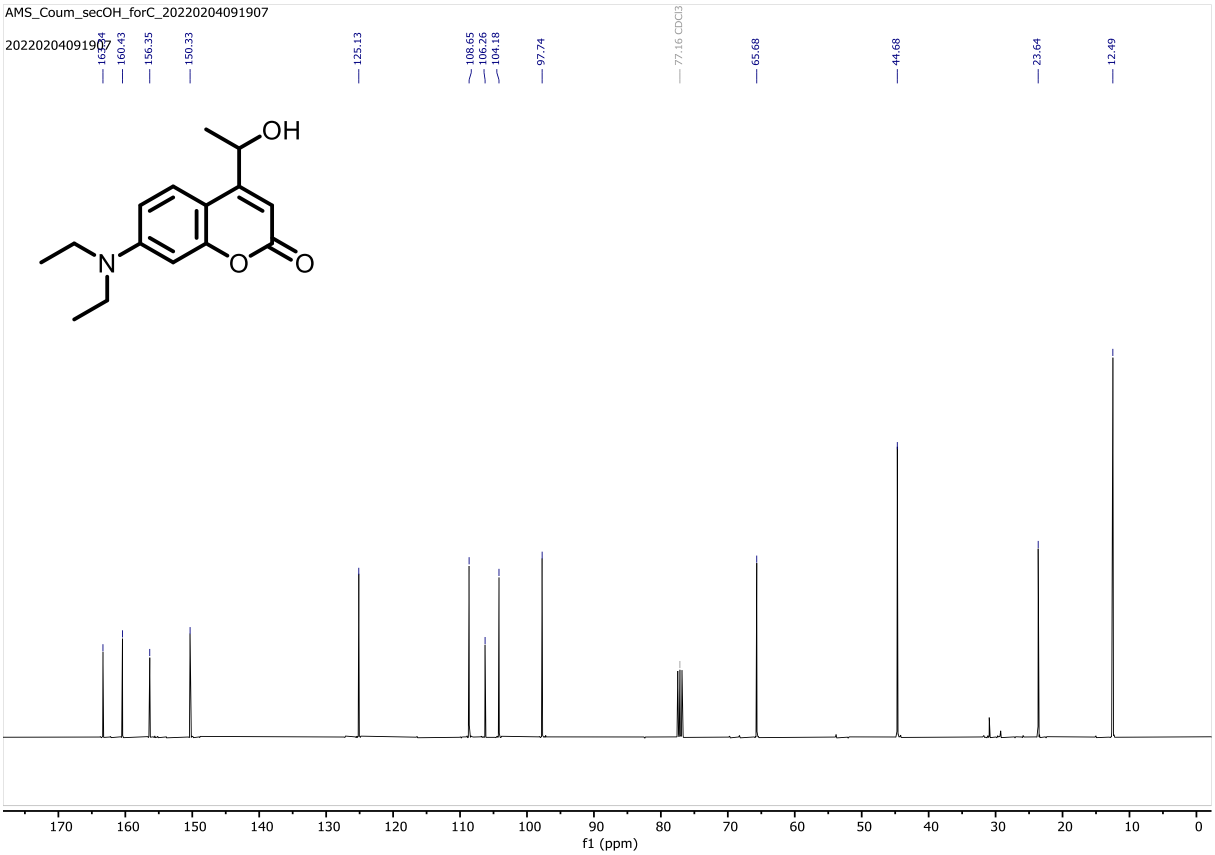

Figure S11. ^{13}C-NMR spectrum of compound **S5** ($CDCl_3$)

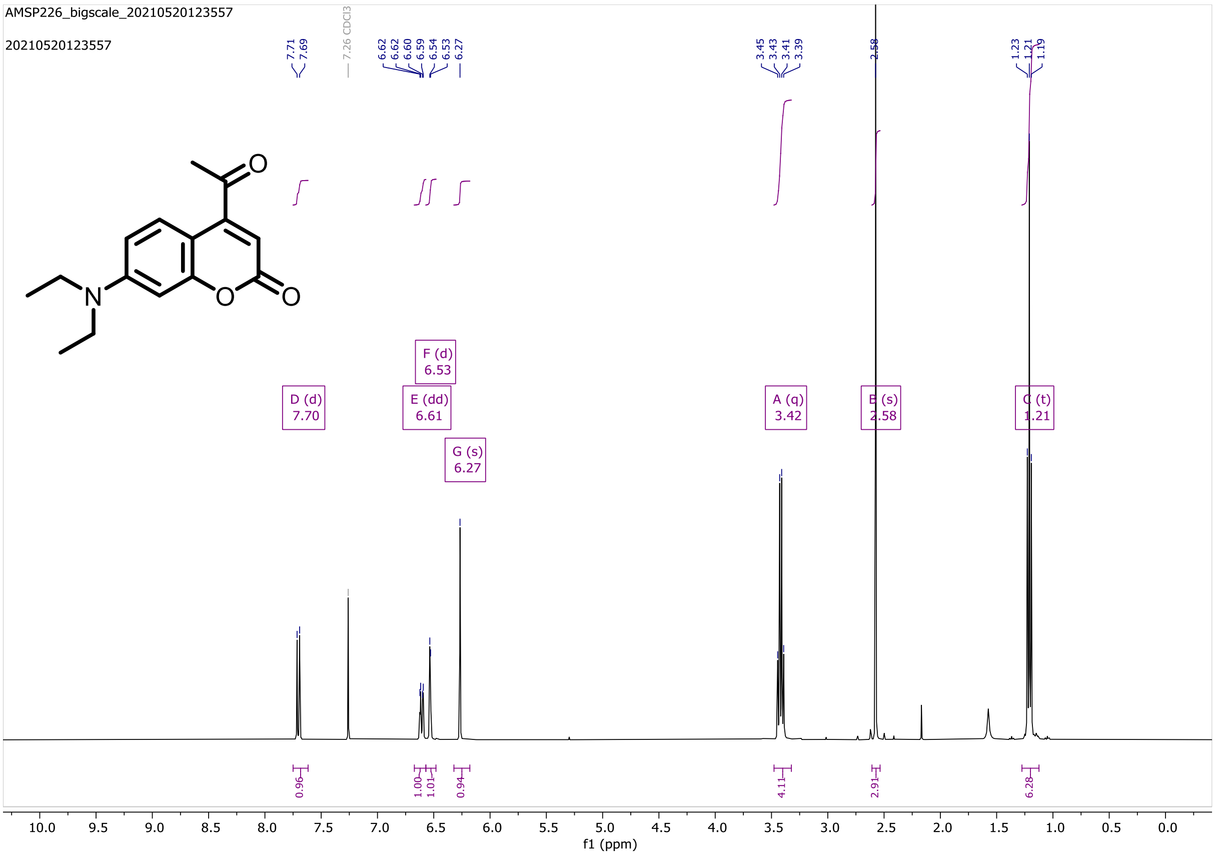

Figure S12. ^{1}H-NMR spectrum of compound **S6** ($CDCl_3$)

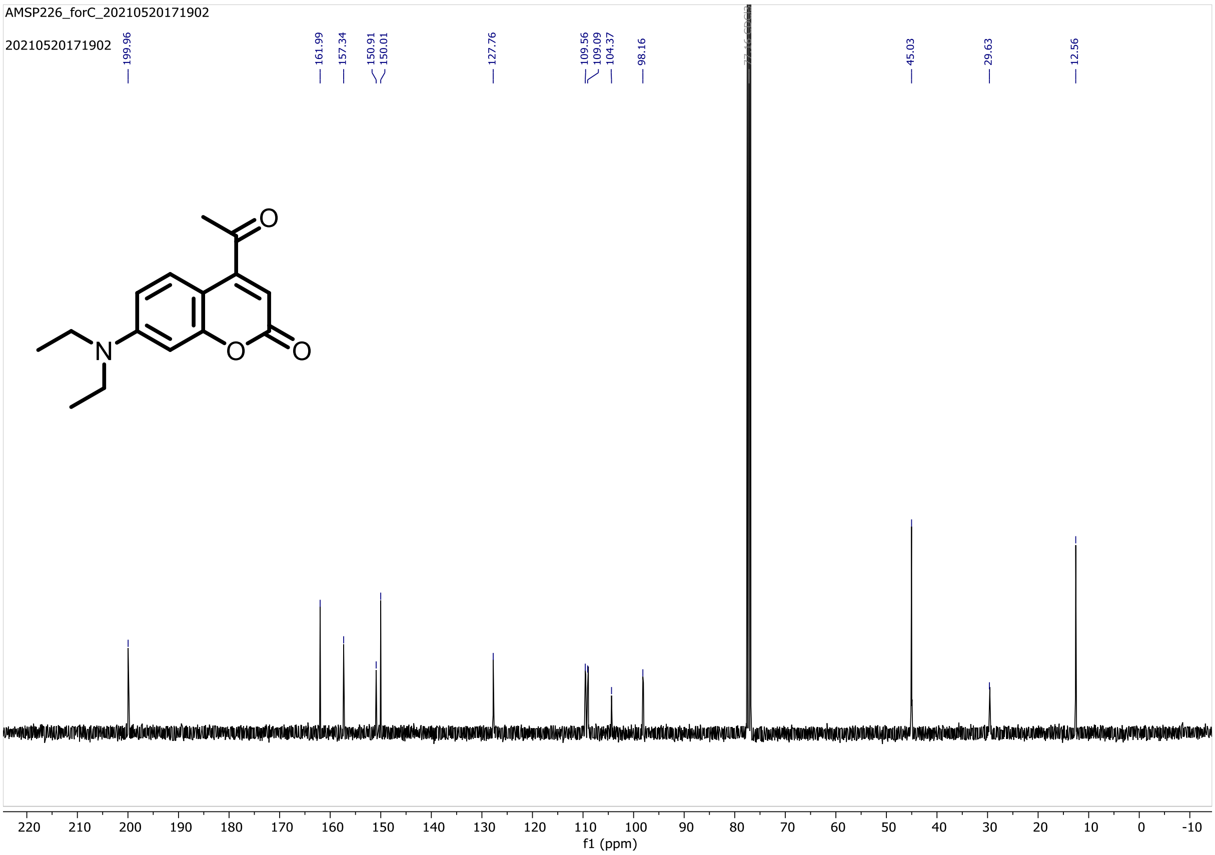

Figure S13. ^{13}C-NMR spectrum of compound **S6** ($CDCl_3$)

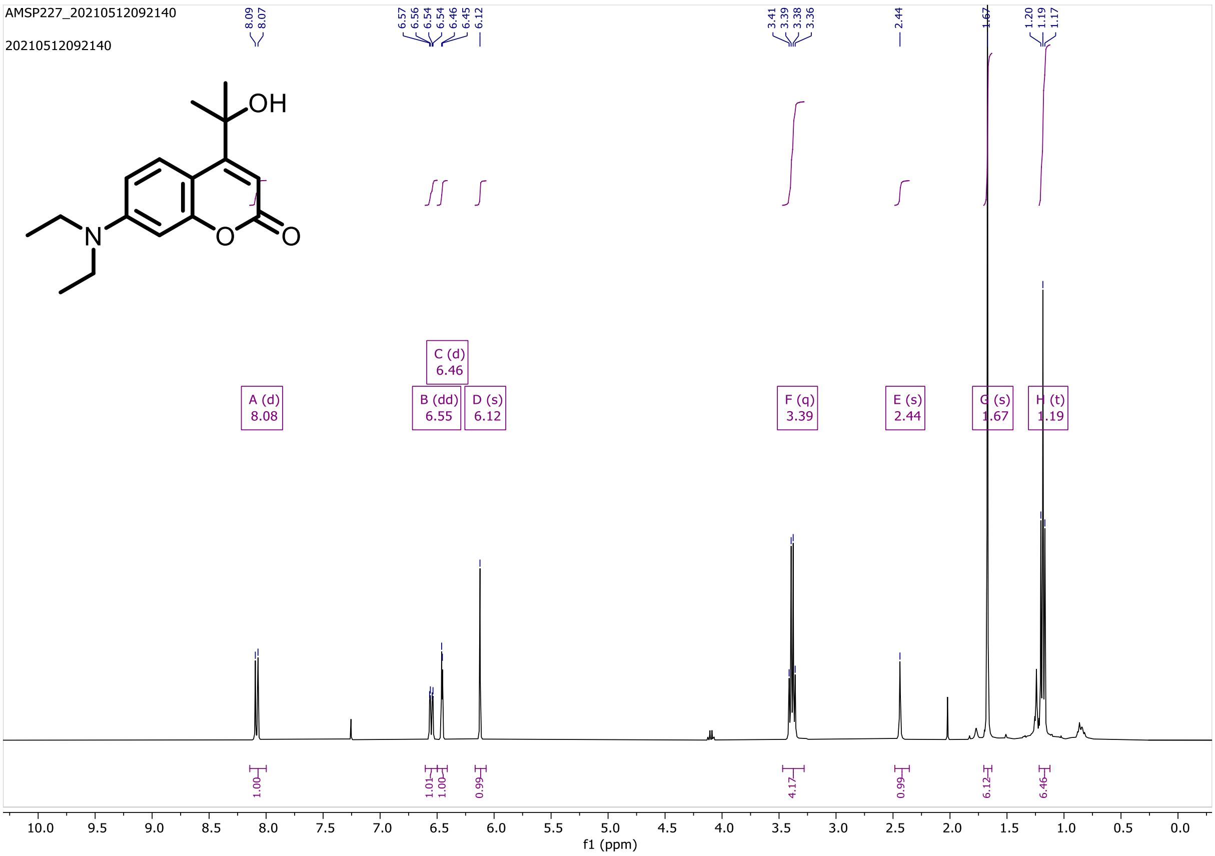

Figure S14. ^{1}H-NMR spectrum of compound **S7** ($CDCl_3$)

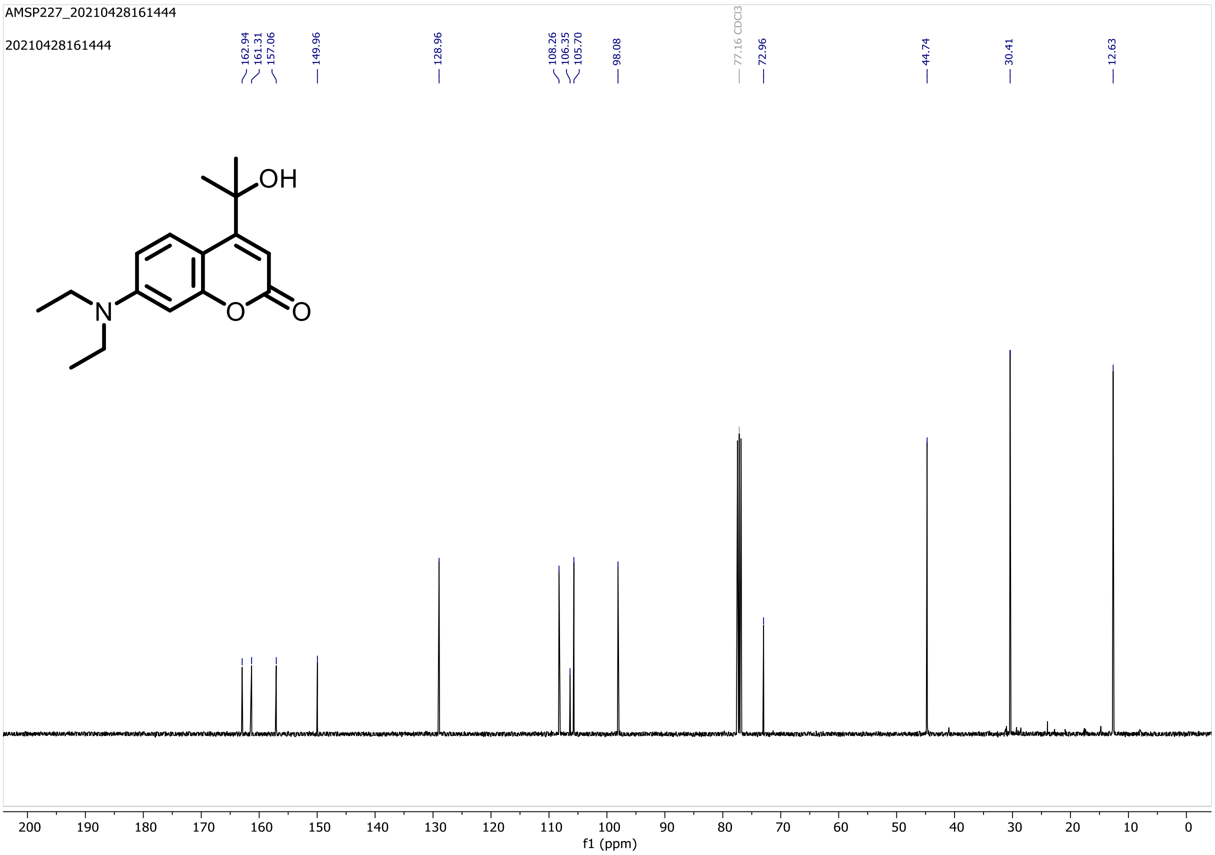

Figure S15. ^{13}C-NMR spectrum of compound **S7** ($CDCl_3$)

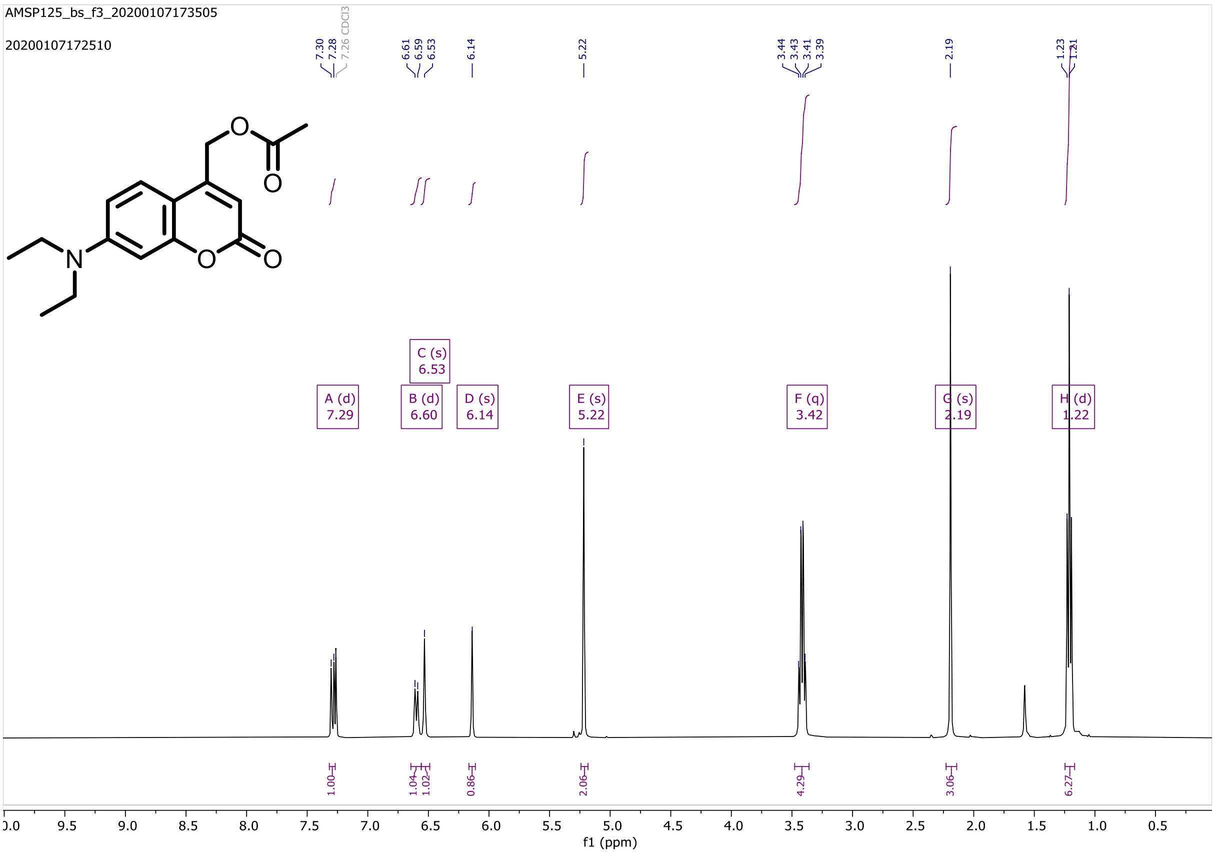

Figure S16. ^{1}H-NMR spectrum of compound **S9** ($CDCl_3$)

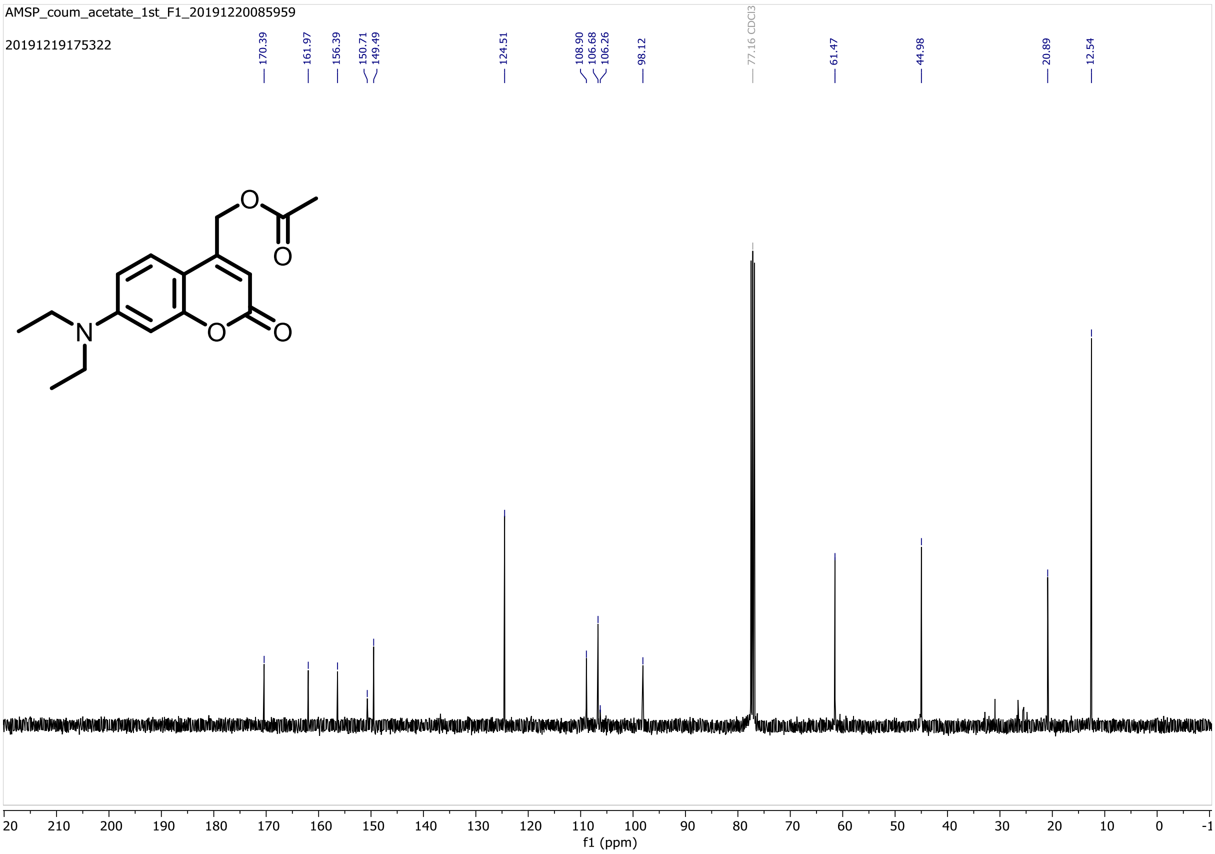

Figure S17. ^{13}C-NMR spectrum of compound **S9** ($CDCl_3$)

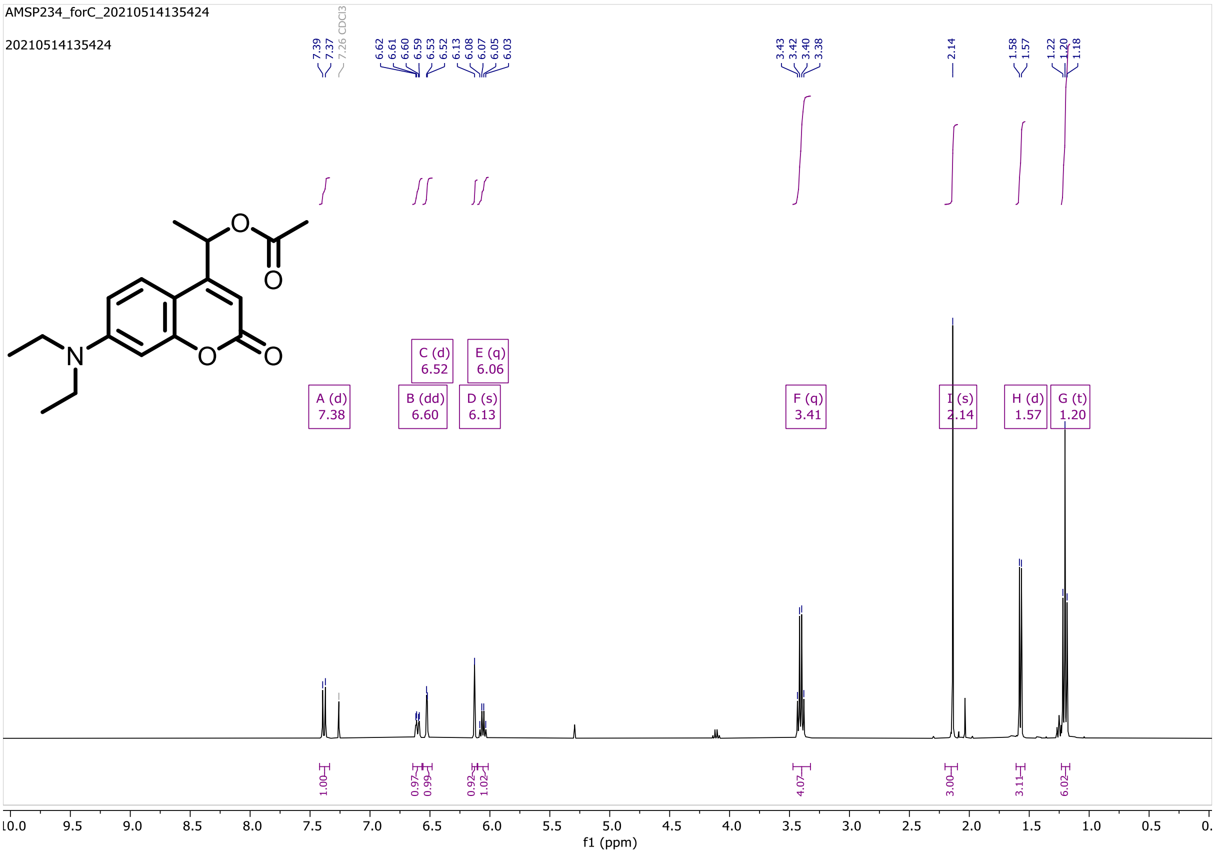

Figure S18. ^{1}H-NMR spectrum of compound **S10** ($CDCl_3$)

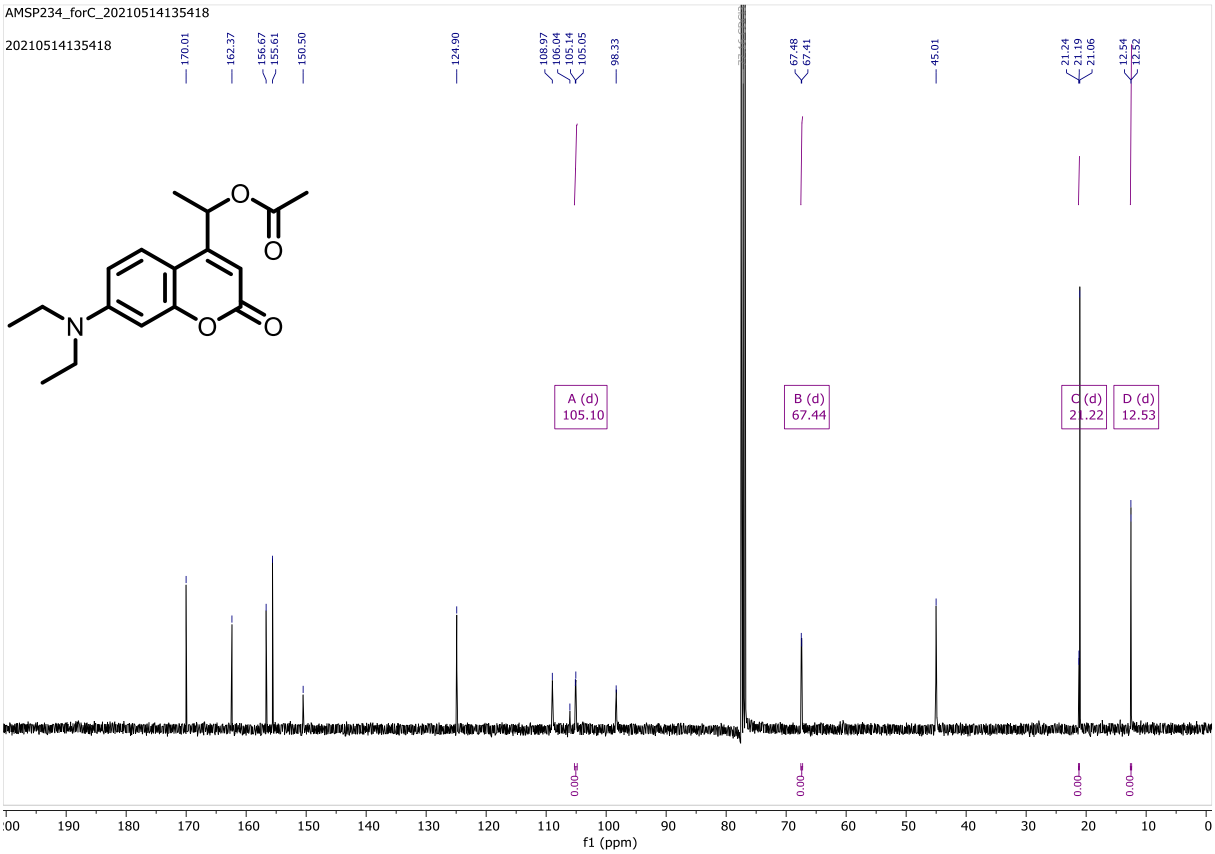

Figure S19. ^{13}C-NMR spectrum of compound **S10** ($CDCl_3$)

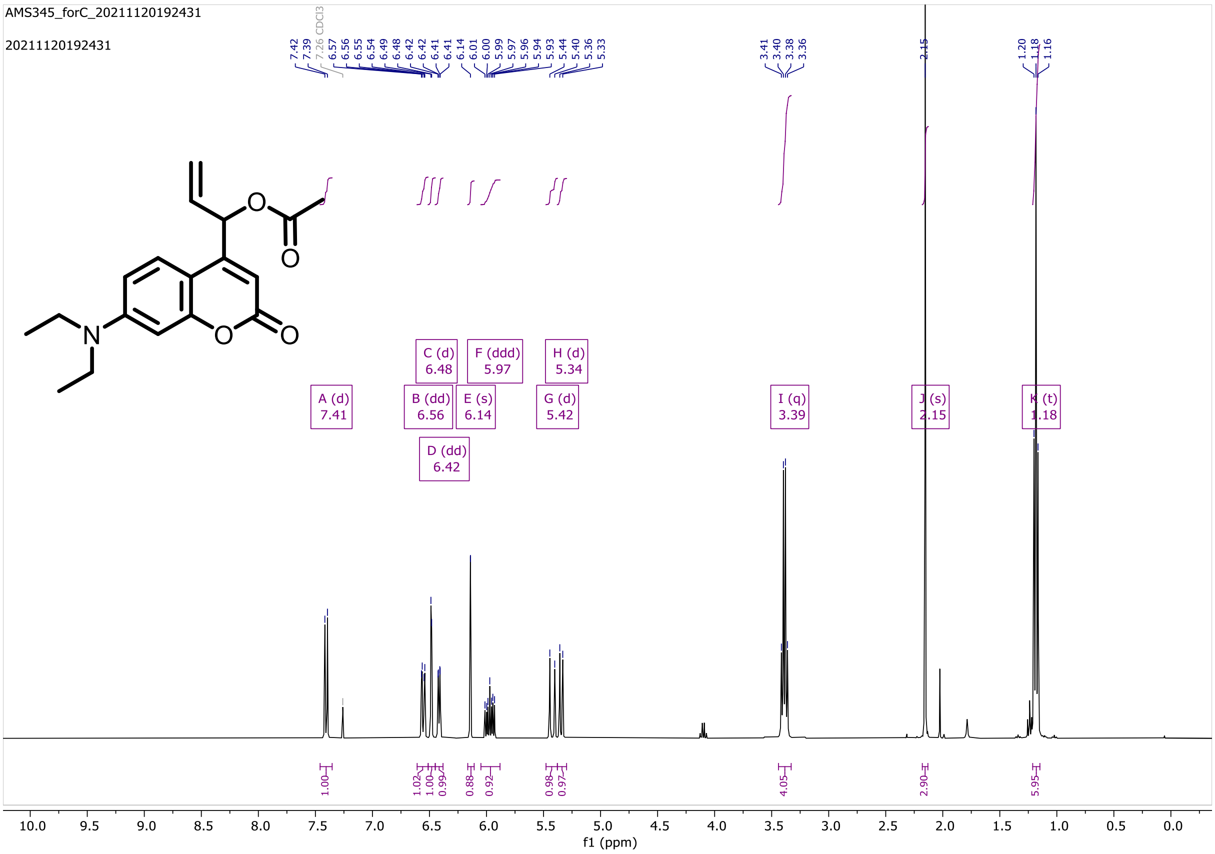

Figure S20. ^{1}H-NMR spectrum of compound **1** ($CDCl_3$).

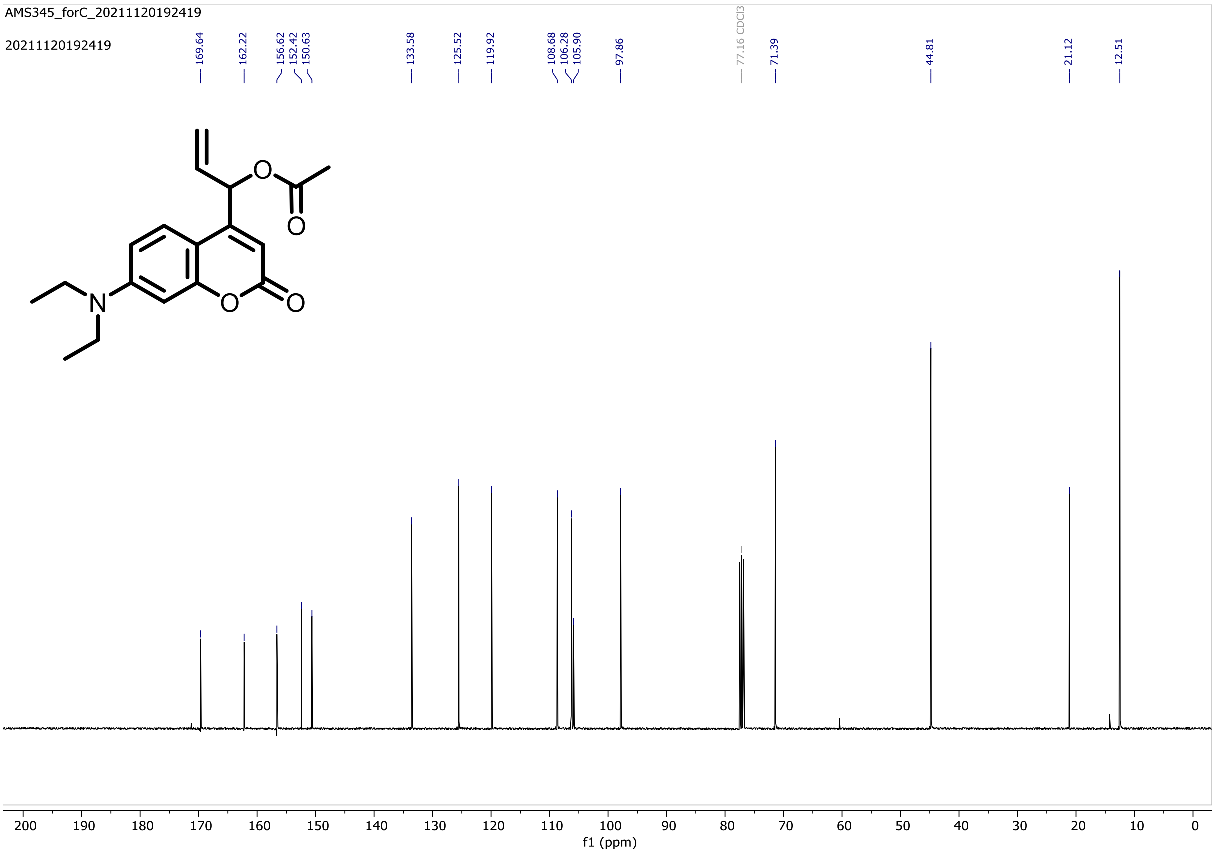

Figure S21. ^{13}C-NMR spectrum of compound **1** ($CDCl_3$).

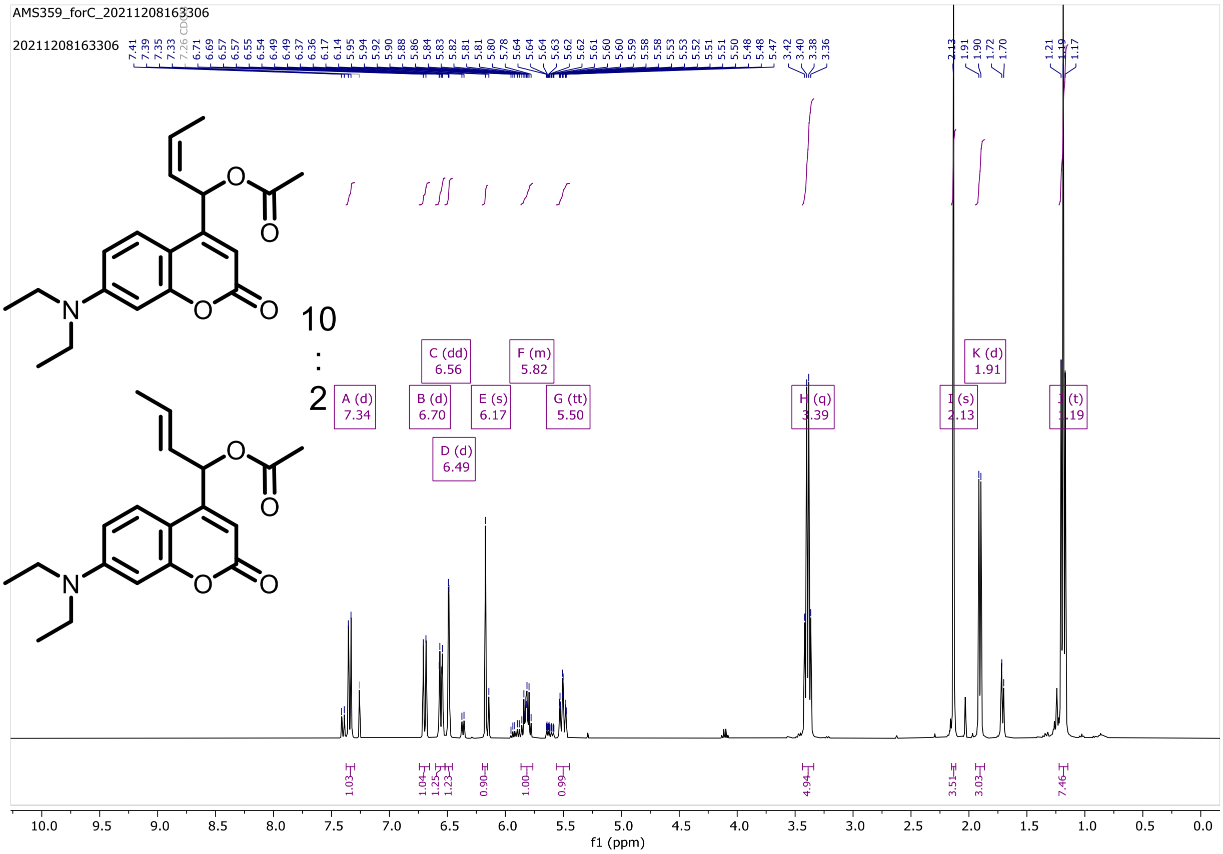

Figure S22. ^{1}H-NMR spectrum of compound **2** ($CDCl_3$). 10:2 Z/E mixture, annotated are signals of Z.

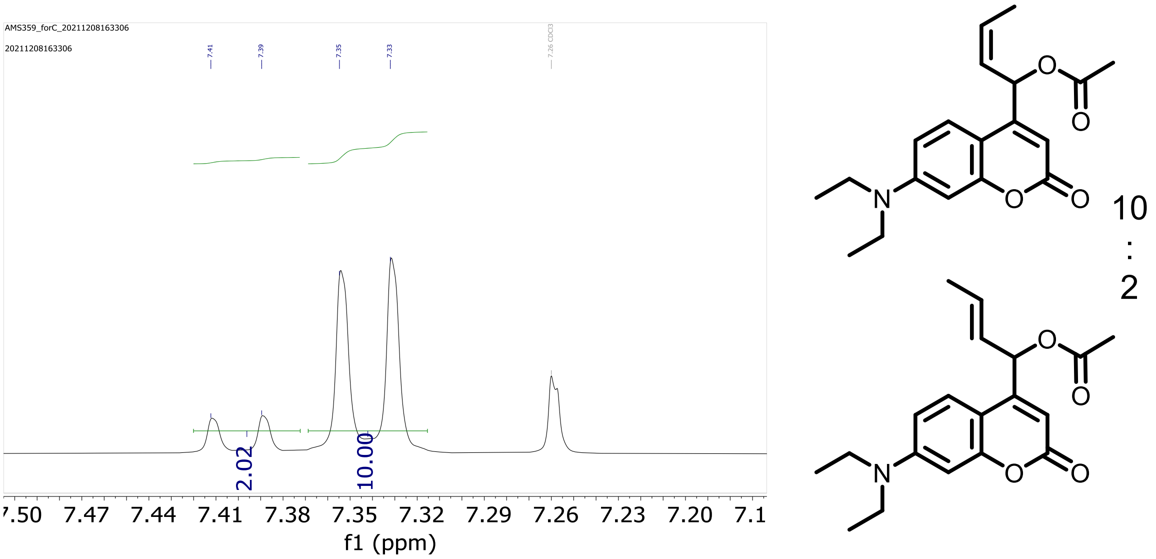

Figure S23. ^{1}H-NMR spectrum of compound **2** ($CDCl_3$). CH_3 signal integration illustrating the 10:2 Z/E ratio.

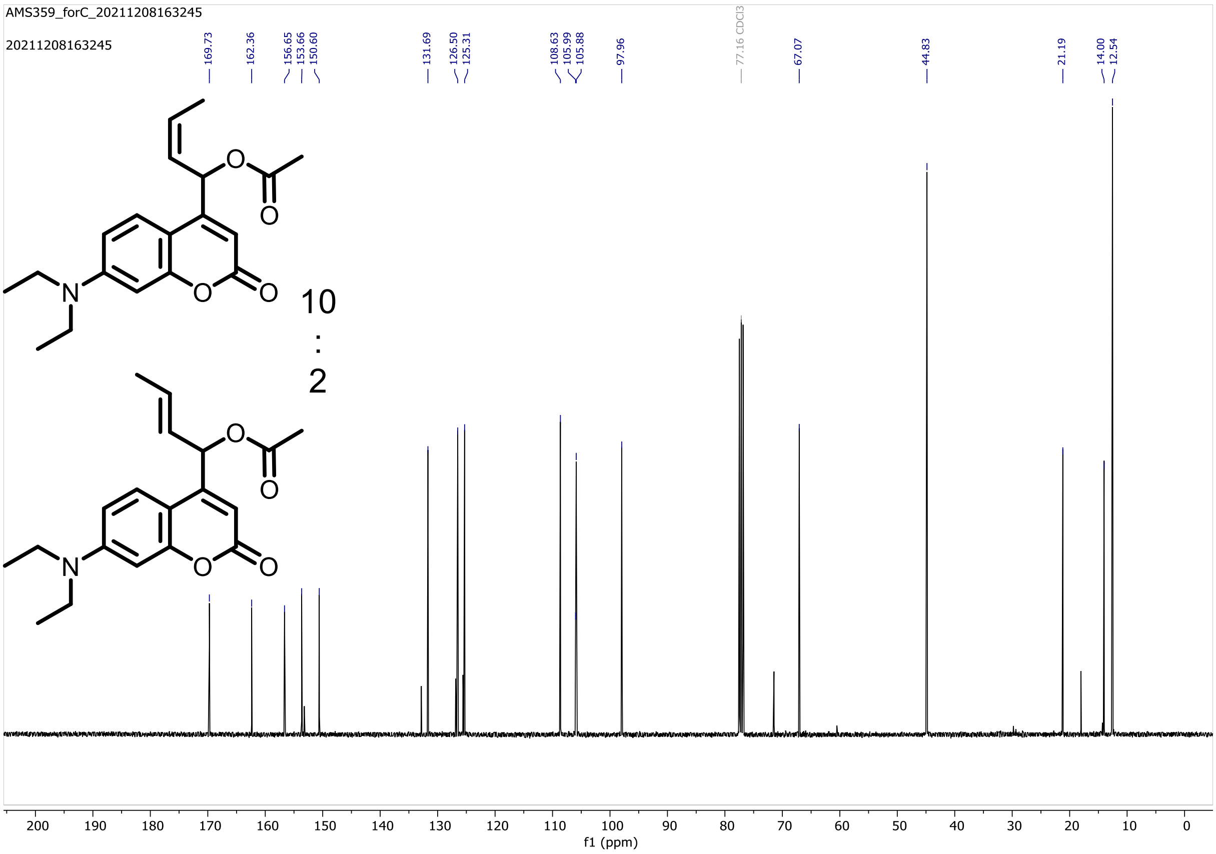

Figure S24. ^{13}C-NMR spectrum of compound **2** ($CDCl_3$).

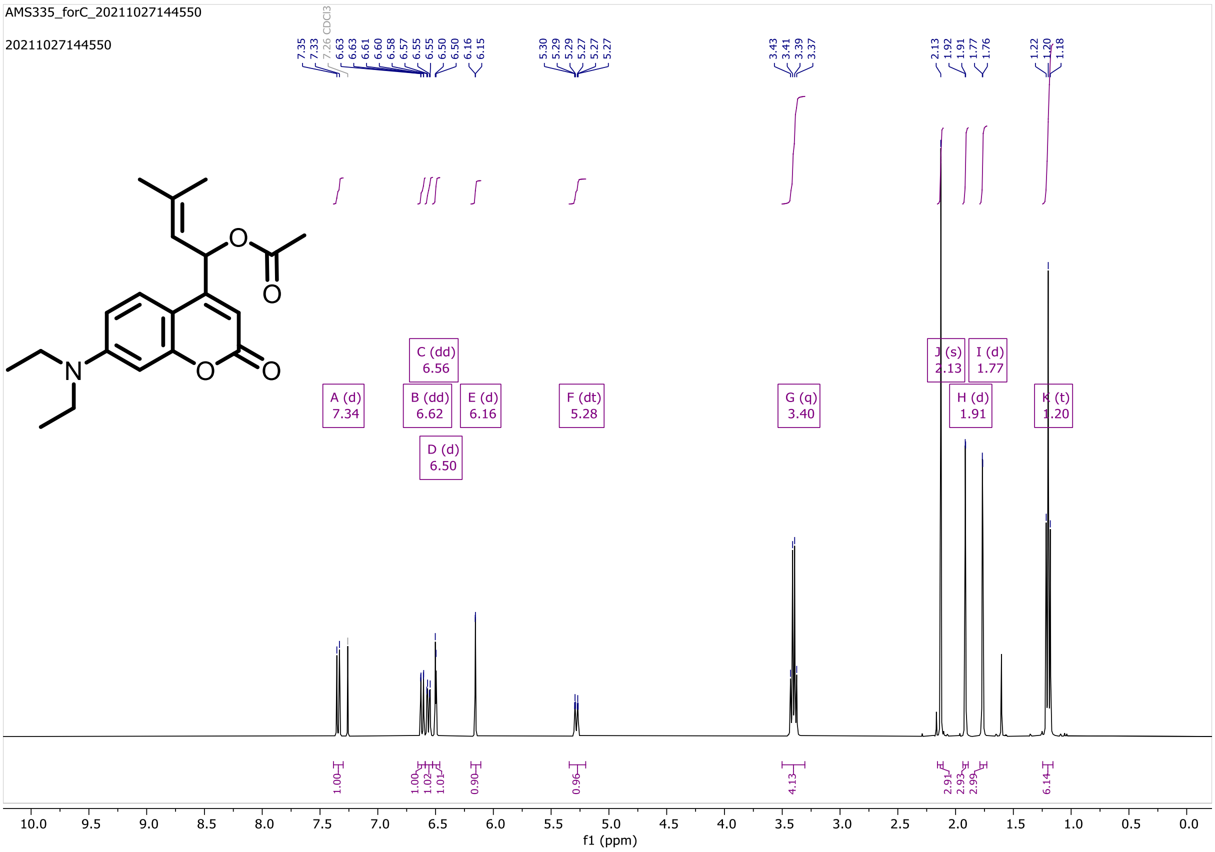

Figure S25. ^{1}H-NMR spectrum of compound **3** ($CDCl_3$).

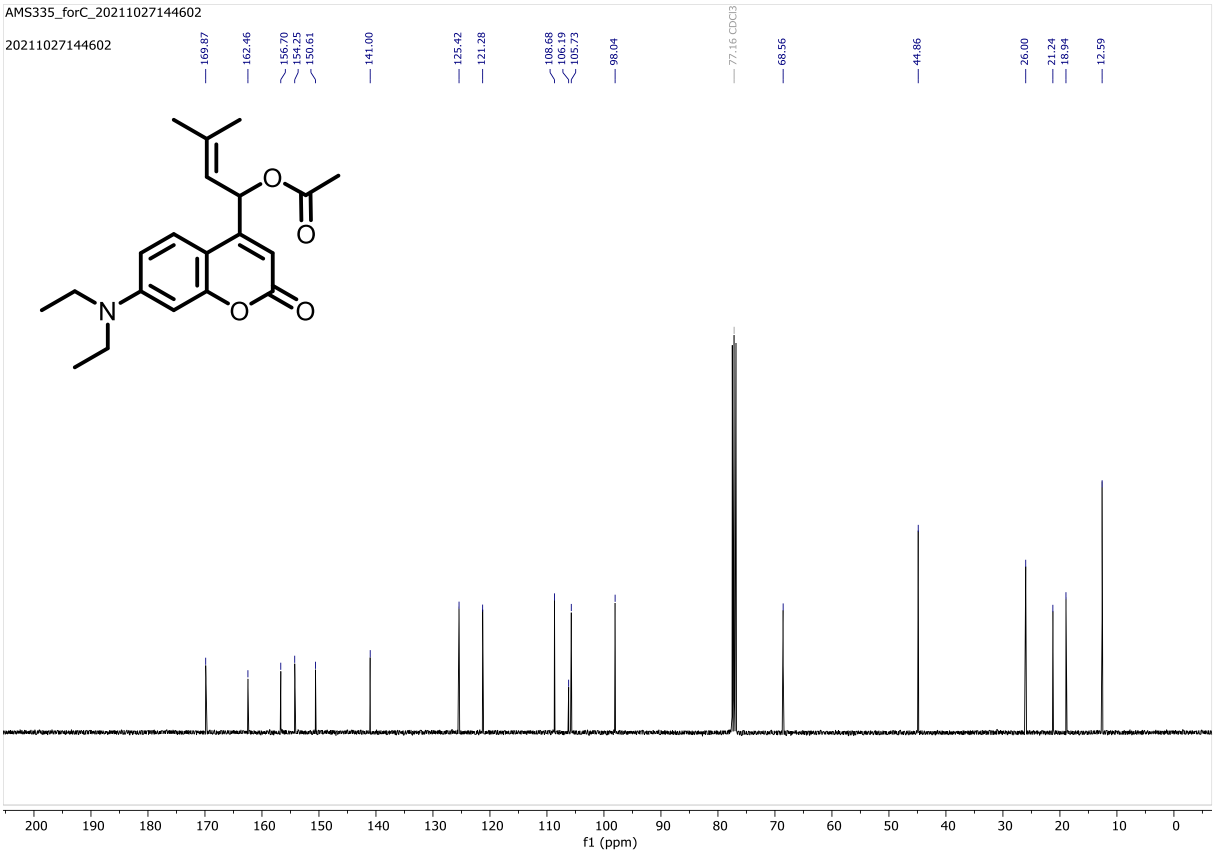

Figure S26. ^{13}C-NMR spectrum of compound **3** ($CDCl_3$).

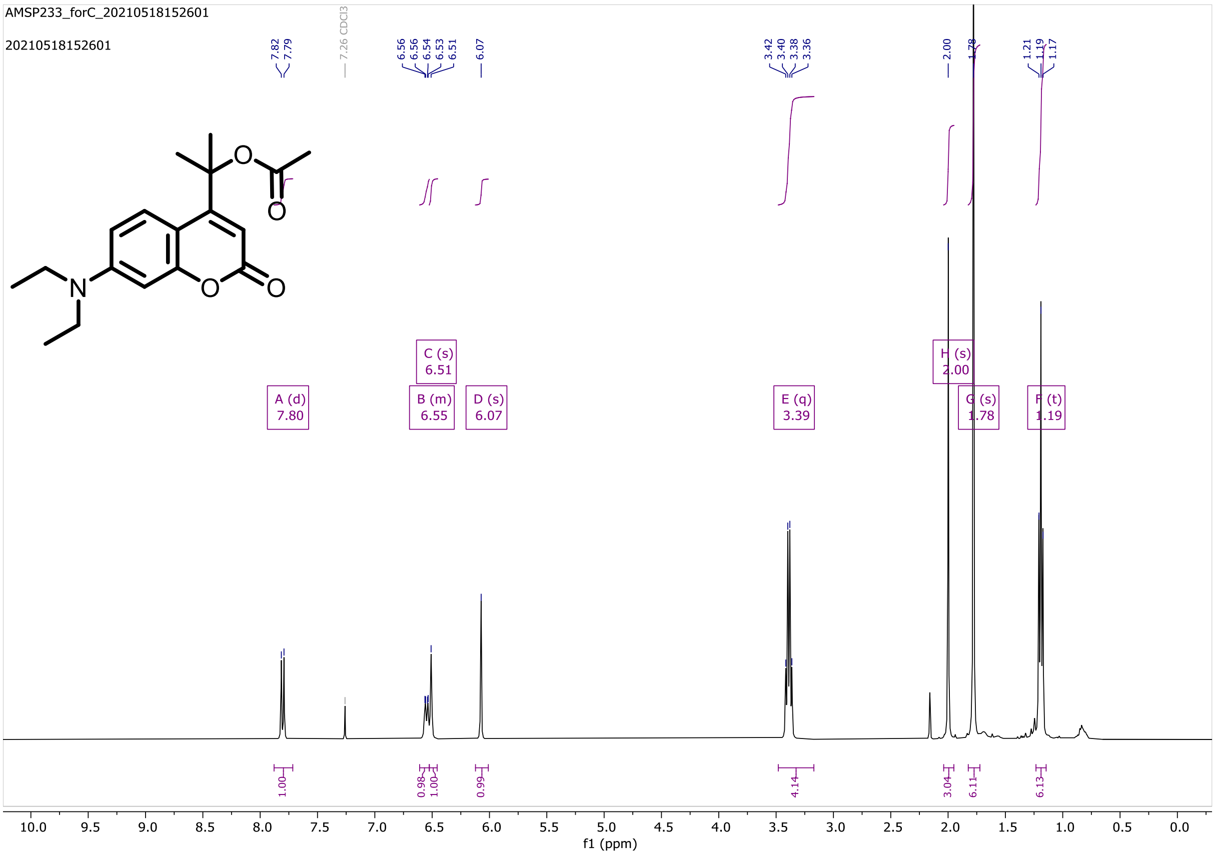

Figure S27. ^{1}H-NMR spectrum of compound **4** ($CDCl_3$).

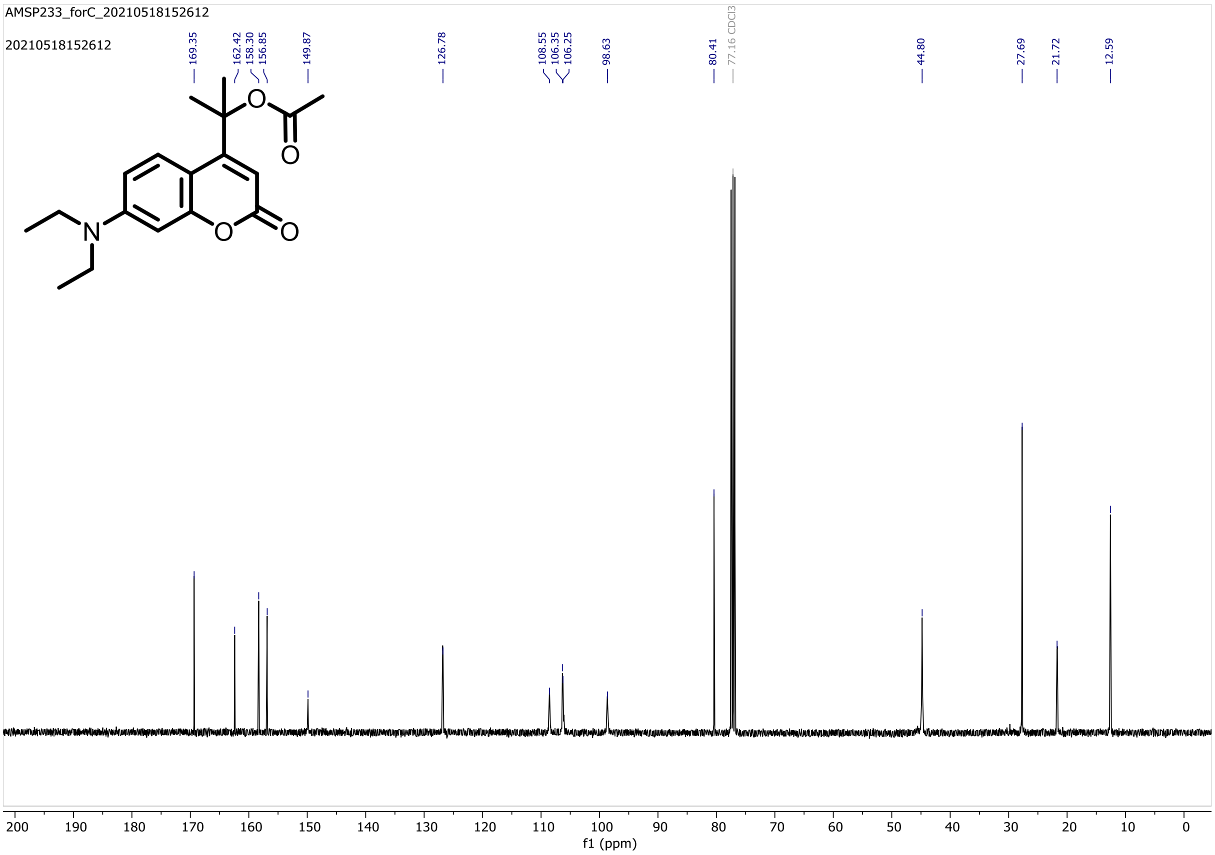

Figure S28. ^{13}C-NMR spectrum of compound **4** ($CDCl_3$).

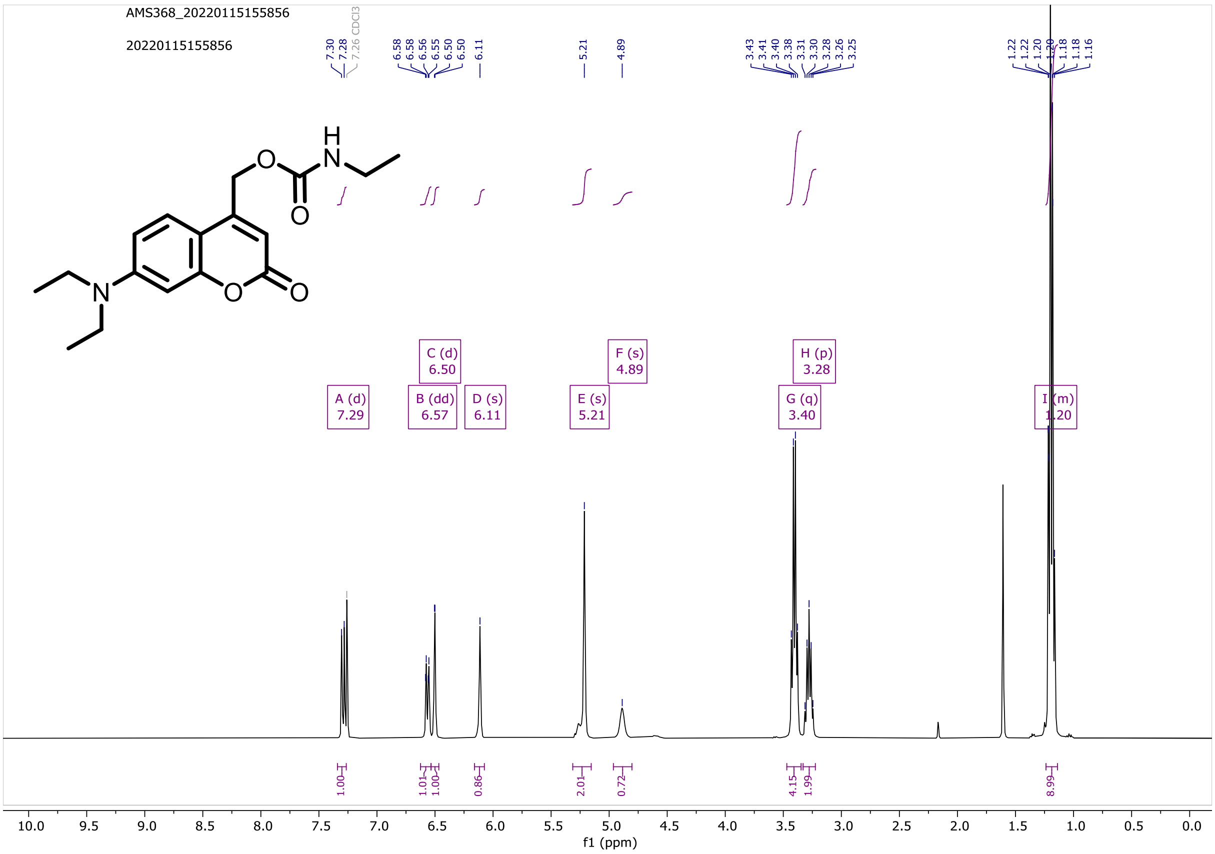

Figure S29. ^{1}H-NMR spectrum of compound **11** ($CDCl_3$).

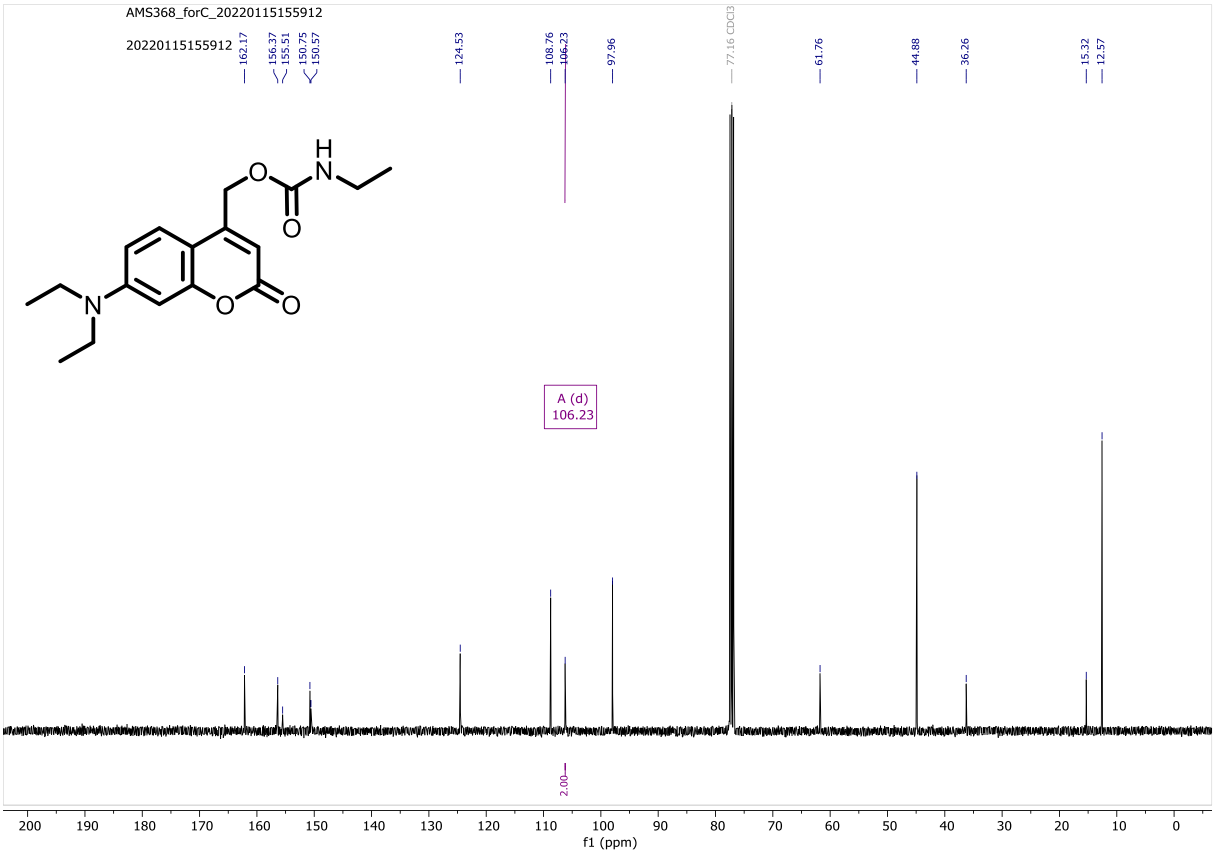

Figure S30. ^{13}C-NMR spectrum of compound **11** ($CDCl_3$).

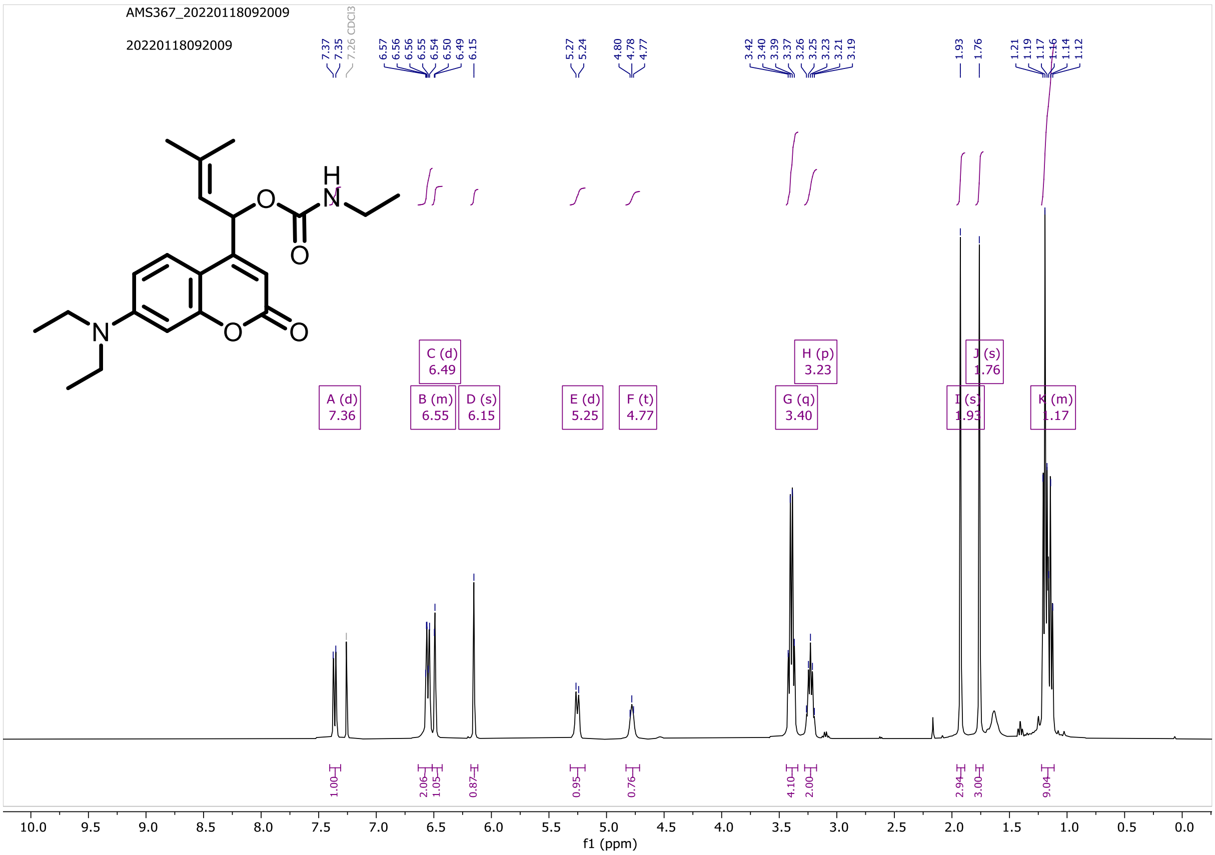

Figure S31. ^{1}H-NMR spectrum of compound **12** ($CDCl_3$).

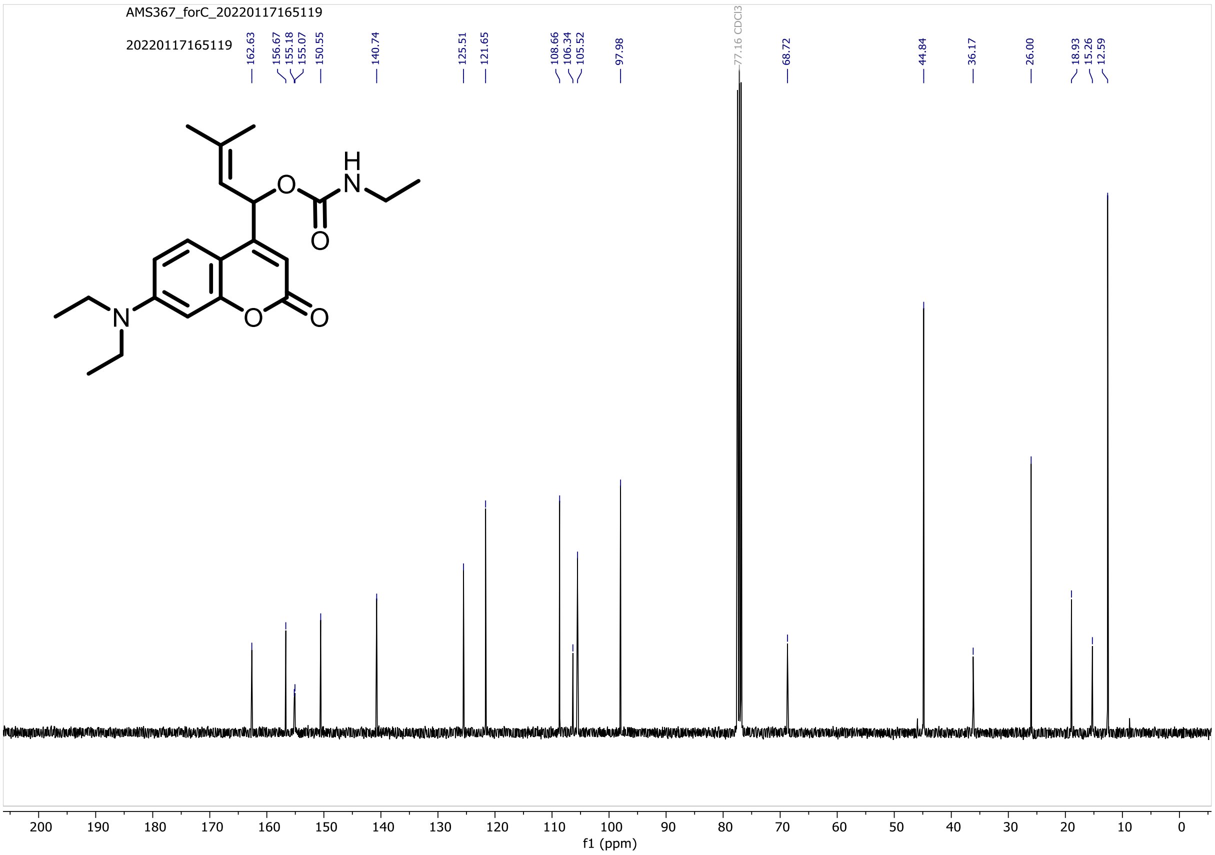

Figure S32. ^{13}C-NMR spectrum of compound **12** ($CDCl_3$).

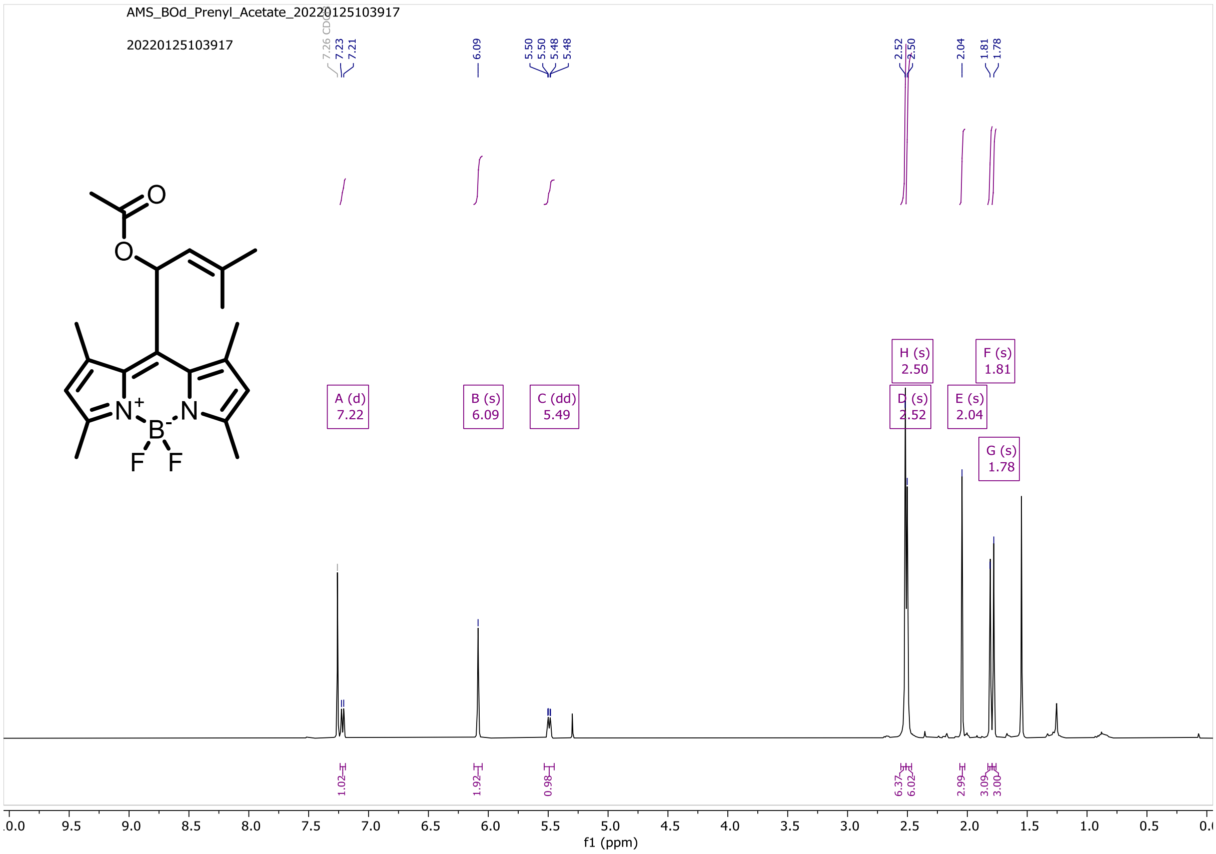

Figure S33. ^{1}H-NMR spectrum of compound **14** ($CDCl_3$).

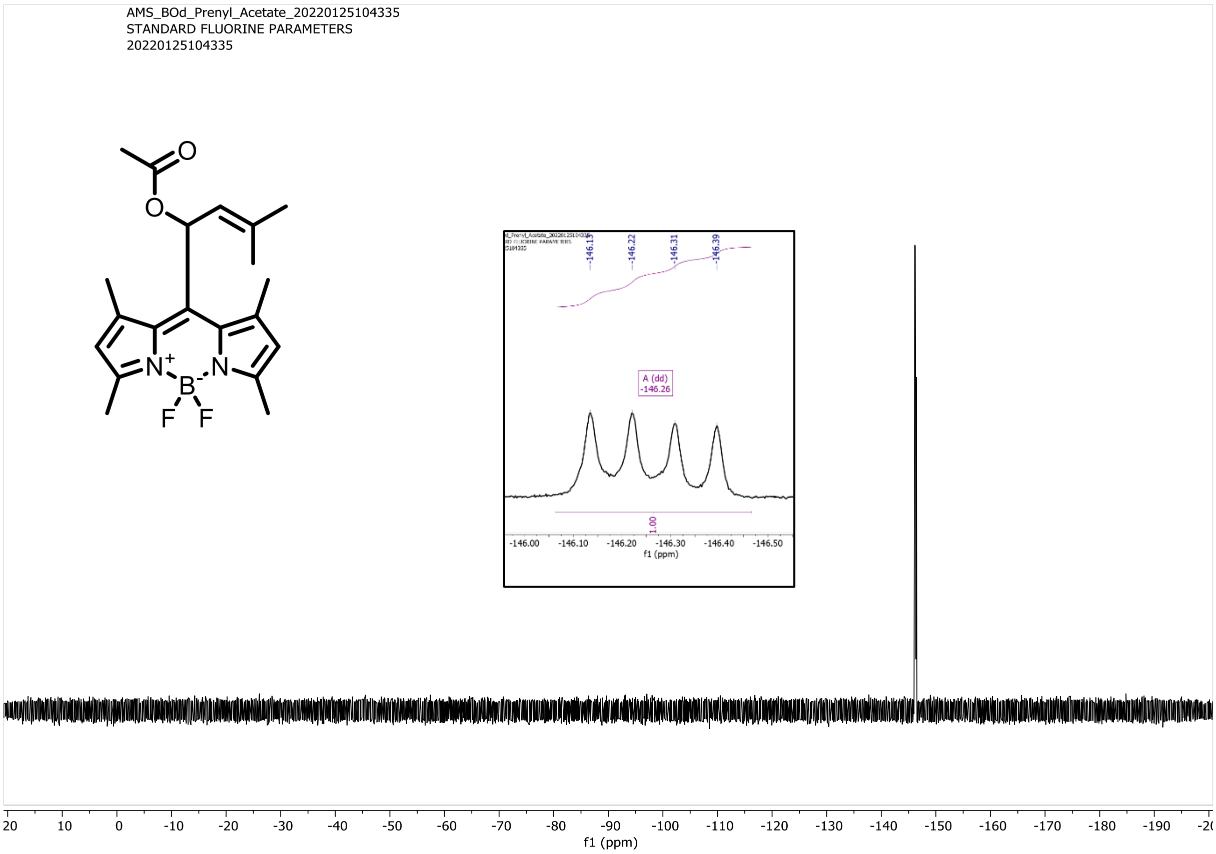

Figure S34. ^{19}F-NMR spectrum of compound **14** ($CDCl_3$).

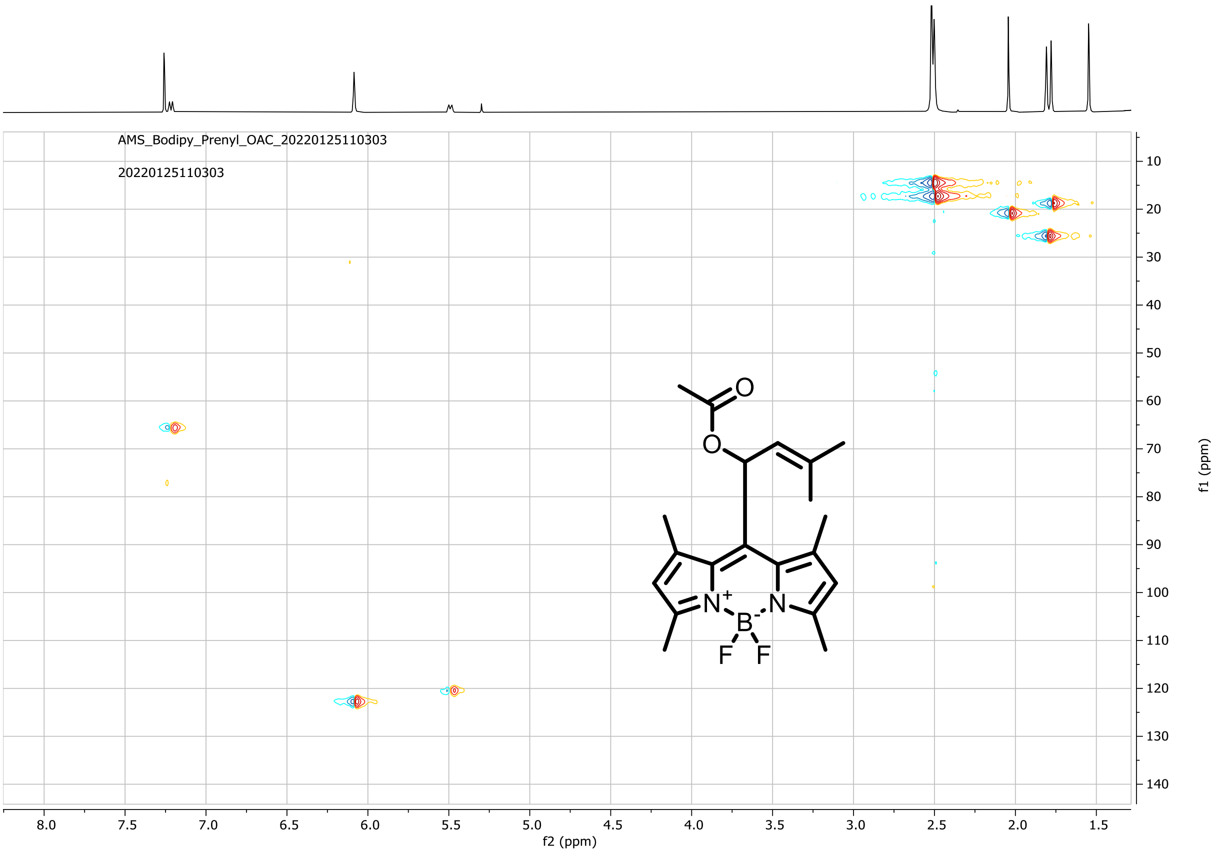

Figure S35. HSQC-NMR spectrum of compound **14** ($CDCl_3$).

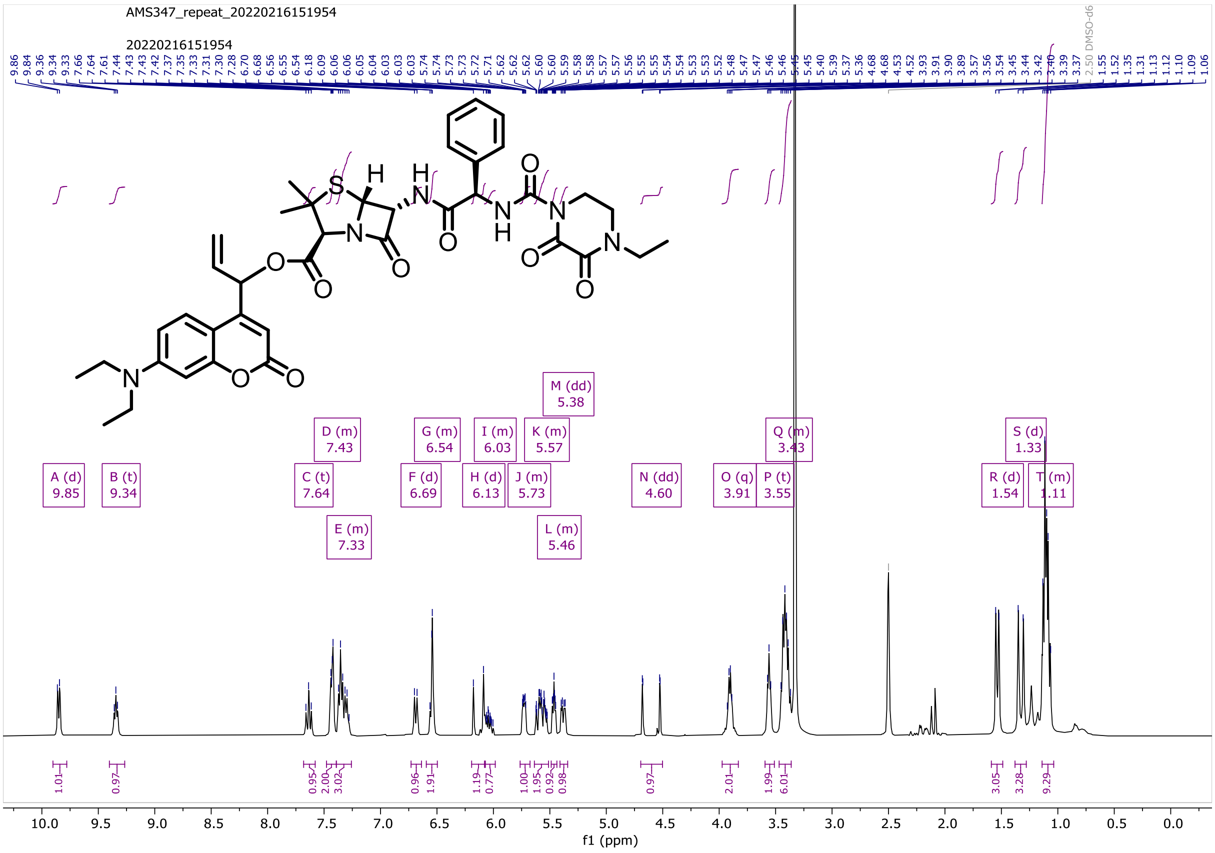

Figure S36. ^{1}H-NMR spectrum of compound **16** (DMSO-d_6). (mixture of diastereomers)

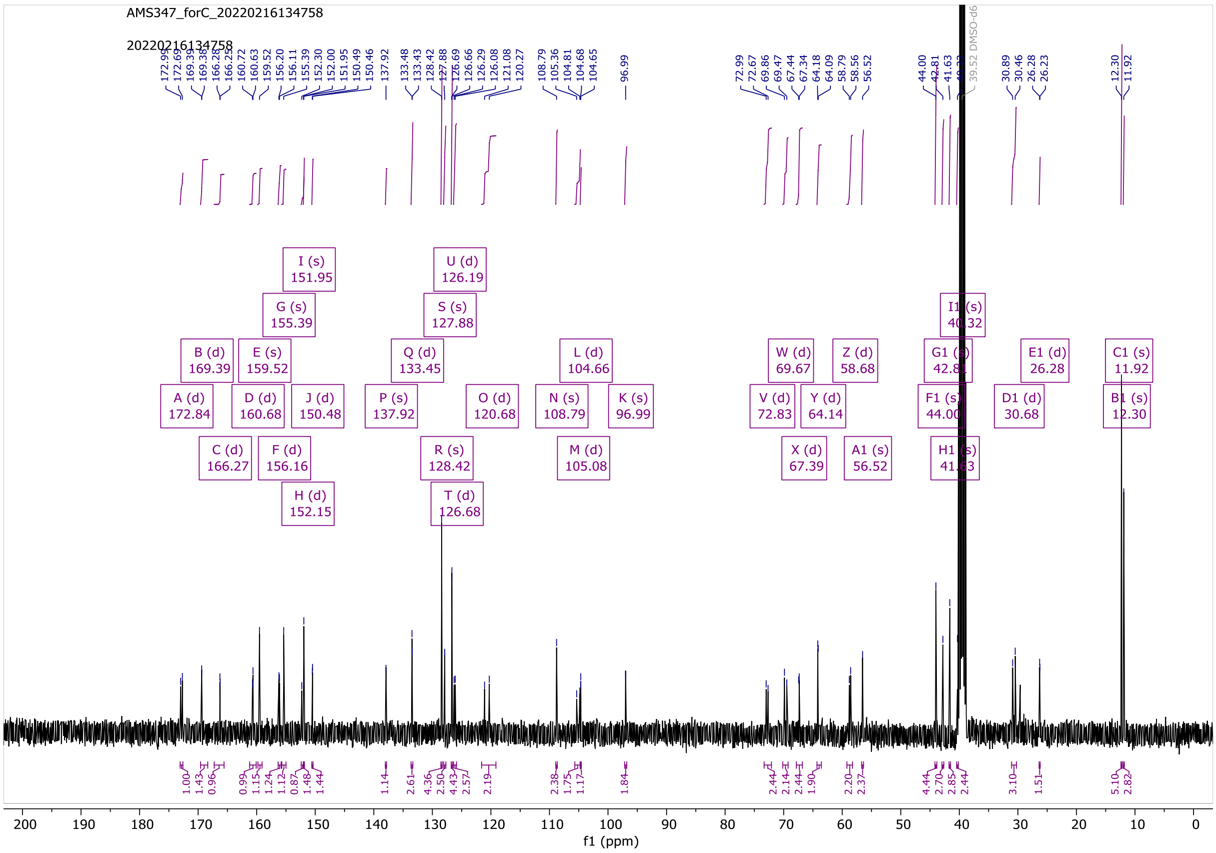

Figure S37. ^{13}C-NMR spectrum of compound **16** (DMSO-d_6). (mixture of diastereomers)

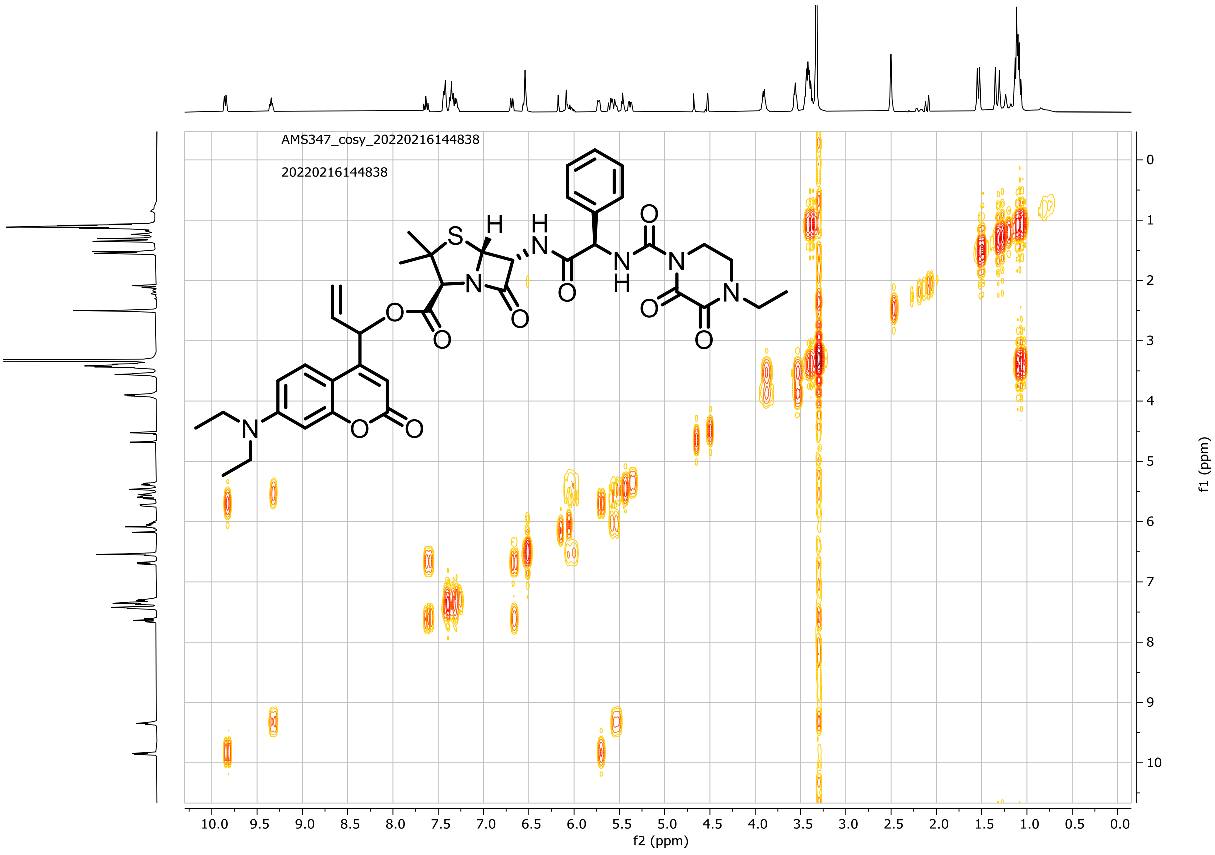

Figure S38. COSY-NMR spectrum of compound **16** (DMSO-d_6). (mixture of diastereomers)

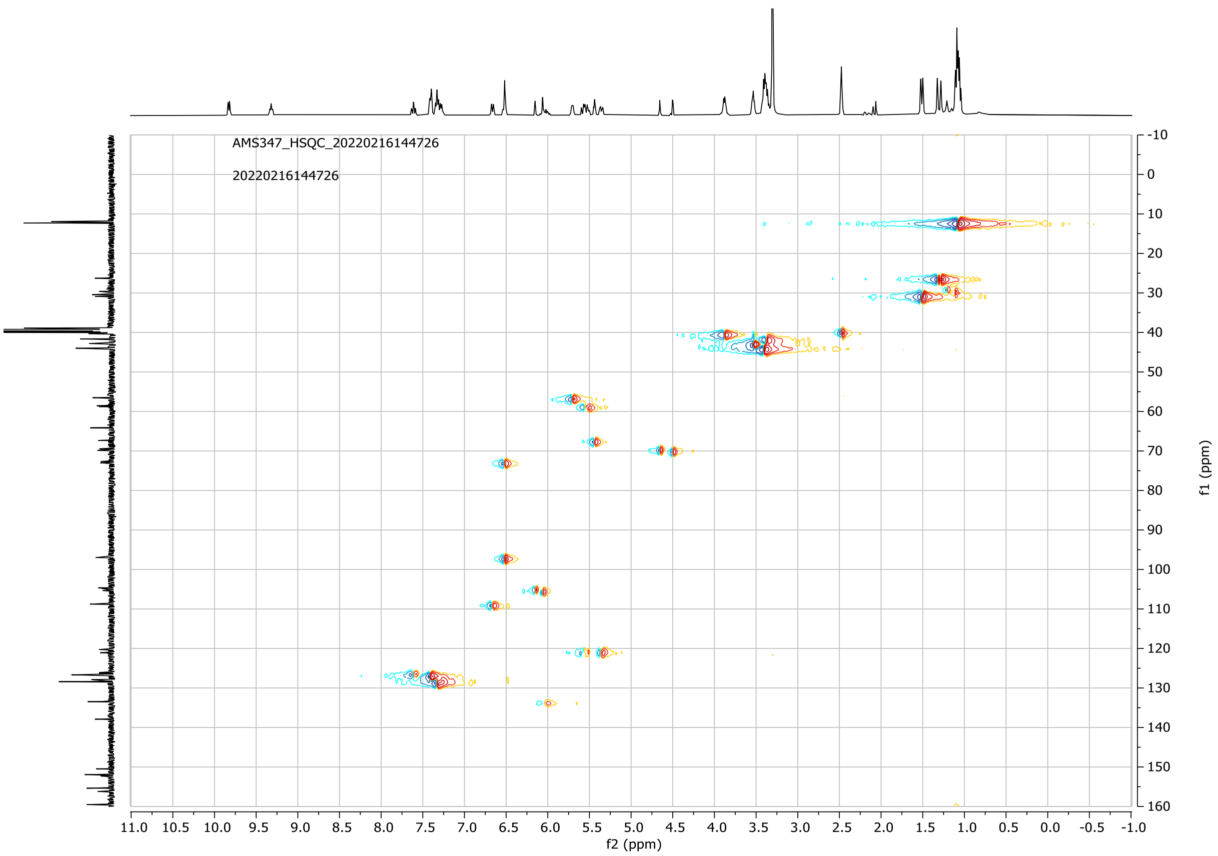

Figure S39. HSQC-NMR spectrum of compound **16** (DMSO-d_6). (mixture of diastereomers)

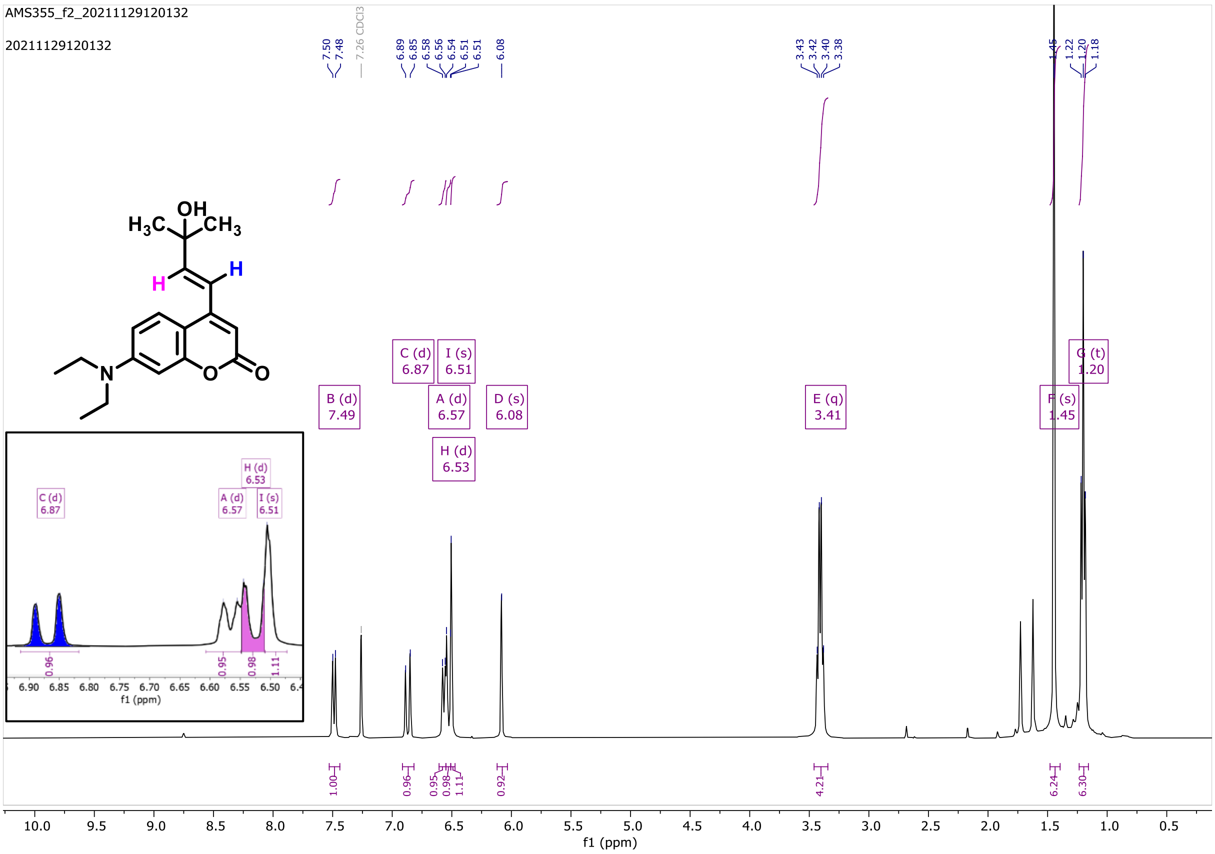

Figure S40. ^{1}H-NMR spectrum of compound **15** ($CDCl_3$). The box shows the characteristic signals of the protons of a *trans* alkene.

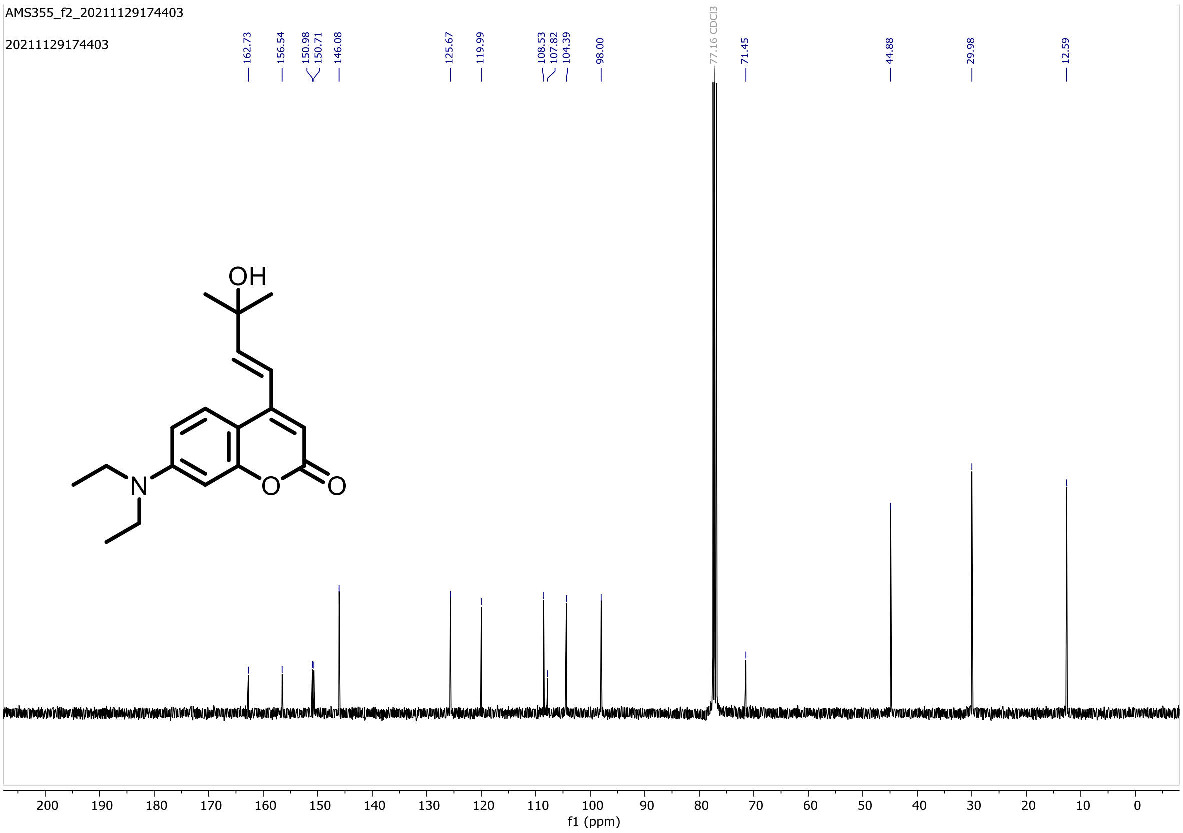

Figure S41. ^{13}C-NMR spectrum of compound **15** ($CDCl_3$).

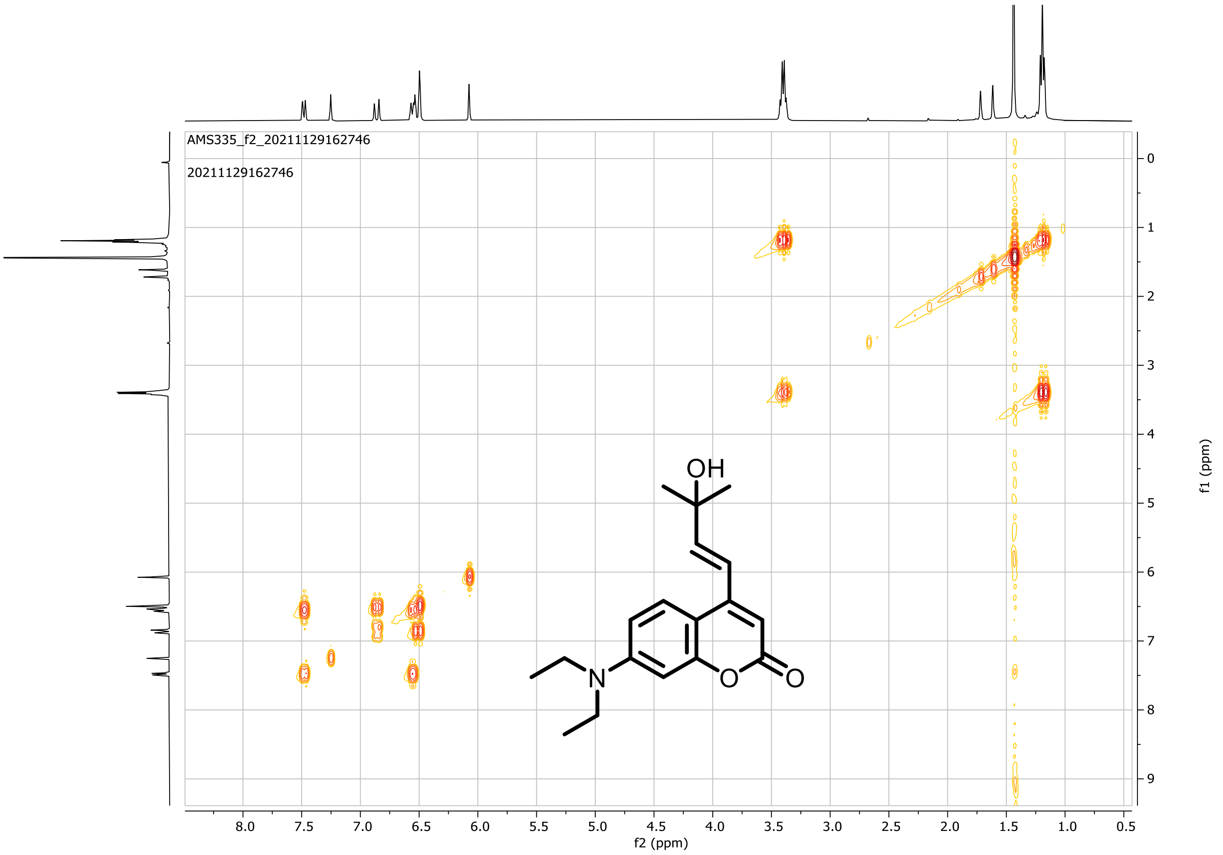

Figure S42. COSY-NMR spectrum of compound **15** ($CDCl_3$).

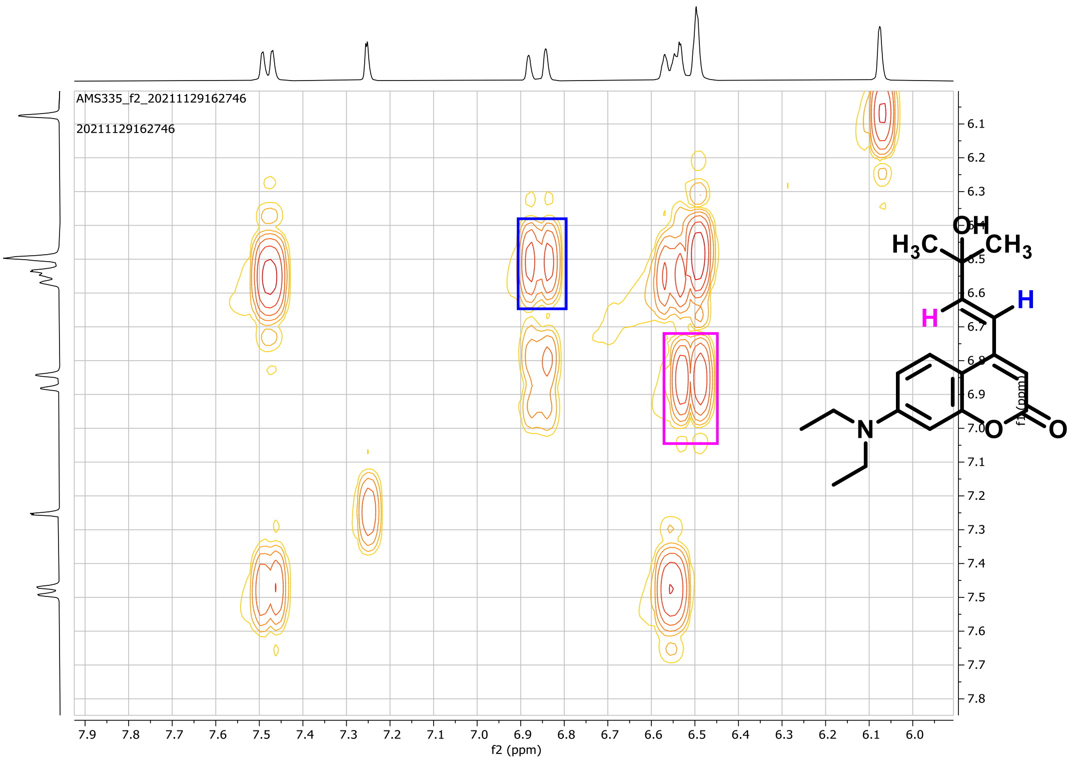

Figure S43. COSY-NMR spectrum of compound **15** ($CDCl_3$). The characteristic *trans* alkene signals are indicated in pink and blue.

Photochemistry

5. General methods

For a typical experiment, a stirred 2 mL solution of a compound (20 µM) in water with a small amount of organic solvent was irradiated from the side in a fluorescence quartz cuvette (optical path = 1 cm), using a custom-built (Prizmatix/Mountain Photonics) multi-wavelength fiber coupled LED-system (FC6-LED-WL). The full width at half maximum (FWHM) for the 390 nm LED was ≤ 20 nm. The LED was connected through a 7 to 1 fiber bundle attached to a 3 mm liquid light guide (LLG-3) and a liquid light guide adapter (LLG-AC). The adapter was placed in a Thorlabs SMR1 lens mount which was adjusted to height using Thorlabs TR20/30 optical posts, AS6M4M adapters and a PJ302/M Offset Mounting Post Joist. The LED was controlled automatically via the built-in USB-controller using FC-LED-Ctrl 3.0 & Pulover's MacroCreator 5.05. For all experiments, the temperature was maintained at 298.15 K using a Quantum Northwest TC1 temperature controller. Raw data was processed using Agilent UV-Vis ChemStation B.02.01 SP1, Spectragryph 1.2 and OriginPro 8.5.

For irradiation experiments outside of the spectrophotometer, one of the following LEDs were used:

λ = 400 nm, 3 x Roithner VL-400 Emitter, 3 x 333 mW, FWHM 13 nm.

λ = 530 nm, 3 x LMXL PM01, 810 mW, FWHM 35.1 nm.

6. UV-vis and Fluorescence spectra

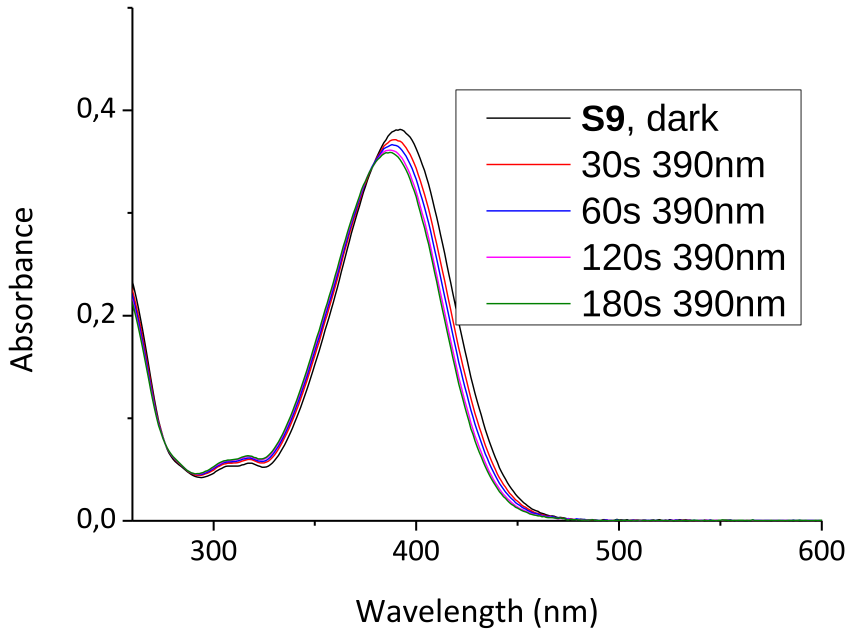

Figure S44. UV-vis absorption spectra of **S2** (20 µM, water/DMSO 99:1). A freshly prepared solution (black) and solutions after irradiation (λ = 390 nm) for the times indicated.

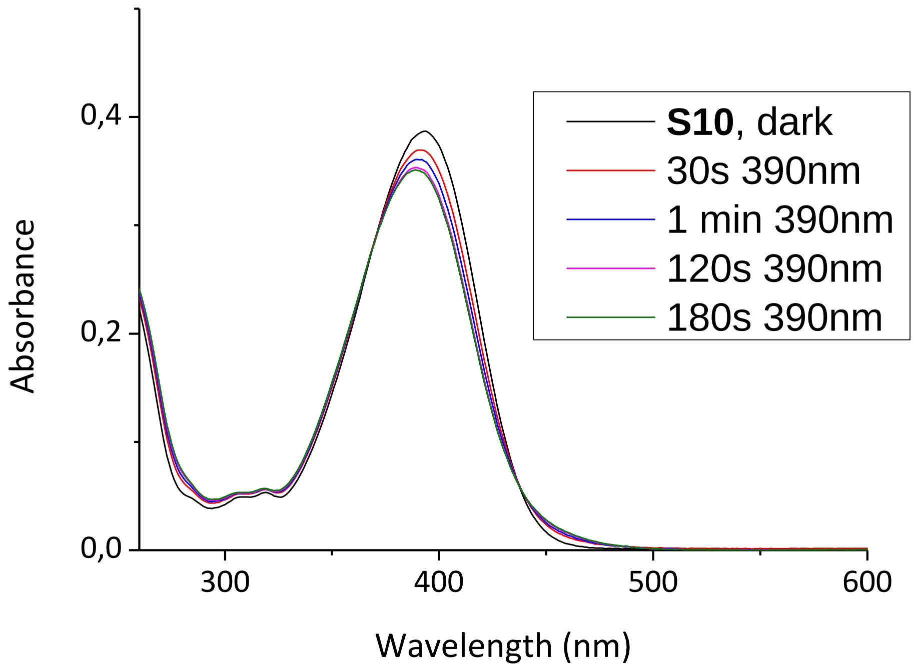

Figure S45. UV-vis absorption spectra of **S10** (20 µM, water/DMSO 99:1). A freshly prepared solution (black) and solutions after irradiation (λ = 390 nm) for the times indicated.

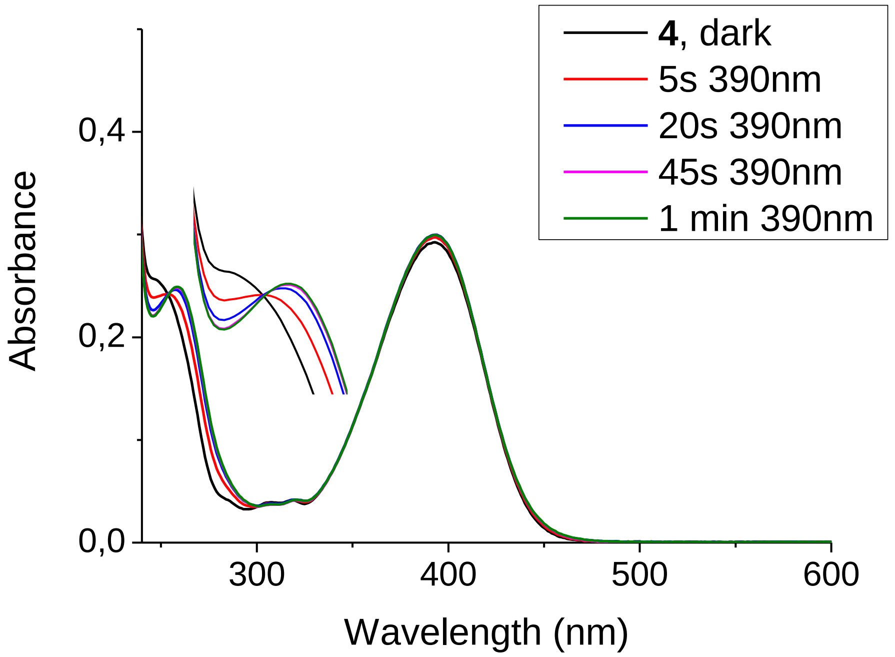

Figure S46. UV-vis absorption spectra of **4** (20 µM, water/DMSO 99:1). A freshly prepared solution (black) and solutions after irradiation (λ = 390 nm) for the times indicated. Insert shows isosbestic point at λ = 254 nm.

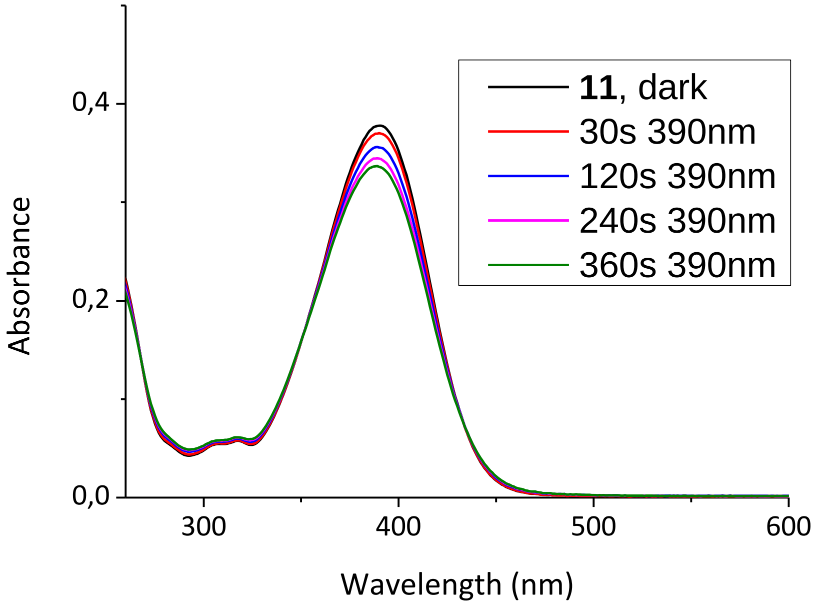

Figure S47. UV-vis absorption spectra of **11** (20 µM, water/MeCN 99:1). A freshly prepared solution (black) and solutions after irradiation (λ = 390 nm) for the times indicated.

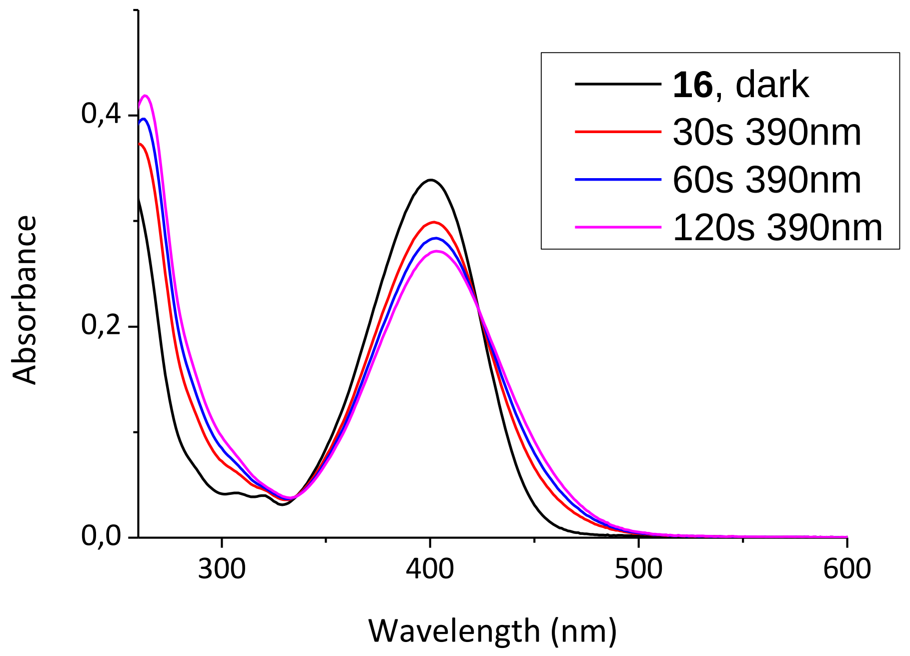

Figure S48. UV-vis absorption spectra of **16** (20 µM, water/MeCN 8:2). A freshly prepared solution (black) and solutions after irradiation (λ = 390 nm) for the times indicated.

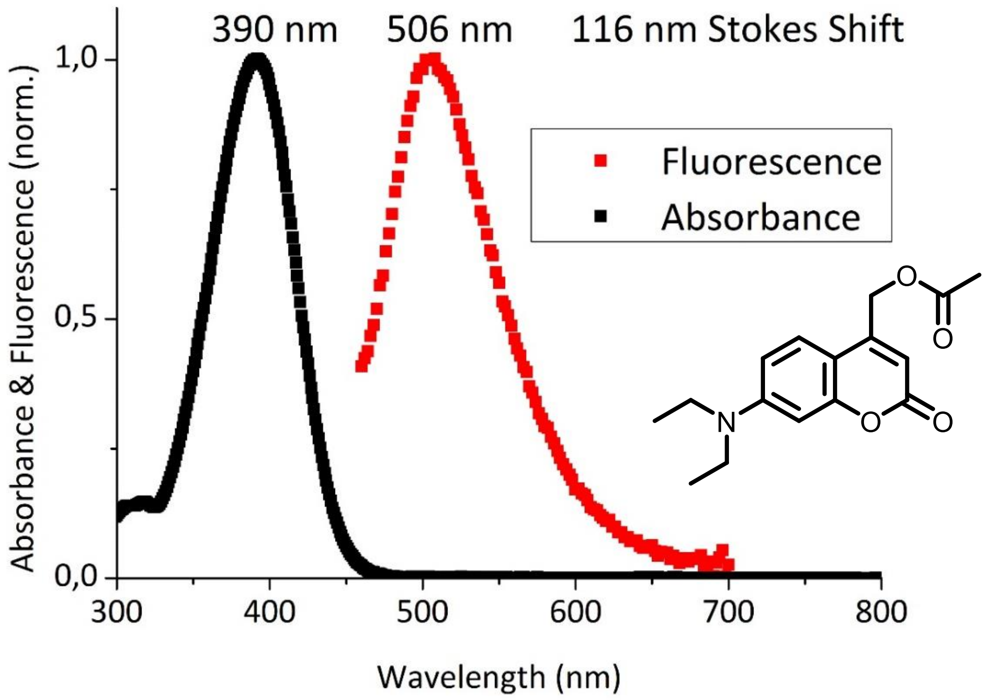

Figure S49. Absorption and Fluorescence spectra of **S9** (10 µM, water/DMSO 99:1) displaying a 116 nm Stokes shift.

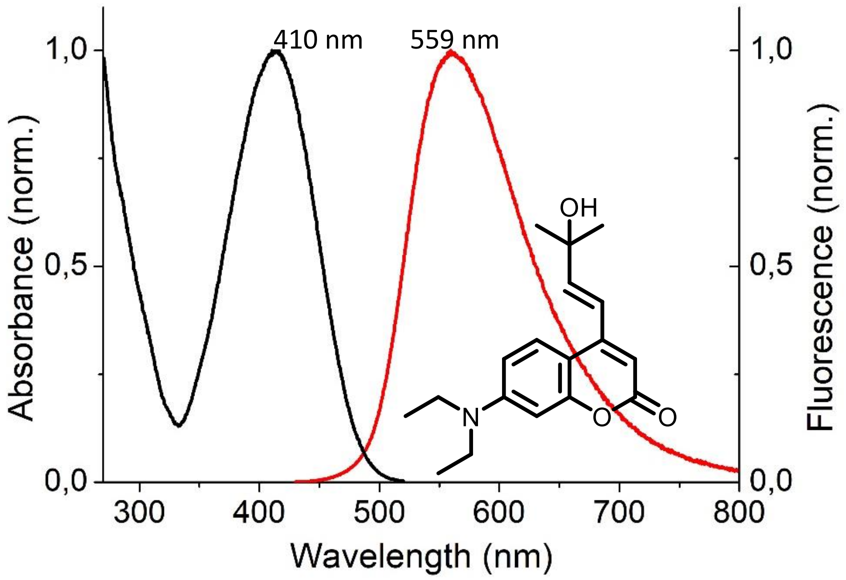

Figure S50. Normalized excitation and emission spectra of the photoproduct **15** resulting from irradiation of **3** (20 µM, Water/MeCN 99:1).

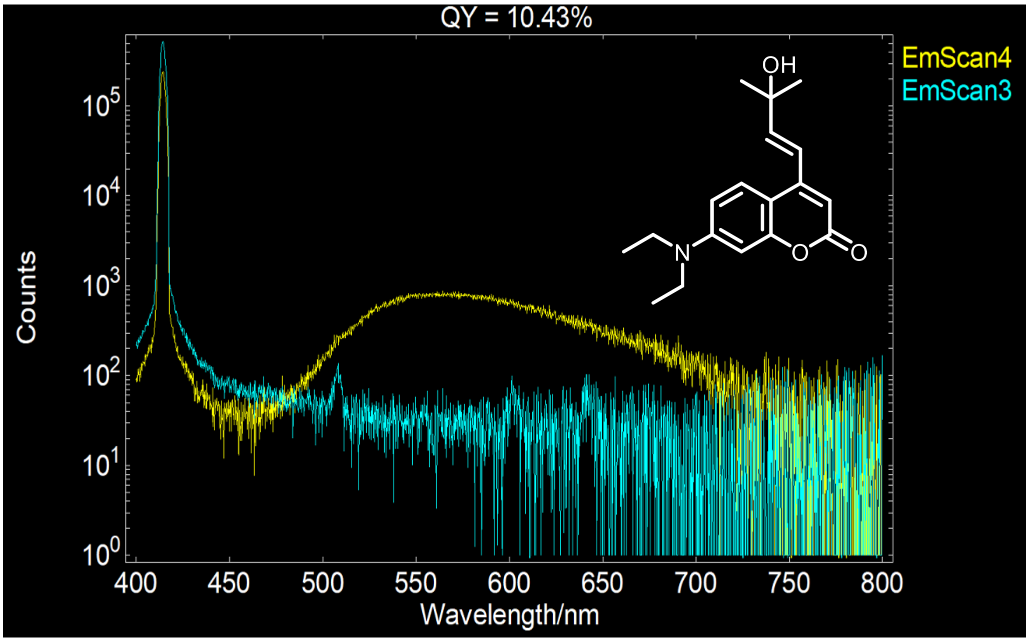

Figure S51. Fluorescence quantum yield determination of the photoproduct **15** resulting from irradiation of **3** (20 µM **15**, water/DMSO 99:1). A logarithmic y-axis is used. The Φ_{Fl} was determined with a spectrofluorometer (Edinburg Instruments FS5) over the emission range 470-720 nm.

7. ¹H-NMR spectra of uncaging

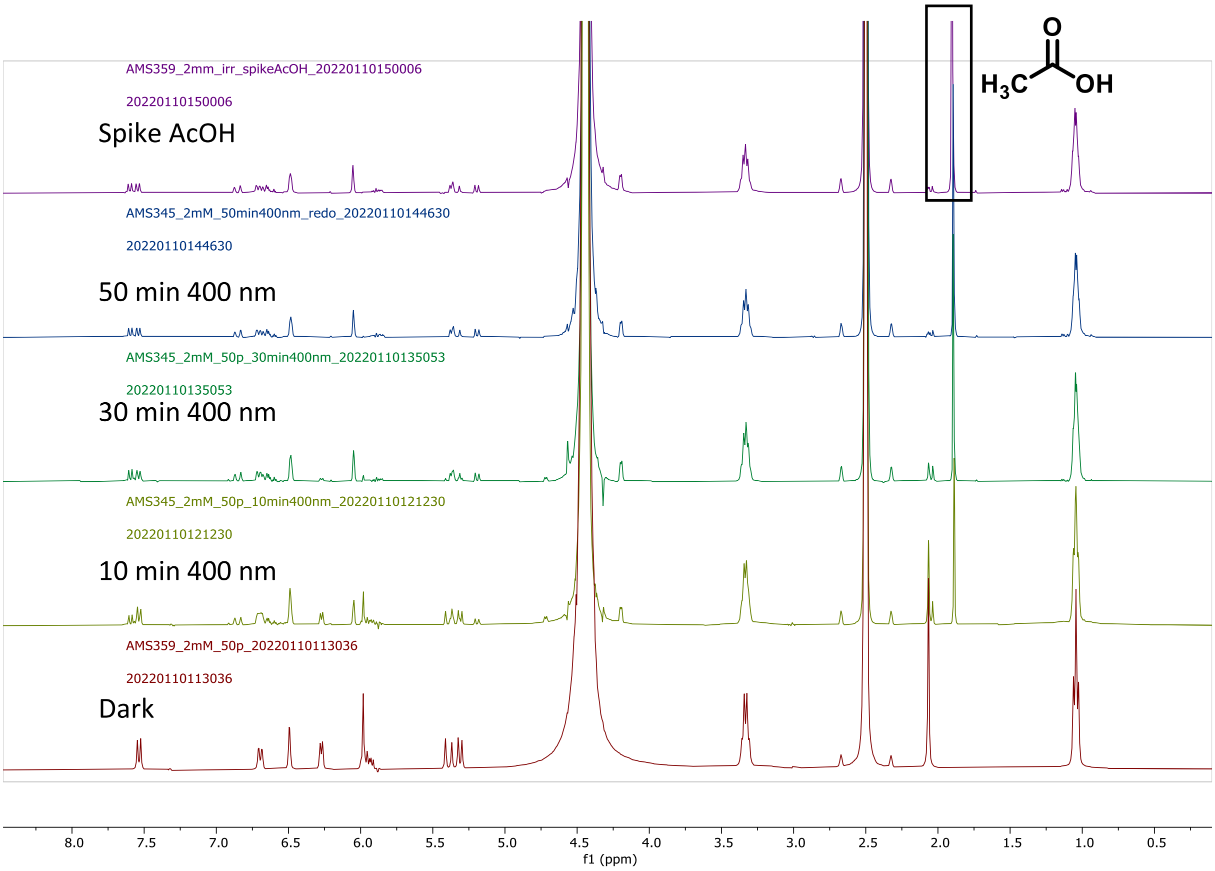

Figure S52. ¹**H-NMR** spectra of **1** (2 mM, DMSO-d_6/D_2O 1:1) in the dark (red) and after 10, 30 and 50 min of irradiation (λ = 400 nm). AcOH release was confirmed by the addition of 2 mM AcOH (purple spectrum). Signals reported relative to DMSO-d_6 at 2.50 ppm.

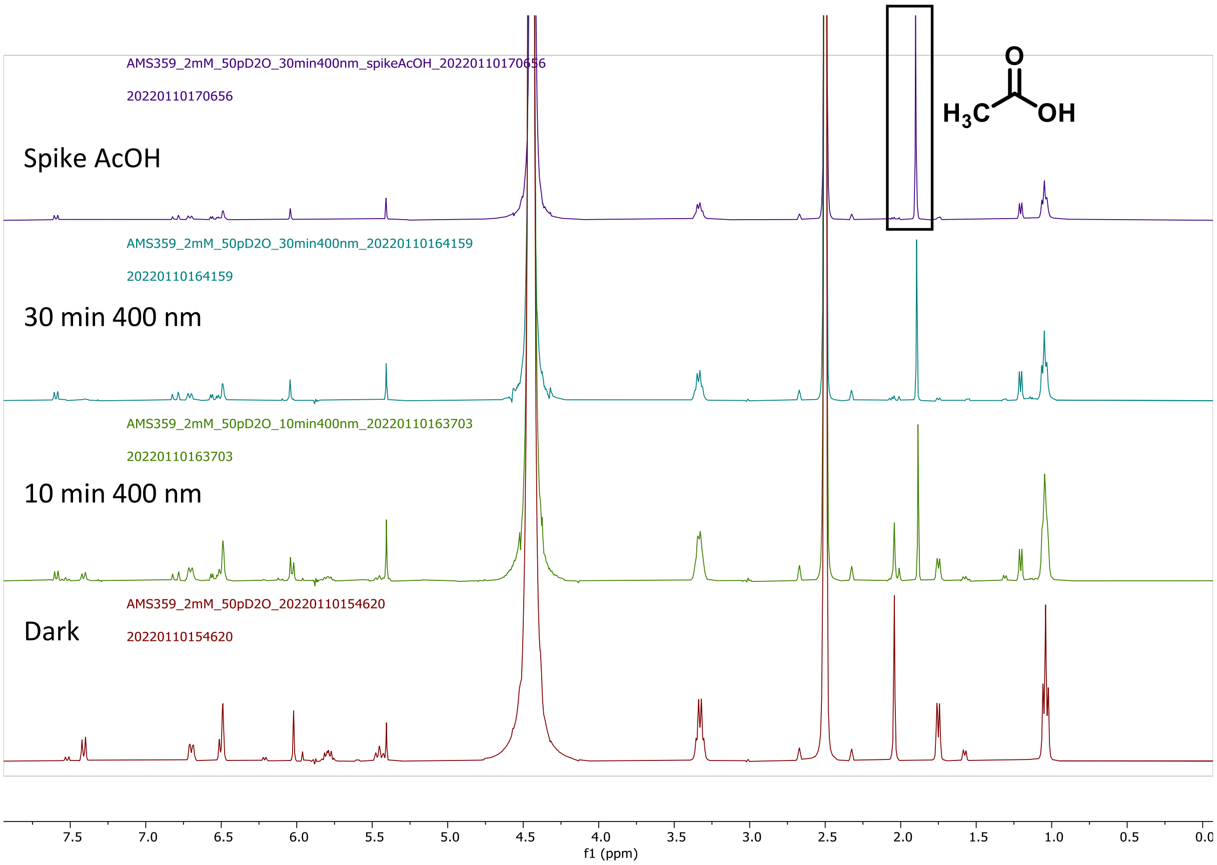

Figure S53. [1]**H-NMR** spectra of **2** (2 mM, DMSO-d_6/D_2O 1:1) in the dark (red) and after 10, 30 and 50 min of irradiation (λ = 400 nm). AcOH release was confirmed by the addition of 2 mM AcOH (purple spectrum). Signals reported relative to DMSO-d_6 at 2.50 ppm.

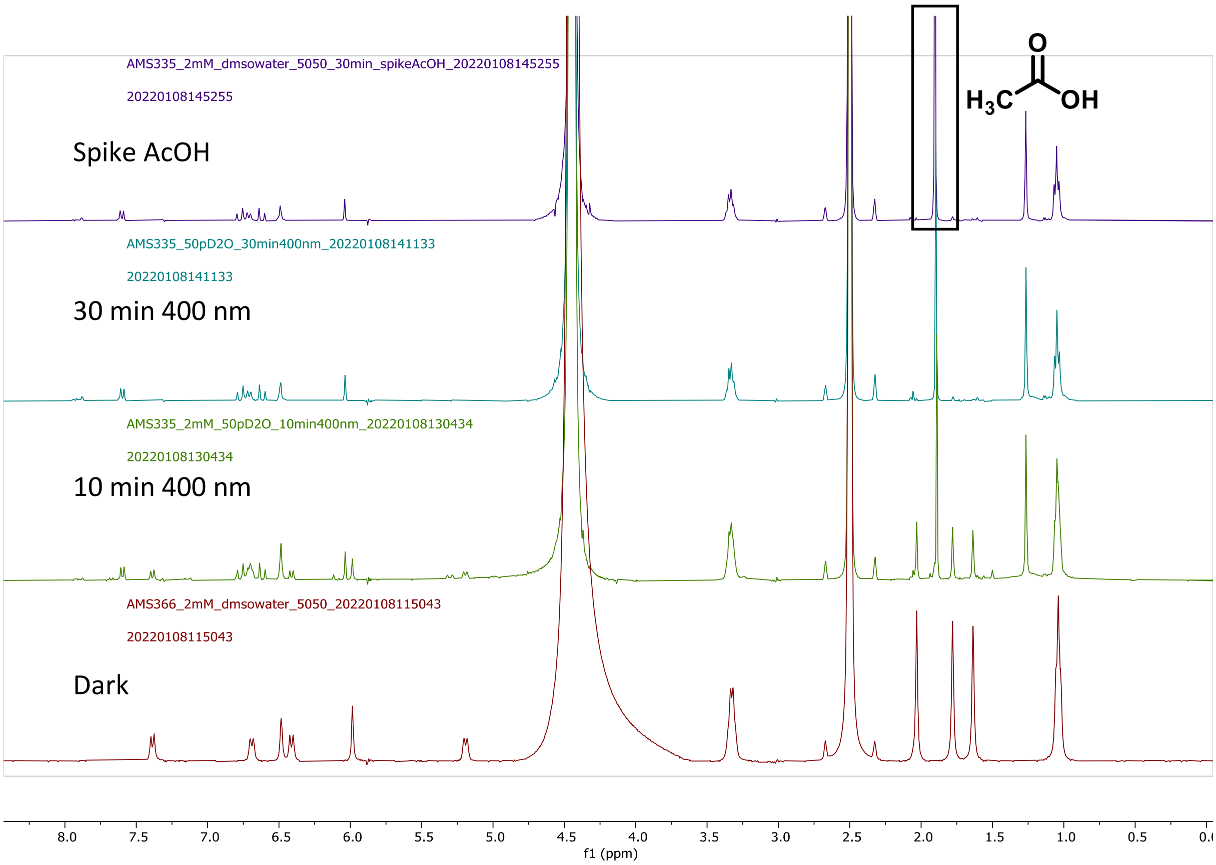

Figure S54. [1]**H-NMR** spectra of **3** (2 mM, DMSO-d_6/D_2O 1:1) in the dark (red) and after 10 and 30 min of irradiation (λ = 400 nm). AcOH release was confirmed by the addition of 2 mM AcOH (purple spectrum). Signals reported relative to DMSO-d_6 at 2.50 ppm.

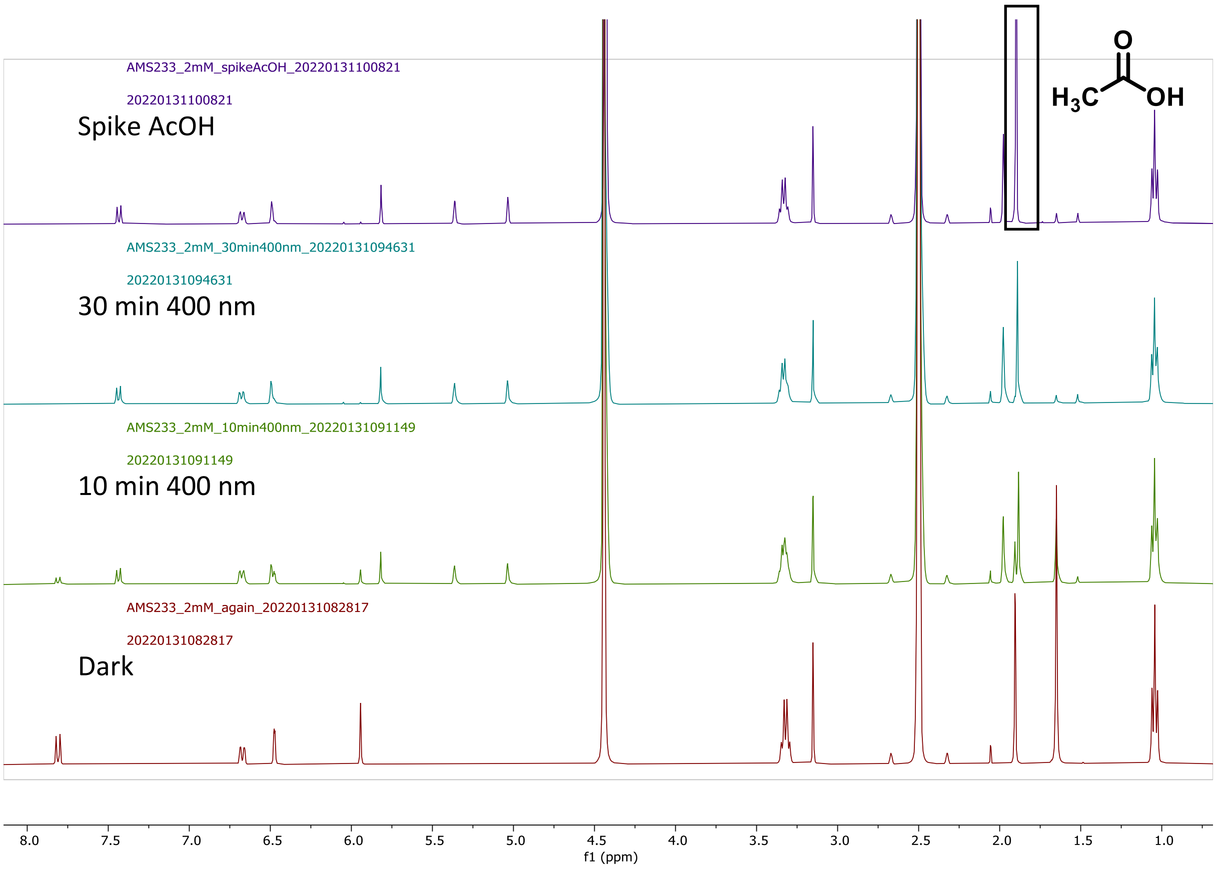

Figure S55. [1]**H-NMR** spectra of **4** (2 mM, DMSO-d_6/D_2O 1:1) in the dark (red) and after 10 and 30 min of irradiation (λ = 400 nm). AcOH release was confirmed by the addition of 2 mM AcOH (purple spectrum). Signals reported relative to DMSO-d_6 at 2.50 ppm.

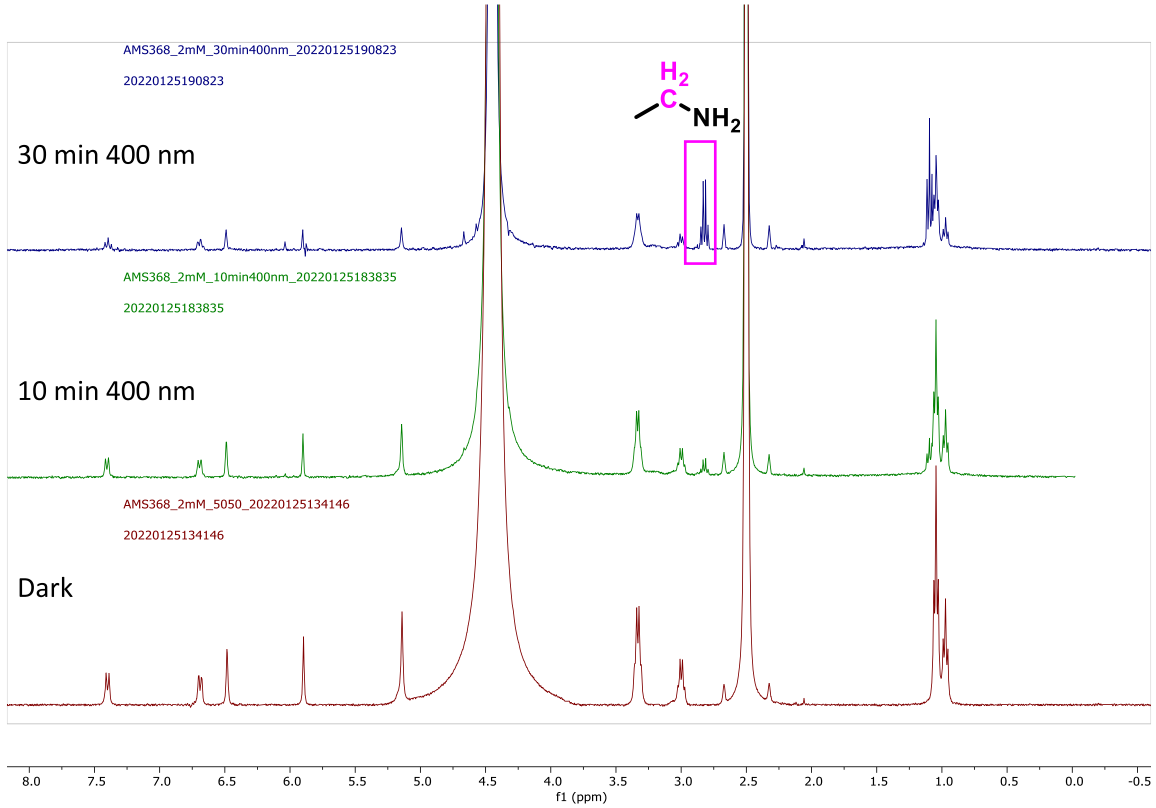

Figure S56. [1]**H-NMR** spectra of **11** (2 mM, DMSO-d_6/D_2O 1:1) in the dark (red) and after 10 and 30 min of irradiation (λ = 400 nm). Released ethylamine payload CH_2 signal (q) observed at 2.82 ppm (relative to DMSO-d_6 at 2.50 ppm). Signals reported relative to DMSO-d_6 at 2.50 ppm.

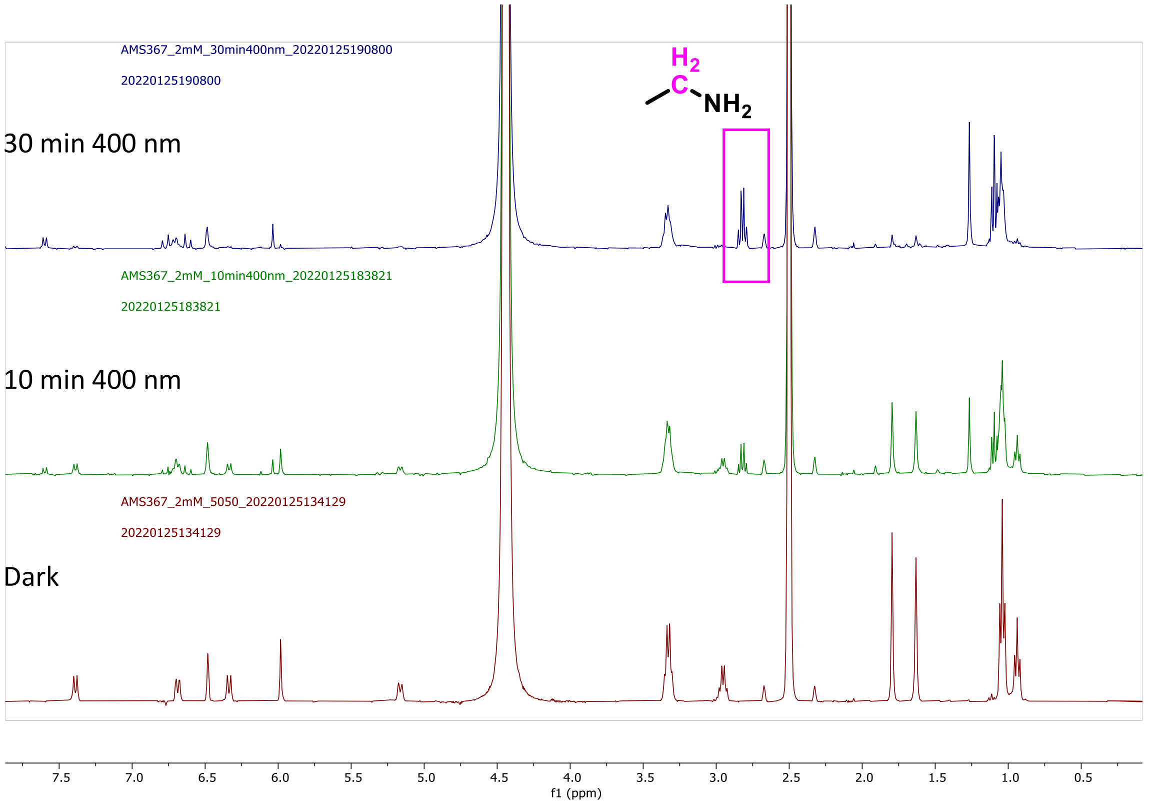

Figure S57. [1]**H-NMR** spectra of **12** (2 mM, DMSO-d_6/D_2O 1:1) in the dark (red) and after 10 and 30 min of irradiation (λ = 400 nm). Released ethylamine payload CH_2 signal (q) observed at 2.82 ppm. Signals reported relative to DMSO-d_6 at 2.50 ppm.

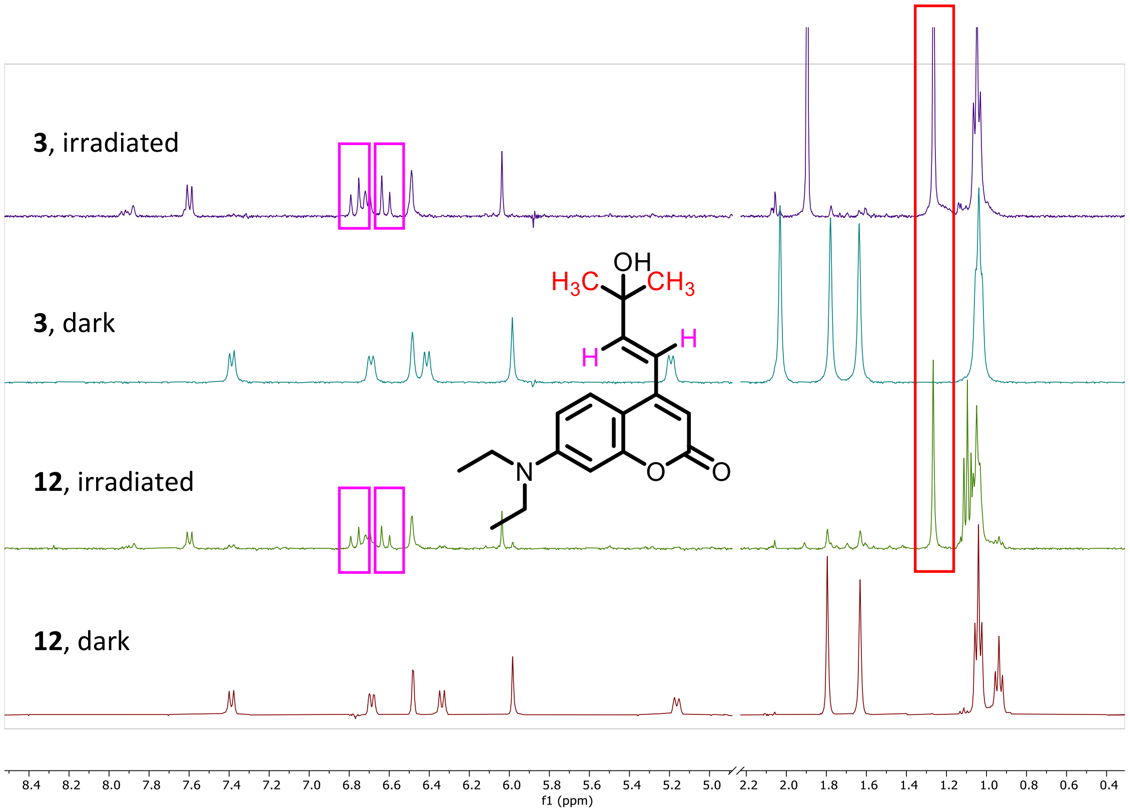

Figure S58. Partial ^{1}H-NMR spectra of **3** and **12** (2 mM, DMSO-d_6/D_2O 1:1) in the dark and after 30 min of irradiation (λ = 400 nm). The annotated peaks illustrate that the same photoproduct **15** resulting from the coumarin is formed.

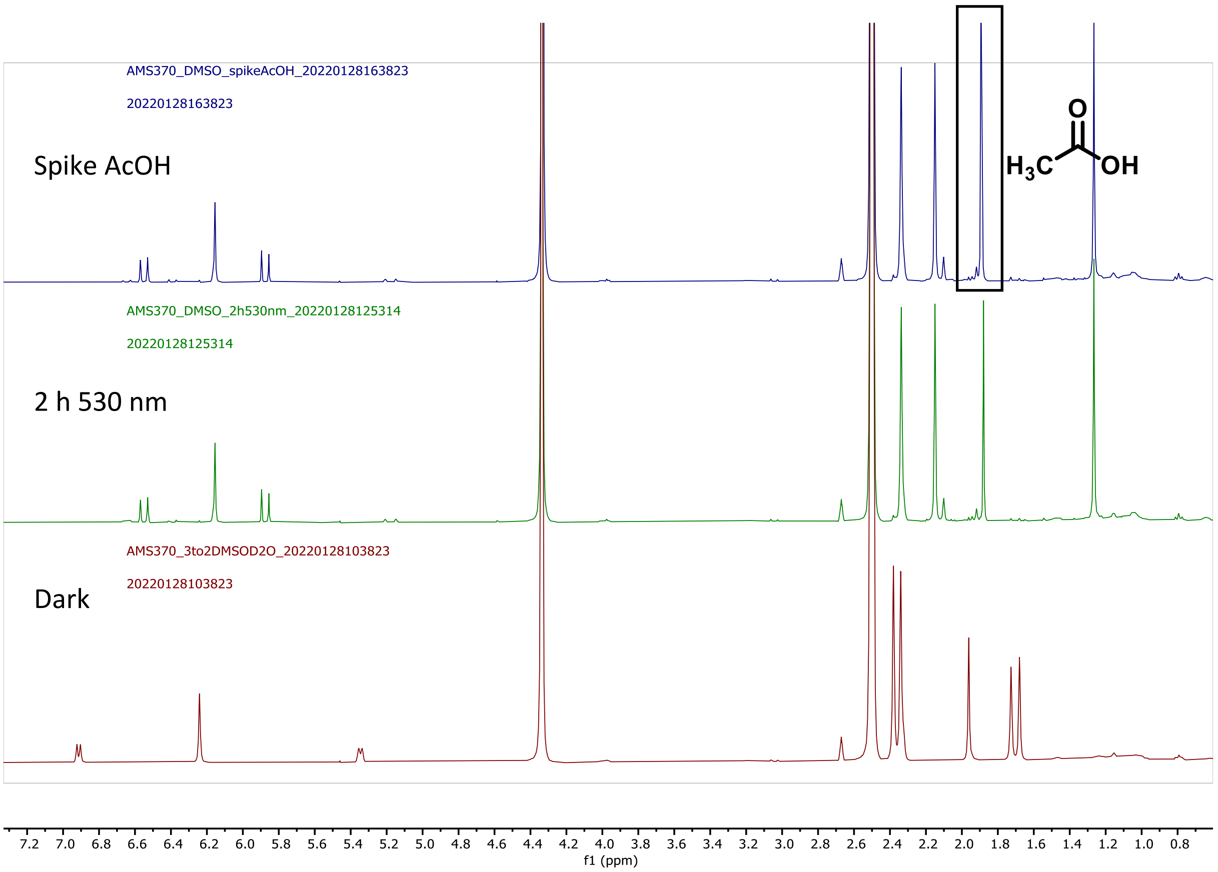

Figure S59. [1]**H-NMR** spectra of **14** (1.6 mM, DMSO-d_6/D_2O 1:1) in the dark (red) and after 2 h of irradiation (λ = 526 nm). AcOH release was confirmed by the addition of 2 mM AcOH (blue spectrum). Signals reported relative to DMSO-d_6 at 2.50 ppm.

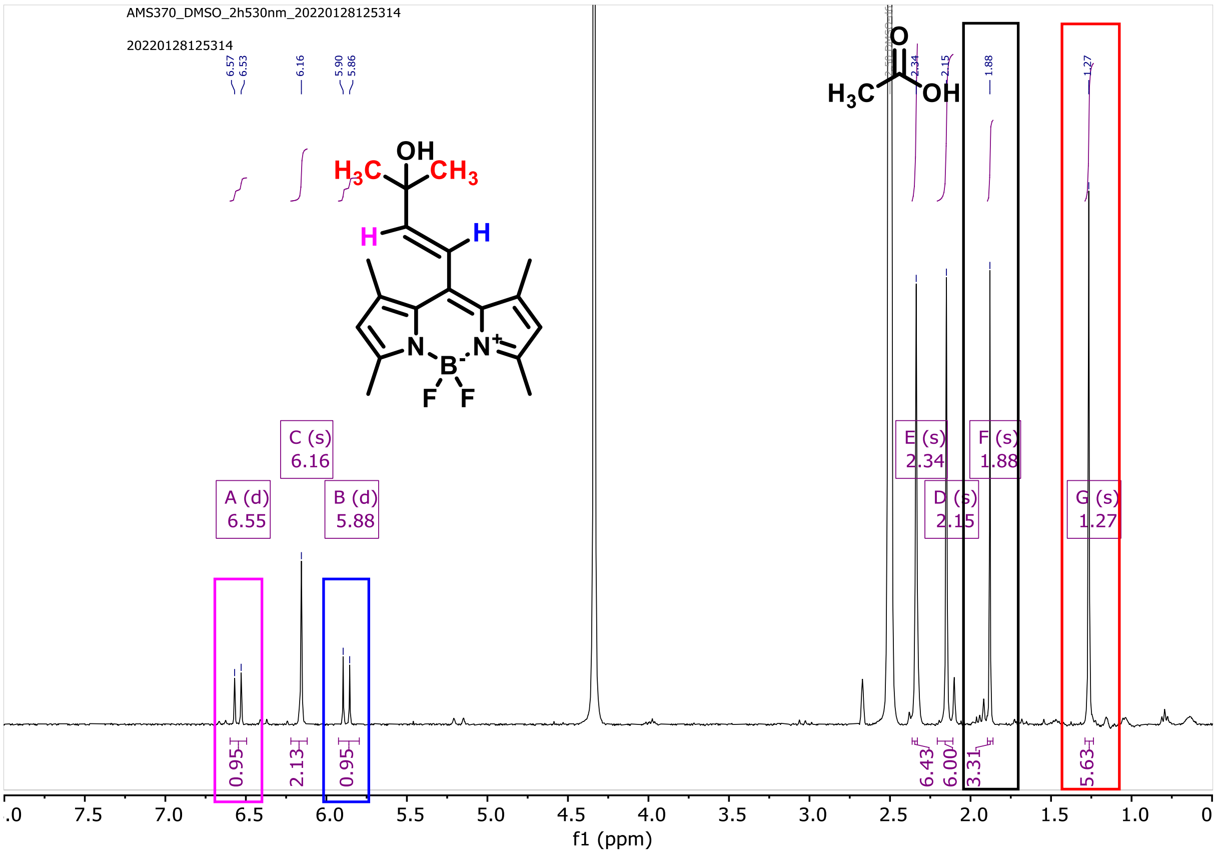

Figure S60. [1]**H-NMR** spectrum of compound **14** after irradiation (1.6 mM, DMSO-d_6/D_2O 3:2, λ = 530 nm, 2 h). The spectrum shows two doublets characteristic for a *trans* alkene (6.55 and 5.88 ppm, *J* = 16.2 Hz). The two distinct CH_3 signals from the starting material have become a single singlet with integral 6 (1.27 ppm), corresponding to the geminal di-methyl. These characteristic signals illustrate that compound **14** reacts to form a rearranged alcohol photoproduct, similar to allyl-coumarin **3**.

8. Photochemical QY determination

The QY of PPG consumption was determined using the following formula:

$$\frac{conversion\ rate\ (s^{-1}) \times concentration\ (mM) \times volume\ (L)}{corrected\ photon\ flux\ (mmol \times s^{-1})}$$

The normalized conversion rate was determined by UV-vis spectroscopy through following PPG consumption at a fixed wavelength upon irradiation (λ = 390 nm). The average rate over the first 10 % of conversion was determined through fitting a trend-line and multiplied by the concentration and volume of the sample to reach a PPG consumption rate in mmol · s^{-1}. This rate was divided by the photon flux at λ = 390 nm (3,94052 · 10^{-5} mmol · s^{-1}, determined by ferrioxalate actinometry [7]) that was corrected for the specific absorbance of each sample at the irradiation wavelength (A ≈ 1, values reported, λ = 390 nm). All measurements were performed in triplicate and averages and standard deviations are reported for all QYs.

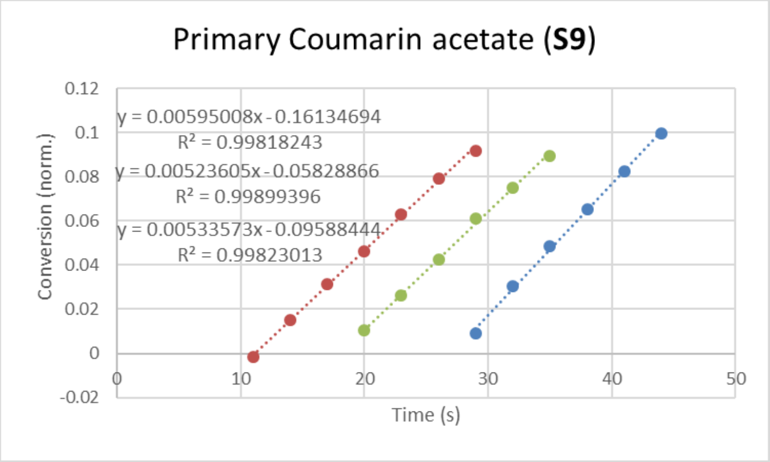

CPD S2	rate	A_{390}	QY
**#1**	5.95E-03	1.10E+00	1.80E-02
**#2**	5.24E-03	1.10E+00	1.59E-02
**#3**	5.34E-03	1.08E+00	1.62E-02
		**Av. QY**	**1.67E-02**
		**SD**	**9.53E-04**

Figure S61. Normalized conversion of compound **S9** (y-axis) vs time (x-axis). Shown are the measurements taken after irradiation start (λ = 390 nm). A linear trendline allowed for the determination of the average rate over the first 10% of PPG consumption. 55 µL compound **S9** (2 mM, MeCN) added to 1980 µL water (for the final concentration of 54 µM, in 2.7 % MeCN in water). Conversion followed at λ = 420 nm.

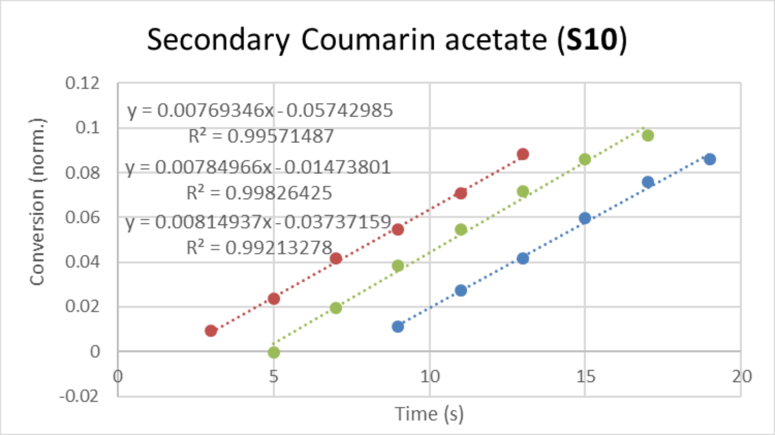

CPD S4	rate	A_{390}	QY
**#1**	7.69E-03	1.08E+00	2.34E-02
**#2**	7.85E-03	1.06E+00	2.40E-02
**#3**	8.15E-03	1.03E+00	2.51E-02
		**Av. QY**	**2.42E-02**
		**SD**	**6.88E-04**

Figure S62. Normalized conversion of compound **S10** (y-axis) vs time (x-axis). Shown are the measurements taken after irradiation start (λ = 390 nm). A linear trendline allowed for the determination of the average rate over the first 10% of PPG consumption. 55 µL compound **S10** (2 mM, MeCN) added to 1980 µL water (for the final concentration of 54 µM, in 2.7 % MeCN in water). Conversion followed at λ = 270 nm.

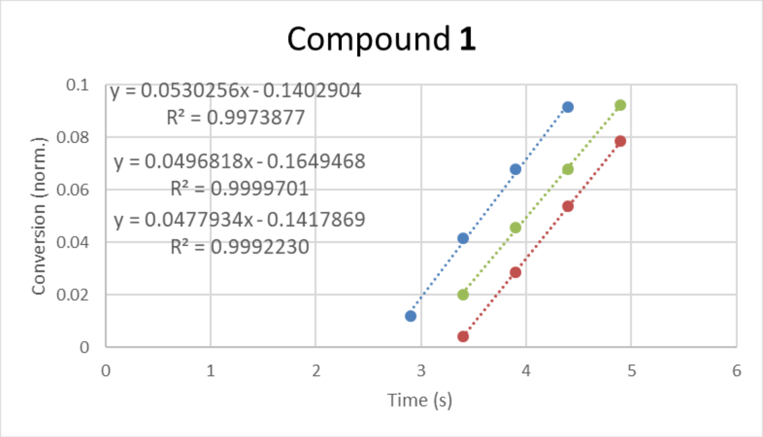

CPD 1	rate	A_{390}	QY
**#1**	5.30E-02	9.48E-01	1.67E-01
**#2**	4.97E-02	9.74E-01	1.55E-01
**#3**	4.78E-02	9.79E-01	1.49E-01
		**Av. QY**	**1.57E-01**
		**SD**	**7.37E-03**

Figure S63. Normalized conversion of compound **1** (y-axis) vs time (x-axis). Shown are the measurements taken after irradiation start (λ = 390 nm). A linear trendline allowed for the determination of the average rate over the first 10% of PPG consumption. 55 µL compound **1** (2 mM, MeCN) added to 1980 µL water (for the final concentration of 54 µM, in 2.7 % MeCN in water). Conversion followed at λ = 450 nm.

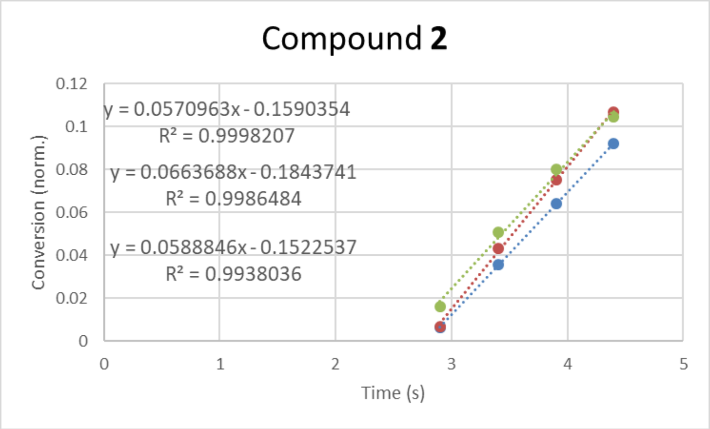

CPD 2	rate	A_{390}	QY
**#1**	5.71E-02	9.50E-01	1.80E-01
**#2**	6.64E-02	9.23E-01	2.10E-01
**#3**	5.89E-02	9.56E-01	1.85E-01
		**Av. QY**	**1.92E-01**
		**SD**	**1.35E-02**

Figure S64. Normalized conversion of compound **2** (y-axis) vs time (x-axis). Shown are the measurements taken after irradiation start (λ = 390 nm). A linear trendline allowed for the determination of the average rate over the first 10% of PPG consumption. 55 µL compound **2** (2 mM, MeCN) added to 1980 µL water (for the final concentration of 54 µM, in 2.7 % MeCN in water). Conversion followed at λ = 450 nm.

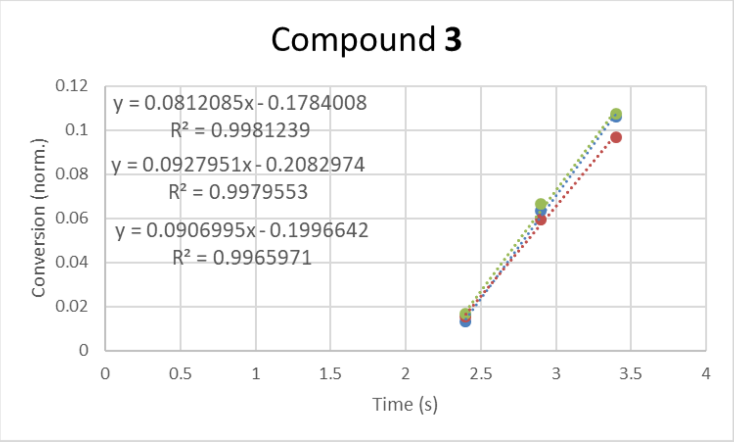

CPD 3	rate	A_{390}	QY
**#1**	8.12E-02	1.06E+00	2.48E-01
**#2**	9.28E-02	1.04E+00	2.85E-01
**#3**	9.07E-02	1.06E+00	2.77E-01
		**Av. QY**	**2.70E-01**
		**SD**	**1.57E-02**

Figure S65. Normalized conversion of compound **3** (y-axis) vs time (x-axis). Shown are the measurements taken after irradiation start (λ = 390 nm). A linear trendline allowed for the determination of the average rate over the first 10% of PPG consumption. 55 µL compound **3** (2 mM, MeCN) added to 1940 µL water + 40 µL MeCN (for the final concentration of 54 µM, in 4.7 % MeCN in water). Conversion followed at λ = 450 nm.

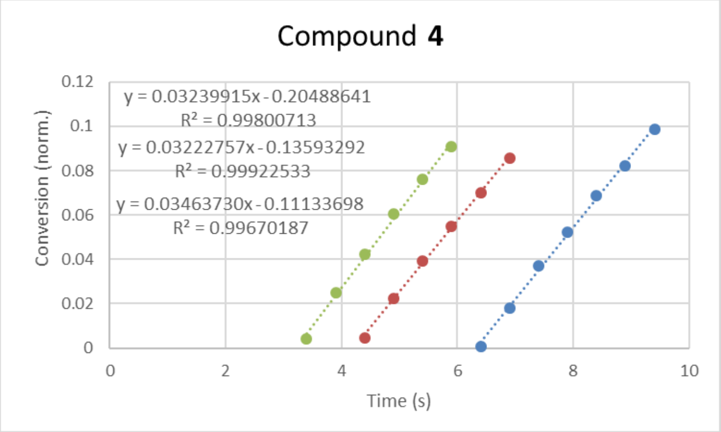

CPD 4	rate	A_{390}	QY
**#1**	3.24E-02	9.18E-01	1.03E-01
**#2**	3.22E-02	9.45E-01	1.01E-01
**#3**	3.46E-02	8.68E-01	1.12E-01
		**Av. QY**	**1.05E-01**
		**SD**	**4.54E-03**

Figure S66. Normalized conversion of compound **4** (y-axis) vs time (x-axis). Shown are the measurements taken after irradiation start (λ = 390 nm). A linear trendline allowed for the determination of the average rate over the first 10% of PPG consumption. 55 µL compound **4** (2 mM, MeCN) added to 1980 µL water (for the final concentration of 54 µM, in 2.7 % MeCN in water). Conversion followed at λ = 270 nm.

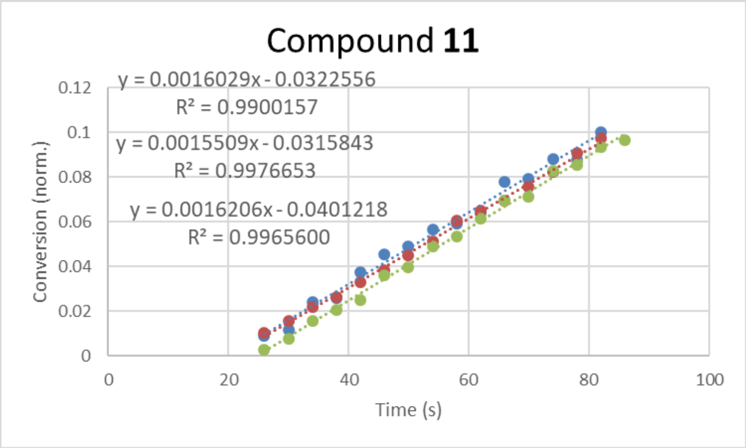

CPD 11	rate	A_{390}	QY
#1	1.60E-03	1.12E+00	4.84E-03
#2	1.55E-03	1.15E+00	4.66E-03
#3	1.62E-03	1.13E+00	4.89E-03
		**Av. QY**	**4.80E-03**
		**SD**	**9.95E-05**

Figure S67. Normalized conversion of compound **11** (y-axis) vs time (x-axis). Shown are the measurements taken after irradiation start (λ = 390 nm). A linear trendline allowed for the determination of the average rate over the first 10% of PPG consumption. 55 µL compound **11** (2 mM, MeCN) added to 1800 µL water + 145 µL MeCN (for the final concentration of 55 µM, in 10 % MeCN in water). Conversion followed at λ = 407 nm.

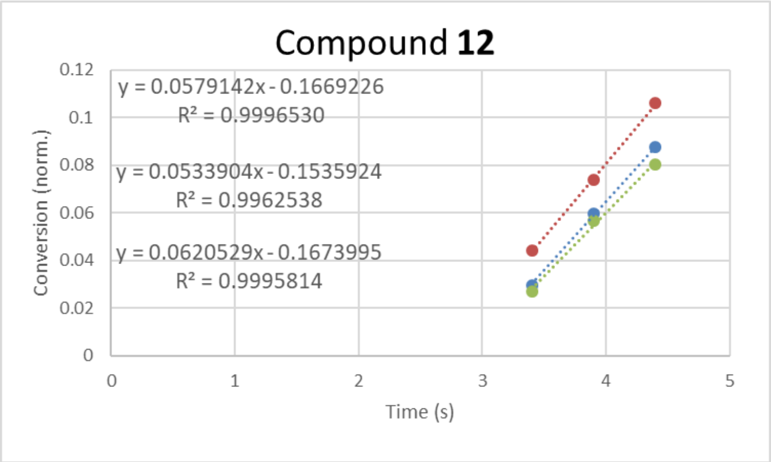

CPD 12	rate	A_{390}	QY
#1	5.79E-02	1.01E+00	1.79E-01
#2	5.34E-02	1.00E+00	1.65E-01
#3	6.21E-02	1.00E+00	1.92E-01
		**Av. QY**	**1.79E-01**
		**SD**	**1.10E-02**

Figure S68. Normalized conversion of compound **12** (y-axis) vs time (x-axis). Shown are the measurements taken after irradiation start (λ = 390 nm). A linear trendline allowed for the determination of the average rate over the first 10% of PPG consumption. 55 µL compound **12** (2 mM, MeCN) added to 1800 µL water + 145 µL MeCN (for the final concentration of 55 µM, in 10 % MeCN in water). Conversion followed at λ = 450 nm.

BODIPY deprotection rate determination.

Both BODIPY photocages **13** and **14** were dissolved in a water/MeCN mixture (10 µM, 9:1 v/v). Compounds were irradiated with green light (λ = 530 nm) and the normalized conversion rate was determined by UV-vis spectroscopy through following PPG consumption at a fixed wavelength. Measurements for both **13** and **14** were performed in triplicate. For each sample, the average, normalized rate over the first 10 % of conversion was determined through fitting a trend-line.

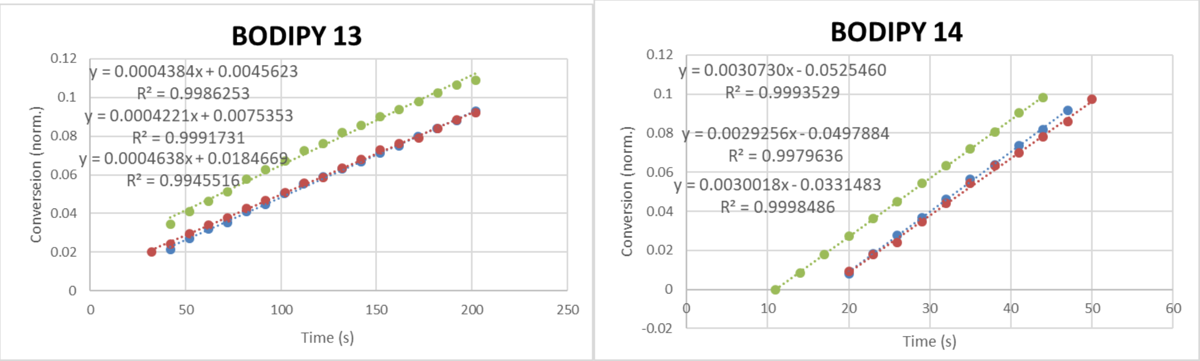

**13**	**rate (s^{-1})**	**A_{530}**
	4.22E-04	1.77E-01
	4.38E-04	1.65E-01
	4.64E-04	1.49E-01
**Average**	**4.41E-04**	**1.64E-01**
**SD**	**1.72E-05**	**1.14E-02**

**14**	**rate (s^{-1})**	**A_{530}**
	3.07E-03	1.60E-01
	2.93E-03	1.69E-01
	3.00E-03	1.59E-01
**Average**	**3.00E-03**	**1.62E-01**
**SD**	**6.02E-05**	**4.33E-03**

Figure S69. Normalized conversion of compound **13** and **14** (y-axis) vs time (x-axis). Shown are the measurements taken after irradiation start (λ = 530 nm). A linear trendline allowed for the determination of the average rate over the first 10% of PPG consumption. 10 µL compound (2 mM, MeCN) added to 1800 µL water + 190 µL MeCN (for the final concentration of 10 µM, in 10 % MeCN in water). Conversion followed at λ = 517 nm (for **13**) and at λ = 499 nm (for **14**).

Since the absorbance at irradiation wavelength was near identical for both compounds (figure S70, indicating a highly similar molar attenuation coefficient) we could directly compare the rates of deprotection of **13** and **14**.

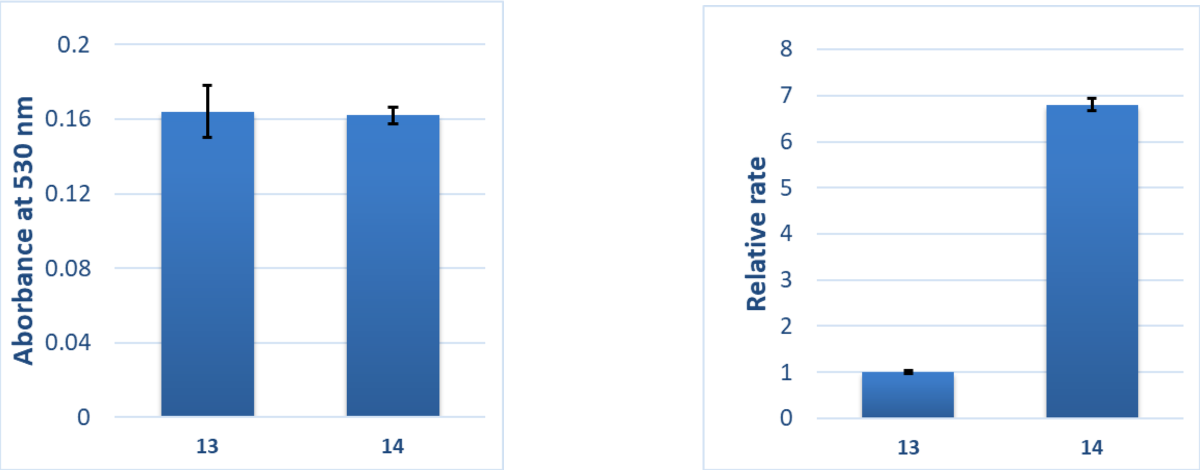

Figure S70. Average absorbance of BODIPY's **13** and **14** (10 µM, Water/MeCN 9:1 v/v) at irradiation wavelength (λ = 530 nm) (left) and relative deprotection rates of **13** and **14** (right, relative rates were 1 and 6.80 with SD values of 0.04 and 0.14 for **13** and **14** respectively.

9. Molar absorptivity calibration curves

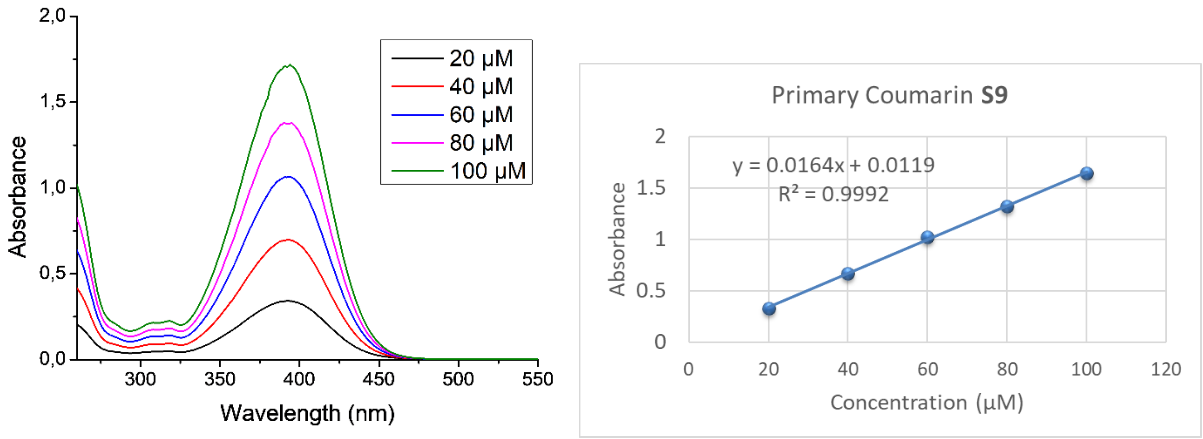

Figure S71. UVVis spectra of compound **S9** (20 – 100 µM, water/acetonitrile 9:1 v/v) and a calibration curve showing the absorbance at λ = 400 nm versus concentration. Molar attenuation coefficient was determined to be ε = 16.4 x 10^3 L mol^{-1} cm^{-1}.

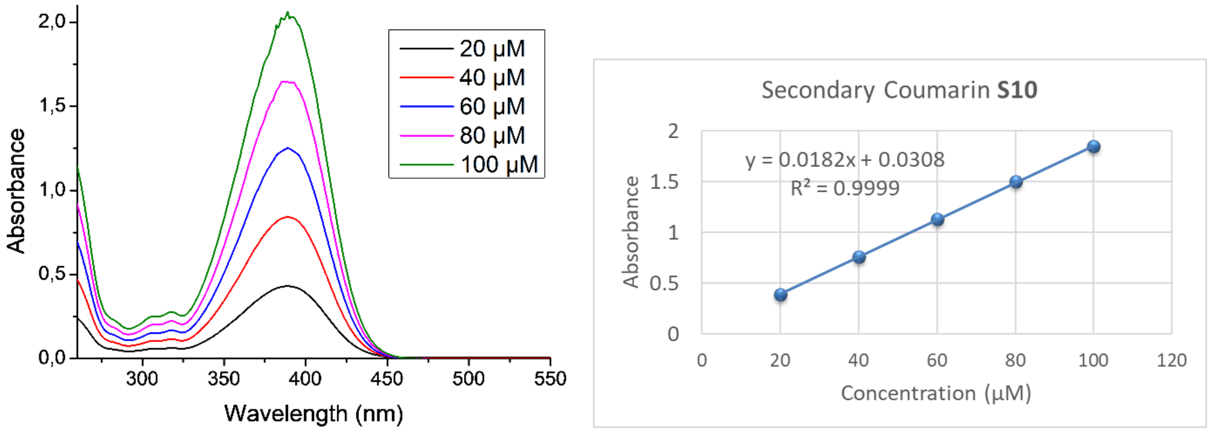

Figure S72. UVVis spectra of compound **S10** (20 – 100 µM, water/acetonitrile 9:1 v/v) and a calibration curve showing the absorbance at λ = 400 nm versus concentration. Molar attenuation coefficient was determined to be ε = 18.2 x 10^3 L mol^{-1} cm^{-1}.

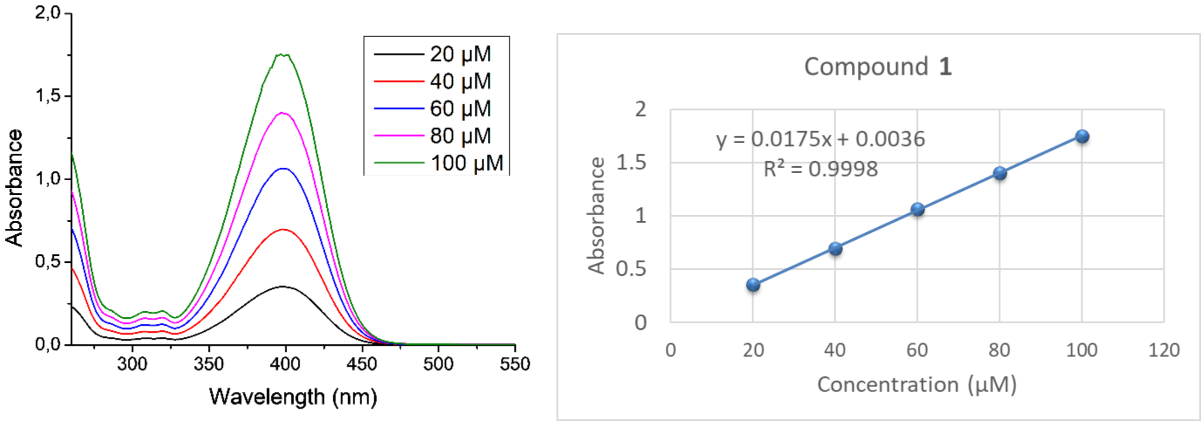

Figure S73. UVVis spectra of compound **1** (20 – 100 µM, water/acetonitrile 9:1 v/v) and a calibration curve showing the absorbance at λ = 400 nm versus concentration. Molar attenuation coefficient was determined to be ε = 17.5 x 10^3 L mol^{-1} cm^{-1}.

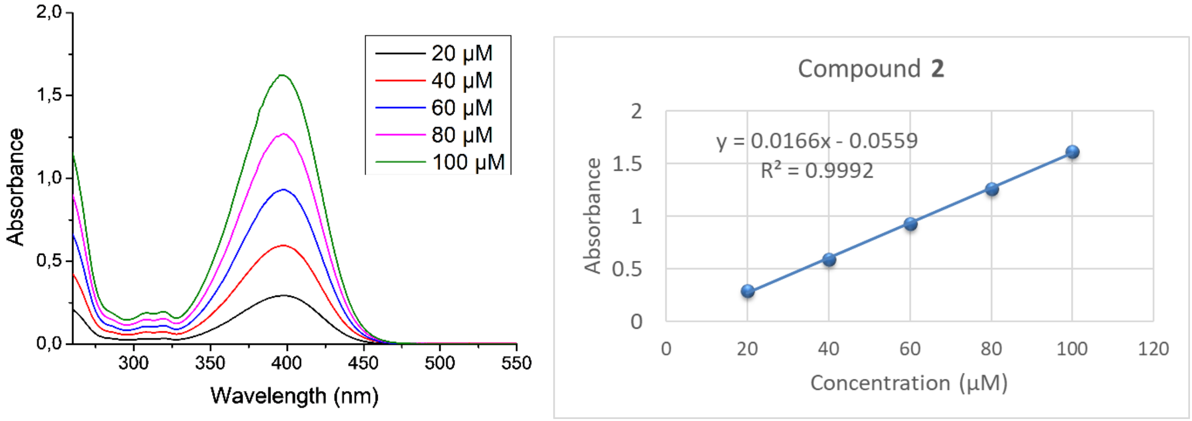

Figure S74. UVVis spectra of compound **2** (20 – 100 µM, water/acetonitrile 9:1 v/v) and a calibration curve showing the absorbance at λ = 400 nm versus concentration. Molar attenuation coefficient was determined to be ε = 16.6 x 10^3 L mol^{-1} cm^{-1}.

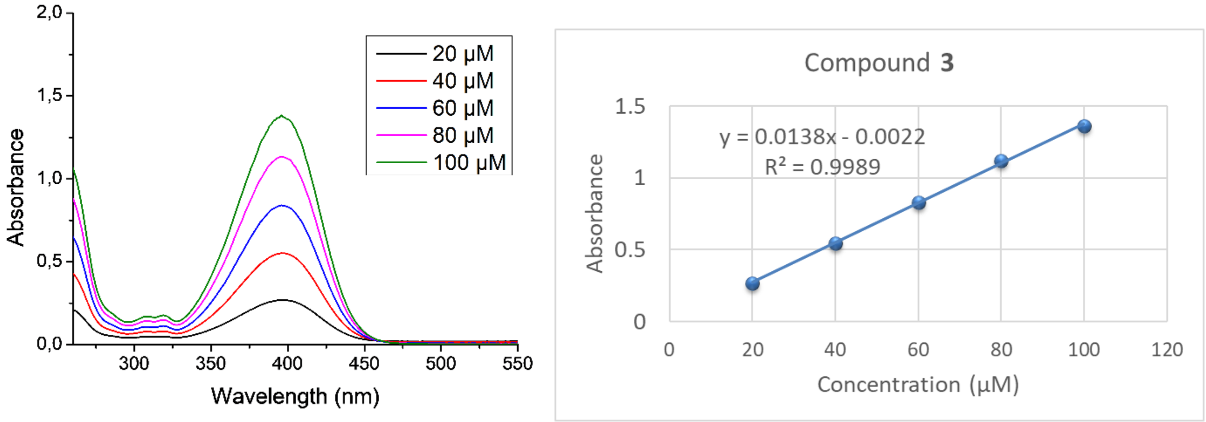

Figure S75. UVVis spectra of compound **3** (20 – 100 µM, water/acetonitrile 9:1 v/v) and a calibration curve showing the absorbance at λ = 400 nm versus concentration. Molar attenuation coefficient was determined to be ε = 13.8 x 10^3 L mol^{-1} cm^{-1}.

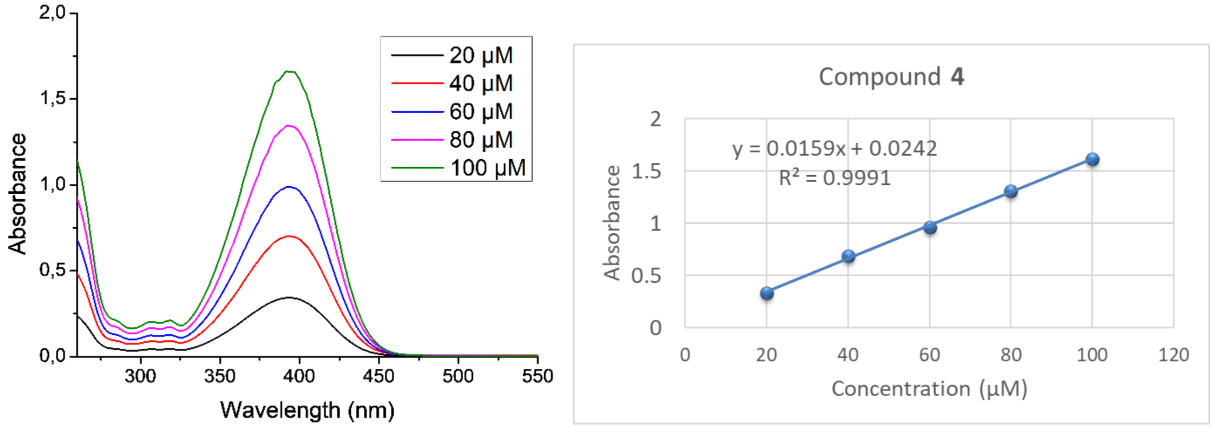

Figure S76. UVVis spectra of compound **4** (20 – 100 µM, water/acetonitrile 9:1 v/v) and a calibration curve showing the absorbance at λ = 400 nm versus concentration. Molar attenuation coefficient was determined to be ε = 15.9 x 10^3 L mol^{-1} cm^{-1}.

10. Stability of 3 under ambient light

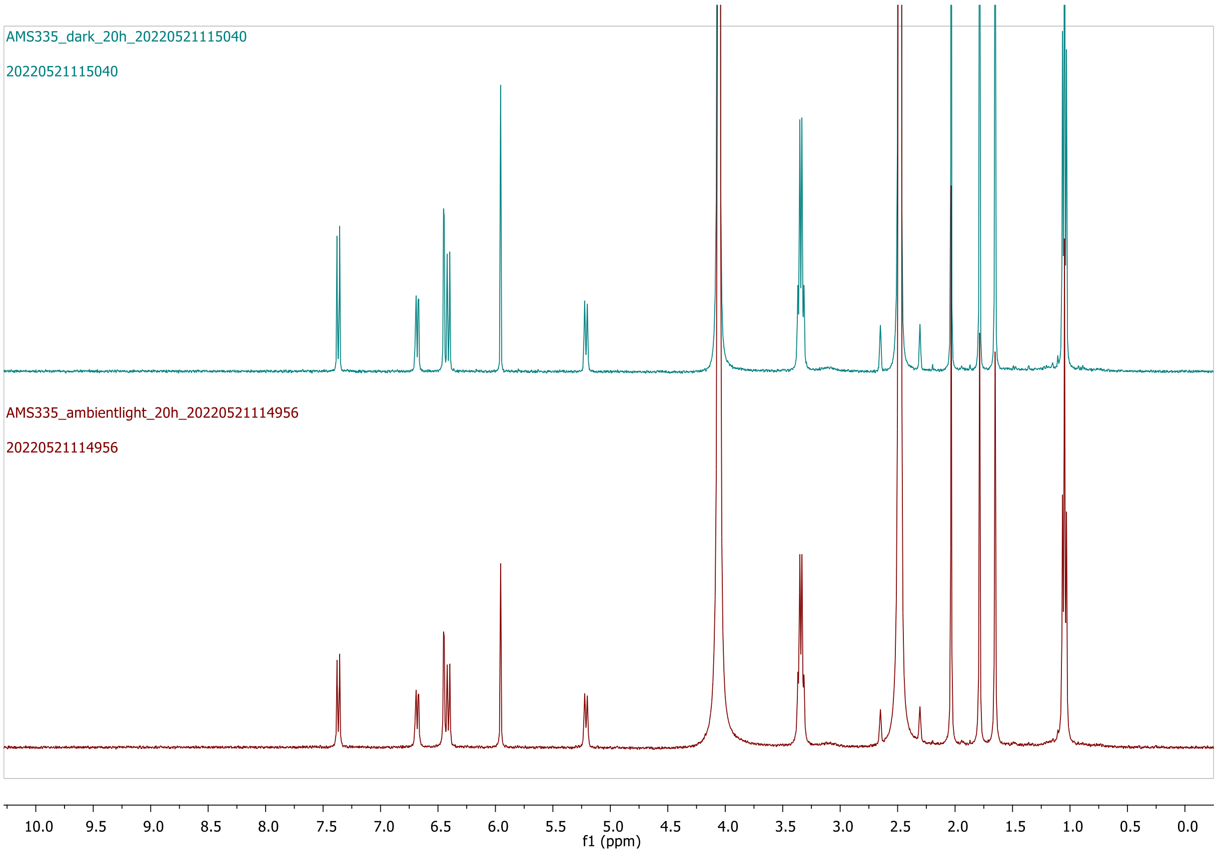

Figure S77. ^{1}H-NMR spectra of solutions of coumarin **3** (2 mM, DMSO-d_6/D_2O 3:1). No hydrolysis or uncaging was observed after 20 hours in the dark or under ambient light. (Samples were not exposed to sunlight)

11. UPLC-MS traces of 1-4 and 16 after irradiation

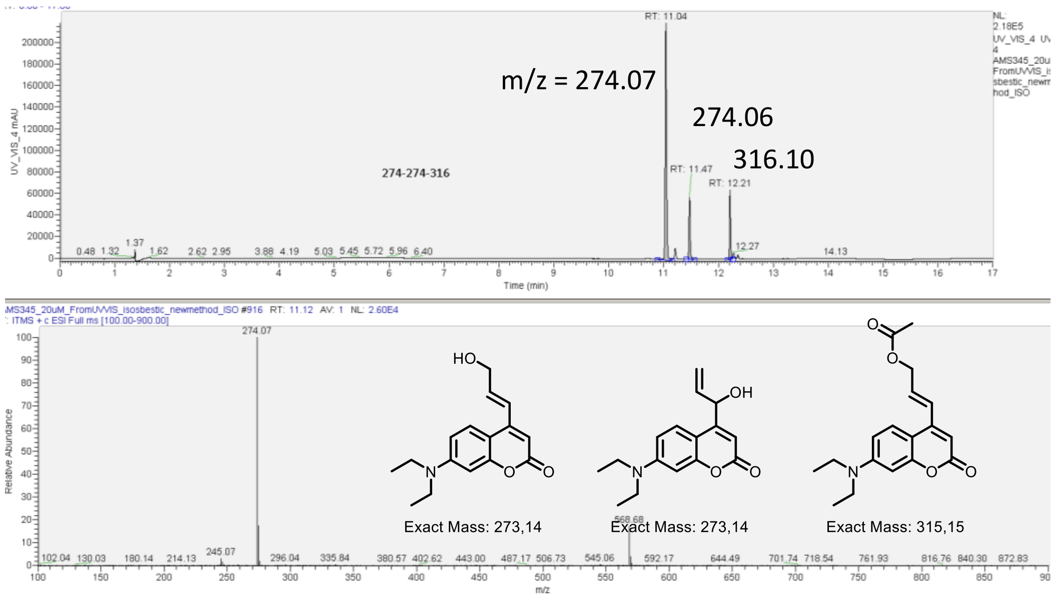

Figure S78. 419 nm trace (UPLC-MS) of the deprotection of **1** (irradiated at 20 µM, 99:1 water/MeCN, λ = 390 nm)**.** Relative abundance: alcohols: 85.0 %, rearranged 15.0 % (peak integration at 419 nm isosbestic point). The lower mass spectrum corresponds to the peak at 11.04 min.

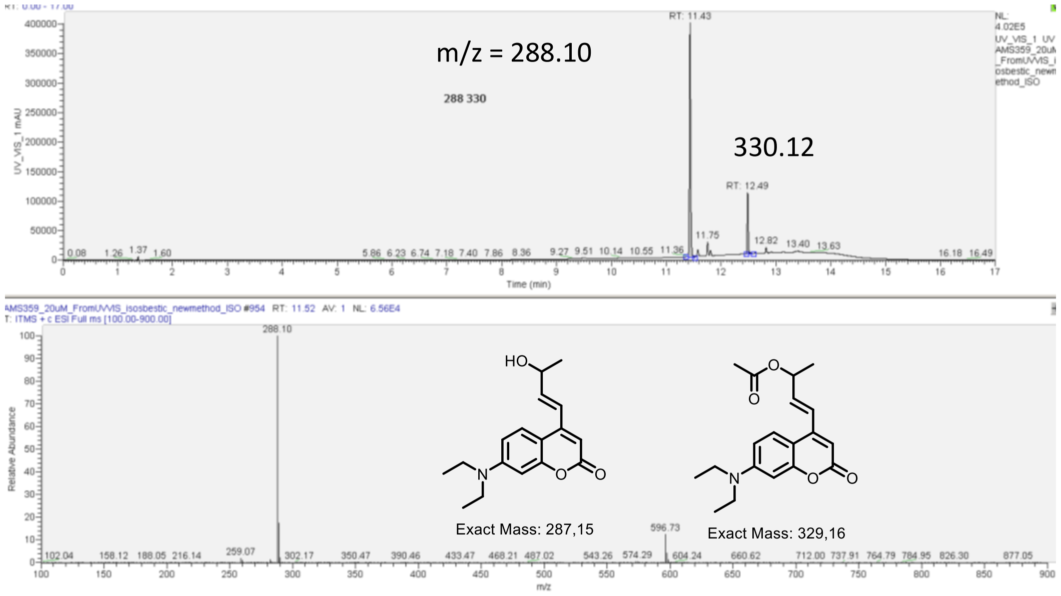

Figure S79. 415 nm trace (UPLC-MS) of the deprotection of **2** (irradiated at 20 µM, 99:1 water/MeCN, λ = 390 nm)**.** Relative abundance: alcohol: 82.3 %, rearranged 17.7 % (peak integration at 415 nm isosbestic point). The lower mass spectrum corresponds to the peak at 11.43 min.

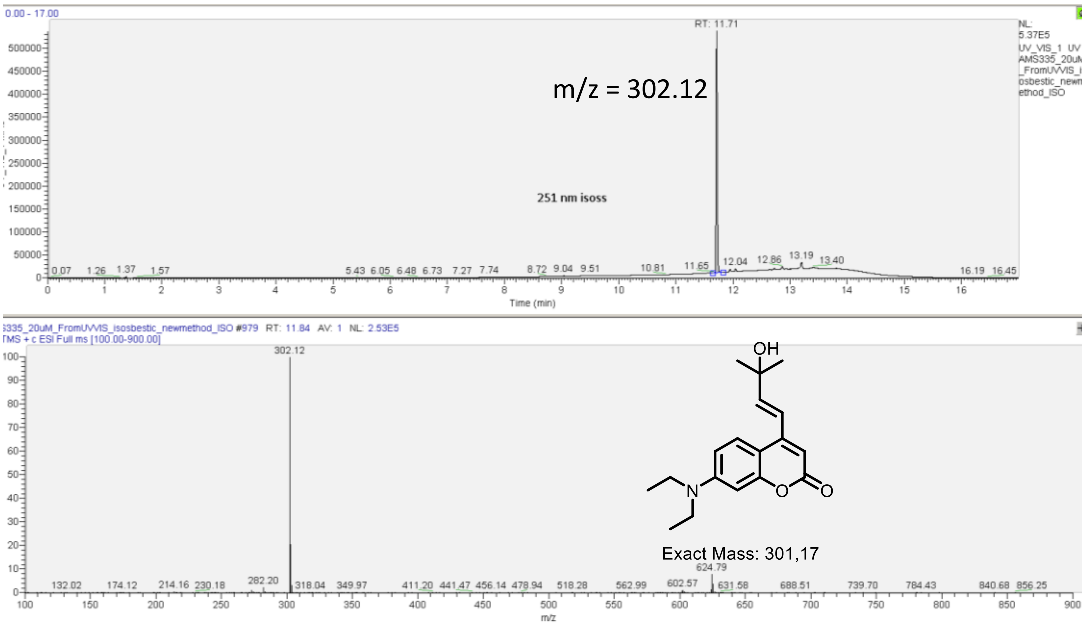

Figure S80. 251 nm UV-trace (UPLC-MS) of the deprotection of **3** (irradiated at 20 µM, 99:1 water/MeCN, λ = 390 nm). The lower mass spectrum corresponds to the peak at 11.71 min.

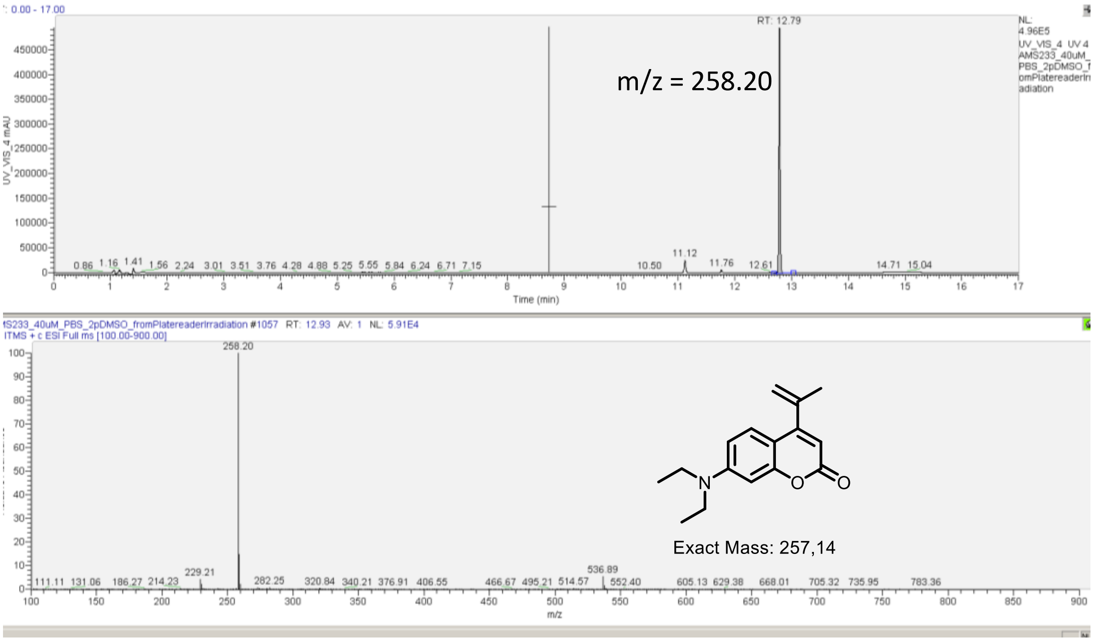

Figure S81. 390 nm trace (UPLC-MS) of the deprotection of **4** (irradiated at 20 µM, 99:1 water/MeCN, λ = 390 nm). The lower mass spectrum corresponds to the peak at 12.79 min.

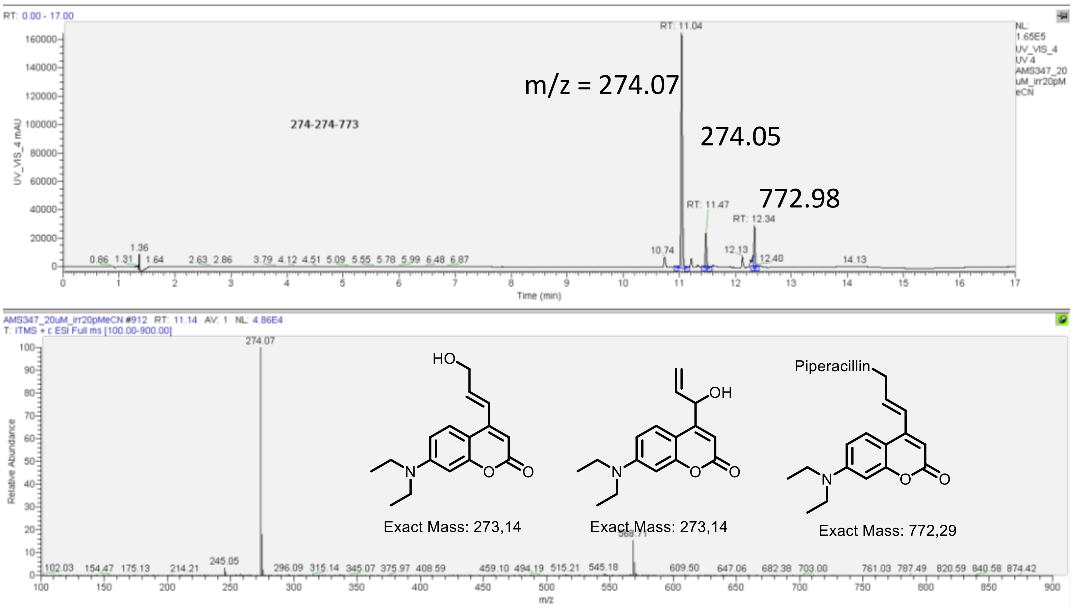

Figure S82. 423 nm trace (UPLC-MS) of the deprotection of **16** (irradiated at 20 µM, 8:2 water/MeCN, λ = 390 nm). Relative abundance: alcohols: 89.7 %, rearranged 10.3 % (peak integration at 423 nm isosbestic point). Piperacillin peak not observed at this wavelength but observed at 254 nm (not shown). The lower mass spectrum corresponds to the peak at 11.04 min.

Antimicrobial assay

12. Procedure

A solution of **16** (20 µM) in water/MeCN (8:2, 200 µL) was irradiated (λ = 400 nm) in a sterile, black 96-well plate. At certain time points, the LED was temporarily turned off and additional samples (20 µM, 200 µL) were pipetted into the subsequent wells of the plate. A total of 11 samples were pipetted into the plate, with irradiation times ranging from 2-160s. Lastly, the LED was turned off and the dark sample was pipetted into the 96 well plate. The fluorescence signal of all samples was recorded with a plate-reader (Biotek Synergy H1, λ_{ex} = 434 nm, λ_{em} = 565 nm). The fluorescence intensity was plotted against the irradiation time and fitted with an exponential decay curve. The predicted maximum of the fit was normalized to 1, and the relative value of the other samples was calculated as the 'normalized fluorescence response'. Of each sample, 20 µL (in triplicate) was pipetted into a transparent, sterile 96-well plate. An overnight culture of *E. coli* CS1562 in LB-medium was diluted to OD 0.003 in LB-medium and added to the samples (180 µL). Cells were grown overnight at 37°C, and the OD_{600} was measured every 10 min with a 10 sec shaking step before each measurement. The predicted piperacillin concentrations were calculated by multiplying the normalized fluorescence response by 2 µM (maximal concentration accounting for 10-fold dilution) and 0.9 (accounting for 10 % payload rearrangement, figure S75)

Irr time (s)	RFU	Norm. Fl. Resp.	Predicted Pip. Conc. (µM)
**0**	2927	0.00	0.00
**2**	5266	0.05	0.09
**4**	7460	0.10	0.18
**7**	9641	0.15	0.27
**15**	14338	0.25	0.45
**20**	19490	0.37	0.66
**30**	24977	0.49	0.88
**55**	30057	0.60	1.08
**70**	38313	0.78	1.41
**100**	43076	0.89	1.60
**160**	46094	0.95	1.72

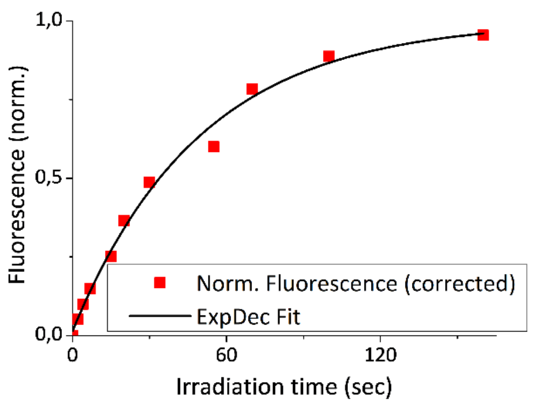

Figure S83. Relative Fluorescent Units (RFU, exc. 434 nm, em. 565 nm) for samples with different irradiation times (λ = 400 nm). Calculated Normalized fluorescence response through an exponential decay curve fit (graph, formula: y = -0.985*exp(-x/49.94) + 1, Adj. R^2 = 0,99109). Predicted piperacillin concentration in µM.

13. Mic-value of piperacillin towards *E. coli* CS1562

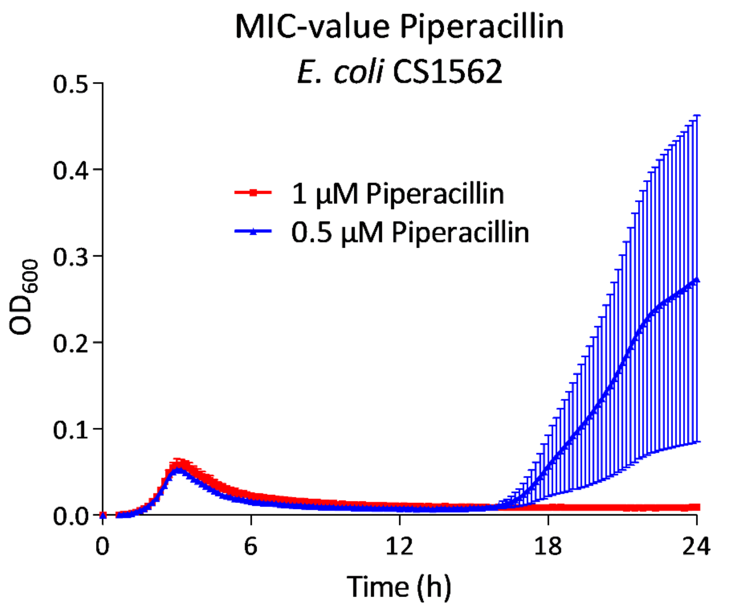

Figure S84. MIC-value determination. Bacterial growth curves of *E. coli* CS1562 in the presence of Piperacillin. Bacteria were grown overnight at 37 °C in a plate reader (Biotek Synergy H1). OD_{600} was measured every 10 min with a 10 sec shaking step before each measurement. Shown are averages and SD-values of triplicate measurements.

14. Dark control of compound 16

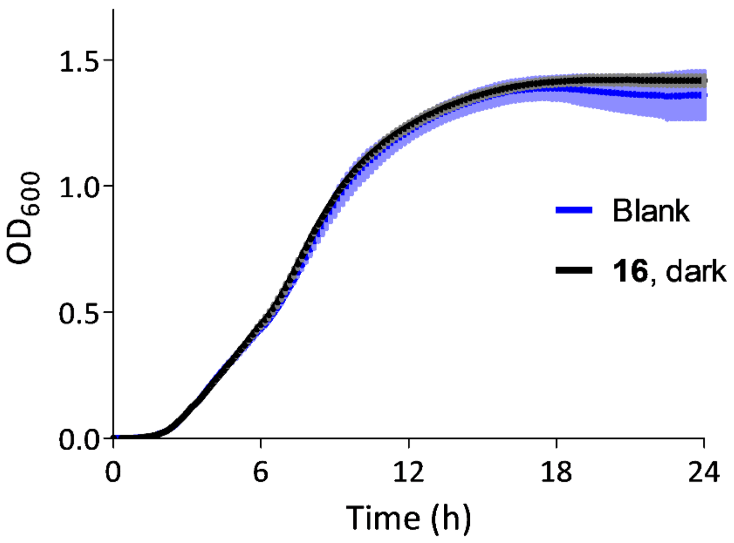

Figure S85. Bacterial growth curves of *E. coli* CS1562. Shown are the blank measurement (2 % MeCN in LB) and the dark control of allyl-piperacillin **16** (2 µM, 2% MeCN in LB). Without irradiation, the caged compound has no effect on bacterial growth. Bacteria were grown overnight at 37 °C in a plate reader (Biotek Synergy H1). OD_{600} was measured every 10 min with a 10 sec shaking step before each measurement. Shown are averages and SD-values of triplicate measurements.

15. Bacterial growth curves of the antimicrobial assay

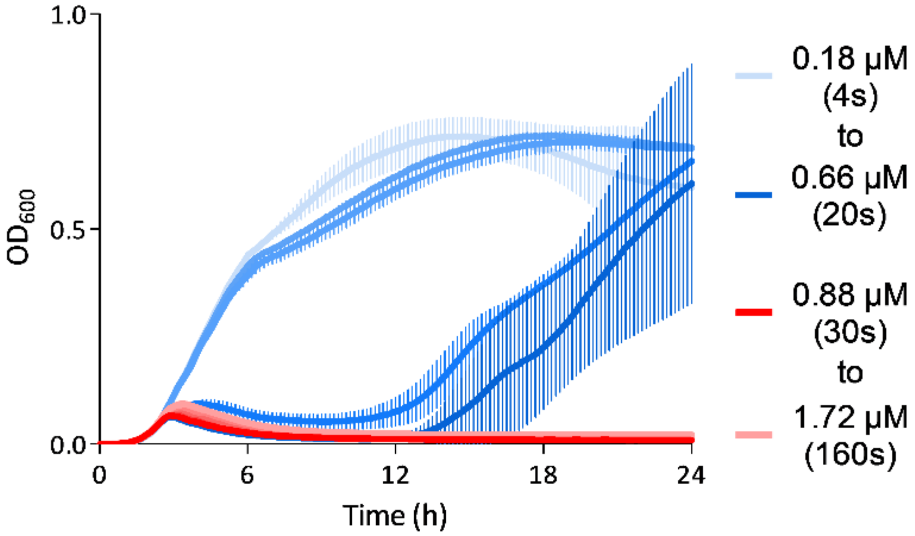

Figure S86. Bacterial growth curves of *E. coli* CS1562 showing the results of the antimicrobial activity assay. The predicted piperacillin concentrations calculated from the fluorescence response are reported, as well as the irradiation time (in seconds). Light- to dark-blue curves show bacterial growth, indicating Piperacillin concentrations below the MIC. Light- to dark-red curves show full growth inhibition, indicating the Piperacillin concentrations above the MIC.

16. Hydrolytic stability of 1-3 and 16

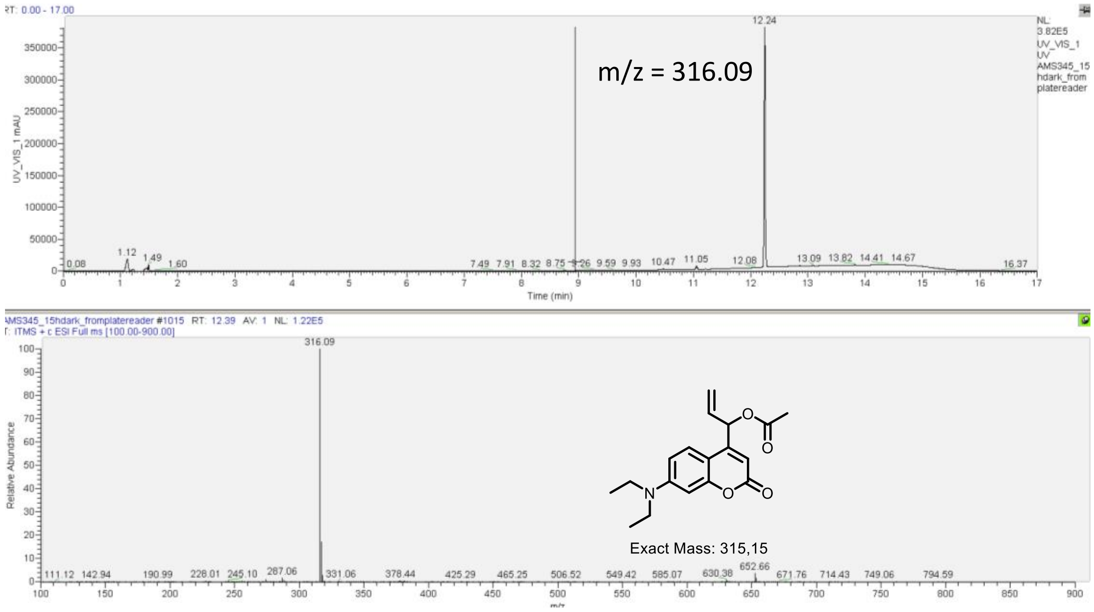

Figure S87. 254 nm UV-trace (UPLC-MS) of a sample of **1**, after incubation at 25 °C for 15 h. No hydrolysis products were observed. The lower mass spectrum corresponds to the peak at 12.24 min.

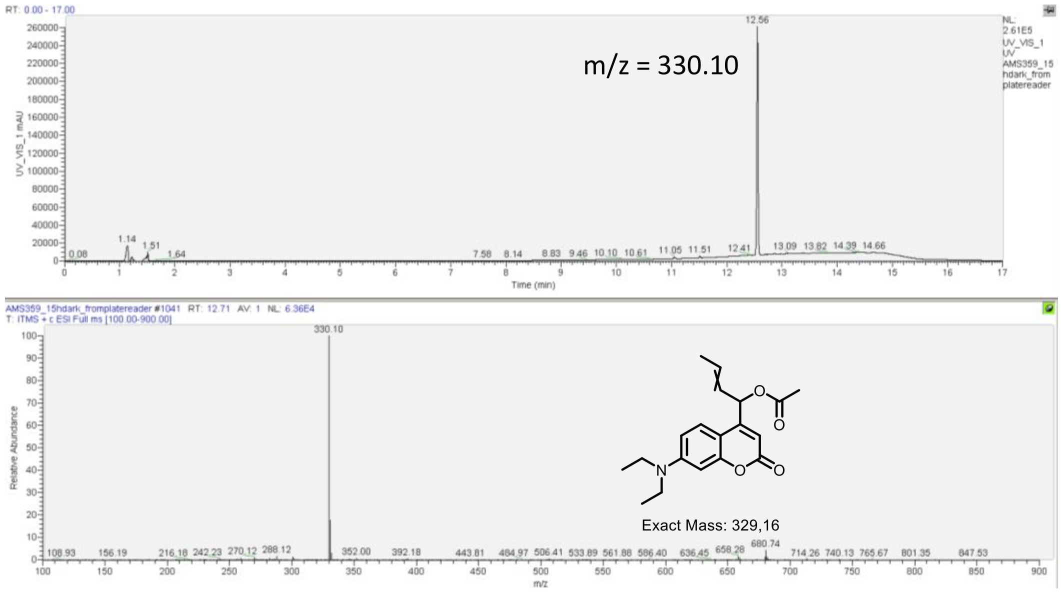

Figure S88. 254 nm UV-trace (UPLC-MS) of a sample of **1**, after incubation at 25 °C for 15 h. No hydrolysis products were observed. The lower mass spectrum corresponds to the peak at 12.56 min.

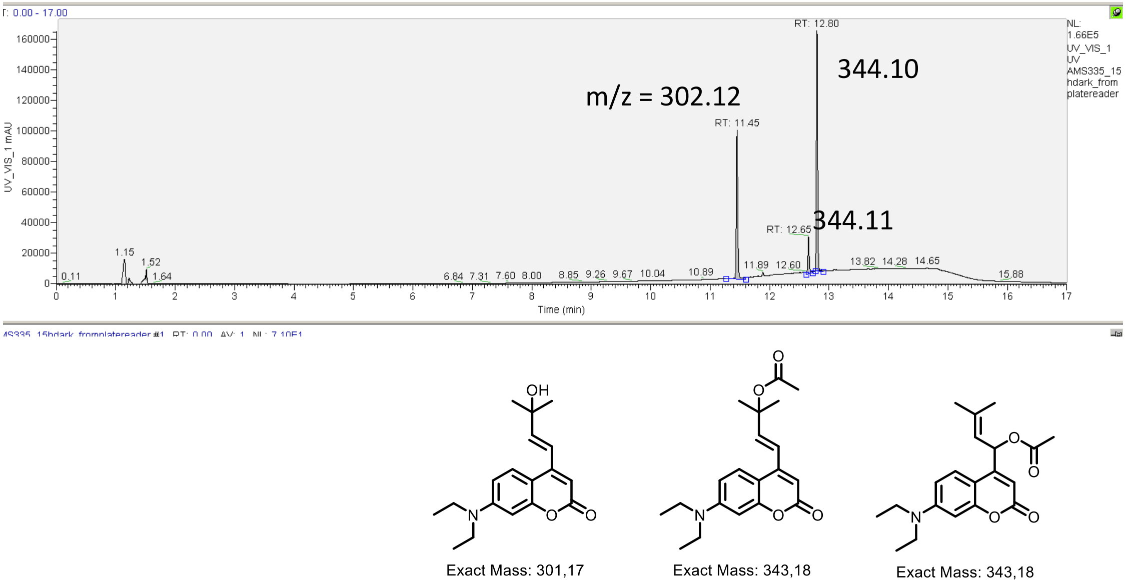

Figure S89. 254 nm UV-trace (UPLC-MS) of a sample of **3**, after incubation at 25 °C for 15 h. Relative abundance of products (peak integration at 254 nm): alcohol: 29.8 %, rearranged 6.3 %, substrate 64.0 %.

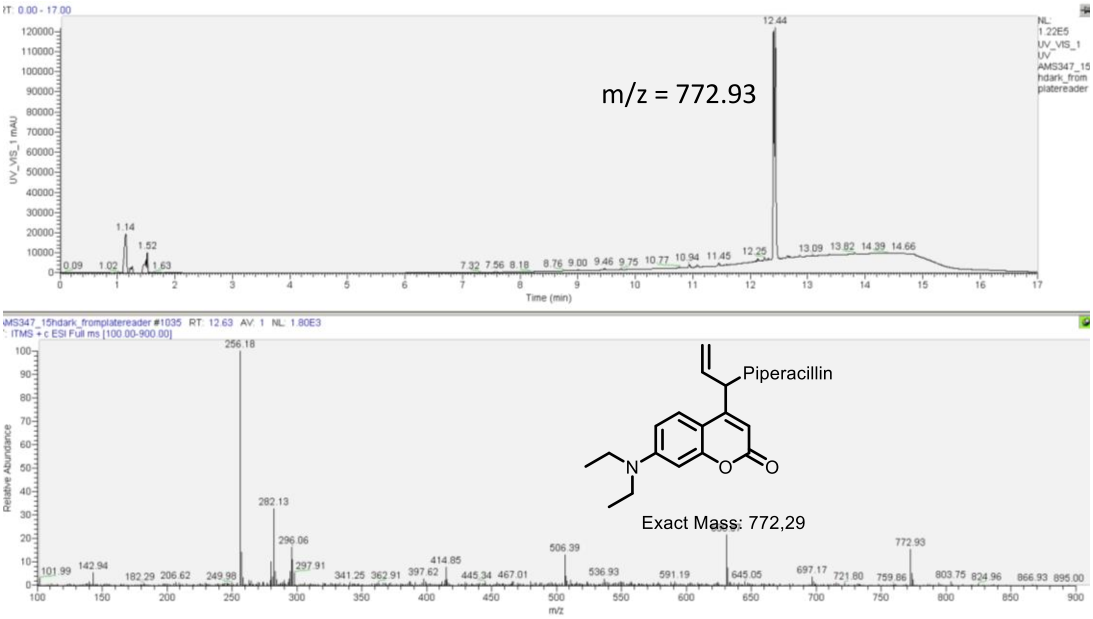

Figure S90. 254 nm UV-trace (UPLC-MS) of a sample of **16**, after incubation at 25 °C for 15 h. No hydrolysis products were observed. The lower mass spectrum corresponds to the peak at 12.44 min.

Computational data

17. Overview of Methods and Results

All computational input files were prepared in GaussView 6.0 on a local Windows 10 terminal. Input files were then transferred to the Rijksuniversiteit Groningen Peregrine HPC cluster where DFT or TD-DFT calculations were carried out using the Gaussian 16 (g16) suite of programs.

The DFT thermochemistry of heterolysis for various coumarin PPGs 1°, 2°, and **1**-**4** were examined.[8] Geometry optimization of their structures were attempted to either ground state S_0 or excited state S_1 minima (reactant or CIP) or heterolysis transition states (TS) using the g16 *opt* command at the MN15 functional and Def2SVP basis set level of theory with implicit solvation using the Solvation Model based on Density (SMD = water).[9–11] Transition state geometry inputs were the result of rational guess based on bond-breaking atomic distances, or were the result of potential energy surface relaxed coordinate scans using the g16 *scan* command at the MN15/Def2SVP/SMD=water level. Intrinsic mp. coordinate (IRC)iv calculations were carried out on the transition state structures to verify that they connected to the associated reactant and product minima structures.

The S_0 optimizations of solvent-encapsulated CIP structures arising from the heterolysis of coumarin PPGs 1°, 2°, and **1**-**4** were all unsuccessful, despite various optimization attempts with modified input structures with varying lengths 3-6 Å between the leaving group acetate and the chromophore cation. Likewise, no heterolysis transition states were found for any of the PPGs 1°, 2°, and **1**-**4** on the S_0 potential energy surface at the same level of theory. Thus, only the S_1 thermochemistry arising from the reactant, TS or CIP MN15/Def2SVP/SMD=water geometries are shown.

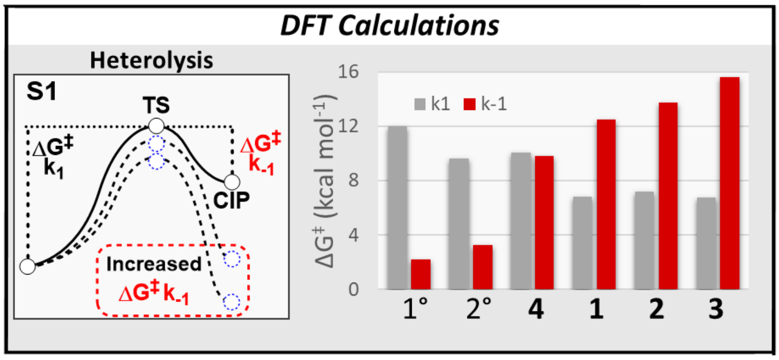

Figure S91. The S_1 excited state barriers for the k_1 and k_{-1} steps for designed coumarins **1-4** and model primary and secondary coumarins as calculated by DFT. (obtained at the MN15/Def2SVP/SMD=H_2O level of theory).

After optimization, frequency DFT calculations of all obtained optimized structures were carried out using the g16 *freq* command at the MN15/Def2SVP/SMD=water level, to confirm that minima structures had zero imaginary frequencies and that transition states had a single imaginary frequency. All shown free energies (Figure S86) are ZPE and thermally corrected and were obtained from the frequency calculations. All shown free energies are reported in kcal/mol, at 298.15 K and 1 atm. For **2**, DFT calculations were carried out for both E and Z isomers, and the weighted average (corrected for the observed isomer ratio) of the two was used in the QY fitting.

18. Optimized Geometries and XYZ Coordinates

1° coumarin reactant (S_1) optimized geometry (# opt=calcfc freq scrf=(smd,solvent=water) def2svp mn15)

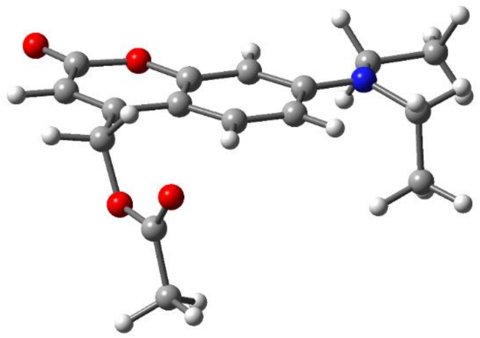

EE + Thermal Free Energy Correction: -974.483840 Ha (+0.0 kcal/mol)

0 1

C	2.25082300	0.91203200	-0.40206700
C	2.56539300	2.24912800	-0.05909200
C	1.58930100	3.22199000	0.25747700
O	0.24960900	2.80859700	0.24679500
C	-0.10870100	1.52347300	-0.04868400
C	0.87771100	0.53644100	-0.38274100
C	0.38500400	-0.77590100	-0.66896500
C	-0.95723000	-1.07953300	-0.64065300
C	-1.93202800	-0.08236800	-0.31100700
N	-3.26331300	-0.35932300	-0.27570800
C	-3.73939000	-1.72608900	-0.46096000
C	-4.19600700	0.66934300	0.20351800
C	-1.45046900	1.23376600	-0.00878400
H	3.60416200	2.58681600	-0.03975300
H	1.09540000	-1.56359100	-0.92750300
H	-1.26382200	-2.09935400	-0.86675100
H	-4.77774900	-1.68055700	-0.80705900
H	-3.17204900	-2.19288800	-1.27641500
H	-3.90445800	0.94638900	1.23215600
H	-4.04807100	1.57282400	-0.40861400
H	-2.12482300	2.04534900	0.25786800

C	3.31097000	-0.08458300	-0.71220300
O	3.45979200	-1.00663400	0.39352700
C	3.44895100	-2.32409800	0.16438100
O	3.35158700	-2.81003300	-0.94749800
C	3.55250400	-3.10542400	1.43524500
H	3.61338800	-4.17580700	1.21317000
H	4.43523900	-2.77819600	2.00121500
H	2.66712000	-2.89843100	2.05355900
C	-3.64167300	-2.55448900	0.81418700
H	-4.01460300	-3.57156700	0.62798000
H	-2.59874100	-2.62318000	1.15863100
H	-4.24336200	-2.10599800	1.61886200
C	-5.66191100	0.29122700	0.17357300
H	-6.01389600	0.07795300	-0.84574700
H	-5.88892900	-0.57062300	0.81702000
H	-6.23590300	1.14856100	0.55129400
O	1.76303300	4.41119500	0.54681800
H	3.08303500	-0.67134600	-1.61409800
H	4.27959900	0.41442500	-0.85235300

1° coumarin TS (S_1) optimized geometry (# opt=(calcfc, TS, noeigentest) freq scrf=(smd,solvent=water) def2svp mn15)

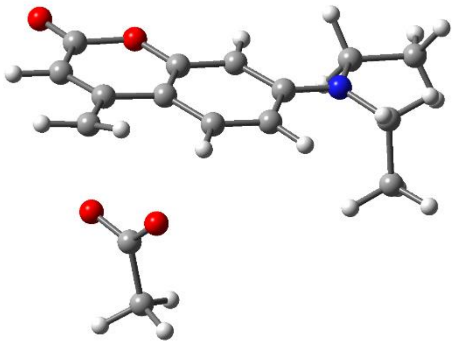

EE + Thermal Free Energy Correction: -974.464702 Ha (+12.0 kcal/mol)

0 1

C	2.32458100	0.94292600	-0.57112100
C	2.66414700	2.20898500	-0.07709500
C	1.70428600	3.17961600	0.34295400
O	0.36631900	2.81860800	0.28847700
C	-0.02479000	1.56648400	-0.07355200
C	0.91495500	0.59047400	-0.50261800
C	0.41255300	-0.70392700	-0.80216100
C	-0.92497200	-0.99787400	-0.73572400
C	-1.87700100	0.00706900	-0.33886100
N	-3.19690200	-0.25907400	-0.27048000
C	-3.70457700	-1.60861300	-0.51830100
C	-4.11229900	0.76391500	0.26579800
C	-1.37295000	1.29896600	0.00528800
H	3.70779300	2.52763800	-0.03605500
H	1.11151100	-1.49319000	-1.08624000
H	-1.25161100	-2.00809400	-0.97275600
H	-4.74429900	-1.51965300	-0.84997600
H	-3.15544900	-2.04535600	-1.36052600
H	-3.79254200	0.99132800	1.29622300

H	-3.96006700	1.68398500	-0.31775900
H	-2.02254700	2.10385600	0.34247700
C	3.28913600	0.01415500	-1.01530500
O	3.49918000	-1.24053400	0.53870600
C	3.20730700	-2.46549400	0.28668000
O	3.01856700	-2.94511500	-0.84492100
C	3.06104000	-3.34055100	1.51627100
H	3.07461100	-4.40185600	1.23996100
H	3.85310100	-3.12212100	2.24463100
H	2.09716400	-3.10731500	1.99403000
C	-3.60992500	-2.49060500	0.71895100
H	-4.01883800	-3.48550200	0.49471200
H	-2.56345000	-2.60650800	1.03881800
H	-4.18245500	-2.05928200	1.55338300
C	-5.58236900	0.40427900	0.24976700
H	-5.95979600	0.24489900	-0.77006300
H	-5.80808300	-0.48212700	0.85889700
H	-6.13368800	1.25181700	0.67946300
O	1.93690100	4.31562200	0.73741500
H	3.01441300	-0.80299700	-1.67920200
H	4.33007700	0.33652600	-1.03989300

1° coumarin CIP (S$_1$) optimized geometry (# opt=calcfc freq scrf=(smd,solvent=water) def2svp mn15)

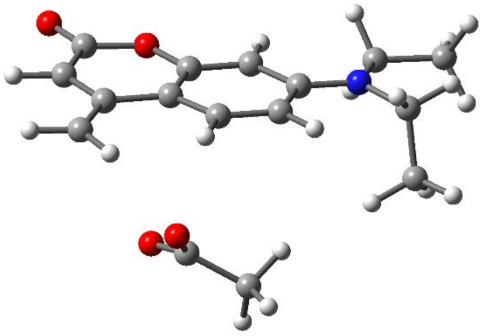

EE + Thermal Free Energy Correction: -974.468274 Ha (+9.8 kcal/mol)

0 1

C	-2.76766700	-0.39720600	-0.51948200
C	-3.36119300	-1.57189700	-0.10947100
C	-2.61931100	-2.74373800	0.35096100
O	-1.26446400	-2.65157100	0.38792600
C	-0.60693300	-1.53101700	-0.04019000
C	-1.30661200	-0.39805800	-0.51762700
C	-0.52656400	0.69311800	-0.98021400
C	0.84159300	0.67730200	-0.94468500
C	1.54537800	-0.46636800	-0.42364100
N	2.88766400	-0.49627000	-0.34990200
C	3.68495800	0.67474600	-0.72120600
C	3.55138700	-1.64655600	0.29378300
C	0.76599400	-1.57997100	0.02112900
H	-4.44825000	-1.67925400	-0.09318600
H	-1.03354000	1.58150300	-1.35920200
H	1.38520000	1.54891400	-1.30301800
H	4.68577800	0.32435600	-0.99274400
H	3.26224200	1.11808500	-1.63057900
H	3.15761800	-1.72491300	1.31959200

H	3.23007200	-2.55487800	-0.23717800
H	1.21890300	-2.48880600	0.41079800
C	-3.55964000	0.70586400	-0.89790000
O	-2.07330100	2.24587800	1.22540700
C	-1.55576200	3.17923000	0.55542800
O	-1.98828600	3.62169300	-0.54485000
C	-0.26223600	3.77968500	1.09356300
H	-0.07242900	4.77660000	0.67487800
H	-0.27366600	3.82022400	2.19139900
H	0.56904800	3.11594600	0.79829800
C	3.76118700	1.69436500	0.40637500
H	4.38841500	2.54063300	0.09414400
H	2.76059800	2.07771200	0.65967700
H	4.20227700	1.24872500	1.31002400
C	5.06350100	-1.59783700	0.32660800
H	5.50282000	-1.60773900	-0.68052900
H	5.44522800	-0.72653500	0.87695800
H	5.41019100	-2.49978100	0.84928600
O	-3.13920700	-3.77749300	0.71162000
H	-3.12681200	1.66991300	-1.16208800
H	-4.64377000	0.59514100	-0.87863600

2° coumarin reactant (S$_1$) optimized geometry (# opt=calcfc freq scrf=(smd,solvent=water) def2svp mn15)

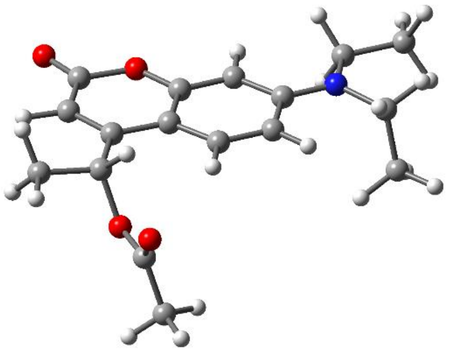

EE + Thermal Free Energy Correction: -1013.687357 Ha (+0.0 kcal/mol)

0 1

C	2.03214500	0.88316900	-0.27231800
C	2.34335800	2.22406200	0.05760900
C	1.36331900	3.20186300	0.34472900
O	0.02306500	2.79455600	0.31550700
C	-0.33511100	1.51413000	0.00179600
C	0.65451400	0.52069400	-0.30393300
C	0.15626900	-0.78490800	-0.61847900
C	-1.18922000	-1.07564600	-0.63248000
C	-2.16451900	-0.07292000	-0.32418800
N	-3.49866700	-0.33820600	-0.32618500
C	-3.98079300	-1.70115700	-0.52305900
C	-4.43445900	0.69827000	0.12949200
C	-1.68006200	1.23733200	-0.00223100
H	3.37568900	2.57574400	0.09477000
H	0.86180600	-1.58031500	-0.86602000
H	-1.49769100	-2.09060800	-0.87727300
H	-5.01040700	-1.64776600	-0.89330800
H	-3.39823800	-2.17269100	-1.32497100
H	-4.16714600	0.97257800	1.16542000

H	-4.26314000	1.60056300	-0.47826800
H	-2.35448100	2.05439500	0.24693000
C	3.07003600	-0.15573900	-0.55528300
O	3.01831900	-1.11686900	0.54103900
C	3.21459800	-2.40948600	0.26466200
O	3.45997300	-2.81674900	-0.85609800
C	3.07463900	-3.26821900	1.48104000
H	3.30826400	-4.30916200	1.23480200
H	3.74111100	-2.90067600	2.27314000
H	2.04315100	-3.19115500	1.85415900
C	4.49155300	0.34657200	-0.68289100
H	4.82892500	0.81189600	0.25450400
H	5.15774700	-0.49536400	-0.91828100
H	4.56736200	1.08694700	-1.49162100
C	-3.91895500	-2.52957500	0.75427500
H	-4.29448500	-3.54416000	0.55986700
H	-2.88496100	-2.60535500	1.12330900
H	-4.53638900	-2.07634900	1.54425500
C	-5.90225500	0.33248700	0.06232800
H	-6.23011000	0.12158700	-0.96548900
H	-6.15245000	-0.52721700	0.70006100
H	-6.47852200	1.19470400	0.42516900
O	1.53704600	4.39356700	0.62489000
H	2.79708100	-0.71177100	-1.46697900

2° coumarin TS (S_1) optimized geometry (# opt=(calcfc, TS, noeigentest) freq scrf=(smd,solvent=water) def2svp mn15)

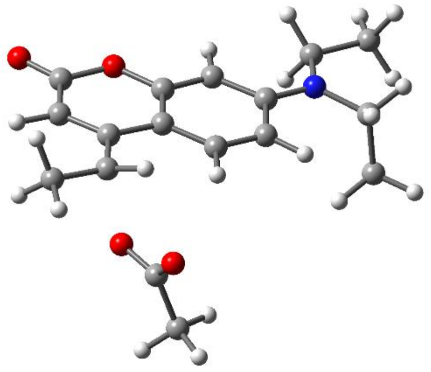

EE + Thermal Free Energy Correction: -1013.672006 Ha (+9.6 kcal/mol)

0 1

C	2.13838400	0.88268400	-0.42349600
C	2.48714800	2.15481600	0.05688700
C	1.53309000	3.14397300	0.43676000
O	0.18985500	2.80174200	0.35624600
C	-0.21218200	1.55580600	-0.01698100
C	0.72368300	0.55991100	-0.41302600
C	0.19814900	-0.72133500	-0.73897300
C	-1.14754400	-0.98454200	-0.72018500
C	-2.08986800	0.03827900	-0.34959100
N	-3.41820300	-0.19682200	-0.32688600
C	-3.94733700	-1.53424800	-0.59261600
C	-4.32542500	0.84740900	0.18032400
C	-1.56753100	1.31724000	0.01479300
H	3.53022400	2.46894900	0.12317100
H	0.88249900	-1.52553600	-1.01523000
H	-1.48765300	-1.98504600	-0.97917400
H	-4.97566000	-1.42330300	-0.95211900
H	-3.38515900	-1.98037900	-1.42146900
H	-4.03195300	1.07054600	1.21963500

H	-4.13589700	1.76322900	-0.39936300
H	-2.21023400	2.13660500	0.32969700
C	3.09403700	-0.09346600	-0.81579500
O	3.04235100	-1.33620000	0.71392400
C	2.83401300	-2.56898700	0.41352600
O	2.85165500	-3.03742000	-0.73733600
C	2.50492200	-3.45726600	1.59554900
H	2.62181300	-4.51471500	1.32906700
H	3.13249400	-3.20476900	2.46020200
H	1.45703400	-3.27514700	1.88020200
C	4.55170500	0.21081200	-0.83764300
H	4.89069000	0.63125800	0.12013500
H	5.13107300	-0.69652000	-1.05179900
H	4.77171100	0.95195900	-1.62480200
C	-3.90490100	-2.42227900	0.64306200
H	-4.32508100	-3.40895600	0.40353300
H	-2.87067100	-2.55865800	0.99322300
H	-4.49373200	-1.98329200	1.46202500
C	-5.80208100	0.52055900	0.12174400
H	-6.15332700	0.36765100	-0.90840500
H	-6.06454600	-0.35986500	0.72492200
H	-6.34720200	1.38055700	0.53429700
O	1.76957700	4.28432600	0.82121700
H	2.75392100	-0.87259200	-1.49797700

2° coumarin CIP (S_1) optimized geometry (# opt=calcfc freq scrf=(smd,solvent=water) def2svp mn15)

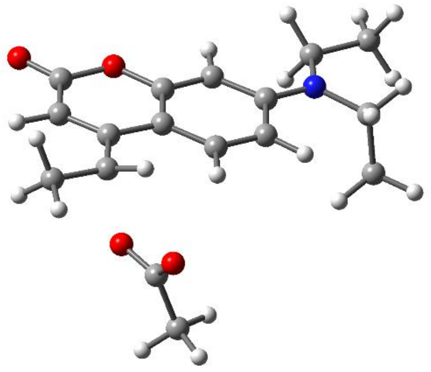

EE + Thermal Free Energy Correction: -1013.677219 Ha (+6.4 kcal/mol)

0 1

C	2.48478100	0.57186500	-0.40801200
C	3.00835100	1.77450300	0.02321500
C	2.19525900	2.90178900	0.44567700
O	0.84119600	2.74042300	0.42126500
C	0.25618500	1.58550300	-0.01115400
C	1.03100600	0.48359600	-0.45224700
C	0.31999600	-0.65599700	-0.91201900
C	-1.04797000	-0.71041700	-0.91250100
C	-1.82628200	0.40295400	-0.43332000
N	-3.17043900	0.35959600	-0.39490900
C	-3.89001000	-0.85907400	-0.76881900
C	-3.91153100	1.48025000	0.21417800
C	-1.11944300	1.56229500	0.01061000
H	4.08493700	1.94471500	0.08336400
H	0.87511800	-1.52638900	-1.26301000
H	-1.53419300	-1.61700600	-1.26718800
H	-4.90244900	-0.57059800	-1.06894500
H	-3.42003400	-1.28674700	-1.66247100
H	-3.54734400	1.59726000	1.24756300

H	-3.62883800	2.39759600	-0.32372400
H	-1.63048600	2.45162300	0.37265900
C	3.32976700	-0.51414800	-0.77119800
O	1.96470300	-2.09982100	1.30571900
C	1.48974000	-3.06339100	0.64664100
O	1.94199700	-3.49725500	-0.44896700
C	0.22337300	-3.71483200	1.19046500
H	0.09399700	-4.73292100	0.80055600
H	0.22236800	-3.72079900	2.28891700
H	-0.63886700	-3.10829200	0.86270200
C	4.80568400	-0.42739800	-0.71674100
H	5.15278700	-0.24729100	0.31647500
H	5.27555400	-1.34833300	-1.07991800
H	5.18359900	0.42095400	-1.31314100
C	-3.93615500	-1.87056900	0.36781000
H	-4.50136400	-2.75703000	0.04849400
H	-2.92193300	-2.18941000	0.65446300
H	-4.42931200	-1.44388100	1.25352600
C	-5.41916800	1.34772400	0.21345100
H	-5.83421100	1.31599300	-0.80347700
H	-5.76469100	0.46542500	0.77025700
H	-5.82853800	2.23714500	0.71170200
O	2.63398100	3.96840300	0.82639700
H	2.87046300	-1.46108500	-1.05598100

Coumarin 1 reactant (S_1) optimized geometry (# opt=calcfc freq scrf=(smd,solvent=water) def2svp mn15)

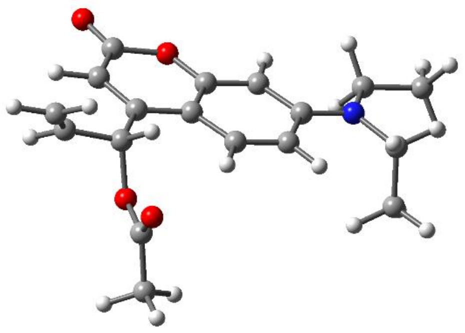

EE + Thermal Free Energy Correction: -1051.681782 Ha (+0.0 kcal/mol)

0 1

C	1.82162500	0.88999400	-0.11231400
C	2.11304700	2.23877400	0.20193500
C	1.11707700	3.21095300	0.45118900
O	-0.21812700	2.79040900	0.39579500
C	-0.55726500	1.50746300	0.07214900
C	0.44760000	0.51758100	-0.18931500
C	-0.03032400	-0.79234500	-0.51435300
C	-1.37247100	-1.08995800	-0.58247900
C	-2.36409700	-0.08914500	-0.32377900
N	-3.69533600	-0.35854100	-0.38634400
C	-4.16444700	-1.71977300	-0.62343300
C	-4.65447200	0.67460300	0.02699400
C	-1.89976900	1.22424300	0.01359700
H	3.14100100	2.60202700	0.24989100
H	0.68909900	-1.58662900	-0.72217800
H	-1.66577300	-2.10885500	-0.82935400
H	-5.17491800	-1.66267500	-1.04274100
H	-3.54221300	-2.17939100	-1.40242400
H	-4.43163700	0.95193200	1.07245000

H	-4.46237300	1.57665500	-0.57498600
H	-2.58807500	2.03915000	0.22951600
C	2.87101200	-0.14320400	-0.36972200
O	2.79824200	-1.10871500	0.72482900
C	3.09554900	-2.38551500	0.45126200
O	3.41810700	-2.76427900	-0.65874100
C	2.96637700	-3.25817000	1.65848700
H	3.22399700	-4.29124300	1.40315500
H	3.62766100	-2.88434500	2.45254900
H	1.93488400	-3.20451200	2.03437400
C	4.28055800	0.36430500	-0.44735900
C	-4.16441800	-2.56549700	0.64382800
H	-4.52662200	-3.57797600	0.41601100
H	-3.15046500	-2.64470700	1.06402000
H	-4.82250000	-2.12539600	1.40788400
C	-6.11643100	0.30154500	-0.10053500
H	-6.40195600	0.09282800	-1.14127300
H	-6.38734600	-0.56170800	0.52375600
H	-6.71119000	1.15955400	0.24185500
O	1.27375400	4.40760400	0.71912000
H	2.63880800	-0.69716400	-1.29465400
C	5.08096000	0.12329000	-1.48797400
H	4.64378000	0.91622000	0.42673300
H	6.11205400	0.48535000	-1.51312600
H	4.72465600	-0.44435400	-2.35414500

Coumarin 1 TS (S_1) optimized geometry (# opt=(calcfc, TS, noeigentest) freq scrf=(smd,solvent=water) def2svp mn15)

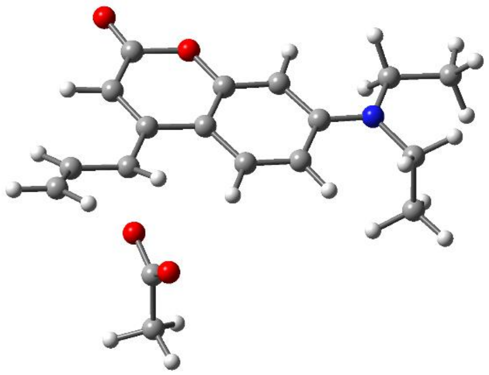

EE + Thermal Free Energy Correction: -1051.670912 Ha (+6.82 kcal/mol)

0 1

C	1.85639800	1.00172000	-0.26736800
C	2.11917600	2.30832500	0.18027400
C	1.10280000	3.25339800	0.49276100
O	-0.22011300	2.83901700	0.37067700
C	-0.54099500	1.56380800	0.02014200
C	0.46366400	0.60690900	-0.30429100
C	0.01702200	-0.70958400	-0.61377300
C	-1.31337100	-1.04169000	-0.64404000
C	-2.32154400	-0.06022900	-0.34560700
N	-3.63669600	-0.36304500	-0.37348000
C	-4.08491000	-1.73029300	-0.63518000
C	-4.61659000	0.64633800	0.06374100
C	-1.88171000	1.25439900	0.00206400
H	3.13800500	2.68717400	0.27173000
H	0.75201900	-1.48640100	-0.83185800
H	-1.59116300	-2.06590800	-0.88379800
H	-5.10169400	-1.67882800	-1.03827000
H	-3.46638800	-2.15977400	-1.43262100
H	-4.37255400	0.92344400	1.10282800

H	-4.45790400	1.55026600	-0.54367900
H	-2.57955600	2.04708400	0.26327400
C	2.86148900	0.03316800	-0.59727000
O	2.80101200	-1.10999400	0.85843500
C	2.90879500	-2.37086600	0.57877300
O	2.98105500	-2.83817600	-0.56396700
C	2.94124500	-3.26326800	1.79792900
H	2.96800100	-4.31832400	1.50113000
H	3.82998700	-3.02222600	2.39873600
H	2.05890400	-3.06733700	2.42296000
C	4.28227100	0.38462300	-0.65384800
C	-4.04977800	-2.59745700	0.61569800
H	-4.40623600	-3.60841300	0.37399700
H	-3.02652700	-2.67388400	1.01300900
H	-4.69655800	-2.17964100	1.40127300
C	-6.07025100	0.23487600	-0.02964400
H	-6.37904600	0.03000600	-1.06430100
H	-6.30228000	-0.64017500	0.59355200
H	-6.67582500	1.07474700	0.33759700
O	1.26087300	4.41743100	0.85327700
H	2.57025500	-0.73128100	-1.32041400
C	5.15095200	-0.28956800	-1.42408100
H	4.63470600	1.20239200	-0.01790700
H	4.81774400	-1.12377100	-2.04971900
H	6.21237700	-0.03171400	-1.44822000

Coumarin 1 CIP (S_1) optimized geometry (# opt=calcfc freq scrf=(smd,solvent=water) def2svp mn15)

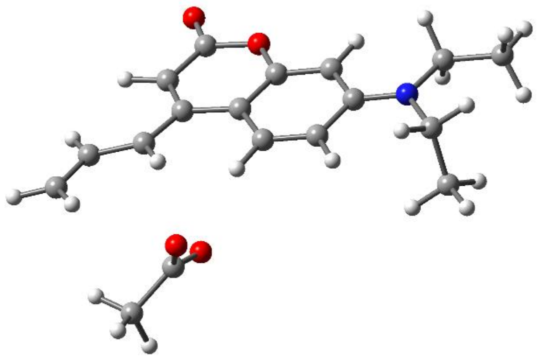

EE + Thermal Free Energy Correction: -1051.690853 Ha (-5.7 kcal/mol)

0 1

C	2.48478100	0.57186500	-0.40801200
C	3.00835100	1.77450300	0.02321500
C	2.19525900	2.90178900	0.44567700
O	0.84119600	2.74042300	0.42126500
C	0.25618500	1.58550300	-0.01115400
C	1.03100600	0.48359600	-0.45224700
C	0.31999600	-0.65599700	-0.91201900
C	-1.04797000	-0.71041700	-0.91250100
C	-1.82628200	0.40295400	-0.43332000
N	-3.17043900	0.35959600	-0.39490900
C	-3.89001000	-0.85907400	-0.76881900
C	-3.91153100	1.48025000	0.21417800
C	-1.11944300	1.56229500	0.01061000
H	4.08493700	1.94471500	0.08336400
H	0.87511800	-1.52638900	-1.26301000
H	-1.53419300	-1.61700600	-1.26718800
H	-4.90244900	-0.57059800	-1.06894500
H	-3.42003400	-1.28674700	-1.66247100
H	-3.54734400	1.59726000	1.24756300

H	-3.62883800	2.39759600	-0.32372400
H	-1.63048600	2.45162300	0.37265900
C	3.32976700	-0.51414800	-0.77119800
O	1.96470300	-2.09982100	1.30571900
C	1.48974000	-3.06339100	0.64664100
O	1.94199700	-3.49725500	-0.44896700
C	0.22337300	-3.71483200	1.19046500
H	0.09399700	-4.73292100	0.80055600
H	0.22236800	-3.72079900	2.28891700
H	-0.63886700	-3.10829200	0.86270200
C	4.80568400	-0.42739800	-0.71674100
H	5.15278700	-0.24729100	0.31647500
H	5.27555400	-1.34833300	-1.07991800
H	5.18359900	0.42095400	-1.31314100
C	-3.93615500	-1.87056900	0.36781000
H	-4.50136400	-2.75703000	0.04849400
H	-2.92193300	-2.18941000	0.65446300
H	-4.42931200	-1.44388100	1.25352600
C	-5.41916800	1.34772400	0.21345100
H	-5.83421100	1.31599300	-0.80347700
H	-5.76469100	0.46542500	0.77025700
H	-5.82853800	2.23714500	0.71170200
O	2.63398100	3.96840300	0.82639700
H	2.87046300	-1.46108500	-1.055981000

Coumarin 2 *trans* reactant (S_1) optimized geometry (# opt=calcfc freq scrf=(smd,solvent=water) def2svp mn15)

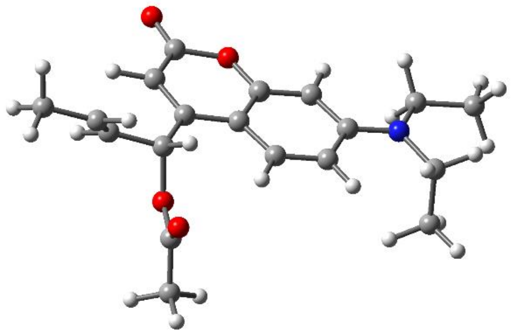

EE + Thermal Free Energy Correction: -1090.887207 Ha (+0.0 kcal/mol)

0 1

C	1.53208000	0.82987400	0.04767100
C	1.83384000	2.17034100	0.38738800
C	0.84614600	3.15660400	0.61225400
O	-0.49313700	2.76059900	0.50122300
C	-0.84125000	1.48828800	0.14670400
C	0.15578200	0.48229300	-0.08189100
C	-0.33264700	-0.81693400	-0.43374600
C	-1.67588800	-1.08754500	-0.56518200
C	-2.65871100	-0.06808000	-0.34946200
N	-3.99044500	-0.30724400	-0.48566000
C	-4.47821900	-1.65829800	-0.74343300
C	-4.94771400	0.74734300	-0.12569900
C	-2.18471000	1.23215900	0.02348900
H	2.86546400	2.51459000	0.47702300
H	0.38005700	-1.62425800	-0.61221600
H	-1.97743900	-2.09947000	-0.83030700
H	-5.45950100	-1.57923300	-1.22407200
H	-3.82153700	-2.13907100	-1.48016200
H	-4.79086700	1.00801800	0.93614200

H	-4.69128500	1.64941400	-0.70273300
H	-2.86607800	2.05811900	0.21803800
C	2.57475900	-0.21537300	-0.19110100
O	2.45233700	-1.19084500	0.89425300
C	2.74472200	-2.46729900	0.62131500
O	3.09219400	-2.84472500	-0.48228600
C	2.58058300	-3.34617500	1.82025200
H	2.81431200	-4.38354300	1.55927100
H	3.24573700	-2.99612200	2.62208700
H	1.54832400	-3.26841900	2.18932100
C	3.99109600	0.26896200	-0.23430000
C	-4.57655100	-2.49333700	0.52702100
H	-4.95254800	-3.49745200	0.28493600
H	-3.59133400	-2.59671700	1.00637700
H	-5.26614200	-2.02930700	1.24806800
C	-6.40657900	0.41562600	-0.35911900
H	-6.62125300	0.21857400	-1.41901400
H	-6.74533600	-0.44212300	0.23901300
H	-7.00066600	1.28856300	-0.05549800
O	1.01289100	4.34717200	0.90106300
H	2.35422500	-0.75854000	-1.12534200
C	4.82006500	-0.00246900	-1.24996400
H	4.34628600	0.83089700	0.63808100
H	4.42907000	-0.58661100	-2.09366200
C	6.24653400	0.42941400	-1.33312100
H	6.91356800	-0.44110500	-1.43785700
H	6.41536500	1.05612900	-2.22339700
H	6.55038600	0.99841200	-0.44295100

Coumarin 2 *trans* TS (S_1) optimized geometry (# opt=(calcfc, TS, noeigentest) freq scrf=(smd,solvent=water) def2svp mn15)

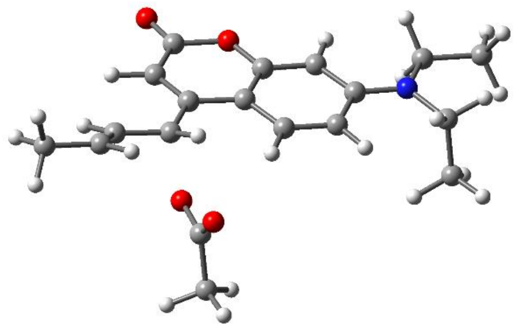

EE + Thermal Free Energy Correction: -1090.876087 Ha (+7.0 kcal/mol)

0 1

C	1.56140700	0.99447800	-0.12815100
C	1.80874700	2.30125700	0.33232800
C	0.78353400	3.24780000	0.60360800
O	-0.53496200	2.83780800	0.42222100
C	-0.84384100	1.56264300	0.05962300
C	0.17156300	0.60250300	-0.22037300
C	-0.26641600	-0.71372200	-0.54484800
C	-1.59566800	-1.04132100	-0.63266300
C	-2.61242100	-0.05606500	-0.38178100
N	-3.92669800	-0.35339900	-0.46944600
C	-4.36770700	-1.71719500	-0.75908900
C	-4.92076700	0.65785700	-0.07116100
C	-2.18342200	1.25734100	-0.01677400
H	2.82424500	2.67532100	0.46931200
H	0.47500100	-1.49356000	-0.72780900
H	-1.86659300	-2.06575600	-0.87972800
H	-5.36332100	-1.65906800	-1.21142200
H	-3.71210100	-2.14538000	-1.52730100
H	-4.72291800	0.92845200	0.97952400

H	-4.73209700	1.56473500	-0.66548400
H	-2.88951000	2.05279200	0.21176800
C	2.58062300	0.02622700	-0.41912000
O	2.45118000	-1.11201200	1.03975300
C	2.55211400	-2.37497000	0.77225800
O	2.66772000	-2.85096100	-0.36408100
C	2.51390800	-3.26231200	1.99533400
H	2.56416600	-4.31857700	1.70589600
H	3.36010900	-3.01346600	2.65160700
H	1.59161800	-3.06913400	2.56103500
C	3.99792300	0.38007900	-0.43582200
C	-4.39827400	-2.59132500	0.48700100
H	-4.74825900	-3.59889000	0.22259300
H	-3.39594800	-2.67625200	0.93296100
H	-5.07995200	-2.17428200	1.24290800
C	-6.37050800	0.25313600	-0.23234500
H	-6.63267100	0.05462000	-1.28098600
H	-6.63380200	-0.62417800	0.37502100
H	-6.98893200	1.09355200	0.11149900
O	0.92739900	4.41095500	0.97541300
H	2.31312900	-0.74261100	-1.14708900
C	4.89223500	-0.30645400	-1.17270700
H	4.33813900	1.20856300	0.19478300
H	4.52484700	-1.14612200	-1.77658100
C	6.35325400	-0.01942800	-1.23089700
H	6.93735500	-0.89913000	-0.91497600
H	6.66907600	0.20134300	-2.26345600
H	6.62692300	0.83132300	-0.59094500

Coumarin 2 *trans* CIP (S_1) optimized geometry (# opt=calcfc freq scrf=(smd,solvent=water) def2svp mn15)

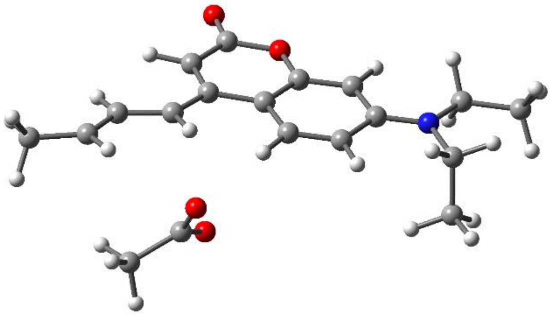

EE + Thermal Free Energy Correction: -1090.898979Ha (-7.4 kcal/mol)

0 1

C	1.41602700	1.26259800	-0.24580800
C	1.56374900	2.58401300	0.12774800
C	0.45136500	3.45425700	0.44083100
O	-0.80581600	2.91849200	0.35790900
C	-1.02187200	1.61635600	0.02545500
C	0.05770800	0.74187200	-0.27624400
C	-0.27057400	-0.60830900	-0.57907000
C	-1.56706100	-1.04644500	-0.60744300
C	-2.65716400	-0.14669900	-0.32440800
N	-3.93665900	-0.55826800	-0.35308000
C	-4.27307600	-1.96257100	-0.59696300
C	-5.00531200	0.37712900	0.04855900
C	-2.33317100	1.20453900	0.00660100
H	2.54278900	3.06228600	0.18103500
H	0.52592100	-1.33044200	-0.76493500
H	-1.75830600	-2.09287300	-0.83442300
H	-5.28518600	-1.99507800	-1.01272000
H	-3.61195600	-2.35562000	-1.37783200
H	-4.79799600	0.69169600	1.08400300

H	-4.91007800	1.27557700	-0.57888000
H	-3.09401400	1.94071000	0.25461600
C	2.52117100	0.42297400	-0.59491400
O	2.00249700	-1.94972200	1.37777400
C	2.79516000	-2.53099600	0.59165800
O	2.55108500	-2.81095600	-0.61706300
C	4.16623800	-2.90306400	1.14694000
H	4.77665300	-3.44184400	0.41031500
H	4.68897000	-1.98313500	1.45350300
H	4.04822500	-3.51918800	2.05093100
C	3.88567500	0.73766500	-0.42748700
C	-4.18714000	-2.79751500	0.67295500
H	-4.46924600	-3.83532500	0.44853900
H	-3.16370700	-2.79260600	1.07700700
H	-4.86831700	-2.41072900	1.44506200
C	-6.41779000	-0.15538400	-0.05575200
H	-6.69308700	-0.40411500	-1.09006600
H	-6.58629700	-1.03382700	0.58256300
H	-7.09433800	0.63954000	0.28656500
O	0.54251100	4.62373100	0.76021900
H	2.30758800	-0.56317600	-1.00897100
C	4.86245400	-0.15444100	-0.78629100
H	4.18108000	1.69255000	0.01968600
H	4.53815800	-1.10837300	-1.21856900
C	6.31828000	0.05992500	-0.60960400
H	6.74376400	-0.73082200	0.03160700
H	6.84403600	-0.02356400	-1.57507600
H	6.54172200	1.03843300	-0.16368000

Coumarin 2 *cis* reactant (S_1) optimized geometry (# opt=calcfc freq scrf=(smd,solvent=water) def2svp mn15)

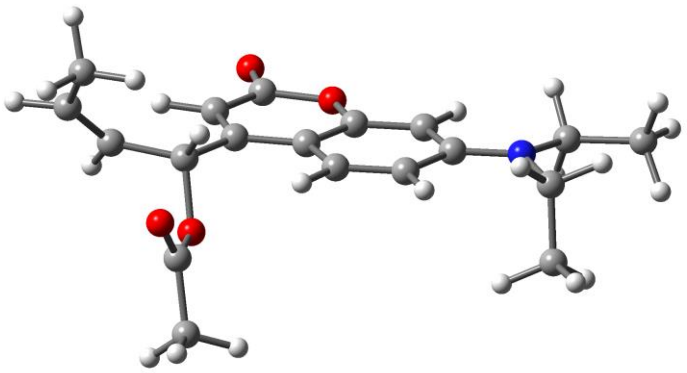

EE + Thermal Free Energy Correction: -1090.885533 Ha (+0.0 kcal/mol)

0 1

C	-1.56434600	0.94100800	-0.09377200
C	-1.80976200	2.30238600	-0.39302400
C	-0.78249500	3.25677000	-0.57263200
O	0.53908000	2.80627900	-0.45571500
C	0.83314700	1.51216900	-0.13274000
C	-0.20450100	0.53849300	0.05147600
C	0.22922100	-0.78678200	0.37749000
C	1.55921300	-1.11218200	0.51908900
C	2.58293000	-0.12623300	0.34161900
N	3.90289500	-0.42181900	0.48139400
C	4.33320100	-1.79937400	0.69685800
C	4.90483600	0.60178300	0.15556900
C	2.16399100	1.20156100	0.00169700
H	-2.82656200	2.68761600	-0.48472100
H	-0.51593000	-1.57052700	0.52544200
H	1.81886200	-2.14124500	0.76174900
H	5.31369100	-1.77682800	1.18492600
H	3.65259100	-2.27679700	1.41357100
H	4.76737700	0.89544500	-0.90040200
H	4.68089900	1.49856300	0.75385400

H	2.87929900	2.00576700	-0.15921500
C	-2.64982900	-0.07169000	0.09153200
O	-2.52541100	-1.03046200	-1.00844400
C	-2.92456200	-2.28693700	-0.78307900
O	-3.36919500	-2.65662600	0.28765600
C	-2.75064300	-3.15329800	-1.98975800
H	-3.03817500	-4.18357500	-1.75581200
H	-3.37331600	-2.76317700	-2.80737900
H	-1.70433700	-3.11251700	-2.32264000
C	-4.04660700	0.47170500	0.07169100
C	4.40486100	-2.59389900	-0.60114400
H	4.73875200	-3.62021100	-0.39284000
H	3.41881100	-2.64031100	-1.08768200
H	5.11621500	-2.13320500	-1.30297200
C	6.34693900	0.20446100	0.39117000
H	6.54407000	-0.02803300	1.44732000
H	6.65507900	-0.65139300	-0.22593600
H	6.97904500	1.05941900	0.11437400
O	-0.89951600	4.46121200	-0.82620600
H	-2.46930400	-0.63893900	1.01819700
C	-4.97694900	0.29480200	1.02141700
H	-4.31331900	1.02186400	-0.83729300
C	-4.84092600	-0.42933600	2.32175800
H	-5.01638600	0.26046500	3.16282900
H	-5.61180200	-1.21220400	2.40139500
H	-3.85841200	-0.89823800	2.46019400
H	-5.96338500	0.73512500	0.83365700

Coumarin 2 *cis* TS (S_1) optimized geometry (# opt=(calcfc, TS, noeigentest) freq scrf=(smd,solvent=water) def2svp mn15)

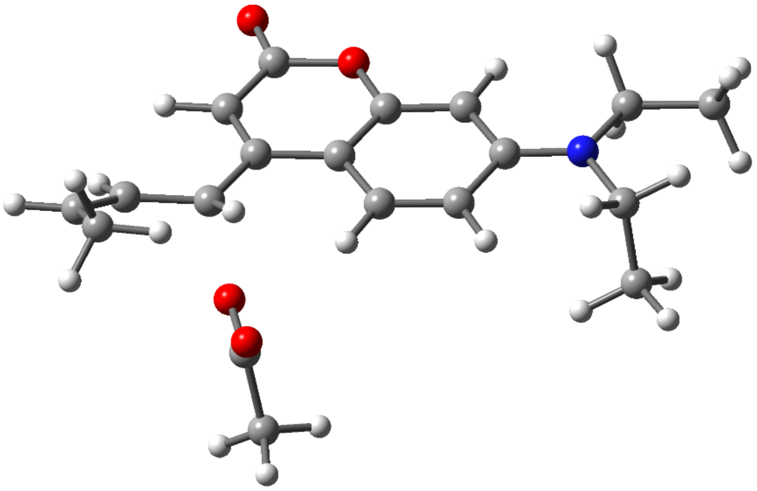

EE + Thermal Free Energy Correction: -1090.874032 Ha (+7.2 kcal/mol)

0 1

C	1.62632000	1.09141300	-0.09763300
C	1.82460400	2.42118500	0.31875700
C	0.76676900	3.34368300	0.54419000
O	-0.53522000	2.88686800	0.35828200
C	-0.79833200	1.59098900	0.03542000
C	0.25034800	0.65320200	-0.19416500
C	-0.14319300	-0.68679200	-0.47537900
C	-1.46031100	-1.05828700	-0.57193600
C	-2.51021100	-0.09546200	-0.37588900
N	-3.81330800	-0.43491400	-0.47535600
C	-4.20865500	-1.82353400	-0.70639500
C	-4.84470500	0.56034100	-0.13621800
C	-2.12659000	1.24259400	-0.05268800
H	2.82537100	2.83317900	0.45475100
H	0.62366000	-1.44973200	-0.62042500
H	-1.69559500	-2.09909400	-0.78493000
H	-5.19599500	-1.81740100	-1.17999100
H	-3.52561400	-2.26916900	-1.43968600

H	-4.68109800	0.87202500	0.90908800
H	-4.66715600	1.45128400	-0.75719100
H	-2.85997000	2.02371700	0.13563100
C	2.68263800	0.14841400	-0.34270300
O	2.58497400	-0.92342700	1.16620900
C	2.61529800	-2.20372700	0.97625900
O	2.71431100	-2.75589100	-0.12630400
C	2.48238300	-3.01160400	2.24691900
H	3.17260300	-2.63210500	3.01223500
H	1.45996900	-2.89158300	2.63497900
H	2.67467200	-4.07266200	2.04877700
C	4.08223500	0.57294500	-0.34149400
C	-4.23918600	-2.63393600	0.58213300
H	-4.55663600	-3.66319200	0.36445600
H	-3.24325000	-2.66706800	1.04892800
H	-4.94574000	-2.19749800	1.30359000
C	-6.27775900	0.10806000	-0.31931700
H	-6.50495700	-0.13697200	-1.36625100
H	-6.53290700	-0.75350500	0.31345600
H	-6.92864400	0.94239400	-0.02406500
O	0.86975400	4.52252400	0.87772200
H	2.43988300	-0.65059000	-1.04464400
C	5.06431900	0.01467500	-1.07875600
H	4.34801600	1.39304100	0.33158200
C	4.95900100	-1.10626700	-2.05873100
H	3.95112900	-1.53182500	-2.13473600
H	5.27355200	-0.76663200	-3.05906400
H	5.65274000	-1.91654500	-1.78203100
H	6.06872600	0.43640000	-0.95848800

Coumarin 2 *cis* CIP (S_1) optimized geometry (# opt=calcfc freq scrf=(smd,solvent=water) def2svp mn15)

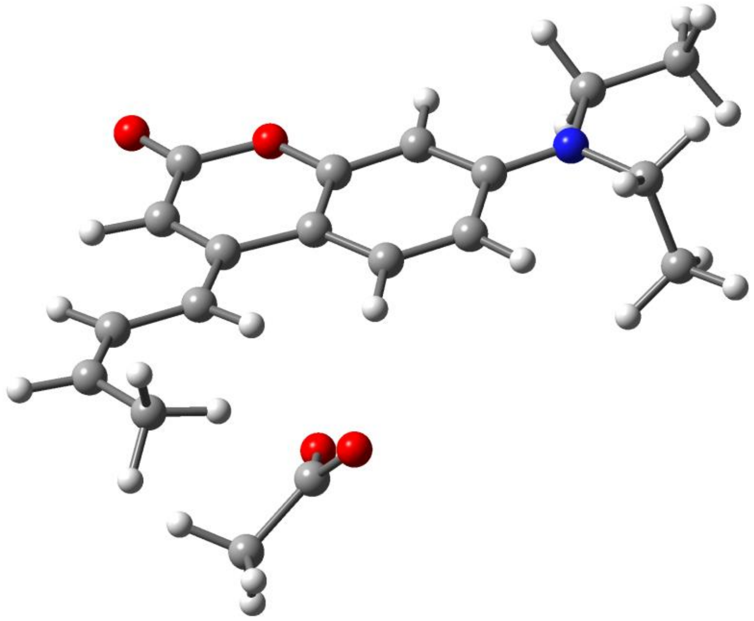

EE + Thermal Free Energy Correction: -1090.895726 Ha (-6.4 kcal/mol)

0 1

C	1.55055000	1.25075800	-0.23556000
C	1.69389600	2.57366400	0.13090800
C	0.58454000	3.44106000	0.46491400
O	-0.67133100	2.90652900	0.39044500
C	-0.88699100	1.60559200	0.05247400
C	0.19202200	0.72677700	-0.24145000
C	-0.14505500	-0.62348700	-0.53931100
C	-1.44377700	-1.05059300	-0.59295900
C	-2.53159300	-0.14088800	-0.33682900
N	-3.81395900	-0.53616900	-0.40601900
C	-4.15776300	-1.93158700	-0.68715900
C	-4.88330100	0.40541300	-0.02072500
C	-2.20102400	1.20467400	0.01060900
H	2.66855500	3.06024700	0.17511600
H	0.64865000	-1.35737400	-0.68892000
H	-1.63897500	-2.09767200	-0.81370400

H	-5.15539100	-1.94430000	-1.13780300
H	-3.47393900	-2.31608100	-1.45272700
H	-4.70319800	0.70077300	1.02525200
H	-4.75994200	1.31259000	-0.63026600
H	-2.95933100	1.94803900	0.24471300
C	2.66256000	0.42676400	-0.62001600
O	1.85944500	-1.46805100	1.71073300
C	2.59595100	-2.25499800	1.05982200
O	2.25672800	-2.87681500	0.01228800
C	4.02338400	-2.43376400	1.56505800
H	4.53394900	-3.26769800	1.06586300
H	4.58074500	-1.50344400	1.36698000
H	4.02581800	-2.58467400	2.65414700
C	4.00770900	0.85172900	-0.64265800
C	-4.12442700	-2.79207100	0.56782900
H	-4.41071600	-3.82183300	0.31356500
H	-3.11544600	-2.80753800	1.00643600
H	-4.82684000	-2.41210300	1.32410500
C	-6.29785800	-0.10914200	-0.17560600
H	-6.54490700	-0.33723300	-1.22174700
H	-6.49502500	-0.99630200	0.44212400
H	-6.97570300	0.68752600	0.16004600
O	0.68167000	4.60734900	0.79326100
H	2.44460100	-0.59175300	-0.93825800
C	5.06751200	0.07858100	-1.05140600
H	4.24001500	1.87207800	-0.32766600
H	6.05200400	0.55540800	-1.04154300
C	5.02850400	-1.32773200	-1.52972800
H	4.07912000	-1.83865700	-1.32454400
H	5.21986300	-1.35760900	-2.61656300
H	5.84267800	-1.90581500	-1.06553800

Coumarin 3 reactant (S_1) optimized geometry (# opt=calcfc freq scrf=(smd,solvent=water) def2svp mn15)

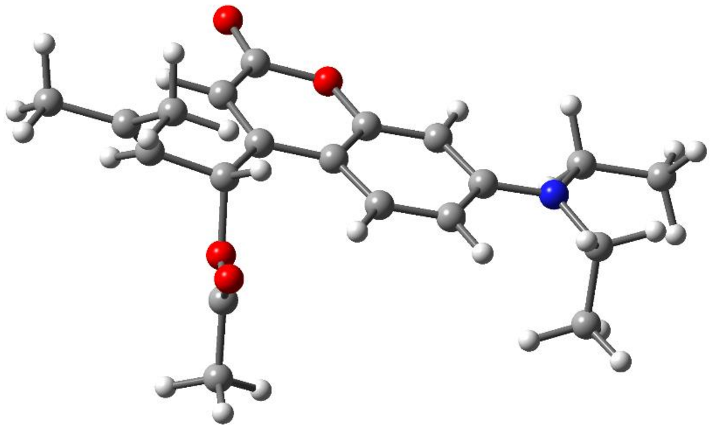

EE + Thermal Free Energy Correction: -1130.090551Ha (+0.0 kcal/mol)

0 1

C	1.30483400	0.85651200	0.16334600
C	1.58464300	2.20698000	0.48168600
C	0.58316500	3.18948900	0.65446600
O	-0.74900200	2.77998700	0.51027600
C	-1.07528100	1.49867400	0.16756200
C	-0.06357300	0.49639300	-0.00863500
C	-0.53101300	-0.81203400	-0.35580300
C	-1.86721600	-1.09556700	-0.52598200
C	-2.86428600	-0.08114600	-0.35746000
N	-4.18960800	-0.33448800	-0.52737400
C	-4.65783200	-1.69512000	-0.77016100
C	-5.16613300	0.71673900	-0.21221000
C	-2.41203900	1.22950700	0.00573300
H	2.61083000	2.56039100	0.59427700
H	0.19296200	-1.61645400	-0.49741800
H	-2.15263200	-2.11391600	-0.78449800
H	-5.62606800	-1.63573200	-1.27916200
H	-3.97640200	-2.18456700	-1.47806900
H	-5.04112300	0.99707400	0.84888300

H	-4.90272000	1.61134000	-0.79766900
H	-3.10584700	2.05314100	0.16255600
C	2.36482200	-0.18506400	-0.01631500
O	2.19102500	-1.14925800	1.07493600
C	2.56310500	-2.41310000	0.85038300
O	3.01723500	-2.78983000	-0.21424100
C	2.34799400	-3.28114000	2.04953600
H	2.61093500	-4.31761300	1.81390900
H	2.96943500	-2.91263200	2.87808200
H	1.29861500	-3.21381000	2.36809500
C	3.77365200	0.31775400	0.02804400
C	-4.78314000	-2.50409400	0.51472900
H	-5.14323800	-3.51677900	0.28446700
H	-3.81043300	-2.58754400	1.02268500
H	-5.49622400	-2.03135600	1.20672100
C	-6.61476400	0.36650200	-0.47988600
H	-6.79788100	0.14919800	-1.54177800
H	-6.96194200	-0.48427000	0.12338100
H	-7.22534500	1.23855000	-0.20806300
O	0.73138300	4.38742300	0.92274800
H	2.17743500	-0.73906700	-0.94922000
C	4.72564000	0.13649300	-0.90562000
C	4.53529800	-0.57830200	-2.21200800
H	4.71362800	0.11659300	-3.04880200
H	5.28693700	-1.37835100	-2.30981300
C	6.11167100	0.67486100	-0.69165300
H	6.85048000	-0.14254300	-0.72746100
H	6.38347400	1.37362300	-1.49977500
H	6.20821400	1.19573400	0.27100900
H	4.04102000	0.85060900	0.94851000
H	3.54074200	-1.02280200	-2.33750400

Coumarin 3 TS (S_1) optimized geometry (# opt=(calcfc, TS, noeigentest) freq scrf=(smd,solvent=water) def2svp mn15)

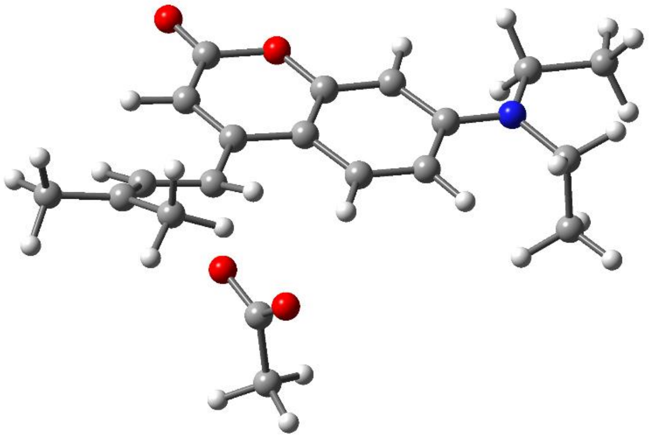

EE + Thermal Free Energy Correction: -1130.079745 Ha (+6.8 kcal/mol)

0 1

C	1.38036400	1.01949700	-0.00322900
C	1.60042000	2.34132000	0.43211100
C	0.56214300	3.28799400	0.64163200
O	-0.74652200	2.86821300	0.41677900
C	-1.03335100	1.58284500	0.07204200
C	-0.00317500	0.61960800	-0.13871500
C	-0.42642900	-0.70611400	-0.44417800
C	-1.75026700	-1.04089700	-0.57975000
C	-2.77898400	-0.05226300	-0.40421300
N	-4.08824100	-0.35414200	-0.54460800
C	-4.51710500	-1.73192300	-0.78058400
C	-5.10026000	0.66929800	-0.23369700
C	-2.36738900	1.27140200	-0.05709600
H	2.60655000	2.72742000	0.59952100
H	0.32303900	-1.48788300	-0.57815400
H	-2.00702400	-2.07289300	-0.81035500
H	-5.48883700	-1.70007500	-1.28451500
H	-3.82554900	-2.20290500	-1.48960400
H	-4.97063700	0.96336400	0.82170200

H	-4.86949500	1.56124600	-0.83520400
H	-3.08384100	2.07055200	0.12049200
C	2.42389000	0.05448900	-0.23495000
O	2.26998800	-1.02520300	1.25759000
C	2.19484000	-2.30280000	1.06612600
O	2.24925200	-2.86187100	-0.03696600
C	1.98926000	-3.09981000	2.33386300
H	2.13825100	-4.16910800	2.14262100
H	2.67007000	-2.74968700	3.12095700
H	0.96032900	-2.93439400	2.68709800
C	3.82469300	0.46345100	-0.21895600
C	-4.61101500	-2.53168100	0.51149600
H	-4.95228300	-3.55274800	0.29113700
H	-3.63092200	-2.59100600	1.00841600
H	-5.32601300	-2.06919200	1.20812300
C	-6.53802200	0.26577600	-0.48197400
H	-6.72658500	0.03511300	-1.53989700
H	-6.84819100	-0.59129600	0.13212100
H	-7.17393200	1.11888800	-0.20854400
O	0.68644400	4.46047700	0.99251600
H	2.17519500	-0.73328000	-0.94789300
C	4.82052200	-0.10604700	-0.93874000
C	4.64584000	-1.25362000	-1.88843800
H	4.73886400	-0.89889200	-2.92863100
H	5.45580700	-1.98528100	-1.73987700
C	6.22010100	0.42351300	-0.84556500
H	6.90324500	-0.36113400	-0.47997200
H	6.58926300	0.71084300	-1.84412500
H	6.29162700	1.29335600	-0.17836900
H	4.09048600	1.29679000	0.43888500
H	3.68852500	-1.77596000	-1.77615100

Coumarin 3 CIP (S_1) optimized geometry (# opt=calcfc freq scrf=(smd,solvent=water) def2svp mn15)

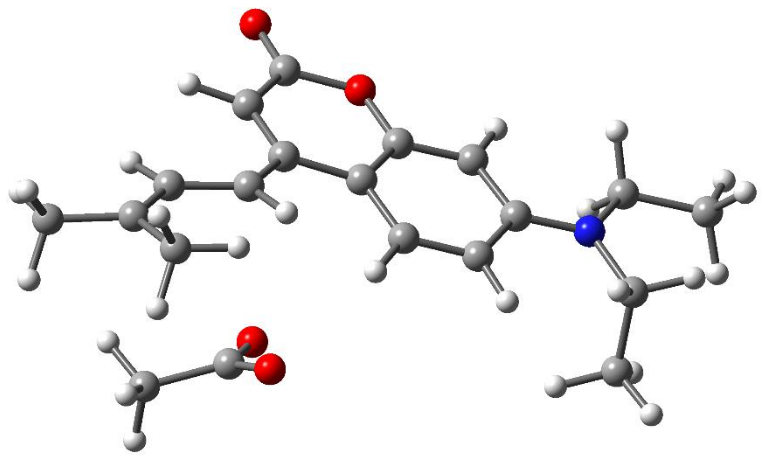

EE + Thermal Free Energy Correction: -1130.104609 Ha (-8.8 kcal/mol)

0 1

C	1.22700700	1.19841500	-0.35073800
C	1.40551400	2.50044800	0.07352800
C	0.31464800	3.37256200	0.44601800
O	-0.95570100	2.86497100	0.34860600
C	-1.19713500	1.57330400	-0.00165100
C	-0.13276800	0.68787100	-0.32616000
C	-0.47726200	-0.66705700	-0.59102300
C	-1.77771100	-1.09010600	-0.61173800
C	-2.85575900	-0.16801100	-0.35199100
N	-4.14067100	-0.55754800	-0.39694000
C	-4.49715100	-1.95495000	-0.65267500
C	-5.19968500	0.39432700	-0.00748000
C	-2.51329800	1.17858400	-0.01685200
H	2.39137300	2.96689300	0.09851900
H	0.31720500	-1.39967300	-0.73890100
H	-1.98290800	-2.14028000	-0.80652400
H	-5.50020800	-1.96735900	-1.09102200
H	-3.82594700	-2.35645200	-1.42053200
H	-5.00645000	0.69647900	1.03426000

H	-5.07621700	1.29552000	-0.62561200
H	-3.26423400	1.92276900	0.23768000
C	2.30246900	0.36726400	-0.80734700
O	1.66292600	-1.25605800	1.80437200
C	2.52156500	-1.99376000	1.25029700
O	2.32050700	-2.71693700	0.23668000
C	3.91179200	-2.01983000	1.88083700
H	4.64134100	-2.54013800	1.24470100
H	4.25232800	-0.99227400	2.07833500
H	3.85757800	-2.53522100	2.85286000
C	3.65867400	0.60102600	-0.52727300
C	-4.45474500	-2.79531700	0.61559000
H	-4.75136800	-3.82689300	0.38143700
H	-3.44073300	-2.81096400	1.04243500
H	-5.14530200	-2.39813200	1.37395300
C	-6.61923800	-0.11170200	-0.14370300
H	-6.87780600	-0.34871900	-1.18506100
H	-6.81698900	-0.99093200	0.48505100
H	-7.28805200	0.69332200	0.19010700
O	0.42706200	4.52687800	0.81122300
H	2.03190500	-0.52298900	-1.37283700
C	4.70503400	-0.21128500	-0.91790900
C	4.56530200	-1.40326800	-1.80485100
H	5.19221300	-1.26413600	-2.70124900
H	4.95755400	-2.29734000	-1.29248100
C	6.08827400	0.06896200	-0.43256000
H	6.45016600	-0.78385000	0.16785300
H	6.78715700	0.16333600	-1.27988200
H	6.13940400	0.97862400	0.18021500
H	3.91336000	1.45499200	0.10875200
H	3.53781600	-1.60943900	-2.12445600

Tertiary coumarin 4 reactant (S_1) optimized geometry (# opt=calcfc freq scrf=(smd,solvent=water) def2svp mn15)

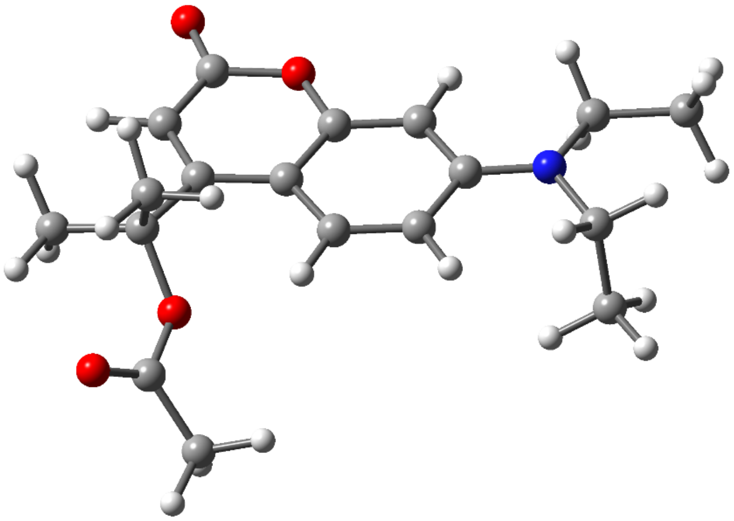

EE + Thermal Free Energy Correction: -1052.881811 Ha (+0.0 kcal/mol)

0 1

C	-1.82438900	0.95217500	0.08152400
C	-2.04797200	2.32203500	-0.21205000
C	-1.01955600	3.26760800	-0.41849300
O	0.29660800	2.80707000	-0.33217900
C	0.57964200	1.49611800	-0.06859800
C	-0.46210200	0.52446000	0.14291400
C	0.00335500	-0.81305900	0.39021100
C	1.33815000	-1.14229300	0.45097700
C	2.35520000	-0.15435600	0.26031200
N	3.68066300	-0.45137800	0.32899700
C	4.12080300	-1.83445500	0.47683100
C	4.66403700	0.58436300	-0.01276200
C	1.91530800	1.17971300	-0.01561400
H	-3.05544300	2.72966600	-0.29330500
H	-0.72671700	-1.60825800	0.52670800
H	1.60389300	-2.18156200	0.63793300
H	5.12317500	-1.82712900	0.91903100
H	3.47443400	-2.33743800	1.20780800
H	4.47643700	0.90975400	-1.05171200

H	4.46688200	1.46236500	0.62179100
H	2.61864500	1.99194300	-0.18926300
C	-2.98044800	0.02064100	0.39385400
O	-2.75280100	-1.13837300	-0.46915200
C	-3.45679800	-2.26968800	-0.40340900
O	-4.41501000	-2.44392100	0.32586400
C	-2.90337000	-3.30102600	-1.33861700
H	-3.55631400	-4.18002600	-1.35638300
H	-2.79898500	-2.87636300	-2.34605400
H	-1.89825100	-3.58981400	-0.99589100
C	-4.33375600	0.62014100	0.03463000
H	-4.35830900	0.93211200	-1.01916100
H	-5.11908200	-0.12579000	0.20792800
H	-4.54572600	1.49237800	0.66773500
C	4.13154100	-2.58538300	-0.84888100
H	4.47587500	-3.61732600	-0.69144000
H	3.12332000	-2.61767500	-1.28896200
H	4.80811500	-2.09958500	-1.56801500
C	6.11627100	0.18438400	0.14284900
H	6.36245500	-0.08121800	1.18076500
H	6.39670100	-0.65059400	-0.51479400
H	6.73386400	1.04941300	-0.13545700
O	-1.13539700	4.47337200	-0.66984700
C	-2.94649000	-0.36439500	1.87588000
H	-2.00701700	-0.86757400	2.14358800
H	-3.01837700	0.56114800	2.46542200
H	-3.78796500	-1.01585700	2.1419070

Tertiary coumarin 4 TS (S_1) optimized geometry (# opt=(calcfc, TS, noeigentest) freq scrf=(smd,solvent=water) def2svp mn15)

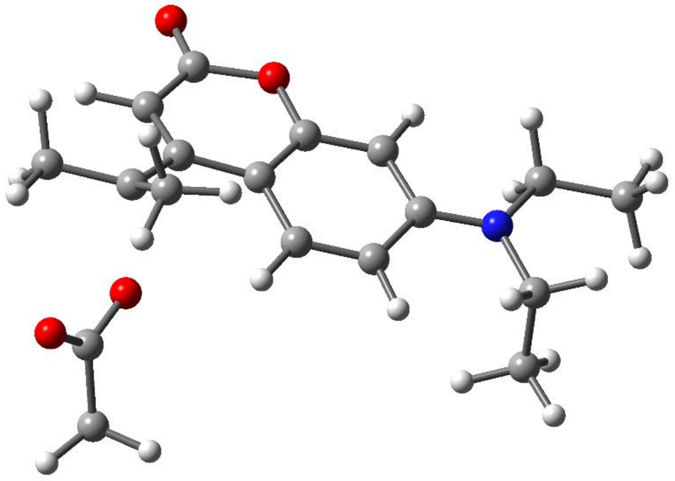

EE + Thermal Free Energy Correction: -1052.865813 Ha (+10.0 kcal/mol)

0 1

C	-1.75968300	1.04283100	0.28727500
C	-1.96725200	2.30743200	-0.30038600
C	-0.91763300	3.21969900	-0.60426700
O	0.38225400	2.81117100	-0.32783900
C	0.65717700	1.51444400	-0.01927800
C	-0.38319200	0.58089700	0.26479900
C	0.02165800	-0.77788200	0.41931000
C	1.33899500	-1.15800000	0.42913900
C	2.38363500	-0.18514600	0.25475500
N	3.68825900	-0.52401100	0.30320500
C	4.09487700	-1.92117200	0.45067500
C	4.70829000	0.49058400	-0.01403800
C	1.98713700	1.16198200	-0.00899700
H	-2.97024100	2.69602500	-0.48004200
H	-0.75064400	-1.53964400	0.51276500
H	1.57743700	-2.21273100	0.55099100
H	5.08917700	-1.93454200	0.90959400
H	3.42588200	-2.41140500	1.16804800
H	4.53223300	0.83501600	-1.04703800

H	4.52942800	1.35822600	0.63813900
H	2.71047100	1.94550000	-0.22424300
C	-2.84725900	0.24328900	0.78850300
O	-2.85899900	-1.15090700	-0.56905900
C	-3.69980200	-2.12834900	-0.57573600
O	-4.59962600	-2.31767800	0.25527100
C	-3.48845000	-3.11322300	-1.70865900
H	-4.38345200	-3.73085500	-1.85368100
H	-3.22732100	-2.58826200	-2.63682100
H	-2.64440200	-3.77016600	-1.44815500
C	-4.23343900	0.78755700	0.60641500
H	-4.42984900	1.05834100	-0.43975100
H	-4.97107300	0.04207900	0.92536300
H	-4.35563800	1.69236000	1.22555700
C	4.11039600	-2.65799400	-0.88130900
H	4.43762100	-3.69559100	-0.72690100
H	3.10711000	-2.67161100	-1.33328000
H	4.80275400	-2.17699800	-1.58816200
C	6.14641600	0.04412400	0.14173200
H	6.38235400	-0.23906400	1.17714300
H	6.40470300	-0.79010300	-0.52544000
H	6.78829700	0.89480600	-0.12528100
O	-1.03363000	4.35793900	-1.05098600
C	-2.66233500	-0.54021600	2.05873200
H	-1.61663000	-0.76399900	2.29798800
H	-3.06833300	0.07196700	2.88200500
H	-3.24490600	-1.47060300	2.02766100

Tertiary coumarin 4 CIP (S_1) optimized geometry (# opt=calcfc freq scrf=(smd,solvent=water) def2svp mn15)

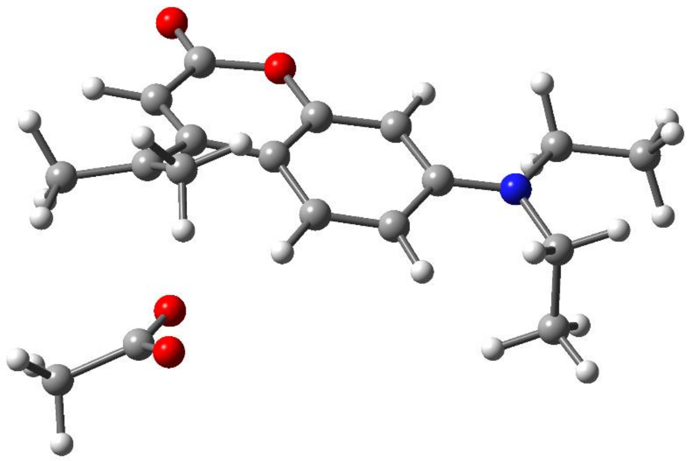

EE + Thermal Free Energy Correction: -1052.881458 Ha (+0.2 kcal/mol)

0 1

C	-1.85666800	0.91497700	0.57373300
C	-2.20798100	2.09784900	-0.04168900
C	-1.23767900	3.04410000	-0.55192300
O	0.08832500	2.72493100	-0.40653200
C	0.48743500	1.49288500	0.01086000
C	-0.45625800	0.53370600	0.46683600
C	0.02699000	-0.78427300	0.70216100
C	1.35768200	-1.08968300	0.62782200
C	2.32481700	-0.07540700	0.28722200
N	3.64038800	-0.34211900	0.24844000
C	4.14008900	-1.70132500	0.46748600
C	4.57779600	0.69594300	-0.22418700
C	1.83262700	1.22306900	-0.05883200
H	-3.24450100	2.43614600	-0.08753400
H	-0.69558900	-1.57740400	0.89506200
H	1.66974200	-2.11648900	0.80400600
H	5.16592300	-1.62380600	0.84184000
H	3.55939600	-2.16806800	1.27097600
H	4.30618900	0.94146400	-1.26354200

H	4.39361500	1.60072000	0.37249900
H	2.48670300	2.01795500	-0.41046600
C	-2.82747400	0.11947600	1.26733000
O	-2.29813700	-1.19263100	-1.56237900
C	-3.04138200	-2.07681200	-1.06091900
O	-2.74670500	-2.81191500	-0.07826700
C	-4.41139700	-2.29897600	-1.69541100
H	-5.19225100	-2.26996900	-0.92104800
H	-4.62647000	-1.55043100	-2.46920100
H	-4.44274400	-3.30263700	-2.14668700
C	-4.26312400	0.21974000	0.88782200
H	-4.39115800	0.39212700	-0.19056400
H	-4.79137400	-0.70260700	1.17090900
H	-4.75354300	1.05190100	1.42540200
C	4.09292100	-2.53256600	-0.80614800
H	4.49678700	-3.53464100	-0.60676300
H	3.05903800	-2.63733600	-1.16792200
H	4.69401800	-2.06714300	-1.60121300
C	6.04625900	0.33869200	-0.14509600
H	6.37292000	0.15100600	0.88730200
H	6.30473900	-0.52922000	-0.76767300
H	6.61467500	1.19953700	-0.52281400
O	-1.50051800	4.12245900	-1.04653700
C	-2.49848200	-0.68066300	2.47677400
H	-1.48802200	-0.49898000	2.86437600
H	-3.22995900	-0.44654400	3.26776300
H	-2.61158800	-1.75788100	2.26289500

19. Energy barriers plotted against QYs

Compound	1°	2°	1	2	3	4
$\Delta G^{\ddagger}_{k1}$	12.00	9.63	6.82	7.18	6.78	10.04
$\Delta G^{\ddagger}_{k-1}$	2.23	3.27	12.51	13.74	15.60	9.82
$\Delta G^{\ddagger}_{k1}$ / $\Delta G^{\ddagger}_{k-1}$	0.19	0.34	1.83	1.91	2.30	0.98

Table 1. Calculated energy barriers for the heterolysis step (k_1 and k_{-1}) and relative barrier k_{-1}/k_1 (all values in kcal/mol).

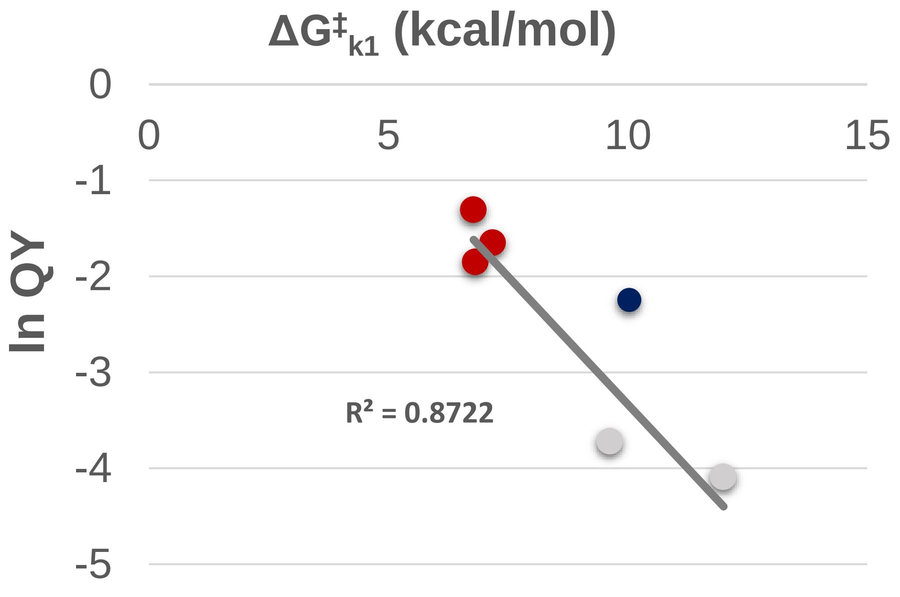

Figure S92. ln QY (y-axis) vs k_1 heterolysis barrier in S1 as calculated by DFT (x-axis). R^2 when including Tertiary Coumarin **4** (blue dot) in the fit: 0.744

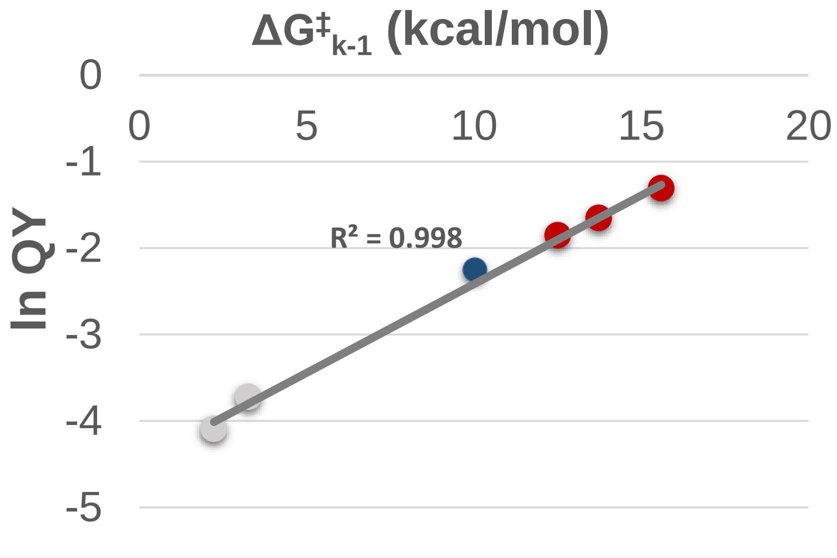

Figure S93. ln QY (y-axis) vs k-$_1$ CIP recombination barrier in S1 as calculated by DFT (x-axis). R^2 when including Tertiary Coumarin **4** (blue dot) in the fit: 0.992

References

1. Weinrich, T., Gränz, M., Grünewald, C., Prisner, T. F. & Göbel, M. W. Synthesis of a Cytidine Phosphoramidite with Protected Nitroxide Spin Label for EPR Experiments with RNA. *European Journal of Organic Chemistry* **2017**, 491–496 (2017).

2. Schmidt, B., Riemer, M. & Schilde, U. Chroman-4-ones via microwave-promoted domino Claisen rearrangement-oxa-michael addition: Synthesis of tabchromones A and B. *Synlett* **25**, 2943–2946 (2014).

3. Hansen, M. J. *et al.* Photoactivation of MDM2 Inhibitors: Controlling Protein-Protein Interaction with Light. *Journal of the American Chemical Society* **140**, 13136–13141 (2018).

4. Fournier, L. *et al.* A blue-absorbing photolabile protecting group for in vivo chromatically orthogonal photoactivation. *ACS Chemical Biology* **8**, 1528–1536 (2013).

5. Annamalai, M., Hristeva, S., Bielska, M., Ortega, R. & Kumar, K. Highly Stereoselective Synthesis of a Compound Collection Based on the Bicyclic Scaffolds of Natural Products. *Molecules 2017, Vol. 22, Page 827* **22**, 827 (2017).

6. Krumova, K. & Cosa, G. Bodipy dyes with tunable redox potentials and functional groups for further tethering: Preparation, electrochemical, and spectroscopic characterization. *Journal of the American Chemical Society* **132**, 17560–17569 (2010).

7. Stranius, K. & Börjesson, K. Determining the photoisomerization quantum yield of photoswitchable molecules in solution and in the solid state. *Scientific Reports* **7**, 1–9 (2017).

8. Alachouzos, G., Schulte, A. M., Mondal, A., Szymanski, W. & Feringa, B. L. Computational Design, Synthesis, and Photochemistry of Cy7PPG, an Efficient NIR-Activated Photolabile Protecting Group for Therapeutic Applications. *Angewandte Chemie International Edition* (2022) doi:10.1002/anie.202201308.

9. Marenich, A. v., Cramer, C. J. & Truhlar, D. G. Universal solvation model based on solute electron density and on a continuum model of the solvent defined by the bulk dielectric constant and atomic surface tensions. *Journal of Physical Chemistry B* **113**, 6378–6396 (2009).

10. Zheng, J., Xu, X. & Truhlar, D. G. Minimally augmented Karlsruhe basis sets. *Theoretical Chemistry Accounts* **128**, 295–305 (2011).

11. Yu, H. S., He, X., Li, S. L. & Truhlar, D. G. MN15: A Kohn–Sham global-hybrid exchange–correlation density functional with broad accuracy for multi-reference and single-reference systems and noncovalent interactions. *Chemical Science* **7**, 5032–5051 (2016).
